# A comparison of Landsat 8, RapidEye and Pleiades products for improving empirical predictions of satellite-derived bathymetry

**DOI:** 10.1016/j.rse.2019.111414

**Published:** 2019-11

**Authors:** C. Cahalane, A. Magee, X. Monteys, G. Casal, J. Hanafin, P. Harris

**Affiliations:** a Department of Geography, Rhetoric House, Maynooth University, Co. Kildare, Ireland; b National Centre for Geocomputation, Iontas, Maynooth University, Maynooth, Co. Kildare, Ireland; c Geological Survey Ireland, Beggars Bush, Haddington Road, Dublin 4 D04 K7X4, Ireland; d TechWorks Marine Ltd, Pottery Road Enterprise Centre, Dun Laoghaire, Co. Dublin, Ireland; e Sustainable Agriculture Systems, Rothamsted Research, North Wyke, Okehampton, Devon EX20 2 SB, UK

**Keywords:** Multispectral, Multi-platform, Geostatistics, LiDAR, Coastal

## Abstract

Satellite derived bathymetry (SDB) enables rapid mapping of large coastal areas through measurement of optical penetration of the water column. The resolution of bathymetric mapping and achievable horizontal and vertical accuracies vary but generally, all SDB outputs are constrained by sensor type, water quality and other environmental conditions. Efforts to improve accuracy include physics-based methods (similar to radiative transfer models e.g. for atmospheric/vegetation studies) or detailed in-situ sampling of the seabed and water column, but the spatial component of SDB measurements is often under-utilised in SDB workflows despite promising results suggesting potential to improve accuracy significantly. In this study, a selection of satellite datasets (Landsat 8, RapidEye and Pleiades) at different spatial and spectral resolutions were tested using a log ratio transform to derive bathymetry in an Atlantic coastal embayment. A series of non-spatial and spatial linear analyses were then conducted and their influence on SDB prediction accuracy was assessed in addition to the significance of each model's parameters. Landsat 8 (30 m pixel size) performed relatively weak with the non-spatial model, but showed the best results with the spatial model. However, the highest spatial resolution imagery used – Pleiades (2 m pixel size) showed good results across both non-spatial and spatial models which suggests a suitability for SDB prediction at a higher spatial resolution than the others. In all cases, the spatial models were able to constrain the prediction differences at increased water depths.

## Highlights

• Satellite Derived Bathymetry key to mapping shallow waters efficiently
• Accuracy limited by sensor/environment but spatial component under-utilised.
• Multiple platforms assessed for comparison of resolution types.
• Turbidity and seabed type assessed as predictors of bathymetry
• Spatial models constrain prediction errors as water depth increases

## 1 Introduction

The rapid expansion of the Irish economy is putting unprecedented pressure on the coastal marine area and its resources (Connolly and Cummins, 2001). The Census 2016 (CSO, 2017) summary results showed that in Ireland 40% of the total population reside within 5 km of the coast. These circumstances demand efficient coastal management procedures able to protect the sustainable use of these environments. Timely and accurate environmental information such as bathymetry is necessary to support effective resource policy and management for coastal areas and assure human security and welfare. Several techniques have been developed to derive depth values used to produce bathymetric maps. Globally, single and multibeam echo sounders provide the most accurate and reliable method to derive depth (e.g. Horta et al., 2014). However, this technique is costly, slow, weather-dependent and large survey vessels are unsuited for operations in shallow waters. Airborne bathymetric LiDAR represents an alternative to vessel campaigns and its suitability has been demonstrated in coastal areas (Chust et al., 2010). This method is rapid, unhindered by maritime restrictions but performs poorly in turbid waters, as demonstrated by tests performed by the national marine mapping programme, INFOMAR (Coveney and Monteys, 2011).

Satellite-derived bathymetry (SDB) is emerging as a cost-effective alternative methodology that provides high resolution mapping over a wide area, rapidly and efficiently. Multispectral satellites of several spectral and spatial resolutions have been assessed for this purpose worldwide (e.g. Lyons et al., 2011; Poursanidis et al., 2019). Deriving bathymetry from multispectral satellite imagery applies the principle that light penetration of the water column at different wavelengths is a function of the properties of sea-water and was first proposed as a potential optical alternative for bathymetric surveys in the 70s (Lyzenga, 1978). However, it is noted that depth penetration is limited by water turbidity and these methods require calibration, particularly in areas of variable seabed type (Bramante et al., 2013; Vahtmäe and Kutser, 2016). Coastal environments are highly dynamic and heterogeneous, and therefore more studies are needed to ensure robust methodologies. Development of applications utilising satellite imagery will help EU member states like Ireland to leverage their investment in this technology.

The frontier of SDB research has advanced from basic linear functions into band ratios of log transformed models (Lyzenga, 1981), non-linear inverse models (Stumpf et al., 2003) and physics-based methods similar to radiative transfer models (Dekker et al., 2011). Empirical SDB prediction methods have been assessed for deriving bathymetry in Irish waters in previous tests (Coveney and Monteys, 2011; Monteys et al., 2015; Casal et al., 2019) and although SDB performance varies depending on the approach, the prediction differences were approximately 10% of water depth, and were influenced by water type and by sensor types.

In this study, we apply and extend a proven empirical approach (Stumpf et al., 2003) to a selection of multi-resolution imagery products: Landsat 8, RapidEye and Pleiades. The potential of these sensors has been individually reported in other studies (e.g. Collin et al., 2018; Hedley et al., 2018; Pacheco et al., 2015; Traganos and Reinartz, 2018; Sagawa et al., 2019). However, in this study, we incorporate multiple spatial, spectral and radiometric resolutions to ascertain their influence on bathymetric accuracy both prior and post image-corrections. In particular, satellite-derived relative depth (SDRD) was determined using the three satellite-based products where different spatial filters, pre-processing steps, atmospheric corrections and multispectral band combinations were investigated. This operation resulted in 23 different formulations of SDRD, each of which was assessed for use as a potential predictor variable in the study's SDB predictions models. The ground-reference water depth was provided via airborne bathymetric LiDAR.

Bathymetry has an inherent, under-utilised spatial element that can be exploited to improve SDB accuracy through application of spatial prediction techniques (e.g. Chilès and Delfiner, 1999; Li et al., 2011). We complement a previous empirical SDB prediction study for Irish coastal waters (Monteys et al., 2015), through the application of (i) linear regression (LR), a non-spatial predictor and (ii) regression kriging (RK) (Hengl et al., 2003), a spatial predictor. Here prediction accuracy is the focus, as similarly assessed in Monteys et al. (2015). Prediction uncertainty accuracy assessments (e.g. prediction confidence intervals) require more sophisticated predictors, using say, Bayesian constructions (e.g. Rue et al., 2009; Biangiardo et al., 2013). Thus, for this study our key objectives can be summarized as follows:

1. Statistically determine the best satellite-derived predictors of bathymetry for each satellite product through linear correlation and regression analyses.
2. Compare LR and RK for their SDB prediction accuracy, together with the significance of each model's parameters found from (1).
3. Evaluate the importance of integrating seabed type and turbidity on prediction accuracy.
4. Suggest steps to upscale to encompass an entire coastal bay in North Atlantic waters.

The paper is structured as follows. Section 2 introduces the study area and the study data sets. Section 3 defines the image processing approach and the formation of image-based predictor variables, the statistical analyses to retain only the most informative predictor variables, and describes the two study prediction models, LR and RK. Section 4 presents the results of the statistical analyses for the predictor variables and the SDB predictive performances of LR and RK. Section 5 highlights the main findings, the implications of this study and is followed by our conclusion.

## 2 Study area and data sets

### 2.1 Study area – Tralee Bay

**Fig. 1:**
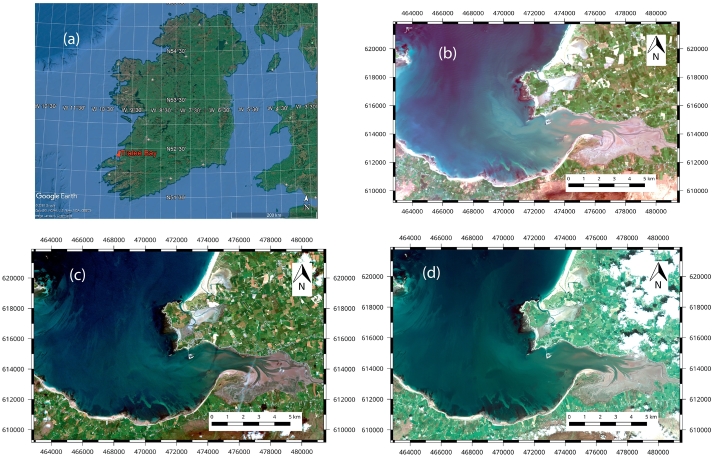
Tralee bay and satellite images selected for SDRD Study: (a) site located in south west corner of Ireland (via Google Earth) and (b) Landsat 8 - 30 m spatial resolution (c) RapidEye - 5 m spatial resolution and (d) Pleiades - 2 m spatial resolution. The presence of clouds in East of Tralee test site in the Pleiades image (2d) results in a noted difference in contrast between this image and the others.

Tralee Bay is located on the west coast of County Kerry, Ireland (Fig. 1a). Several small rivers feed into the bay through the town of Tralee and the River Lee, a large river also feeds into the bay increasing turbidity in the area surrounding the mouth. Tralee bay is representative of many of the coastal embayments on the Irish west coast where rivers enter the North Atlantic.

### 2.2 Multi resolution satellite imagery

Incorporating imagery from different satellite platforms enabled an investigation of the influence of image resolution, offering a range of spatial resolutions (pixel size), spectral resolutions (number of bands and portion of spectrum covered), temporal resolutions (seasonal variations) and radiometric resolutions (bit depth). As this research was initiated prior to Sentinel 2a becoming fully operational, Landsat 8 was the primary open data set utilised in our tests. Third Party Mission imagery was provided by the European Space Agency (ESA), which was available under license, and this enabled more tests than utilising the open data alone. The final satellite datasets selected for the project (Table 1) were Landsat 8 (30 m spatial resolution with a coastal/aerosol band), RapidEye (5 m) and Pleiades (2 m) - all multispectral satellite data sets with extensive archive coverage for Ireland.

**Table 1:** Details of satellites used in project.

Satellite	Product ID	Resolution	Bands used in project (μm)	Source
Landsat 8	LC08_L1TP_208024_20150419_20170409_01_T1	30 m	Costal Blue (0.435–0.451) Blue (0.452–0.512) Green (0.533–590) Red (0.696–0.673) Near-Infrared (0.851–0.879)	USGS Earth Explorer
RapidEye	23605785	5 m	Blue (0.440–0.510) Green (0.520–590) Red (0.630–0.685) Near-Infrared (0.760–0.850)	RapidEye's EyeFind
Pleiades	DS_PHR1B_201405291144248_FR1_PX_W010N52_0205_04866	2 m	Blue (0.43–0.55) Green (0.50–62) Red (0.59–0.71) Near-Infrared (0.74–0.94)	AIRBUS GeoStore

(USGS, 2016) (BlackBridge, 2015) (ASTRIUM, 2012)

For each Satellite data source, the choice of image was based on the following criteria:

• Extent of cloud cover over and near the study area
• Visible effects of sun glint over water
• Visible effects of turbidity within bay.
• Date of Image acquisition.
• Tidal level during image acquisition.

Cloud cover was the most significant limiting factor in the selection of satellite data. For example, in 2015 of the 69 Landsat 8 scenes captured over the survey area, only two dates were considered ‘cloud free’ and warranted further consideration. Data on tidal level was obtained from Castletownbere Tide Gauge which is part of the Irish National Tide Gauge Network and located approximately 65 km south of Tralee bay. Considering the above criteria, an optimal image from each satellite was chosen, the details of which are listed in Table 2. The Optimal images are displayed in Fig. 1(b–d).

**Table 2:** Details of satellite data used in project.

Satellite	Acquisition data	Satellite acquisition time	Time of low tide^a^	Visible turbidity	Visible sun glint
Landsat 8	19/04/2015	11:34	11:45	Moderate	Low
RapidEye	16/06/2010	12:48	12:45	Moderate to low	Moderate
Pléiades	29/05/2014	11:34	11:30	Moderate	Low

a Water Level based on Ordnance Survey Ireland Datum - Malin Head.

Satellite imagery available from free and commercial sources are generally available with varying degrees of pre-processing. The degree of processing applied to each image can range from raw, uncorrected data up to a level where all possible corrections have been applied and the secondary data generated. To ensure fair comparisons between each multispectral image source, it was important that each image was processed using the same technique. For this reason, the Landsat 8 data used in this report was processed to ‘Level 1 T’, RapidEye was processed to ‘3A’ and Pleiades to ‘ORTHO’ level – all of which are prior to application of atmospheric correction. Each data source used a differing naming convention to indicate the processing level, however, processing levels can be assumed as equivalent in terms of how raw data from each source was converted to absolute radiance with precision terrain-corrections. During radiometric correction for each data source, radiometric artefacts and relative differences between bands were detected and corrected. For each satellite source, the data was converted to absolute radiometric values using calibration coefficients developed specifically for that satellite. Each data source was geometrically corrected using accurate Digital Surface Models and ground control points. The only difference in the methodology used for geometric correction between the data sources was in the kernel type used during resampling. Unlike Landsat 8 Level 1 T data and RapidEye 3A data which use a Cubic Convolution Resampling Kernel, Pleiades ORTHO is resampled using a spline kernel (USGS, 2016) (BlackBridge, 2015) (ASTRIUM, 2012).

### 2.3 LiDAR and SONAR bathymetry data

Ground reference bathymetry data for Tralee Bay was acquired between 2008 and 2014 by the INFOMAR program. In 2008 Tenix Laser Airborne Depth Sounder (LADS) carried out a LiDAR survey of the bay (5 m spatial resolution) covering most of the bay and at 200% coverage to allow multiple passes over the same area. Data was processed using Tenix LADS proprietary hydrographic software and tidally corrected using local tide gauges. Category Zone of Confidence (CATZOC) values are used to indicate the accuracy of data presented on navigation charts. The resulting dataset can be classified to CATZOC B type and the survey report is provided as supplemental material with this paper. The seabed between Kerry Head and Brandon Point was mapped via multibeam SONAR in 2009, 2011 and 2014 by the Celtic Voyager. The RV Geo, RV Keary and Cosantóir Bradán mapped the shallower waters along the coast of Tralee Bay in 2014. Sonar data meets IHO order 1 specifications with an overall vertical error of <2% water depth and is classified as CATZOC type A1.

### 2.4 Additional data

**Fig. 2:**
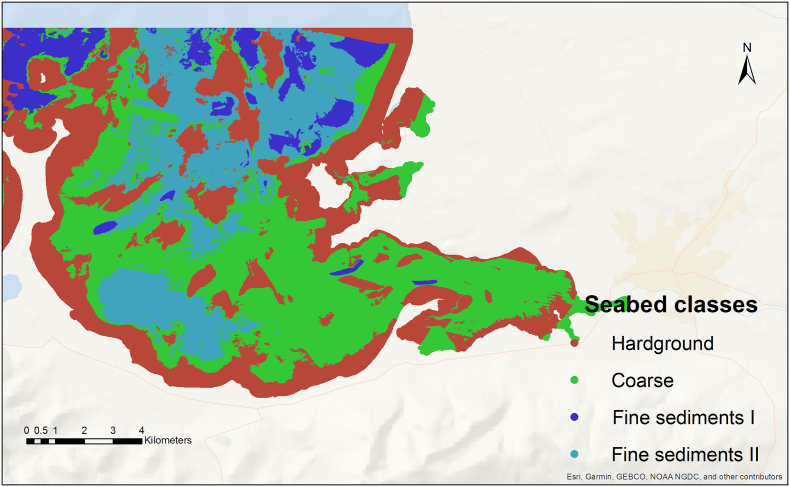
Seabed classes for Tralee bay – derived from multibeam and backscatter data and subdivided into 4 classes ranging from hardground to fine sediments.

Further data is available on seabed-type, where four classes characterizing different seabed properties in Tralee Bay may help explain the variation in water depth. Seabed information was derived from the seabed geological maps and databases published by Geological Survey Ireland. These maps, produced by interpreting multibeam bathymetry and backscatter data, inform about seabed-type and geomorphological factors. Data points for Tralee bay (Fig. 2) showing similar characteristics were grouped into four discrete classes (see Table 3). Sediment samples were used to label these classes with geological descriptors. Hardground (class 1) and Coarse (class 2) account for 35% and 35.1%, respectively of the Bay's seafloor. Fine-grained sediments (classes 3 and 4) account for over 29.9%. The difference in fine-grained sediment can primarily be attributed to two distinct backscatter acoustic signatures, which are typically related to sediment properties. “Fine sediments II” could be finer grained sediments than “Fine sediments I”, but due to insufficient sediment samples to confirm this trend these were left as undifferentiated.

**Table 3:** Seabed-type and descriptions.

Class	Name	Description	Topography
1	Hardground	Rock outcrops and coarse gravel	Rough
2	Coarse	Gravel and sand	Medium
3	Fine sediments I	Featureless mixed fine sediments	Smooth
4	Fine sediments II	Featureless mixed fine sediments	Smooth

In addition, it is always possible to use the coordinate data (Eastings and Northings) to help explain variation in water depth. Thus, seabed-type and the coordinates, together with satellite derived data are all employed as potential predictors of bathymetry using the study models.

## 3 Methodology

### 3.1 Data processing

Each of the following steps used to process the data were undertaken in the open source R statistical programming language version R 3.5.1(R core team, 2013).

#### 3.1.1 Atmospheric correction

In this paper we applied Dark Object Subtraction (DOS) atmospheric correction, since the absence of thick clouds casting shadows over deep water (as evident in Fig. 1b–d) eliminated the possibility of applying the method proposed by Hernandez and Armstrong (2016) – previously identified as optimal when atmospherically correcting imagery for deriving bathymetry.

DOS is an image-based atmospheric correction method which assumes that within an image there is a dark object that has near zero reflectance because of shadow or deep water. In its simplest implementation, this involves subtracting all values in the image by the minimum value in the image. However, some authors (e.g. Chavez, 1988) recommends the user manually select the minimum value upon examination of the DN histogram of the image. This method assumes a near zero reflectance and so a 1% reflectance is assumed which was calculated for each band by (Chavez, 1988; Zhang et al., 2010):

(1) $$L_{1\%}=\frac{0.01E_{\mathrm{sun\lambda}}\mathrm{cos\theta}\;}{\pi d^{2}}$$

where, E_sunλ_ = Exo-atmospheric solar irradiance; and θ = the solar zenith angle. Once calculated the surface radiance for each band was found by:

(2) $$L_{\text{coor}}=L_{\text{satλ}}-L_{\text{darkλ}}-d$$

where, L_satλ_ = the radiance recorded at the sensor; and L_darkλ_ = the radiance of the dark object.

#### 3.1.2 Sun-glint correction

Sun-glint can have an impact on the accuracy of depth derivations. Sun-glint is the reflection caused by light intersecting a surface at a favourable angle and as a result, is reflected directly to the sensor. Overwater waves are one of the main contributing factors to creating the conditions required for sun-glint (Kay et al., 2009). In each of the images used in this study, the effect of sun-glint was visibly noticeable and so a technique developed by Hedley et al. (2005) to correct its effect was employed. This technique assumes that in deep water, light with a wavelength >700 nm was almost completely absorbed by water. As a result, reflectance recorded in a region of deep water using a near-infrared band should have the same value as the minimum value within that region. Therefore, any deviation from this is an indication of the degree by which sun-glint is impacting the reflectance at any point over water. Following this assumption, each band can be corrected by determining the slope of a LR between it and the NIR band and applying the following formula (Hedley et al., 2005):

(3) $$L_{i}\left(\mathrm{VIS}\right)\prime =L_{i}\left(\mathrm{VIS}\right)-b_{1}\;\left[L\left(\mathrm{NIR}\right)-L_{\min}\left(\mathrm{NIR}\right)\right]$$

where, b_1_ = the slope of the LR line; L_i_(VIS) = the original value for the visible band; L(NIR) = the value for the NIR band; and L_min_(NIR) = the minimum value in sample area for the NIR band.

#### 3.1.3 Empirical determination of satellite derived relative depth (SDRD)

An empirical method that has demonstrated potential to estimate water depth in Irish waters (Monteys et al., 2015) is that developed by Stumpf et al. (2003):

(4) $$Z=m_{1}\frac{\ln\left(nR_{w}\left(\lambda_{i}\right)\right)\;}{\ln\left(nR_{w}\left(\lambda_{j}\right)\right)}-m_{0}$$

where: Z = the estimated depth; m_1_ = a tuneable constant to scale the ratio; m_0_ = the offset for a depth where Z is equal to zero; R_w_(λ_i_) = the reflectance of blue band; R_w_(λ_j_) = the reflectance at green band; and n = a fixed constant to ensure that the logarithm is positive and that a positive linear response with depth is produced.

This empirical method applies a LR to relate known depth measurements to the SDRD values (Stumpf et al., 2003). Using this method, SDRD maps were generated by calculating the log ratio of the blue and green bands of the recorded image. Here multiple derivations of SDRD were found using the Landsat 8, RapidEye or Pleiades imagery, where in turn, different spatial filters, different pre-processing steps, different atmospheric corrections and different multispectral band combinations were used (i.e., that presented above). This resulted in a total of 23 different formulations of SDRD for use as potential predictors of bathymetry in each of the study prediction models.

#### 3.1.4 Accounting for turbidity

Water turbidity can have a significant impact on water leaving radiance and thus the derived depth (e.g. Casal et al., 2019). Water turbidity can result in higher water-leaving radiances across the visible and Near Infrared (NIR) portions of the spectrum, overestimating depths in shallower areas (Bramante et al., 2013) and underestimating depths in deep areas (6 m–10 m) (Casal et al., 2019).

To remotely quantify turbidity levels, the Normalized Difference Turbidity Index (NDTI) developed by Lacaux et al. (2007), was calculated for potential use as a predictor of bathymetry and was divided into 5 equal interval classes to provide an indicator of water quality. In clear water, green light with a wavelength between 0.5 μm and 0.6 μm will have a much greater spectral response than red light with a wavelength of roughly between 0.6 μm and 0.7 μm. An increase in turbidity results in a change in the ratio of the response between green and red light and in cases of extreme turbidity, red light may even have a greater response than green light (Lacaux et al., 2007). The NDTI can be defined as:

(5) $$\text{NDTI}=\left(\text{ρred}-\text{ρgreen}\right)/\left(\text{ρred}+\text{ρgreen}\right)$$

#### 3.1.5 Data integration with INFOMAR bathymetry

The geo-referenced SDRD and NDTI values were then combined with the INFOMAR bathymetric LiDAR data. To further ensure no anomalies were introduced into the analysis by including data over land or in areas prone to high degrees of turbidity such as the river mouth, all data above the high-water mark as defined by Ordnance Survey Ireland vector shapefiles and all ground reference data with elevation values above ground level were removed.

#### 3.1.6 Summary and further data processing

The full study data set thus consisted of a single response variable (LiDAR-B) together with six distinct predictor variables, as detailed in Table 4. The spatial resolution of the full study data was 5 m (reflecting the point density of the LiDAR-B data) with *n* = 4,464,329 observations. Fitting prediction models to such a massive spatial dataset presents a problem computationally (e.g. Cressie and Johannesson, 2008), and for this reason, the study prediction models (LR and RK) were specifically chosen to reflect this (see 3.2, 3.3). To better approximate a smaller ground-truth dataset and demonstrate the utility of SDB for application anywhere, use of the full data set was unnecessary, and as such, the full data set was sub-sampled via random sampling to a smaller, more manageable size.

**Table 4:** Summary of response and predictor variables.

Variable	Variable description
Response variable	
LiDAR-B	INFOMAR bathymetry based on LiDAR survey

Predictor variables (SDRD with Landsat 8)	
LS8-CB	Coastal aerosol band only
LS8-B	Blue band only
LS8-G	Green band only
LS8-L-CBG	Log ratio using coastal aerosol/green bands
LS8-L-BG	Log ratio using blue/green bands
LS8-DOS-L-CBG	Log ratio using coastal aerosol/green bands (Atmospherically Corrected)
LS8-DOS-L-BG	Log ratio using blue/green bands (Atmospherically Corrected)

Predictor variables (SDRD with RapidEye)	
RE-B	Blue band only
RE-G	Green band only
RE-L-BG	Log ratio using blue/green bands
RE-3X3-B	3 × 3 low-pass filter applied to blue band only
RE-3X3-G	3 × 3 low-pass filter applied to green band only
RE-3X3-L-BG	3 × 3 low-pass filter applied and log ratio using blue/green bands
RE-DOS-L-BG	Log ratio using blue/green bands (Atmospherically Corrected)
RE-DOS-3X3-L-BG	3 × 3 filter & log ratio using blue/green bands (Atmospherically Corrected)

Predictor variables (SDRD with Pleiades)	
PL-B	Blue band only
PL-G	Green band only
PL-L-BG	Log ratio using blue/green bands
PL-3X3-B	3 × 3 low-pass filter applied to blue band only
PL-3X3-G	3 × 3 low-pass filter applied to green band only
PL-3X3-L-BG	3 × 3 low-pass filter applied and log ratio using blue/green bands
PL-DOS-L-BG	Log ratio using blue/green bands (Atmospherically Corrected)
PL-DOS-3X3-L-BG	3 × 3 filter applied and log ratio using blue/green bands (Atmospherically Corrected)

Predictor variables (Turbidity)	
LS8-NDTI	NDTI from Landsat 8 imagery
RE-NDTI	NDTI from RapidEye imagery
PL-NDTI	NDTI from Pleiades imagery

Predictor variable (Seabed-type)	
	Four classes of seabed-type (Table 3)

Predictor variables (Coordinates)	
Easting	Easting coordinate
Northing	Northing coordinate

**Fig. 3:**
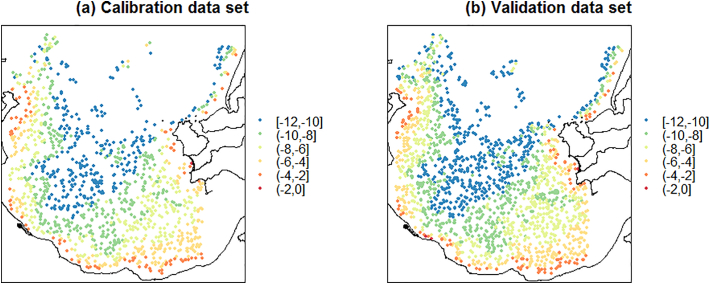
LiDAR-B maps for the (a) calibration and (b) validation data sets – depths in metres (m).

Furthermore, as it was necessary to objectively evaluate the prediction models, the decimated data set was split into a calibration (training) and validation data set, with a 40:60 split, where the specified split was judged to provide reasonably well-informed model calibrations but not at the expense of too few validation sites. This resulted in a decimated data set size of *n* = 4462 (0.10% of full data set), a calibration data set of size of *n* = 1768 (0.04% of full data set) and a validation data set size of *n* = 2678 (0.06% of full data set). In addition, it was considered inappropriate to attempt to predict LiDAR-B at depths below 12 m because the imagery data will not accurately represent such depths in all the satellite scenes evaluated for a valid cross-comparison exercise (Monteys et al., 2015). In this respect, the calibration and validation data sets were further processed to remove observations with LiDAR-B values deeper than 12 m. This resulted in a revised decimation data set size of *n* = 3041 (0.067% of full data set), a revised calibration data set of size of *n* = 1214 (0.027% of full data set) and a revised validation data set size of *n* = 1827 (0.04% of full data set). These final data sets are mapped in Fig. 3. It is stressed that this study's reported results were representative of numerous explorations with different data decimations and different randomly-sampled calibration and validation data sets, where the decimated data sets were allowed to vary in size from 0.05% (*n* = 2232) to 1% (*n* = 44,643) of the full data.

### 3.2 Statistical analyses

For the statistical analyses, objectives were to determine the strongest relationships between LiDAR-B and: (i) each of the SDRD variables derived from the Landsat 8, RapidEye or Pleiades products; (ii) the NDTI from the satellite products; (iii) seabed-type; and (iv) the coordinates. This was achieved through basic assessments of: (a) normality, to gauge where a Box-Cox transform (Box and Cox, 1964) is appropriate; (b) linear correlations (*r*) and associated scatterplots; (c) conditional boxplots for categorical variables, LS8-NDTI, RE-NDTI, PL-NDTI and Seabed-type; and (d) ‘in-sample’ non-spatial and spatial LR fits. For assessment (d), ‘in-sample’ LR fits are opposed to ‘out of sample’ LR fits where they are calibrated with the calibration data to fit ‘out-of-sample’ at the validation data sites.

Parameters of the non-spatial LR were estimated through ordinary least squares (OLS), whilst the parameters of the spatial LR were estimated using restricted maximum likelihood (REML) to account for a spatially-autocorrelated error term (as modelled via a given variogram). The OLS and REML LR fits were conducted using the linear mixed model function in the R nlme package (Pinheiro et al., 2018), where model fit statistics of *R*^2^ (fit with the highest *R*^2^, the best) and AIC (fit with the lowest AIC, the best) are reported for comparison. The results of these statistical analyses were used to determine the final predictor variable sub-sets for retention in the two ‘out-of-sample’ prediction models (LR and RK), where comparisons of prediction accuracy with respect to the predictor variables from different imagery products were undertaken. Observe that REML LR fits are computationally intensive, but with small calibration data sets (*n* < 5000, say) they can provide directions and insights for predictor variable retention for much larger calibration data sets, leading up to the full data set.

### 3.3 Methods for spatial prediction

The study prediction models consist of LR and RK only, both of which were calibrated to predict LiDAR-B informed by some combination of the SDRD data, the NDTI data, seabed-type and the coordinates. For computational reasons, RK has been chosen over its close counterpart of kriging with an external drift (KED), where RK and KED have the following properties.

Both RK and KED are LR-based geostatistical predictors, designed to account for spatial autocorrelation effects in the error term via the residuals of a LR trend fit, where RK in this study, is viewed as a statistically sub-optimal but computationally simpler version of KED. RK is an *explicit* two-stage procedure, where the LR predictions are found first, then added to the ordinary kriging (OK) predictions of the LR residuals. For this study, RK is statistically sub-optimal since: (1) its LR trend component is estimated via OLS, (2) its residual variogram parameters are estimated via a weighted least squares (WLS) model fit to the empirical variogram (Zhang et al., 1995), and (3) a local (non-unique) kriging neighbourhood of the nearest 20% of the residual data is specified (see Rivoirard, 1987).

Conversely, KED can be viewed as statistically optimal, where the LR trend and residual variogram parameters are estimated concurrently (i.e. an *implicit* single-stage procedure) using REML and provided a global (unique) kriging neighbourhood is specified. In this form, KED presents a problem computationally but is required for best linear unbiased prediction (BLUP), whereas the chosen specifications of RK (i.e. the OLS trend fit, the WLS variogram fit and the local kriging neighbourhood) are each chosen to alleviate computational burden, whilst still providing tolerable levels of prediction accuracy (i.e. as would be expected from a corresponding KED fit). Theoretical details, equivalents and comparisons for explicit RK models and implicit KED models can be found in Bailey and Gatrell (1995); Hengl et al. (2003). For this study's RK models, an isotropic exponential variogram model was specified; and the various components of an RK calibration were achieved using gstat (Pebesma, 2004) and geoR (Ribeiro and Diggle, 2001) R packages.

**Fig. 4:**
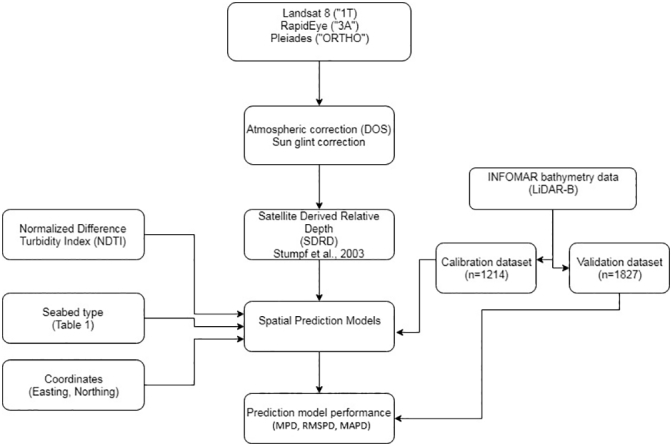
Simplified workflow processing steps for the generation and validation of the spatial prediction models used in this study.

The following set of diagnostics was used to assess LR and RK prediction accuracy. For observed *z*(x_*v*_) and predicted $\hat{z}\left(x_{v}\right)$ LiDAR data at a validation location x_*v*_, overall prediction accuracy was measured by:

(6) $$\mathrm{MPD}=\left(1/M\right)\sum_{v=1}^{M}\left\{z\left(x_{v}\right)-\hat{z}\left(x_{v}\right)\right\}$$

(7) $$\text{RMSPD}=\sqrt{\left(1/M\right)\sum_{v=1}^{M}{\left\{z\left(x_{v}\right)-\hat{z}\left(x_{v}\right)\right\}}^{2}}$$

(8) $$\text{MAPD}=\left(1/M\right)\sum_{v=1}^{M}\left|z\left(x_{v}\right)-\hat{z}\left(x_{v}\right)\right|$$

where (6), (7), (8) are the mean prediction difference (MPD), the root mean squared prediction difference (RMSPD) and the mean absolute prediction difference (MAPD), respectively; and where *M* is the size of the validation data set. All such diagnostics should tend to zero for accurate and unbiased prediction. The correlation coefficient between *z*(**x**_***v***_) and $\hat{z}\left(x_{v}\right)$ is also found where a positive value tending to one is sought, but noting that this diagnostic is scale-invariant, so consistent over- or under-prediction would not be captured. Minimum and maximum absolute prediction differences (APDs) are also given, and all diagnostics are supplemented with plots and maps to provide detailed context. Observe that the prediction difference is defined as the difference between the *observed* LiDAR-B data and the *predicted* LiDAR-B data by either LR or RK. A simplified workflow for the implementation of the study's spatial prediction models is given in Fig. 4.

## 4 Results

### 4.1 Statistical analyses

**Fig. 5:**
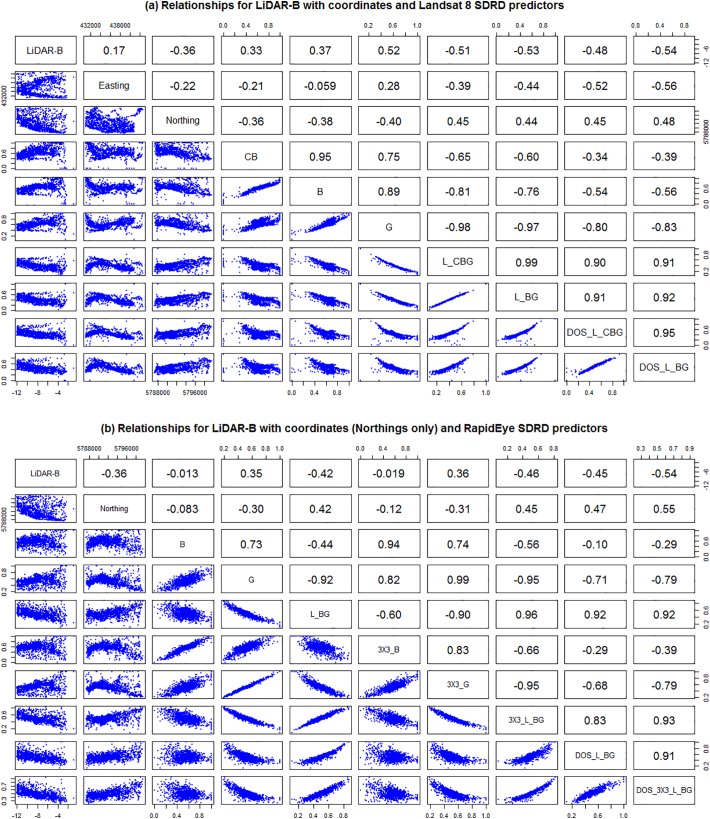
Relationships for LiDAR-B with: (a) coordinates and Landsat 8 SDRD predictors, and (b) Northings and RapidEye SDRD predictors.

**Fig. 6:**
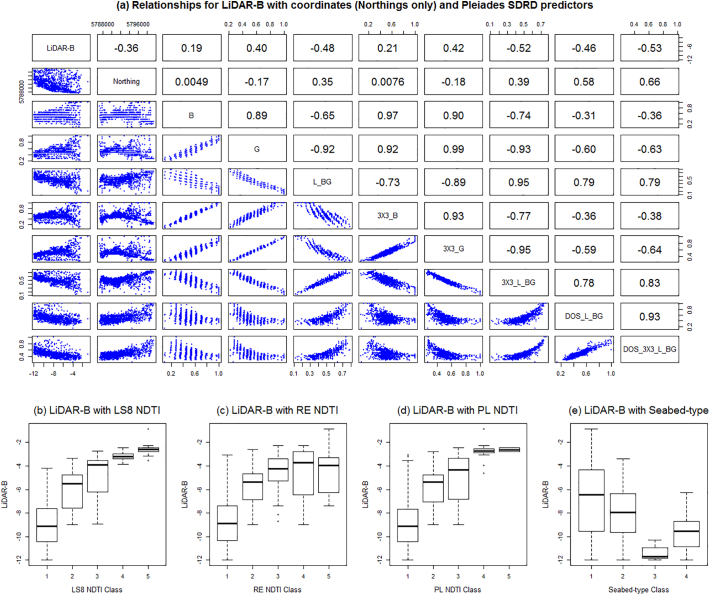
Relationships for LiDAR-B with: (a) Northings and Pleiades SDRD predictors, and (b-e) LS8 NDTI, RE NDTI, PL NDTI and Seabed-type predictors, via conditional boxplots.

We first report the results of the statistical analyses using the study calibration data set only. All variables displayed reasonable normality, so in the interest of model parsimony, no variables were transformed to such. For data relationships, the linear correlation coefficients and associated scatterplots are given in Fig. 5, Fig. 6(a), while conditional boxplots are given in Fig. 6(b–e).

The exploratory analysis also allowed us to assess the impact of the atmospheric correction on the relationship of LiDAR-B to SDRD - and in each case the atmospheric correction provided no worthwhile improvement in the r values (−0.53 moving to −0.54 for Landsat 8 after DOS, −0.42 moving to −0.45 for RapidEye and −0.48 moving to −0.46 for Pleiades). Additionally, the decrease in reflectance from single bands provided the weakest relationship. It was found that LS8_DOS_L_BG was the most strongly correlated Landsat 8 SDRD variable with LiDAR-B (*r* = −0.54). Similarly, RE_DOS_3X3_L_BG and PL_DOS_3X3_L_BG provided the strongest correlations for RapidEye and Pleiades, respectively (*r* = −0.54 and *r* = −0.53). As there was a high degree of collinearity among the SDRD predictors from each satellite product group, only the above variables were retained and used in any one LR/RK fit. In addition, the Northing coordinate was negatively and moderately correlated to LiDAR-B (*r* = −0.36), and was also retained, reflecting the north-south orientation of Tralee Bay. From the conditional boxplots, LS8-NDTI, RE-NDTI, PL-NDTI (the satellite-derived turbidity measures) and Seabed-type, could all strongly discriminate across the range of LiDAR-B values; and were thus, all worthy of retention.

**Fig. 7:**
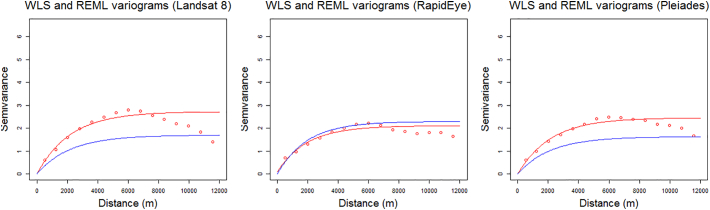
Variogram models estimated by WLS (for RK) and by REML (for spatial LR) for each satellite product. Empirical residual variograms = red circles; WLS variogram model fits to empirical residual variograms = red lines; REML model fits = blue lines (note these fits are entirely independent of the empirical residual variograms). (For interpretation of the references to colour in this figure legend, the reader is referred to the web version of this article.)

The results of the ‘in-sample’ OLS and REML LR fits are given in Table 5. The residual variograms for the three REML LR fits are given in Fig. 7, which all displayed clear spatial dependence. These are also given with the WLS estimated residual variograms used in RK, where some degree of similarity between the WLS and REML variogram fits is expected. Observe that the parameters from the REML variogram could have been used in the RK fit instead of those from the WLS variogram, but the objective here is ultimately to provide computationally feasible solutions for large data sets. There was no evidence to suggest a non-spatial LR would suffice over a spatial LR for inference, or over RK for prediction (i.e. as would be the case if the residual variograms were flat, with each indicating random variation).

**Table 5:** Preliminary (‘in-sample’) non-spatial LR (OLS) and spatial LR (REML) fits, with ***, ** and * indicating significance at *p* = 0.001, 0.01 and 0.05 levels, respectively.

Estimator	Landsat 8 LR: Estimate and significance		RapidEye LR: Estimate and significance		Pleiades LR: Estimate and significance	
	OLS	REML	OLS	REML	OLS	REML
Coefficients						
Intercept	1160***	2531	320.8***	3490	273.4*	3049
LS8_DOS_L_BG	−4.885***	0.3455	–	–	–	–
RE_DOS_3X3_L_BG	–	–	−13.149***	−1.630***	–	–
PL_DOS_3X3_L_BG	–	–	–	–	−1.115***	−1.731***
LS8-NDT1 2	1.702***	0.579***	–	–	–	–
LS8-NDT1 3	3.146***	1.236***	–	–	–	–
LS8-NDT1 4	3.643***	1.563***	–	–	–	–
LS8-NDT1 5	4.194***	1.950***	–	–	–	–
RE_NDTI 2	–	–	2.177***	0.413***	–	–
RE_NDTI 3	–	–	3.325***	0.832***	–	–
RE_NDTI 4	–	–	4.003***	1.057***	–	–
RE_NDTI 5	–	–	5.185***	2.440***	–	–
PL_NDTI 2	–	–	–	–	1.737***	0.404***
PL_NDTI 3	–	–	–	–	3.736***	1.243***
PL_NDTI 4	–	–	–	–	4.137***	2.215***
PL_NDTI 5	–	–	–	–	5.503***	3.892***
Northing	−0.000***	−0.000	−0.000***	−0.001	−0.000*	−0.000
Seabed-type 2	−0.979***	−0.573***	−0.978***	−0.629***	−1.175***	−0.582***
Seabed-type 3	−2.379***	−1.023***	−3.085***	−1.059***	−2.651***	−0.947***
Seabed-type 4	−1.931***	−0.768***	−1.890***	−0.802***	−1.908***	−0.760***

Fit statistics						
*R*^2^ (OLS only)	0.63	–	0.70	–	0.66	–
AIC	4467.0	1786.1	4213.5	1824.0	4352.9	1732.8

From Table 5, the RapidEye product provided the best SDRD and NDTI predictors in terms of the best fitting OLS LR model (*R*^2^ = 0.70), but conversely yielded an increase in AIC of 1824.0–1732.8 = 91.2 units over the Pleiades REML model, which provided the most parsimonious LR fit (i.e. with the lowest AIC at 1732.8). Landsat 8 provided the weakest OLS fit (*R*^2^ = 0.63), but not the weakest AIC results (as its REML fit reduced AIC over RapidEye). Lending weight to pursuing a spatial analysis, all REML LR fits provided large reductions in AIC over their OLS LR counterparts.

Interestingly, all predictor variables tended to be highly significant in the OLS LR fits, while the intercept and Northings tended to insignificance in the corresponding REML LR fits. This reflected improved specification in the REML case, where the Northings acted as a useful surrogate spatial effect in the OLS case, but where its predictive value reduced in the REML case. Furthermore, for the Landsat 8 case only, its SDRD predictor moved from being significant in the non-spatial case, to insignificant in the spatial case. Given these analyses, it was decided that the LR and RK prediction models would take the following three functional forms:

(9) $$\text{LiDAR}-B=f\;\left(\text{SDRD}\;\left(\mathrm{LS}8\_\mathrm{DOS}\_L\_\mathrm{BG}\right),\text{Northings},\mathrm{LS}8-\text{NDTI},\text{Seabed}-\text{type}\right)$$

(10) $$\text{LiDAR}-B=f\;\left(\text{SDRD}\;\left(\mathrm{RE}\_\mathrm{DOS}\_\text{3X}3\_L\_\mathrm{BG}\right),\text{Northings},\mathrm{RE}-\text{NDTI},\text{Seabed}-\text{type}\right)$$

(11) $$\text{LiDAR}-B=f\;\left(\text{SDRD}\;\left(\mathrm{PL}\_\mathrm{DOS}\_\text{3X}3\_L\_\mathrm{BG}\right),\text{Northings},\mathrm{PL}-\text{NDTI},\text{Seabed}-\text{type}\right)$$

Observe that Northings and LS8_DOS_L_BG (for the Landsat 8 models) were retained as they were still considered informative to ‘out-of-sample’ prediction, and it would still be considered a significant predictor in RK as RK's trend component is the ‘in-sample’ OLS LR fit.

### 4.2 Prediction model performance

**Fig. 8:**
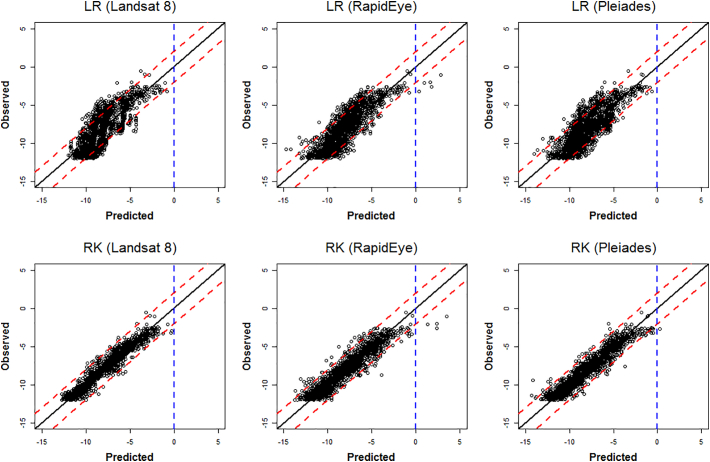
Observed versus predicted LiDAR-B for LR and RK, and all three satellite products (Landsat 8, RapidEye and Pleiades). Shown with ideal performance 45° line (black line), associated 2 m prediction difference lines (dashed red lines) and a 0 m threshold line (dashed blue line). (For interpretation of the references to colour in this figure legend, the reader is referred to the web version of this article.)

**Fig. 9:**
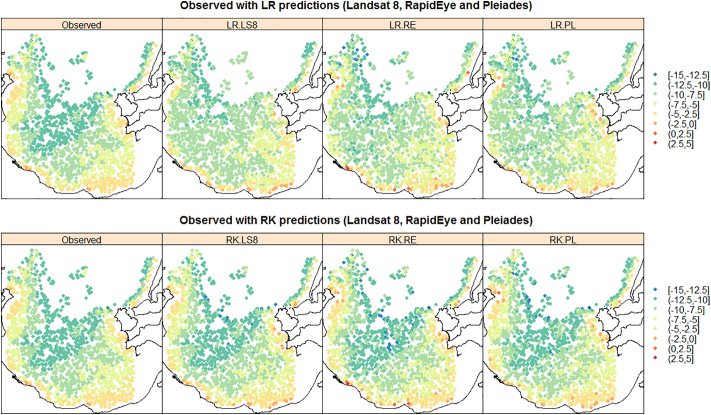
Observed LiDAR-B maps and LiDAR-B prediction maps for LR and RK, and all three satellite products. Key: Landsat 8 (LS8), RapidEye (RE) and Pleiades (PL) (depths in metres).

**Fig. 10:**
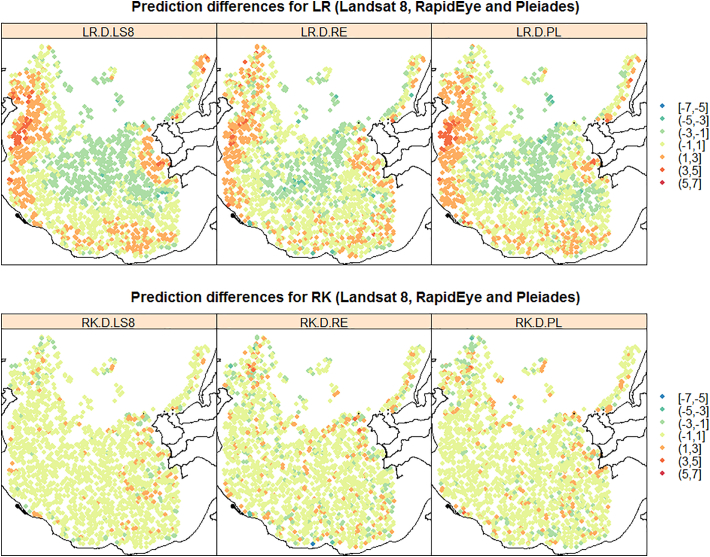
Prediction difference maps for LR and RK, and all three satellite products. Key: Landsat 8 (LS8), RapidEye (RE) and Pleiades (PL) (differences in metres).

**Fig. 11:**
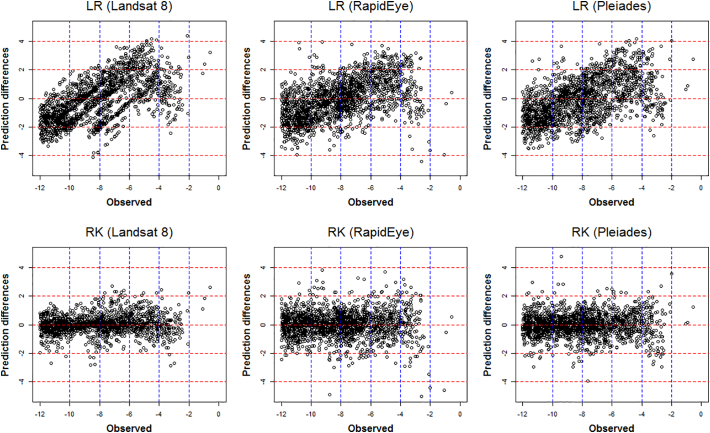
Prediction differences for LR/RK versus observed LiDAR-B for all three satellite products (Landsat 8, RapidEye and Pleiades). Plots are given with (dashed blue) lines at regular 2 m depth intervals of the observed LiDAR-B data to aid interpretation. (For interpretation of the references to colour in this figure legend, the reader is referred to the web version of this article.)

The prediction accuracy performance of the two prediction models and the three satellite products are summarized via the single-figure diagnostics in Table 6, together with plots and maps in Fig. 8, Fig. 9, Fig. 10, Fig. 11. On viewing the diagnostics in Table 6, some clear trends emerge, where the most accurate model was RK with Landsat 8 products. For all three satellite products, RK always out-performed LR, both in terms of average bias (MPD) and average prediction accuracy (MAPD, RMSPD). Interestingly, with LR only, prediction using RapidEye followed by Pleiades, both outperformed prediction with the Landsat 8 products. However, when residual spatial information was considered with RK, this behaviour was reversed, where prediction was most accurate using Landsat 8 products, then Pleiades, then RapidEye. This behaviour appears unusual, but can in part, be explained by high prediction differences with RK informed by the Pleiades and RapidEye products (see Maximum APDs) - higher than that found with the corresponding LR model. However, for prediction using the Landsat 8 products, RK significantly reduced high prediction differences over its LR counterpart (see Maximum APDs). Tentatively, this suggests that prediction using the relatively high-resolution Pleiades and RapidEye products is more prone to spatial anomalies, than that found with the comparatively low-resolution Landsat 8 products.

**Table 6:** Prediction accuracy diagnostics for LR and RK, and all three satellite products. Key: Landsat 8 (LS8), RapidEye (RE) and Pleiades (PL).

Diagnostic	Minimum APD			Maximum APD			MPD		
Model/product	LS8	RE	PL	LS8	RE	PL	LS8	RE	PL
LR	0	0	0.001	4.368	4.430	4.170	−0.049	−0.043	−0.081
RK	0	0	0	2.879	5.024	4.756	0.027	0.017	0.003


Diagnostic	MAPD			RMSPD			Correlation		
Model/product	LS8	RE	PL	LS8	RE	PL	LS8	RE	PL
LR	1.252	1.088	1.182	1.514	1.346	1.445	0.787	0.836	0.809
RK	0.436	0.668	0.595	0.637	0.905	0.797	0.967	0.938	0.950

These effects are more clearly seen in the observed versus predicted scatterplots of Fig. 8, where obvious outlying points were evident for RK using the RapidEye and Pleiades products. Prediction with the RapidEye products also resulted in impossible (positive) LiDAR-B predictions with both LR and RK. RK clearly performed better than LR as points are more clustered around the 45° line. These results were contrary to the *in-sample* results of Section 4.1 with the OLS and REML LR fits but should not be viewed as unusual given the assessment here was *out-of-sample* (i.e. with the validation data) and the results were not always linear in behaviour. Further, differences in the *in-sample* LR results were often marginal. Our study therefore demonstrates that clearly, prediction model choice was always of more importance than satellite product choice. In terms of spatial performance, the observed data and the predictions are mapped in Fig. 9, and the corresponding prediction differences are mapped in Fig. 10 for all six model/satellite product combinations. From Fig. 9, all three RK models appear to reflect the spatial characteristics of the observed LiDAR-B data reasonably well, but some impossible predictions occur in the shallows. The prediction difference maps (Fig. 10) clearly depicts where the RK models out-perform the LR models, especially in the western shallow areas. Finally, Fig. 11 plots the observed LiDAR-B data versus the prediction differences, where all three LR fits tend to over-predict in shallow waters but tend to under-predict in deep water. This characteristic disappears with all three RK fits.

## 5 Discussion

The extraction of bathymetric information from optical remote sensing data can be generally divided into two main approaches: empirical approaches and “physics-based” model-inversion approaches. Among the empirical approaches, one of the most commonly used is the band ratio regression model (Stumpf et al., 2003). However, in recent years, new studies have focused on enhancing empirical model performance, for example, through spatial rather than standard, non-spatial modelling (e.g. Su et al., 2015; Monteys et al., 2015; Chybicki, 2018). Following this trend, this study assessed an empirical modelling framework through the incorporation of spatial autocorrelation effects. This was specifically achieved via a REML estimated LR model for inference (i.e. significance of predictor variables) and also by an RK model for prediction (that was designed to save on computational costs). Both models were applied to a selection of satellite products (Landsat 8, RapidEye and Pleiades) each with different spatial and spectral resolutions in order to better constrain SDB prediction accuracy. The temporal offset between the images and the ground reference LiDAR ranged from a few months to a number of years and therefore this represents a potential error source and does not allow for a definitive comparison. Delays in finding cloud free images with minimal evidence of turbidity also influenced the temporal offset. However, the bathymetric LiDAR dataset was selected for this study as it provided complete coverage of the whole bay with overlaps for verification. It was considered sufficiently accurate for SDB comparison as similar temporal offsets between satellite imagery and reference LiDAR have been incorporated successfully in SDB studies before (Vinayaraj et al., 2016) and when compared with subsequent localised SONAR surveys in later years for Tralee bay, it displayed no significant variation. The LiDAR dataset also enables testing of consistency across images, particularly regarding water depth intervals.

In terms of SDB prediction performance, LR models using the RapidEye and Pleiades products showed smaller and more consistent prediction differences than that found with Landsat 8; however, Landsat 8 models seemed to work better than RapidEye and Pleiades models locally in the deeper parts of the bay (water depths >10 m) (Fig. 9). All three LR models tended to over-predict in shallow waters but tended to under-predict in deeper waters, but importantly, this was not the case for RK, where this prediction bias was not present (Fig. 10). Conversely, for the RK models, Landsat 8 marginally outperforms RapidEye and Pleiades based on the prediction accuracy diagnostics (Table 6) and on the prediction difference plots (Fig. 11). Performance was also assessed at different water intervals and as a general indication of the success of the methodology for the whole test site.

### 5.1 Water depth intervals

#### 5.1.1 0–4 m

In very shallow water depths (<4 m water depths) the trends observed across all three satellite images indicate a similar over-prediction pattern generally increasing with depth (see the observed versus LR predicted and observed versus LR prediction difference plots in Fig. 9, Fig. 10). The pattern observed is inverse to the 4 to 12 m interval. The most plausible explanation for this effect is the degree of influence in the observed seafloor reflectance values. Reflectance values from the visible bands can carry significant reflected light from the seafloor contribution. Seafloor variability at the pixel scale can occur primarily due to changes in seafloor type, variations in slope or aspect; or when it is covered by algae or other non-geological factors. Local seafloor variation is present in the study area as observed, for instance, in the high resolution bathymetry images of the seafloor that appear with glacially shaped terrain characteristics. The reflectance response, at the pixel scale, from the three platforms are expected to differ substantially and are difficult to quantify. The same issue was reported by other studies using only LR models (e.g. Vahtmäe and Kutser, 2016; Casal et al., 2019). Algae and rocky bottoms present a darker signal compared to deep water areas having an influence on the performance of the model.

#### 5.1.2 4–8 m

As the bay deepens the trend in model performance gradually changes from over-prediction to prediction values clustered around the 45° trend line with minimal prediction difference. The influence of the seafloor gradually diminishes and other factors linked to water properties might now play a more important role. In deeper waters (water depth > 6 m) the results show prediction differences gradually increasing towards negative values. This trend towards under-prediction possibly reflects a depth threshold where the contribution of the seafloor is negligible or absent. A similar depth limit has been reported in other studies carried out on the Irish coast using empirical methods but without a spatial component and also using Sentinel-2 data (Casal et al., 2019). This confirmatory evidence suggests that around this depth (~6 m) lies a critical limit for SDB prediction using non-spatial LR models in similar regions on the Irish coast.

#### 5.1.3 8–12 m

The central and deepest part of the study area, where water depths ranged between 8 and 12 m had generally low prediction differences for all three RK models, whereas for the LR models, relatively high prediction differences were present, representing a continued tendency to under-predict (Fig. 10). This behaviour is reinforced in the observed versus predicted scatterplots for LR (Fig. 8 - top panel), where the scatterplot trends have a slope change when compared to that at 4 to 8 m depths. This slope change is most pronounced in the Landsat 8 LR model. This change can be attributed to the non-linear relationship between reflectance versus water depth as the plateau reflects a water depth threshold caused by the combination of an absence of seafloor component and maximum light penetration.

### 5.2 General bay discussion

The distribution of the prediction differences in the North East corner of the bay displayed high spatial variability, both with large negative and large positive prediction differences. This was true both for LR and RK. This high variability in prediction accuracy can be attributed primarily to local changes in seabed type between rock outcrops and fine-grained sediments. The influence of hardgrounds (rock outcrops and mixed gravelly sediments) has already been described in other studies carried out on the Irish coast (also using spatial prediction models) as a source of high prediction difference (Monteys et al., 2015). The inclusion of seabed class in the prediction models helps to understand its influence on prediction accuracy and the local limitations in the overall bathymetry results. In general, the LR models exhibited large positive prediction differences (+2 m) around the edges of the bay, particularly in areas characterised by hardgrounds and coarse gravel (Seabed class 1). Fine-grained sediments (Seabed classes 3 and 4) presented lower prediction differences. For the spatial RK models, this non-conformity was partially addressed, however large prediction differences were still present due to local variability driven by seabed type. For further avenues of research, firstly an investigation making SDRD itself more spatially-explicit would be worthwhile. Secondly, for upscaling the study results to the whole bay (i.e. use the full data set), tools providing cloud-based computing like Google Earth Engine should be explored further, as demonstrated in Traganos et al. (2018). Computational savings could also be achieved via mathematical adjustments to the LR and RK models (e.g. Cressie and Johannesson, 2008). On the other hand, Sentinel-2 data with improved technical capabilities in comparison to Landsat-8 (such as higher spatial resolution and 13 spectral channels ranging from the Visible and the Near-Infrared (VNIR) to the Shortwave Infrared (SWIR)), becomes a potential dataset that could provide new advancements in the performance of SDB and in the generation of more detailed and accurate satellite derive bathymetry maps.

## 6 Conclusions

In this study, methods for improving accuracies of satellite derived bathymetry (SDB) were explored using three satellite datasets and two linear prediction models, one non-spatial (linear regression), the other spatial (regression kriging). For the satellite derived relative depth (SDRD) predictor variables, a total of 23 different constructions were evaluated, with different spectral band combinations, spatial filters and log ratios. Turbidity and seabed type were also assessed as predictors of bathymetry. By using LiDAR derived bathymetric maps as ground reference data, we can conclude that:

1. All three satellite products provide robust and meaningful results to assess SDB prediction accuracy at different spatial and spectral resolutions in the test area, Tralee bay.
2. SDB predictions using Landsat 8 products showed the most accurate results when using the spatial, RK model, but returned the largest prediction differences with the non-spatial, LR model.
3. Pleiades products returned good results both with the LR and the RK models, suggesting a certain suitability for SDB at high spatial resolutions.
4. In all cases, the spatial RK model was able to constrain SDB prediction differences as water depth increased, whereas the non-spatial LR performed poorly in this respect.

## Appendix A Supplementary data

Report of Bathymetric LiDAR Survey - Ireland 2008. Image 1
