# Supplementary material for: A comparison of Landsat 8, RapidEye and Pleiades products for improving empirical predictions of satellite-derived bathymetry
Source: Remote Sens Environ. 2019 Nov;233:111414. doi: 10.1016/j.rse.2019.111414 (PMC6876676; doi:10.1016/j.rse.2019.111414)
Supplement: Supplementary file 1 — Report of Bathymetric LiDAR Survey - Ireland 2008. [file mmc1.pdf]

Tenix®

Report of Survey

Provision of Hydrographic LiDAR Services NW Ireland – Galway Bay, Tralee Bay, Blacksod Bay, Lough Foyle, Sligo Bay and Donegal Harbour

INFOMAR Contract LADS_08_01

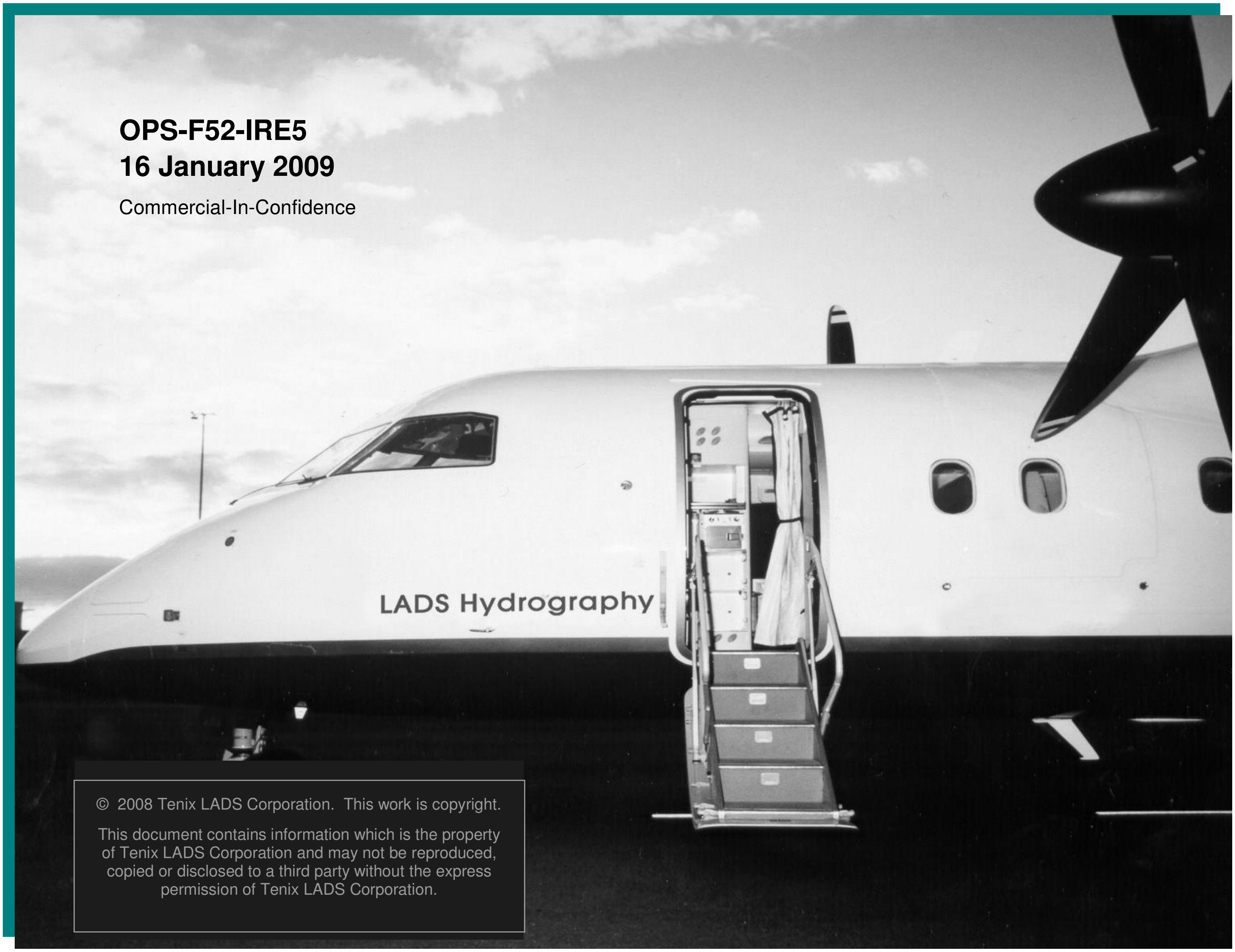

OPS-F52-IRE5
16 January 2009

Commercial-In-Confidence

© 2008 Tenix LADS Corporation. This work is copyright.

This document contains information which is the property of Tenix LADS Corporation and may not be reproduced, copied or disclosed to a third party without the express permission of Tenix LADS Corporation.

1. Introduction

This Report of Survey applies to data collected by Tenix LADS Corporation during survey operations between 13 May and 16 June 2008 off the north and west coasts of Ireland, including North Galway Bay, north coast of the Aran Islands, Tralee Bay, Blacksod Bay and Lough Foyle, using the LADS Mk II LiDAR system; and provided to the Geological Survey of Ireland (GSI) as an enclosure to this report under contract number LADS_08_01.

In addition data was also collected by a subcontractor, BLOM Aerofilms, using the Hawkeye II LiDAR system in Donegal Harbour, Sligo Bay, Greatman's and Cashla Bay. The collection of this data whilst referred to in this report, will be fully detailed in a separate report by BLOM Aerofilms.

Hawkeye data collected in Greatman's Bay and Cashla Bay has been merged with LADS data collected along the north coast of Galway Bay and is delivered and reported on under this report. Separate products for the Hawkeye II areas of Sligo and Donegal Bay will be delivered separately with the BLOM Aerofilm's report.

1.1 Purpose

The survey was conducted as part of the Integrated Mapping for Sustainable Development of Ireland's Marine Resource (INFOMAR) project, which is a follow on strategy from the Irish National Seabed Survey (INSS). Data collected and supplied under this contract will be used by the Geological Survey of Ireland (GSI) and the Irish Marine Institute (MI) to provide sustainable management of marine based activities including:

- Mineral, oil and gas exploration and exploitation
- Fishing and offshore aquaculture
- Marine safety
- Coastal zone management
- Coastal engineering and erosion
- Renewable energy development

1.2 Survey Areas

Three survey areas were initially nominated in the contract; these areas were located inside Donegal Bay, Galway Bay and Sligo Bay. One area, Sligo Bay, was conducted solely using the Hawkeye system and will be reported separately; the remaining two areas were planned to be surveyed in a joint operation involving both the LADS and Hawkeye systems. Hawkeye was tasked to survey the intertidal areas of Donegal Harbour at the head of Donegal Bay and Greatman's Bay and Cashla Bay running off the north west coast of Galway Bay, whilst the LADS system was tasked to survey the intertidal areas of north eastern Galway Bay and all coastal areas and deep water areas with the nominated areas of Donegal Bay and Galway Bay.

Whilst the Hawkeye operations in Donegal Harbour were successfully completed in April, algae blooms in May and June resulted in the remaining parts of Donegal Bay being unsuitable for LiDAR survey operations by the time the LADS system commenced survey operations in mid May. As a result LADS operations in Donegal Bay were cancelled and alternative areas were nominated by GSI.

Two alternative areas, Tralee Bay and Blacksod Bay, were nominated. During the course of the survey an additional area, Lough Foyle, was also nominated for operations and the Galway Bay area was extended to include the northern shore of the Aran Islands.

All areas were required to be flown at between 100% and 200% coverage. Coverage flown was varied to maintain the required sounding density in coastal areas and to also allow coverage in

alternative areas to be maximised at 100% coverage. Decisions made in relation to the variation in survey effort to trade off 200% survey coverage in favour of extending the 100% coverage into alternative areas was made through discussion between the Tenix Survey Project Manager and the GSI Project Manager.

Areas covered during this survey were:

- **Northern Coastline - Galway Bay**

Survey area extends along the north coast of Galway Bay from Golam Head in the west to Galway Harbour and North Bay in the east and south to meet up with the 2006 LADS survey. The area included Greatman's Bay and Cashla Bay

- **Northern Coastline - Aran Islands**

Survey area extends along the northern shore line of Inishmore Island, Inishmaan Island, and Inisheer Island.

- **Tralee Bay**

Survey area covering the main part of Tralee Bay, extending from the Seven Hogs and Rough Point in the north west, Muckaghmore Rock in the north, to Fenit Island, Barrow Harbour and Fenit Harbour in the south east and including the southern shore line.

- **Blacksod Bay**

A survey area covering the southern part of Blacksod Bay extending from the seaward side of the Mullet Peninsula to the eastern shore line, and south to enclose the southern tip of the Mullet Peninsula. Coverage was extended north into Blacksod Bay as far as could be achieved in a single flight.

- **Lough Foyle**

A land locked bay surveyed from the seaward of the entrance to Ture Point in the south west and extended south east as far as could be achieved in a single neap tide period.

- **Sligo Bay**

Survey area covers all parts of Sligo Bay, including the intertidal areas Drumcliff Bay, Sligo Harbour, and Ballysdare Bay, to the 10m contour.

- **Donegal Harbour**

Survey area covers the intertidal areas of Donegal Harbour, at the head of Donegal Bay, to the 10m contour.

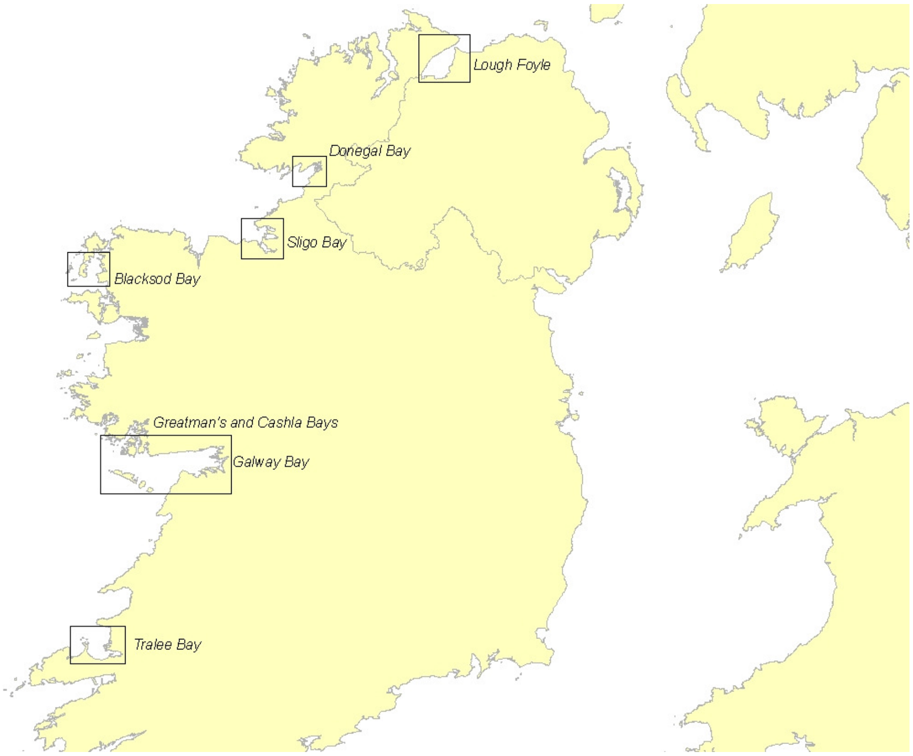

Figure 1 – Chartlet of the 2008 Survey Areas

2. LADS Survey Operations

2.1 Field Deployment

Operations in Ireland commenced with the arrival of a tide gauge insertion team on 13 May. After a short delay due to the late arrival of freight, tidal operations commenced on 16 May with the insertion of a Bottom Mounted Tide Gauge (BMTG) at the head of Donegal Bay from a local fishing boat. A wharf tide gauge was then inserted into Mullagmore Harbour during the late afternoon of 16 May and 25 hour check conducted. On 16 May a GSI surveyor inserted a benchmark on the pier at the head of Donegal Bay to enable a transfer of datum to the BMTG via a gauge / gauge comparison; however due to technical difficulties a temporary gauge on this pier could not be installed on 18 May. A LADS tide gauge was installed at Rossaveel Harbour on 20 May.

The remainder of the Survey / Logistics / Engineering team arrived between 18 May and 20 May. A GPS base station was established on the roof of the SAS Radisson Hotel, Galway, on 18 May and coordinated by LADS surveyors using static GPS methods. On 19 May a local survey company, Arrigan Geo-Surveyors, conducted a check coordination of the base station and independently established and coordinated three check marks on the tarmac at Galway airport.

The LADS Aircraft VH-LCL arrived at Galway at 1600 Tuesday 20 May. After a full set off checks on the airborne system was completed, a static positional check on all aircraft systems was conducted during the afternoon of 21 May.

Flying operations were commenced with a flight to Galway Bay on 22 May, during which data was successfully collected in all parts of the Galway Bay survey area; weather conditions in Donegal Bay at this time were not suitable due to strong north easterly winds and low cloud. A second successful flight to Galway Bay was achieved on 24 May, however a third flight on 25 May was aborted as the strong north easterly winds generated high levels of turbulence throughout both the Donegal and Galway Bay survey areas. A successful fourth flight was achieved on 27 May to Galway Bay before weather conditions closed in again.

On 28 May LADS surveyors recovered the tide gauge at Mullagmore Harbour, Donegal Bay, and installed it at Feint Harbour in Tralee Bay during the morning of 29 May. This provided an alternative to the Donegal and Galway Bay areas during the periods of poor weather. During the afternoon of 29 May the alternative Tralee Bay survey area was activated with a successful fifth flight completed and extended via a refuelling stop at Kerry airport. A sixth flight was achieved in Galway Bay on 31 May in relatively calm conditions. During this flight operations were attempted in Donegal Bay but had to be aborted due to the presence of an algae bloom caused by warming water temperatures within the bay. A seventh flight was aborted on 01 June as a large bank of sea fog moved into the Tralee and Galway Bay survey areas.

A successful eighth flight was flown to Tralee and Galway Bays on 02 June, working around a storm front that moved through the area during the afternoon. The ninth flight on 03 June was aborted due to poor water quality in Donegal Bay and strong winds and rough sea conditions in Galway and Tralee Bays. Further operations in Donegal Bay were officially cancelled after this flight, with efforts now to be concentrated in Galway Bay, Tralee Bays and extended into Blacksod Bay.

Flight approvals from the United Kingdom for operations in Northern Ireland were received at the start of June. As the next neap tide window opportunity for operations in Lough Foyle was mid June the deployment was extended.

Abating weather conditions in Galway Bay on Thursday 05 allowed a full tenth flight to be flown in the Galway and Aran Island areas to finish off the 200% coverage. An eleventh flight was conducted to Tralee Bay on Friday 06 June to finish off the southern main part of the bay.

No flying was undertaken over the weekend period 07 to 09 June whilst waiting for the onset of the neap tide period in Lough Foyle. During this period LADS surveyors installed a temporary tide gauge at the wharf in Derry and GSI surveyors installed a gauge at Greencastle on the north west coast of Lough Foyle. Weather and water conditions were then unsuitable until 12 June when a successful twelfth flight was flown to Lough Foyle during the optimum neap tide period. A successful thirteenth flight was flown to Lough Foyle on 13 June, by the end of which all possible coverage during that neap tide period was achieved.

With further data collection not considered possible in Lough Foyle until the next neap tide period, the fourteenth and final flight was flown to Blacksod Bay on 14 June to collect a large block of data in the entrance to the bay.

During the final week of operations two LADS surveyors visited all survey areas to progressively recover all deployed gauges as areas were finished, in addition they inserted a temporary gauge at Mount Charles Pier, Donegal Bay to allow a gauge / gauge comparison to be conducted to transfer datum to the Bottom Mounted Tide Gauges. These gauges were recovered on Monday 16 June.

The LADS aircraft VH-LCL departed Ireland on 16 June, as did most of the LADS survey team. The remaining tides team departed on 18 June after recovering all remaining tides equipment.

Operations were not affected by any major technical issues or delays.

A total of 60 hours 19 minutes was spent on task collecting survey data; there was no downtime due to system issues.

2.2 Weather

Weather conditions off the west coast of Ireland are highly variable and were the dominant factors affecting operations with strong winds, rain and cool temperatures followed by warm calm conditions, thunderstorms, low cloud and fog.

The survey commenced with strong north easterly winds causing heavy turbulence throughout both the Donegal and Galway survey areas; heavy rainfall was associated with this weather pattern especially in the Donegal Bay area. Winds abated towards the end of May and were replaced with calm warmer conditions, isolated thunderstorms with heavy rainfall and large banks of sea fog off the west coast. Conditions in early June were much calmer with predominately light westerly winds allowing Galway Bay and Tralee Bay to be completed. The warmer conditions combined with high rainfall meant that water conditions in Donegal Bay deteriorated as an algae bloom reduced water penetration to almost zero; as a result further operations in Donegal Bay were substituted for operations in Tralee Bay.

2.3 Deployment Site

Operations were undertaken from Galway airport. The data processing facility was located at the Radisson SAS Hotel, where personnel were also accommodated.

2.4 Support

Support for the project was received from the GSI Seabed Mapping department who inserted and supplied tide data at several temporary gauge locations and supplied details and access to permanent tide stations installed as part of the Irish National Tide Gauge network.

Galway based survey surveying company Arrigan Geo-Surveyors was subcontracted to conduct geodetic observations to establish static position check marks.

2.5 Defects

Two specialist maintainers and a comprehensive set of Airborne and Ground System spares were deployed to the field site. This approach ensured that the progress of the survey was not adversely affected by defects.

No AS failures occurred that affected the survey data quality.

3. LADS Technical Description

3.1 Digital Surveying System

Data was collected using the LADS Mk II Airborne System and processed using the LADS Mk II Ground System.

Data Collection. The LADS Mk II Airborne System (AS) is installed in a Dash 8-202 series aircraft, which has a transit speed of 250 knots at altitudes of up to 25000 feet and an endurance of up to eight hours. Survey operations are conducted from heights between 1200 and 2200 feet at a ground speed of 175 knots. The aircraft is fitted with a Nd:Yag laser on a stabilised platform, which operates at 900 hertz to provide 5x5 metre laser spot spacing in the main line sounding mode of operation across a swath width of 240 metres. The electro-mechanical scanner can also be operated to provide laser spot spacings of 6x6, 4x4, 3x3, 2.5x2.5 and 2x2 metres with reduced swath widths. The green returned laser energy is captured by the green receiver, and then digitised and logged onto Digital Linear Tape (DLT). The height of the aircraft is determined from the infrared laser, AHRS and GPS. The system can operate during both day and night. Night operations are enhanced by removing the filter on the receiving optics. Real-time positioning is provided by Fugro WADGPS or raw GPS. The AS also contains a GPS logger (roving receiver) that is used in the calculation of post-processed KGPS positions using the GrafNAV processing software.

Data Processing. The LADS Mk II Ground System (GS) consists of a Digital Alpha Series ES 40 multi-processor server computer that can be transported in the aircraft to the deployment site. PC terminals provide the user interface to the hydrographic surveyor. Data processing on the GS was commenced in the field and completed at the survey depot in Adelaide. The GS also contains a GPS logger (base receiver). This allows the subsequent calculation of post-processed Kinematic GPS positions (L1/L2 carrier phase) from the local GPS base station. The KGPS positions are calculated off-line using GrafNAV software then imported into the GS and applied to soundings.

Additional Quality Control Software. Terramodel was used for survey planning, while Fledermaus and in-house developed QC Tools software, using GMT and VTK, were used to review the data. Arc GIS and MicroStation were used in the product generation phase.

Details of the digital surveying system are provided in Annex B.

3.2 Geodetic Control

Horizontal control for the data collection was based on the World Geodetic System 1984 (WGS84). All data was post-processed relative to the European Terrestrial Reference System (ETRS89) datum during the application of the post-processed Kinematic GPS positions. All coordinates unless, otherwise stated, refer to the Universal Transverse Mercator projection, Zone 29 Central Meridian 9° West.

Fugro OmniSTAR Wide Area Differential GPS was used with a continually updated virtual reference position for real time positioning.

A local GPS base station was established by Tenix LADS Corporation surveyors at the SAS Radisson Hotel in Galway to provide post-processed Kinematic GPS (KGPS) positional solutions for each data point. Details on the coordination of this mark are provided in Annex C.

Arrigan Geo-Surveyors of Galway established three independently coordinated check marks on the Galway International Airport apron using a combination of rapid static and terrestrial methods on 19

May. These marks were used to conduct static position checks of all aircraft positioning systems. Further details of the geodetic control and static position checks are provided in Annexes C and D.

3.3 Position Fixing Systems

Real-time positions were determined using an Ashtech GG24 GPS receiver with WADGPS (Fugro OmniSTAR) corrections received via satellite. Corrections were provided using the Virtual Base Station (VBS) service and delivered to the aircraft via the communication satellite.

L1/L2 carrier phase KGPS post-processed positions relative to a local GPS base station on the roof of the Radisson SAS Hotel were calculated using GrafNAV software from two Z12 dual frequency GPS receivers. These positions were imported into the Ground System and applied to all soundings.

3.3.1 Position Checks

The positioning systems were subject to the following checks prior to, during and following data collection, as follows:

a. Static Position Check. Prior to commencing data collection the derived coordinates of the aircraft GPS antenna were determined relative to the control point on the tarmac apron at Galway International Airport. The static position check was conducted on 21 May 2008. Data was logged by each LADS Mk II positioning system during two sessions; this enabled the positions to be verified against the known surveyed point. The autonomous GPS logging session took place over a two-hour period and the results were autonomous GPS ± 6.109 metres (95% confidence) and post-processed KGPS ± 0.170 metres (95% confidence). The WADGPS logging session took place over a two-hour period and the results were WADGPS ± 2.728 metres (95% confidence) and post-processed KGPS ± 0.170 metres (95% confidence).

b. Dynamic Position Check. During each sortie, GPS data was logged on the aircraft and at the local GPS Base Station. The WADGPS real-time and the post-processed KGPS positions of the aircraft have been compared. The mean difference was 0.97 metres (95% confidence) and the maximum difference was 4.23 metres, which is consistent with historical results.

c. Position Confidence. The position quality was also monitored using a post-processed position confidence (C3), which is determined from the AS platform error, GPS error and residual errors between the actual GPS positions and aircraft position as determined from the line of best fit.

d. Navigation Checks (Navcals). Navigation checks were conducted over the temporary GPS base station located on the roof of the Radisson SAS Hotel. This enabled the known position of the antenna to be checked against the image from the digital camera, thus providing a gross error check.

Details of position checks in paragraphs a. to c. above are provided in Annex D.

3.3.2 Accuracy of Position

Based on the results of the static position check, dynamic comparisons, navigational checks and theoretical calculations it is considered that IHO Order-1 position accuracy was achieved.

Details of position accuracy are provided in Annex G.

3.4 Vertical Control

3.4.1 Survey Datum

All depths are reduced to Malin Head (MH) as defined by observed tides connected to local survey marks in Galway Bay, Blacksod Bay and Tralee Bay. All depths in Lough Foyle are reduced to Belfast Lough (BL) as defined by observed tides connected to local survey marks. A separate digital dataset of all areas reduced to Lowest Astronomical Tide (LAT) was provided for delivery to the United Kingdom Hydrographic Office (UKHO) for charting purposes.

3.4.2 Tides

Observed tides were used to reduce all soundings to sounding datum

Observed tides were supplied from a combination of temporary gauges installed by Tenix LADS Corporation surveyors; temporary gauges installed by GSI staff and permanently installed tide gauges operated as part of the Irish National Tide Gauge network. Separate tide models were established for each of the survey areas, a total of 12 tide stations were incorporated into 24 tide areas.

Full details of the tidal model and the algorithms used for this survey are provided in Annex E.

3.5 Bathymetry

Main lines of sounding were conducted using 5x5 metre laser spot spacing with a swath width of 240 metres, at an aircraft ground speed of 175 knots. Main lines of sounding were flown at 110 and 220 metre spacing which provided 100% or 200% coverage of the seabed.

Main lines of sounding were orientated along the longest side of each defined survey polygon, with at least one cross line flown at right angles to the main lines in each survey polygon.

Data quality is dependent on the signal to noise ratio of the laser return from the seabed. The quality of data collected during this survey varied from area to area. In general, very good data quality was achieved in flat or shallow sandy areas, whilst the steeper rock areas had increased levels of noise, possibly due to high levels of seaweed and kelp.

A full description of the results achieved in each study area is provided in Section 4.

3.5.1 Footprint of the Laser Beam

At the sea surface the footprint of the laser beam is approximately 2.5 metres in diameter. As the beam passes through the water column it diverges slightly due to scattering.

3.5.2 Depth Benchmarks

A benchmark line was surveyed north of the Aran Islands at the start of the survey; however the highly variable water clarity and weather conditions meant that no meaningful data was collected over the benchmarks on subsequent sorites. In addition, the wide geographical separation of each of the five survey areas meant that there was little opportunity to obtain repeat observations at the one location. As a result no meaningful benchmark comparisons were observed, and historical values for the system accuracy have been adopted and used in the calculation of the overall vertical accuracy of the depths.

The overlap between lines was consistent with the LADS Mk II system operating correctly, that the tidal model was consistent and that tides were correctly applied.

3.5.3 Cross Lines

Fourteen cross lines were flown with at least one in each survey area. These cross lines provided high quality data to assess the consistency of the tide model.

For the survey area an average mean depth difference between the cross lines and main lines was 0.03 metres with an average standard deviation of ±0.18 metres. These values were generated from the intersection of 290 runs and the comparison of 447615 individual depths. The results in the deeper offshore areas were slightly noisier than would normally be expected indicating increased levels of near seabed turbidity, large areas of kelp and a rough seabed which is the nature of the seabed along the west coast of Ireland.

These results are consistent with correct operation of the LADS system, and indicate a consistent tidal model throughout the area and that tides were applied correctly. Details of the cross line comparisons are provided in Annex F.

3.5.4 Object Detection

The object detection capability achieved during a LiDAR survey is a function of both the environmental conditions experienced during the survey and the survey parameters adopted for the survey.

The environmental conditions that determine object detection include parameters such as water depth, water clarity, seabed reflectivity and background noise such as sunlight, all of which affect the Signal to Noise Ratio (SNR) of returns and subsequent object detection capability.

In addition, the survey parameters adopted for the survey such as the sounding pattern used (which determines the data density) and the line spacing (which determines the coverage) also influence the object detection capability. In general, 2.5x2.5m laser spot spacing at 100% coverage is recommended to detect objects the size of 2x2m.

Main lines of sounding were flown using the 5x5m laser spot spacing. The BOD (Bottom Object Detection) algorithm was applied to the data to detect small features on the raw laser waveforms. Numerous BOD detections were made, especially in the deeper areas, and where these are considered real they have been retained in the dataset. However, IHO-Order 1 feature detection was not specified in the contract, nor was it achieved in this area.

3.6 Accuracy of Soundings

The depth accuracy of the survey has been assessed theoretically and is presented in Annex G.

The accuracy of the LiDAR returns is also a function of the SNR of returns. Recent analysis of LADS trials data has indicated that a SNR value of 18 or greater (equating to the system quality indicator C0, with a value of 6 or better) is consistent with IHO Order-1 accuracy data.

Given the consistent cross line comparison results, consistent overlap between lines and analysis of C0 values during validation of the data, it is considered that IHO Order-1 depth accuracy was achieved to depths of at least 10 metres.

4. Survey Results

4.1 Completeness

Successful operations were completed in Galway Bay, Tralee Bay, Blacksod Bay and Lough Foyle using the LADS Mk II system and in Greatman's Bay and Cashla Bay (north western Galway Bay), Sligo Bay and Donegal Harbour by the Hawkeye II system. Hawkeye data sets collected in Sligo Bay and Donegal Harbour will be reported on separately.

Coverage achieved in each area is described below.

North Eastern Galway Bay

This area was flown at 5x5m spot spacing at 100% to 200% coverage. Depths were achieved from drying 50 metres to depths of 15 metres (Malin Head). In general data was of a high quality in depths less than 10 metres. Deep areas showed signs of increased levels of noise on the waveforms near the seabed and as such the data is slightly noisier in nature. This increased level of noise was reflected in the cross line comparisons. Some gaps exist along the shore line due to kelp and in the deeper areas due to variable turbidity. There are also some clear gaps south of the sewage works due to persistent turbidity. The required survey area was fully flown.

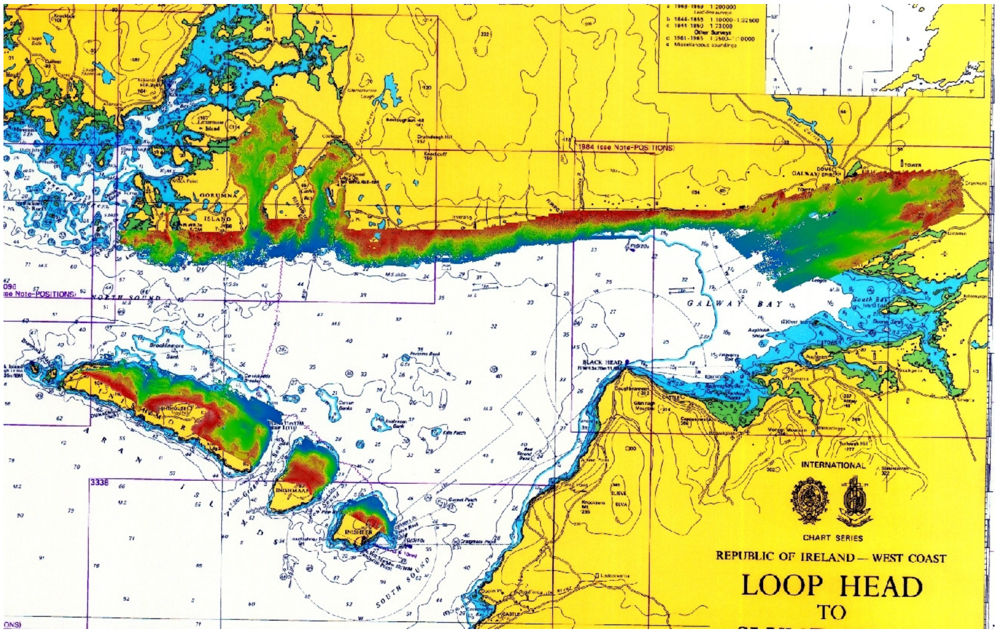

Figure 2 – Chartlet of the Aran Island and Galway Bay Survey areas.

North Coast Galway Bay

This area was flown at 5x5m spot spacing at 200%. Depths were achieved from drying 50 metres to depths of 15 to 20 metres relative to Malin Head. The required survey area was fully covered.

The coast line in this area is particularly steep and rocky in nature, with a large amount of kelp growing along the length of the coastline. This resulted in patches of flat deeper data in sandy areas and large patches of sparse noisy data in shoaler rocky kelp areas. These sparse areas

tended to be shallower than the surrounding sandy areas. Least depths in the rocky kelp areas may not have been achieved, despite being flown at 200%,

Full coverage was achieved inside Greatman's Bay and Cashla Bay using the Hawkeye II system, with overlap between the LADS and Hawkeye systems datasets achieved.

North Coast Aran Islands

This area was flown at 5x5m spot spacing at 200%. Depths were achieved from drying 50 metres to depths of approximately 15 metres relative to Malin Head. The required survey area was fully covered.

Excellent coverage was achieved in the shoaler areas less than 10 metres deep. Deep areas showed signs of increased levels of noise on the waveforms near the seabed and as such the data is slightly noisier in nature. This increased level of noise was reflected in the cross line comparisons.

Tralee Bay

This area was flown at 5x5m spot spacing at 100% to 200%. Depths were achieved from drying 50 metres to depths of 10 to 15 metres relative to Malin Head.

Coverage was achieved throughout the main part of Tralee Bay in depths less than 10 metres, including inside Fenit Harbour and Barrow Harbour. Parts of two lines, one on the north side and one on the south side of the intertidal harbour east of Derrymore Point had to be rejected during the processing due to poor quality leaving striped holes in the dataset. Likewise parts of three lines in the intertidal areas of the northern part of Barrow Harbour and part of one line over the Fahamore Peninsula also had to be removed. Data collected along the northern edge of the survey area in the vicinity of the Seven Hogs and Rough Point in the north west, and Muckaghmore Rock in the north, and west of the Fahamore Peninsula was of a slightly sparse and noisy nature due to near seabed turbidity caused by swell and large kelp areas. Full seabed coverage was not achieved inside the kelp areas where the data was generally shoaler in nature and lesser depths may still exist. This area was outside the required survey area and so further effort into improving coverage in this area was not made.

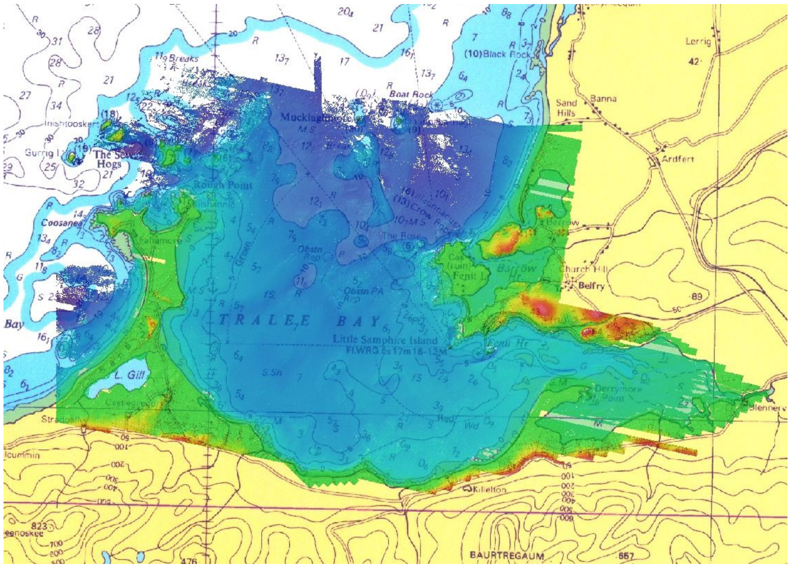

Figure 3 – Chartlet of Tralee Bay survey area.

Lough Foyle

This area was flown at 5x5m spot spacing at 200%. Depths were achieved from drying 50 metres to depths of 10 metres inside the Lough, and 30 metres seaward of the Lough. All depths were reduced relative to Belfast Lough. Operations were suspended once turbid conditions were encountered in the southern and south eastern parts of Lough Foyle. It may be possible to extend coverage further to the south east during an appropriate neap tide window at a later time.

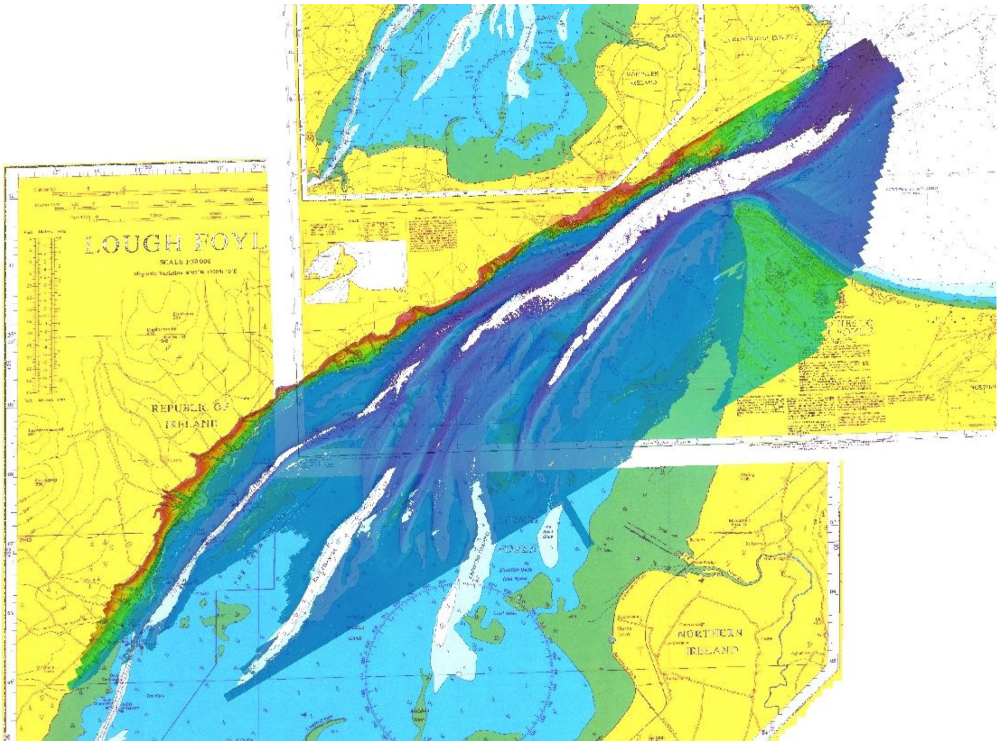

Figure 4 – Chartlet of Lough Foyle survey area.

Blacksod Bay

This area was flown at 5x5m spot spacing at 100%. Depths were achieved from drying 50 metres to depths of 25 metres relative to Malin Head. Operations in Blacksod Bay were optimised to maximise coverage in depths to 10 metres as required by the contract and so deeper coverage was not achieved in the southern part of the bay where it may have been possible to obtain deeper depths.

Only one flight was available for operations in Blacksod Bay, thus the survey of Blacksod Bay was progressed from the south to the north until the completion of the sortie. The northern part of the bay remains unsurveyed, but could easily be surveyed by LADS at a later time.

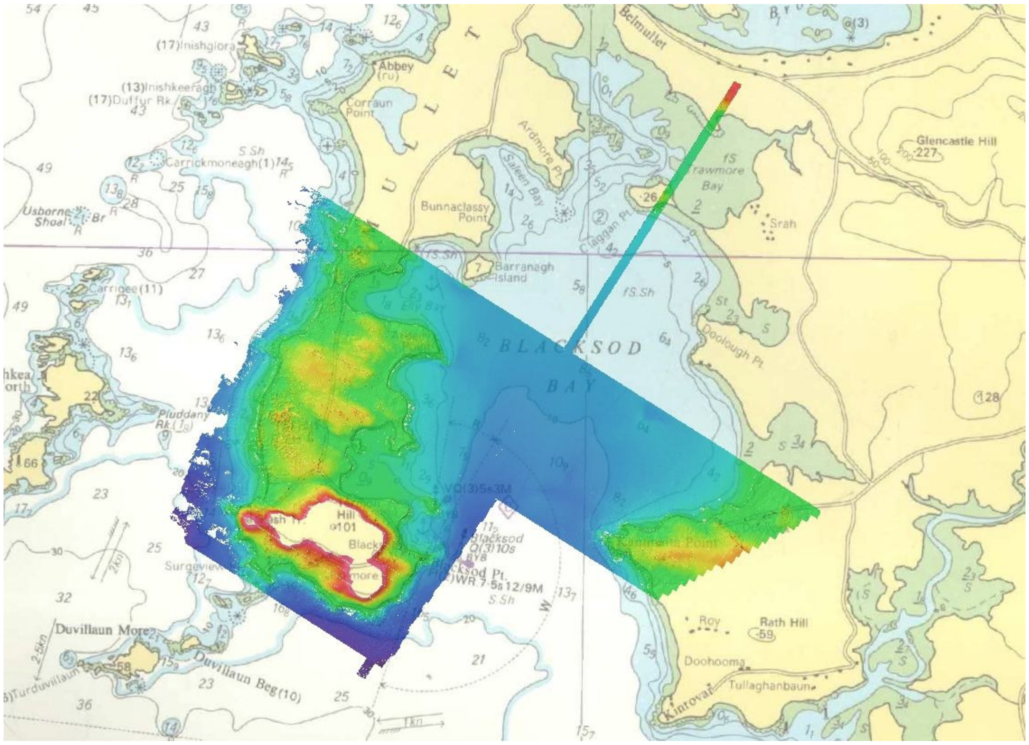

Figure 5 – Chartlet of Blacksod Bay survey area.

4.2 Navigational Aids

Many navigation beacons exist within the survey area marking channels into Tralee Bay and Galway Harbour. Where detected by the system they have been retained, tagged with the appropriate S-57 tag and listed at Annex K. Due to the small size of some of these beacons, not all beacons were detected by the system.

There were also many minor jetties, buoys and moorings detected, however these have not been attributed in the data. The geo-referenced digital imagery provides excellent coverage for the purpose of identifying these features.

The light characteristics of the beacons have not been checked, nor are they indicated on the sheet drawings. Contours around beacons have been removed from the drawings.

4.3 Wrecks and Obstructions

No charted wrecks exist within the survey areas. No wrecks were identified in the data.

Two charted obstructions exist in the Lough Foyle survey area; these features have been identified in the data. Full details are provided at Annex J.

4.4 Coastline and Topography

Within the survey area, soundings were collected to approximately 50 metres above the drying line. The topographic data has been checked for consistency; topographic data with anomalies, such as

errors in datum, has been rejected and removed from the dataset. Individual topographic points have not been validated. The accuracy of the topographic dataset has been assessed to be better than ±1m (95% confidence).

Man made features such as buildings, roads, bridges and breakwaters were also surveyed and are present in the topographic data.

4.5 Features Recommended for Further Investigation

During the survey a number of features were identified inside Lough Foyle which appear on the datasets as shoals and correspond to charted obstructions. The return from the laser of these features was strong enough to indicate a feature exists and they have been retained in the dataset. No additional examination of these features was conducted. Features which fall into this category have been tabulated in Annex J.

4.6 Hawkeye II Operations

A separate report will be provided to cover the Hawkeye operations. A brief description of the Hawkeye system and processes is provided at Annex N.

4.7 Quality Control of Hawkeye data

A comprehensive review and quality control process was conducted by LADS to quality control data supplied by BLOM Aerofilms and incorporated into the Galway Bay survey areas. This process has been described in Annex N.

4.8 Merging of LADS / Hawkeye Datasets

LADS Galway Bay data was merged with the Hawkeye Greatman's Bay and Cashla Bay data to create a merged data set for use in the generation of the required products. For the creation of the merged data file, the fields of 'Eastings', 'Northings' and 'Depths' were extracted from the Hawkeye raw data files for the Malin Head datum reference, and from the LADS data, also to the Malin Head datum reference. The output files were then merged using an AWK script within UNIX. This created an ASCII XYZ file, which was imported into Fledermaus and visualized to confirm correspondence.

This merged LADS / Hawkeye data set was used in the generation of sheet LADS 04, and to create the average depth grid for this sheet.

The Hawkeye LAT data for Greatman's Bay and Cashla Bay has not been merged and will be supplied separately.

5. Deliverables

5.1 Drawings

A set of eight A1 drawings (sheets), covering each survey area to an appropriate scale, have been compiled as agreed with GSI as follows:

Sheet LADS 01	-	Lough Foyle	-	1 x 45 000 Overview Sheet
Sheet LADS 02	-	Blacksod Bay	-	1 x 45 000 Overview Sheet
Sheet LADS 03	-	Tralee Bay	-	1 x 45 000 Overview Sheet
Sheet LADS 04-06	-	Galway Bay	-	3 x 1: 45 000 Sheets
Sheet LADS 07	-	Aran Islands	-	1 x 1:45 000 Overview Sheet
Sheet LADS 08	-	Galway Bay	-	1 x 120 000 Overview sheet

A digital copy of each sheet is also provided in Adobe PDF format and MicroStation DGN format

5.2 Digital Aerial Mosaic

During flying operations digital photographs were taken at 1 Hz (1 second) intervals using a downward looking geo-referenced two mega pixel digital camera. These images were then processed to provide 40cm per pixel resolution. Full coverage was achieved.

Images captured in the main survey areas were then joined together using a semi-automated processing technique to provide a total series of 47 geo-referenced aerial mosaics in 8km tiles, which have been provided as digital data.

These individual tiled mosaics have then been joined together in ArcGIS to form overview mosaics at a reduced resolution for each individual survey area. Full details and a copy of each overview mosaic are provided at Annex M.

5.3 Relative Reflectance Data

Relative reflectance data was been generated from the accepted data in each of the main survey areas.

These files have been generated as ASCII files with the suffix RR1. The file format consists of the beam footprint position in easting and northing then a reflectivity value between 0 and 255. Full details on how the reflectance data was generated area at Annex O.

5.4 Interim Data

An initial set of interim digital data of Galway Bay and Tralee Bay was provided prior to the departure of the survey team on 14 June. A follow on set of interim digital data of Blacksod Bay and Lough Foyle was provided on 31 July 08.

5.5 Final Digital Data

An initial set of final digital data for Galway Bay, Blacksod Bay and Lough Foyle along with digital copies of the associated fairsheets for this data was delivered on 14 November 2008.

Final delivery of all final approved processed data for all survey area, including those previously delivered, accompanies this report; this data has been provided in the following formats:

- 4m shoal bias clashed CARIS .CAF export format for each sheet relative to Malin Head (relative to Belfast Lough for Lough Foyle data),
- 4m shoal bias clashed simplified ASCII x,y,z format for each sheet relative to Malin Head (relative to Belfast Lough for Lough Foyle data). Note for Sheet 04 this data contains merged LADS / Hawkeye data,
- A 6m average grid DTM, ASCII x,y,z format file for each sheet relative to Malin Head (relative to Belfast Lough for Lough Foyle data), Note for Sheet 04 this data contains merged LADS / Hawkeye data,
- A MicroStation .DGN file for each sheet,
- A .PDF file of each sheet,
- Relative Reflectance data set in .RR format for each sheet,
- 4m shoal bias clashed CARIS .CAF export format for each sheet relative to Lowest Astronomical Tide (LAT).
- A Digital Aerial Mosaic of each survey area in GeoTIFF format,
- 8 km Tiled Digital Aerial Mosaics at 40cm resolution.

All digital data has been supplied on a USB Hard Disk, with full details of the supplied data provided at Annex A

Nigel Townsend
Survey Manager - Adelaide
Project Manager
Tenix LADS Corporation Pty Ltd

Mark J. Sinclair
Survey Program Director
Tenix LADS Corporation Pty Ltd

Annexes

A. Accompanying Documents

B. Digital Surveying System

C. Geodetic Records

D. Position Fixing Systems / Position Fixing System Validation Results

E. Vertical Datum, Tide Model and Tidal Stations

F. Checks - Benchmark and Cross Line Comparison Results

G. Vertical Accuracy of Soundings and Horizontal Accuracy of Soundings

H. Summary of Surveying Activity

I. Personnel

J. List of Obstructions and Fish Farms

K. List of Detected Navigational Aids

L. Daily Survey Logs

M. Digital Aerial Mosaics

N Hawakeye II Sensor, Aircraft and Data Quality Control

O Relative Reflectance Data

Annex A. Accompanying Document

A.1 Interim Deliverables

A.1.1 Interim Data Deliveries - 14 June 08

1 x DVD containing interim data was delivered on completion field activities for Galway Bay and Tralee Bay

A.1.2 Interim Data Deliveries - 31 Jul 08

1 x DVD containing digital survey data in the following formats:

- Survey data in ASCII format, clashed at 4 metres for Lough Foyle.
- Survey data in ASCII format, clashed at 4 metres for Blacksod Bay.
- Survey data in CARIS format, clashed at 4 metres for Lough Foyle.
- Survey data in CARIS format, clashed at 4 metres for Blacksod Bay.
-

Reference	Remarks
LAF5C2I01_01.XYZ	ASCII Data (XYZ Export Format)
PARAMS.TXT	Export parameters file, Text Format
BLA5C1I01_01.XYZ	ASCII Data (XYZ Export Format)
PARAMS.TXT	Export parameters file, Text Format
LAF5C2I01_01.CAF	CARIS Export
PARAMS.TXT	Export parameters file, Text Format
BLA5C1I01_01.CAF	CARIS Export
PARAMS.TXT	Export parameters file, Text Format

A.1.3 Interim Approved Survey Data – 14 Nov 08

A.1.3.1 Survey Data

4 X DVDs containing digital survey data for the areas of Galway Bay, Blacksod Bay and Lough Foyle, in the following formats:

- Full density survey data in ASCII format, clashed at 4 metres (all data included), by sheet area.
- Full density survey data in CARIS format, by sheet area.

Reference	Remarks
LOF5C2S01_01.XYZ	ASCII Data (XYZ Export Format)
PARAMS.TXT	Export parameters file, Text Format
LOF5U2S01_03.CAF	CARIS Export
PARAMS.TXT	Export parameters file, Text Format
BLS5C1S02_01.XYZ	ASCII Data (XYZ Export Format)
PARAMS.TXT	Export parameters file, Text Format
BLS5U1S02_03.CAF	CARIS Export
PARAMS.TXT	Export parameters file, Text Format
GAL5C2S04_01.XYZ	ASCII Data (XYZ Export Format)
PARAMS.TXT	Export parameters file, Text Format
GAL5U2S04_03.CAF	CARIS Export
PARAMS.TXT	Export parameters file, Text Format
GAL5C2S05_01.XYZ	ASCII Data (XYZ Export Format)
PARAMS.TXT	Export parameters file, Text Format
GAL5U2S05_03.CAF	CARIS Export
PARAMS.TXT	Export parameters file, Text Format
GAL5C2S06_01.XYZ	ASCII Data (XYZ Export Format)
PARAMS.TXT	Export parameters file, Text Format
GAL5U2S06_03.CAF	CARIS Export
PARAMS.TXT	Export parameters file, Text Format
GAL5C2S07_01.XYZ	ASCII Data (XYZ Export Format)
PARAMS.TXT	Export parameters file, Text Format
GAL5U2S07_03.CAF	CARIS Export
PARAMS.TXT	Export parameters file, Text Format
GAL5C2S08_01.XYZ	ASCII Data (XYZ Export Format)
PARAMS.TXT	Export parameters file, Text Format
GAL5U2S08_03.CAF	CARIS Export
PARAMS.TXT	Export parameters file, Text Format

A.1.3.2 Grid Files

4 X DVDs containing Grid Files for the areas of Galway Bay, Blacksod Bay and Lough Foyle, in the following formats:

- 6m shoal bias depth grids, by sheet area.

Reference	Remarks
LADS_01_6mShoalBias.xyz	ASCII Shoal Bias Grid (XYZ Export Format)
LADS_02_6mShoalBias.xyz	ASCII Shoal Bias Grid (XYZ Export Format)
LADS_04_ 6mShoalBias.xyz	ASCII Shoal Bias Grid (XYZ Export Format)
LADS_05_6mShoalBias.xyz	ASCII Shoal Bias Grid (XYZ Export Format)
LADS_06_6mShoalBias.xyz	ASCII Shoal Bias Grid (XYZ Export Format)
LADS_07_6mShoalBias.xyz	ASCII Shoal Bias Grid (XYZ Export Format)
LADS_08_ 6mShoalBias.xyz	ASCII Shoal Bias Grid (XYZ Export Format)

A.1.3.3 Digital Drawings

4 x DVD's containing digital copies of the drawings in the following formats:

- Set of Contour Drawings (fairsheets), sheets 1 - 7 (excluding sheet 3) at 1:45000 (A1 format), sheet 8 at 1:120000 (A1 format), in MicroStation DGN format (zipped).
- Set of Sun Illuminated Images, sheets 1 - 7 (excluding sheet 3)at 1:45000 (A1 format), sheet 8 at 1:120000 (A1 format). North West angle, in MicroStation DGN format (zipped).
- Set of Sun Illuminated Images, sheets 1 - 7 (excluding sheet 3)at 1:45000 (A1 format), sheet 8 at 1:120000 (A1 format). North East angle, in MicroStation DGN format (zipped).
- Set of Contour Drawings (fairsheets), sheets 1 - 7 (excluding sheet 3)at 1:45000 (A1 format), sheet 8 at 1:120000 (A1 format), Adobe PDF format.
- Set of Sun Illuminated Images, sheets 1 - 7 at 1:45000 (excluding sheet 3) (A1 format), sheet 8 at 1:120000 (A1 format). North West angle, Adobe PDF format.
- Set of Sun Illuminated Images, sheets 1 - 7 at 1:45000 (excluding sheet 3) (A1 format), sheet 8 at 1:120000 (A1 format). North East angle, Adobe PDF format.

Reference	Remarks
LADS_01_FC	MicroStation PZIP
LADS_01_SI1	MicroStation PZIP
LADS_01_SI2	MicroStation PZIP
LADS_02_FC	MicroStation PZIP
LADS_02_SI1	MicroStation PZIP
LADS_02_SI2	MicroStation PZIP
LADS_04_FC	MicroStation PZIP
LADS_04_SI1	MicroStation PZIP
LADS_04_SI2	MicroStation PZIP
LADS_05_FC	MicroStation PZIP
LADS_05_SI1	MicroStation PZIP
LADS_05_SI2	MicroStation PZIP
LADS_06_FC	MicroStation PZIP
LADS_06_SI1	MicroStation PZIP
LADS_06_SI2	MicroStation PZIP
LADS_07_FC	MicroStation PZIP
LADS_07_SI1	MicroStation PZIP
LADS_07_SI2	MicroStation PZIP
LADS_08_FC	MicroStation PZIP
LADS_08_SI1	MicroStation PZIP
LADS_08_SI2	MicroStation PZIP
LADS_01_FC	Fairsheet, Sheet 1, Adobe Acrobat Document
LADS_01_SI1	Sun Illuminated Image (NW), Sheet 1, Adobe Acrobat Document
LADS_01_SI2	Sun Illuminated Image (NE), Sheet 1, Adobe Acrobat Document
LADS_02_FC	Fairsheet, Sheet 2, Adobe Acrobat Document
LADS_02_SI1	Sun Illuminated Image (NW), Sheet 2, Adobe Acrobat Document
LADS_02_SI2	Sun Illuminated Image (NE), Sheet 2, Adobe Acrobat Document
LADS_04_FC	Fairsheet, Sheet 4, Adobe Acrobat Document

LADS_04_SI1	Sun Illuminated Image (NW), Sheet 4, Adobe Acrobat Document
LADS_04_SI2	Sun Illuminated Image (NE), Sheet 4, Adobe Acrobat Document
LADS_05_FC	Fairsheet, Sheet 5, Adobe Acrobat Document
LADS_05_SI1	Sun Illuminated Image (NW), Sheet 5, Adobe Acrobat Document
LADS_05_SI2	Sun Illuminated Image (NE), Sheet 5, Adobe Acrobat Document
LADS_06_FC	Fairsheet, Sheet 6, Adobe Acrobat Document
LADS_06_SI1	Sun Illuminated Image (NW), Sheet 6, Adobe Acrobat Document
LADS_06_SI2	Sun Illuminated Image (NE), Sheet 6, Adobe Acrobat Document
LADS_07_FC	Fairsheet, Sheet 7, Adobe Acrobat Document
LADS_07_SI1	Sun Illuminated Image (NW), Sheet 7, Adobe Acrobat Document
LADS_07_SI2	Sun Illuminated Image (NE), Sheet 7, Adobe Acrobat Document
LADS_08_FC	Fairsheet, Sheet 8, Adobe Acrobat Document
LADS_08_SI1	Sun Illuminated Image (NW), Sheet 8, Adobe Acrobat Document
LADS_08_SI2	Sun Illuminated Image (NE), Sheet 8, Adobe Acrobat Document

A.2 Final Deliverables – 16 Jan 09

A.2.1 Hard Copy Deliverables

A.2.1.1 Report of Survey

- Report of Survey in A4 hardcopy (1 copy: A4 format).

A.2.1.2 Drawings

- Set of Contour Drawings (fairsheets), sheets 1 - 7 at 1:45000 (1 copy: A1 format), sheet 8 at 1:120000 (1 copy: A1 format).
- Set of Sun Illuminated Images, sheets 1 - 7 at 1:45000 (1 copy: A1 format), sheet 8 at 1:120000 (1 copy: A1 format). North West angle.
- Set of Sun Illuminated Images, sheets 1 - 7 at 1:45000 (1 copy: A1 format), sheet 8 at 1:120000 (1 copy: A1 format). North East angle.

Reference	Remarks
LADS_01_FC	Fairsheet, Sheet 1, 1:45000
LADS_01_SI1	Sun Illuminated Image, Sheet 1, 1:45000, NW Angle
LADS_01_SI2	Sun Illuminated Image, Sheet 1, 1:45000, NE Angle
LADS_02_FC	Fairsheet, Sheet 2, 1:45000
LADS_02_SI1	Sun Illuminated Image, Sheet 2, 1:45000, NW Angle
LADS_02_SI2	Sun Illuminated Image, Sheet 2, 1:45000, NE Angle
LADS_03_FC	Fairsheet, Sheet 3, 1:45000
LADS_03_SI1	Sun Illuminated Image, Sheet 3, 1:45000, NW Angle
LADS_03_SI2	Sun Illuminated Image, Sheet 3, 1:45000, NE Angle
LADS_04_FC	Fairsheet, Sheet 4, 1:45000
LADS_04_SI1	Sun Illuminated Image, Sheet 4, 1:45000, NW Angle
LADS_04_SI2	Sun Illuminated Image, Sheet 4, 1:45000, NW Angle
LADS_05_FC	Fairsheet, Sheet 5, 1:45000
LADS_05_SI1	Sun Illuminated Image, Sheet 5, 1:45000, NW Angle
LADS_05_SI2	Sun Illuminated Image, Sheet 5, 1:45000, NE Angle
LADS_06_FC	Fairsheet, Sheet 6, 1:45000
LADS_06_SI1	Sun Illuminated Image, Sheet 6, 1:45000, NW Angle
LADS_06_SI2	Sun Illuminated Image, Sheet 6, 1:45000, NE Angle
LADS_07_FC	Fairsheet, Sheet 7, 1:45000
LADS_07_SI1	Sun Illuminated Image, Sheet 7, 1:45000, NW Angle
LADS_07_SI2	Sun Illuminated Image, Sheet 7, 1:45000, NE Angle
LADS_08_FC	Fairsheet, Sheet 8, 1:120000
LADS_08_SI1	Sun Illuminated Image, Sheet 8, 1:120000, NW Angle
LADS_08_SI2	Sun Illuminated Image, Sheet 8, 1:120000, NE Angle

A.2.2 Digital Data Deliverables

A.2.2.1 Report of Survey

1 x Hard Disk containing the Report of Survey in Adobe PDF format.

Reference	Remarks
Report of Survey	Adobe Acrobat Document

A.2.2.2 Survey Data

1 x Hard Disk containing digital survey data in the following formats:

- Full density survey data in ASCII format, clashed at 4 metres (all data included), by sheet area.
- Full density survey data in CARIS format, by sheet area.
- Relative Reflectivity data, 8 bit (0.255) range, by sheet area.
- Full density survey data in CARIS format reduced to LAT, by sheet area.

Reference	Remarks
LOF5C2S01_01.XYZ	ASCII Data (XYZ Export Format)
PARAMS.TXT	Export parameters file, Text Format
LOF5U2S01_03.CAF	CARIS Export
PARAMS.TXT	Export parameters file, Text Format
LOF5URF01_01.RR1	Relative Reflectance Export
LOF5URF01_01. PRM	Export parameters file, Text Format
LOF5U2LAT_01.CAF	CARIS Export
PARAMS.TXT	Export parameters file, Text Format
BLS5C1S02_01.XYZ	ASCII Data (XYZ Export Format)
PARAMS.TXT	Export parameters file, Text Format
BLS5U1S02_03.CAF	CARIS Export
PARAMS.TXT	Export parameters file, Text Format
BLS5URF02_01.RR1	Relative Reflectance Export
BLS5URF02_01. PRM	Export parameters file, Text Format
BLS5U1LAT_02.CAF	CARIS Export
PARAMS.TXT	Export parameters file, Text Format

TRA5C2S03_01.XYZ	ASCII Data (XYZ Export Format)
PARAMS.TXT	Export parameters file, Text Format
TRA5U2S03_03.CAF	CARIS Export
PARAMS.TXT	Export parameters file, Text Format
TRA5URF03_01.RR1	Relative Reflectance Export
TRA5URF03_01. PRM	Export parameters file, Text Format
TRA5U2LAT_03.CAF	CARIS Export
PARAMS.TXT	Export parameters file, Text Format
GAL5C2S04_01.XYZ	ASCII Data (XYZ Export Format)
PARAMS.TXT	Export parameters file, Text Format
GAL5U2S04_03.CAF	CARIS Export
PARAMS.TXT	Export parameters file, Text Format
GAL5URF04_01.RR1	Relative Reflectance Export
GAL5URF04_01. PRM	Export parameters file, Text Format
GAL5U2LAT_04.CAF	CARIS Export
PARAMS.TXT	Export parameters file, Text Format
GAL5C2S05_01.XYZ	ASCII Data (XYZ Export Format)
PARAMS.TXT	Export parameters file, Text Format
GAL5U2S05_03.CAF	CARIS Export
PARAMS.TXT	Export parameters file, Text Format
GAL5URF05_01.RR1	Relative Reflectance Export
GAL5URF05_01. PRM	Export parameters file, Text Format
GAL5U2LAT_05.CAF	CARIS Export
PARAMS.TXT	Export parameters file, Text Format
GAL5C2S06_01.XYZ	ASCII Data (XYZ Export Format)
PARAMS.TXT	Export parameters file, Text Format
GAL5U2S06_03.CAF	CARIS Export
PARAMS.TXT	Export parameters file, Text Format
GAL5URF06_01.RR1	Relative Reflectance Export
GAL5URF06_01. PRM	Export parameters file, Text Format
GAL5U2LAT_06.CAF	CARIS Export
PARAMS.TXT	Export parameters file, Text Format
GAL5C2S07_01.XYZ	ASCII Data (XYZ Export Format)
PARAMS.TXT	Export parameters file, Text Format
GAL5U2S07_03.CAF	CARIS Export

PARAMS.TXT	Export parameters file, Text Format
GAL5URF07_01.RR1	Relative Reflectance Export
GAL5URF07_01. PRM	Export parameters file, Text Format
GAL5U2LAT_07.CAF	CARIS Export
PARAMS.TXT	Export parameters file, Text Format
GAL5C2S08_01.XYZ	ASCII Data (XYZ Export Format)
PARAMS.TXT	Export parameters file, Text Format
GAL5U2S08_03.CAF	CARIS Export
PARAMS.TXT	Export parameters file, Text Format
GAL5URF08_01.RR1	Relative Reflectance Export
GAL5URF08_01.PRM	Export parameters file, Text Format
GAL5U2LAT_08.CAF	CARIS Export
PARAMS.TXT	Export parameters file, Text Format

A.2.2.3 Grid Files

1 x Hard Disk containing Grid Files:

- 6m average depth grids, by sheet area.

Reference	Remarks
LADS_01_6mAverage.xyz	ASCII Average Grid (XYZ Export Format)
LADS_02_6mAverage.xyz	ASCII Average Grid (XYZ Export Format)
LADS_03_6mAverage.xyz	ASCII Average Grid (XYZ Export Format)
LADS_04_Combined_Data_6mAverage.xyz	ASCII Average Grid (XYZ Export Format)
LADS_05_6mAverage.xyz	ASCII Average Grid (XYZ Export Format)
LADS_06_6mAverage.xyz	ASCII Average Grid (XYZ Export Format)
LADS_07_6mAverage.xyz	ASCII Average Grid (XYZ Export Format)
LADS_08_Combined_Data_6mAverage.xyz	ASCII Average Grid (XYZ Export Format)

A.2.2.4 Digital Drawings

1 x Hard Disk containing digital copies of the drawings in the following formats:

- Set of Contour Drawings (fairsheets), sheets 1 - 7 at 1:45000 (A1 format), sheet 8 at 1:120000 (A1 format), in MicroStation DGN format (zipped).
- Set of Sun Illuminated Images, sheets 1 - 7 at 1:45000 (A1 format), sheet 8 at 1:120000 (A1 format). North West angle, in MicroStation DGN format (zipped).
- Set of Sun Illuminated Images, sheets 1 - 7 at 1:45000 (A1 format), sheet 8 at 1:120000 (A1 format). North East angle, in MicroStation DGN format (zipped).
- Set of Contour Drawings (fairsheets), sheets 1 - 7 at 1:45000 (A1 format), sheet 8 at 1:120000 (A1 format), Adobe PDF format.
- Set of Sun Illuminated Images, sheets 1 - 7 at 1:45000 (A1 format), sheet 8 at 1:120000 (A1 format). North West angle, Adobe PDF format.
- Set of Sun Illuminated Images, sheets 1 - 7 at 1:45000 (A1 format), sheet 8 at 1:120000 (A1 format). North East angle, Adobe PDF format.

Reference	Remarks
LADS_01_FC	MicroStation PZIP
LADS_01_SI1	MicroStation PZIP
LADS_01_SI2	MicroStation PZIP
LADS_02_FC	MicroStation PZIP
LADS_02_SI1	MicroStation PZIP
LADS_02_SI2	MicroStation PZIP
LADS_03_FC	MicroStation PZIP
LADS_03_SI1	MicroStation PZIP
LADS_03_SI2	MicroStation PZIP
LADS_04_FC	MicroStation PZIP
LADS_04_SI1	MicroStation PZIP
LADS_04_SI2	MicroStation PZIP
LADS_05_FC	MicroStation PZIP
LADS_05_SI1	MicroStation PZIP
LADS_05_SI2	MicroStation PZIP
LADS_06_FC	MicroStation PZIP
LADS_06_SI1	MicroStation PZIP

LADS_06_SI2	MicroStation PZIP
LADS_07_FC	MicroStation PZIP
LADS_07_SI1	MicroStation PZIP
LADS_07_SI2	MicroStation PZIP
LADS_08_FC	MicroStation PZIP
LADS_08_SI1	MicroStation PZIP
LADS_08_SI2	MicroStation PZIP
LADS_01_FC	Fairsheet, Sheet 1, Adobe Acrobat Document
LADS_01_SI1	Sun Illuminated Image (NW), Sheet 1, Adobe Acrobat Document
LADS_01_SI2	Sun Illuminated Image (NE), Sheet 1, Adobe Acrobat Document
LADS_02_FC	Fairsheet, Sheet 2, Adobe Acrobat Document
LADS_02_SI1	Sun Illuminated Image (NW), Sheet 2, Adobe Acrobat Document
LADS_02_SI2	Sun Illuminated Image (NE), Sheet 2, Adobe Acrobat Document
LADS_03_FC	Fairsheet, Sheet 2, Adobe Acrobat Document
LADS_03_SI1	Sun Illuminated Image (NW), Sheet 2, Adobe Acrobat Document
LADS_03_SI2	Sun Illuminated Image (NE), Sheet 2, Adobe Acrobat Document
LADS_04_FC	Fairsheet, Sheet 4, Adobe Acrobat Document
LADS_04_SI1	Sun Illuminated Image (NW), Sheet 4, Adobe Acrobat Document
LADS_04_SI2	Sun Illuminated Image (NE), Sheet 4, Adobe Acrobat Document
LADS_05_FC	Fairsheet, Sheet 5, Adobe Acrobat Document
LADS_05_SI1	Sun Illuminated Image (NW), Sheet 5, Adobe Acrobat Document
LADS_05_SI2	Sun Illuminated Image (NE), Sheet 5, Adobe Acrobat Document
LADS_06_FC	Fairsheet, Sheet 6, Adobe Acrobat Document
LADS_06_SI1	Sun Illuminated Image (NW), Sheet 6, Adobe Acrobat Document
LADS_06_SI2	Sun Illuminated Image (NE), Sheet 6, Adobe Acrobat Document
LADS_07_FC	Fairsheet, Sheet 7, Adobe Acrobat Document
LADS_07_SI1	Sun Illuminated Image (NW), Sheet 7, Adobe Acrobat

	Document
LADS_07_SI2	Sun Illuminated Image (NE), Sheet 7, Adobe Acrobat Document
LADS_08_FC	Fairsheet, Sheet 8, Adobe Acrobat Document
LADS_08_SI1	Sun Illuminated Image (NW), Sheet 8, Adobe Acrobat Document
LADS_08_SI2	Sun Illuminated Image (NE), Sheet 8, Adobe Acrobat Document

A.2.2.5 Geo-referenced Photograph Mosaics

1 x Hard Disk containing digital Geo-referenced Mosaics in GeoTiff format, (0.4 metre resolution):

Reference	Remarks
blacksod01	TIF File
blacksod01	TIF File
blacksod02	TFW file
blacksod02	TFW file
blacksod03	TIF File
blacksod03	TFW file
blacksod04	TIF File
blacksod04	TFW file
blacksod05	TIF File
blacksod05	TFW file
blacksod06	TIF File
blacksod06	TFW file
blacksod_overview	TIF File
blacksod_overview	TFW file
buoy01	TIF File
buoy01	TFW file
buoy02	TIF File
buoy02	TFW file
buoy_overview	TIF File
buoy_overview	TFW file
donegal01	TIF File

donegal01	TFW file
donegal02	TIF File
donegal02	TFW file
donegal03	TIF File
donegal03	TFW file
donegal04	TIF File
donegal04	TFW file
donegal05	TIF File
donegal05	TFW file
donegal06	TIF File
donegal06	TFW file
donegal07	TIF File
donegal07	TFW file
donegal_overview	TIF File
donegal_overview	TFW file
galway01	TIF File
galway01	TFW file
galway02	TIF File
galway02	TFW file
galway03	TIF File
galway03	TFW file
galway04	TIF File
galway04	TFW file
galway05	TIF File
galway05	TFW file
galway06	TIF File
galway06	TFW file
galway07	TIF File
galway07	TFW file
galway08	TIF File
galway08	TFW file
galway09	TIF File
galway09	TFW file
galway10	TIF File
galway10	TFW file

galway11	TIF File
galway11	TFW file
galway12	TIF File
galway12	TFW file
galway13	TIF File
galway13	TFW file
galway14	TIF File
galway14	TFW file
galway_overview	TIF File
galway_overview	TFW file
lough_foyle01	TIF File
lough_foyle01	TFW file
lough_foyle02	TIF File
lough_foyle02	TFW file
lough_foyle03	TIF File
lough_foyle03	TFW file
lough_foyle04	TIF File
lough_foyle04	TFW file
lough_foyle05	TIF File
lough_foyle05	TFW file
lough_foyle06	TIF File
lough_foyle06	TFW file
lough_foyle07	TIF File
lough_foyle07	TFW file
lough_foyle_overview	TIF File
lough_foyle_overview	TFW file
tralee01	TIF File
tralee01	TFW file
tralee02	TIF File
tralee02	TFW file
tralee03	TIF File
tralee03	TFW file
tralee04	TIF File
tralee04	TFW file
tralee05	TIF File

tralee05	TFW file
tralee06	TIF File
tralee06	TFW file
tralee07	TIF File
tralee07	TFW file
tralee08	TIF File
tralee08	TFW file
tralee09	TIF File
tralee09	TFW file
tralee10	TIF File
tralee10	TFW file
tralee11	TIF File
tralee11	TFW file
tralee_overview	TIF File
tralee_overview	TFW file

Annex B. LADS Mk II Digital Surveying System

The LADS Mk II hydrographic survey system comprises two main sub-systems: the Airborne System (AS) used for acquiring raw bathymetric data, and the Ground System (GS) which is used to plan operations, calculate depth values from the raw data, provide tools which allow the hydrographic surveyor to validate processed depth values, apply tidal corrections, generate fairsheets and digital survey data and conduct general survey management. Other tools required for quality control activities, in particular contouring and 3-D visualisation software complement these two sub-systems. GPS logging and data processing hardware and software are also provided.

All sounding data are acquired by the AS which is mounted in the LADS Mk II DeHavilland Dash-8 fixed wing aircraft.

The GS software is supported by the UNIX operating system and operates on a ground based server.

Prior to a sortie, planning information is passed from the GS to the AS on disk. During the sortie, logged raw sounding, position and airborne system data is logged on digital linear tape. This is processed on the GS at the completion of each sortie.

The primary Quality Control tools used during this survey were:

- Generic Mapping Tools (GMT)
- Visualisation Tool Kit (VTK)
- Fledermaus
- Geocomp Terramodel
- MicroStation
- ArcGIS

Data is output from the GS in a format suitable for each particular QC tool.

Post-processed GPS positioning is accomplished with Ashtech logging software (datalogr) and post-processed using Novatel GrafNAV software.

B.1 Equipment

This section provides a description of the LADS Mk II Airborne System (AS) and the Ground System (GS).

B.1.1 Airborne System

A laser, scanner, optical system, photo-multiplier tube and conditioning electronics collect the raw sounding signal. These items are mounted on a stabilised platform controlled via servo systems using information from an Attitude and Heading Reference System (AHRS) mounted on the platform. Aircraft position information is obtained from the Global Positioning System. Three computers, linked via an FDDI optic fibre network, control and monitor the AS operations. These computers are:

- The System Control Computer (SCC) for operator interface, logging and overall system coordination.
- The Navigation System and Support (NSS) computer for position monitoring and control.
- The Laser Control and Acquisition (LCA) computer for control of the scanner and laser and digitisation of raw sounding data. The LCA also synchronises overall AS timing.

AS system time is synchronised with GPS time and all data acquired for logging is appropriately time stamped at the point of acquisition then passed to the SCC to be written to digital linear tape.

Ancillary equipment includes:

- A downward looking video camera and VCR to provide images below the aircraft and a forward looking video camera.
- A downward looking Redlake MegaPlus II ES 2020 digital camera to capture digital imagery below the aircraft.
- Systems for temperature control of equipment.
- VHF transceiver and aircraft intercom.
- Satellite phone.

The operator interface allows the operator to monitor the quality of sounding, position and other data in order to set appropriate system parameters and control the sequence of sortie operations.

Detailed descriptions of the main AS components and their functions are given under the following headings below. Each of these components were checked by the Tenix LADS Technical Department during trials and acceptance flights conducted during March 2007, in order to achieve the requirements of the LADS Mk II Performance Verification Certificate (provided in Enclosure 1).

- Sounding Equipment
- Positioning Equipment
- Sortie Control
- Ancillary Equipment
- Operator Interface

B.1.2 Sounding equipment

Soundings in the LADS Mk II system are obtained by the transmission of laser pulses from the aircraft through a scanning system and detecting return signals from land, the sea surface, the water body and the seabed. The transmitting and receiving components are housed on a stabilised platform that compensates for aircraft pitch and roll. The return signals are electronically amplified and conditioned prior to being digitised and logged.

The primary sounding components of the AS are:

- Laser. A Nd: Yag laser producing infrared energy at a wavelength of 1064nm at 990 pulses per second of which 900 pulses are used for sounding purposes.
- Optical Coupler. The optical coupler is used to split the infrared beam. Part of the IR beam is transmitted vertically to nadir on the sea surface. The other part of the split beam is frequency doubled to produce green laser pulses of wavelength 532nm. The green pulses are transmitted onto the mirror of the scanner.
- Scanning System. The scanning mirror is oscillated in both the major (across track) and minor (along track) axes. The required scan pattern is generated by controlling software. All possible patterns are listed in B.9, Sounding Patterns section.
- Optical Receivers. The IR and green return signals are detected by two separate receivers. The IR return from the surface of the sea is used to establish a height datum. The IR receiver is a solid state detector producing an electronic signal from the IR return. The green return comprises energy returned from the surface, subsurface and seabed and is used to determine water depth. The green return is transmitted via the scanner into a photomultiplier tube. The electronic output of the two return signals are electronically mixed prior to digitisation.
- Attitude and Heading Reference System (AHRS). The AHRS is a laser gyro inertial navigation system providing platform attitude information to the platform servo system that in turn maintains platform stability. The AHRS also reports platform attitude to the LCA computer and provides height data.
- LCA computer. This controls the laser and scanner operations and digitises (8 bits at 500MHz) appropriate sections of the composite electronic red/green return signal along with platform attitude data and other system parameters. This digital information is passed to the System Control Computer (SCC) where it is logged to digital linear tape.
- Waveform Display. This CRT display presents the operator with sounding waveforms as digitised and is used by the operator to check data quality during acquisition.

B.1.3 Position equipment

The centre of the scanning mirror is the survey reference point on the aircraft. The GPS antenna is positioned relative to this point as described in B.7, Laybacks.

The signal from the antenna is split and fed to two independent GPS receivers: an Ashtech GG24 single frequency GPS receiver used for real-time aircraft position fixing and track keeping and an Ashtech Z12 dual frequency GPS receiver is used to compute post-processed KGPS positions. The data from this receiver is independently logged and post-processed as described in Annex C.

The output of the real-time GPS receiver is fed to the NSS to:

- fix aircraft position and determine ground speed
- calculate aircraft cross track error and automatically maintain track along survey lines
- provide pilot display information
- establish and maintain system UTC time.

The NSS passes the received GPS and derived information to the SCC computer for logging.

B.2 Sortie control

A sortie plan is generated on the GS to transfer survey information to the AS. The sortie plan contains spheroidal, grid and magnetic variation parameters and a list of survey objectives including the line number, start/end coordinates and coordinates for navigation checks. During the course of the sortie, the airborne operator amends the sequence of execution to suit local conditions and can amend the scan pattern parameters for the survey lines to suit survey requirements.

The SC computer controls the sequence of survey operations by:

- planning all required flight paths and communicating these to the NSS
- transmitting required parameters for scan patterns, aircraft altitude, etc. to the LCA
- initiating the starting and stopping of system operations, via commands sent to the LCA and NSS at specific waypoints on the run-in and run-out of survey lines.

The operator may abort and restart the sortie operations at any time and the sequence of objectives may be amended at any time. Scan patterns can be amended on all lines except the executing objective. A display of the planned survey line and received GPS data is situated in the cockpit and used to advise the pilots of required aircraft configurations. The display provides an indication of cross track error with required and actual values for altitude and ground speed.

Aircraft position during survey acquisition is under automatic control of the NSS via the aircraft autopilot. Aircraft turns are under pilot control assisted by the display. Aircraft altitude and speed are under pilot control, and communication between the operator and pilots is via the aircraft intercom system.

The management of survey operations can be impacted by both low cloud and high ground in the survey area. LADS Mk II is able to operate at different survey heights so that adequate clearances can be maintained while surveying and survey activities can continue below low cloud ceilings. Survey altitudes at 200ft increments are available from 1200 to 2200 feet (366 to 671m). Altitudes must be constant for the duration of a survey line but may be varied from line to line by the AS operator during the course of a sortie.

During daytime operations a narrow band green filter is used to filter out other light frequencies from the photomultiplier tube. This filter has a slight attenuating effect on the laser returns, which reduces the maximum depth performance. This filter can be removed once the ambient sunlight levels drop which results in improved performance at night.

Glassy sea conditions may result in very strong IR surface returns that can saturate the IR receiver causing a loss of surface datum. The AS monitors the IR surface return performance and advises the operator if IR saturation occurs. The operator can activate an attenuator that provides correct IR surface return amplitudes to be fed to the IR receiver. Should sea surface conditions change which may result in lower IR return amplitudes the AS informs the operator to deactivate the attenuator.

The laser is designed to be eye safe in accordance with the following standards:

a. ANSI Z136.1-2000 American National Standard for Safe Use of Lasers.

b. IEC 60825-1 (Edition 1.2) International Standard – Safety of Laser Products.

c. AS/NZS 2211.1 Supplement 1:1999 Australian/New Zealand Standard Laser Safety.

The laser power can be reduced by a further factor of four using a built-in attenuator. The operator may activate/deactivate the attenuator at any time.

B.3 Ancillary equipment

A video camera is positioned on the stabilised platform and directed downward at nadir. A calibrated graticule is superimposed on the camera image to provide the operator with a scan width and distance reference. The image, graticule and other relevant system information including position and time are presented to the operator and recorded throughout a sortie.

A forward-looking video camera is also provided to assist the AS operator for the purpose of evaluating the conditions ahead of the aircraft.

A digital imagery system provides geo-referenced imagery. This system comprises of a RedLake MegaPlus II ES 2020 digital camera, a Matrox 4sight M frame grabber and a Matrox embedded computer running Windows EP embedded operating systems. Images are taken at one-second intervals with a 1600x1200 resolution and a 2-megapixel interline-transfer camera head and controller. At the end of each sortie, the images are copied to the LADS GS using a removable hard drive.

B.4 Operator interface

The operator monitors and controls system operation from the console. The following key information is provided to monitor system performance:

- Sortie Information. The Sortie ID, spheroid and grid in use and available survey objectives are displayed. Sortie objective information includes the scan pattern set for the objective and estimated time to complete the objective.
- Objective Information. The Objective ID, selected scan pattern, required speed and altitude pertaining to the current objective being executed and objective status such as time to completion are presented.
- Waveform Display. This display is a CRT on which is displayed each of the mixed red/green sounding return signals as digitised by the LCA (the traces are overlayed). The operator continually assesses this display to determine data quality.

- Depth Profile. A depth profile determined from nadir soundings is available to the operator with an associated confidence factor. As the algorithm is limited by real-time considerations these depths and confidences are only indicative.
- Aircraft Position, Speed, Altitude and Cross Track Error. A number of displays including a copy of the pilot display are available to the operator to determine the aircraft position and performance parameters. Speed and altitude are continually monitored and the pilot informed of deviations from the desired values.
- GPS status. The operator is provided with the data from the GPS receiver including number of satellites, satellite altitudes and azimuths, S/N ratio and which satellites are being used.
- Equipment Status. System status and performance parameters are available to the operator including laser power and temperature, dynamic gain values, AHRS status and scanner performance.

Items controlled by the operator for sortie execution and data acquisition are:

- sequence of objective execution
- scan pattern for each objective
- operating height for each objective
- depth logging range and topographic height range for each objective
- dynamic gain limits
- IR and Green receiver attenuator positions.

B.5 Depth and topographic mode

During normal bathymetric survey mode (Depth Mode) LADS Mk II determines the depth of water with the height datum being determined from the reflected IR laser signal, GPS height and AHRS height. When over land this IR signal is not valid and the height datum is obtained from the GPS and AHRS.

This ancillary height datum allows LADS Mk II to measure topographic heights. The topographic height range is dependent on the depth range being used.

B.6 LADS Mk II aircraft and system specifications

Aircraft Type	DeHavilland Dash 8-200, twin turbo prop, high wing
Aircraft Modifications	Long range tanks, pressurised laser bay window and autopilot interface
Transit Cruise Speed	250 knots (maximum 275 knots)
Transit Altitude	To 25000ft
Survey Speed	Dependant on Scan Pattern: Nominal 140 – 210 knots (72-108 metres per second)
Survey Height	1200 to 2200ft (366 to 671m) in 200 ft increments
Survey Track-Keeping	+/- 5 m (manual or via autopilot coupling)
Survey Endurance	8 hours nominal
Operational Capability	Day/Night operation
Depth Sounding Rate	900 soundings per second
Swath Width	Dependant on Scan Pattern: Nominal 50 – 288m (independent of aircraft height and water depth)
Scan Pattern	Rectilinear
Sounding Density	Variable: 6x6m, 6x5m, 5x5m, 4x4m, 3x3m, 2.5x2.5m and 2x2m
Soundings per sq km	Dependant on scan pattern. For 4x4m – 75000/ km^2 (assuming 32m overlap)
Soundings per hour	Up to 3 million
Topographic and Depth Range	-50m (topo) to 70m (depth)
Area Coverage	Dependant on scan pattern. For 4x4m – up to 41.5km^2/hour (12.1 sq nm/hr) assuming 32m overlap
Position Fixing	Autonomous GPS and post-processed L1+L2 dual frequency KGPS
Recording Media	DLT, VHS Video Tape, USB Hard Drive
Digital Camera	Image Area at 1500ft operating altitude: ~330m x 250m.<br>Image Resolution: >4 pixels/m at an altitude of 1600ft.<br>Digital Image Capture Rate: 1 per second.<br>Digital Image Horizontal Accuracy: +/-5m (95% confidence).

B.7 Laybacks

All laybacks are measured relative to the survey reference position on the aircraft which is the centre of the scanning mirror. The GPS antenna used for position determinations in the AS is positioned on the upper side of the aircraft fuselage forward and to the left (facing forward) of the sounding reference position. The signal from this antenna is passed to a splitter, one signal going to the GPS receiver in the Navigation System computer and the other passes to the GPS airborne logger.

Offsets are from the sounding reference point to the antenna with the following axis and sign convention assuming the aircraft is level:

X positive toward the nose of the aircraft

Y positive to the left facing forward

Z positive vertically up

The offsets are:

X offset: + 1.895m

Y offset: + 0.43m

Z offset: + 2.45m

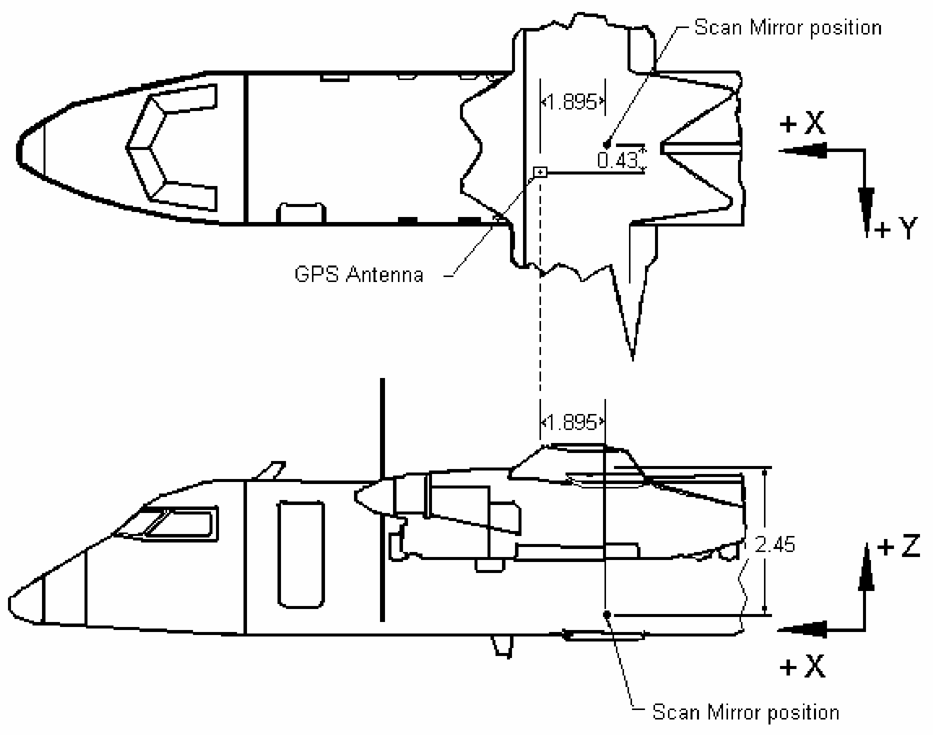

Figure 1 – Laybacks

B.8 Logging parameters

B.8.1 Position fixing

The Airborne System obtains a position fix every 0.5 seconds.

B.8.2 Navigation update

While executing a survey line under AS control navigation correction is passed to the aircraft autopilot every 0.5 seconds.

B.8.3 Post-Processed GPS

The GPS airborne and base logging stations log position information from GPS satellites at 0.5 second intervals.

B.8.4 Sounding rates

LADS Mk II obtains depth soundings in a rectilinear pattern where the sounding density is variable (see Table 1) but sounding rate is invariant.

For all sounding patterns the soundings are grouped into one second frames made up of 18 scan lines. Each of the 18 scan lines contain 50 laser pulses, of which 48 pulses are used for depth sounding. The outermost laser pulses are not used for depth sounding. This provides an effective sounding rate of 864 soundings per second.

B.9 Sounding patterns

LADS Mk II has variable scan pattern functionality as detailed in the following table. The 4x4 and 4ax4a patterns both provide 4x4 metre spot density but have different swath width and survey speeds. All patterns are available at each of the operational altitudes (1200 –2200ft at 200ft increments).

Sounding Density (m)	Swath Width (m)	Line Spacing 200% Coverage (m)	Line Spacing 100% Coverage (m)	Survey Speed m/sec (kts)
6x6	288	125	250	108 (210)
5x6	288	130	260	90 (175)
5x5	240	100	200	90 (175)
4x4	192	80	160	72 (140)
4ax4a	150	60	120	90 (175)
3x3	100	40	80	77 (150)
2x2	50	20	40	72 (140)

Table 1 – Scan configuration

B.10 Ground System

The GS provides the facilities for all LADS survey management tasks from initial mission planning through to production of fairsheets and deliverable digital data.

The primary functions are:

- Mission planning. This includes the specification of the total survey area, spheroid and grid, survey sub-areas, line spacing, swath widths, survey lines to cover the sub-area, individual survey lines, cross lines, tidal areas and navigation check points.
- Sortie planning. A sortie plan is the specification of a series of survey objectives to be executed by the AS. Survey lines and navigation check objectives are selected by the operator and written to disk along with grid and spheroidal information.
- Sortie processing. This function calculates sounding depths and positions from the raw sounding data logged by the AS. Depths and positions are associated with various confidence metrics.
- Data validation, checking and approval. Surveyors validate the calculated soundings on a run by run basis editing soundings as appropriate. The validated data is checked by a more senior surveyor and finally approved by the Field Party Leader.
- Data output. Approved data is output to the client in digital form along with hardcopy fair sheets.

In addition, the GS provides facilities for the generation of survey management plots and reports.

B.10.1 Mission planning

At the commencement of a survey one or more databases are established on the GS. Each database contains spheroid and grid data, tide data and survey objectives.

Sub-areas are defined covering the specific areas to be surveyed. Survey lines are then generated within each sub-area at an operator specified line spacing. Other survey lines can be specified by entering start and end coordinates.

B.10.2 Sortie planning

Prior to each sortie survey objectives are selected from the appropriate database. The start and end coordinates of the required survey lines are written, together with spheroid and grid data, to a sortie plan on disk. This plan is read by the AS and used to control sortie operations.

B.10.3 Data processing

Processing parameters suitable for the sortie are set prior to processing. The post-processed KGPS positions from the local GPS base station are applied to the data.

Raw sounding data logged by the AS is automatically processed by the GS to produce depth, position and a series of confidence parameters.

Preliminary tides are applied and final verified tides can be reapplied at a later time.

On completion of automatic line processing; operator validation, checking and approval of the sounding data can be conducted.

B.10.4 Data organisation

Data within the GS database is held on a line by line basis. Within lines, data is grouped into one second frames made up of 18 scans of 48 sounding pulses ie. 864 pulses per frame. (The outer two laser pulses are not used for sounding purposes.)

B.10.5 Primary and secondary soundings

All soundings comprise the primary sounding set. Where data set reduction is required a shoal biased subset of the primary soundings called secondary soundings is created. Secondary soundings form a shoal biased sub-set based on operator selected confidence and secondary selection radius criteria. Only secondary soundings are validated, checked, approved and output. For this survey a secondary sounding reduction radial of one metre has been used which means all soundings have been hydrographically reviewed and all valid soundings have been provided in the final data set.

B.10.6 Automatic data processing

Automatic processing is completed in two stages:

1. Sortie Tape Processing (STP). STP reads the data on the tape and stores it in the internal GS database for further processing. The data is line based, and consists of raw waveform data, navigation data, platform data, system data, and error and event logs. This process also includes producing a backup of the Raw Data Tape on DAT or DLT.
2. Sortie Run Processing (SRP). SRP is the second and major processing phase during which sounding depths and positions are calculated on a line by line basis. The process is normally triggered automatically by STP as each line becomes available, but may be invoked later by the operator if reprocessing of lines with different processing parameters is required.

The major processing steps of SRP are:

- Apply post-processed KGPS positions to the raw data and digital images from the downward looking camera.
- Process the Raw Waveform to identify surface reflections.
- Process the Raw Waveform to identify and calculate initial depths for the two most likely bottom return pulses.
- Classify each of the identified bottom return pulses by signal noise ratio, agreement with near neighbours and a maximum likelihood estimator.
- Select the most likely bottom return pulse based on the above classification and a shoal weighting function.
- Model the sea surface from the available surface pulses.
- Correct the bottom depths for sea surface datum including tide, slant range, optical propagation and early/late entry. Tidal corrections may be reapplied later if required.
- Calculate position of each sounding on the seabed. This algorithm uses corrected GPS fixes, aircraft track and speed, antenna offsets, platform attitude (heading, roll and pitch), beam scan angles and sounding depth. Where the GS is unable to determine a depth from the raw data the sounding is classified as “No Bottom Detected” (NBD).

- Calculate primary confidence indices (0-9) for each non-NBD sounding and all frames where:

  C0 = Subsurface Pulse Confidence (signal to noise)

  C1 = Near Neighbour Confidence

  C2 = Pulse Type Confidence

  C3 = Position Confidence

  C4 = Sea Surface Reference Confidence

  C5 = Not Used

  C6 = Coverage Confidence (confidence that the swath covered the planned width)

  CW = Weighted Primary Confidence
- Store each sounding and associated confidence data in the database.

B.10.7 Bottom Object Detection (BOD)

A particular feature in the SRP improves the ability of the LADS Mk II GS to detect small objects on the seabed.

The BOD algorithm proceeds in two phases, each phase can be independently enabled/disabled and tuned via a series of BOD processing parameters set by the operator prior to SRP processing.

Phase one of the algorithm is designed to detect objects 2-3m in height while phase two is only invoked if phase one fails. Phase two is more sensitive and intended to find objects less than 2m in height.

B.10.8 Line reprocessing and segmentation

It may be necessary to reprocess the same raw sounding data with different processing parameters. The run identification scheme adopted in LADS Mk II provides a mechanism to manage the reprocessing of survey line data a number of times.

After a line is reprocessed the required segment can then be set to accepted, and the remaining data can be set to anomalous or rejected and is therefore ignored by the system.

B.11 Quality Control

B.11.1 Data processing

Data processing involves the following stages:

- Automatic Data Processing, described earlier
- Pre-Validation and initial batch and filter cleaning of the data by survey personnel under the supervision of the senior hydrographic surveyor
- Validation of the data
- Checking of the data by a Hydrographic Surveyor / Degree Surveyor
- Visualisation of the data
- Approval of the data

B.11.2 Validation

Validation proceeds through the following steps:

Examining the Depth Profile for the correct processing of each expected Survey Run.

Examining a range of position, coverage and system performance confidences to ensure only good data is accepted.

Resolving anomalous soundings by examining data points in the Survey Run by checking:

a. the Primary Depth Display

b. the Waterfall Display

c. the Waveform Display

d. the Local Area Display

Editing operations include selection of the alternate depth, assignment of NBA or deletion of the sounding as appropriate.

Based on assessments made in the above steps the operator segments the line classifying each segment as:

a. Accepted

b. Anomalous, (data not to be used)

c. Rejected, (for refly)

All operator interactions during the validation phase are logged so that complete traceability is maintained.

The imagery, collected by the downward-looking digital camera in real-time, is processed along with the raw data. These images are geo-referenced and can be either manually or automatically displayed alongside of the Raw Data Display, the Waveform Display or the Local Area Display. The images are automatically rotated to fit the current display and are used during all phases of data processing.

These images are displayed in the GS Digital Image Window on the second dual screen monitor. This display is automatically linked to all of the GS displays mentioned previously and the selected sounding is highlighted in the downward-looking image with a yellow circle of 5m diameter.

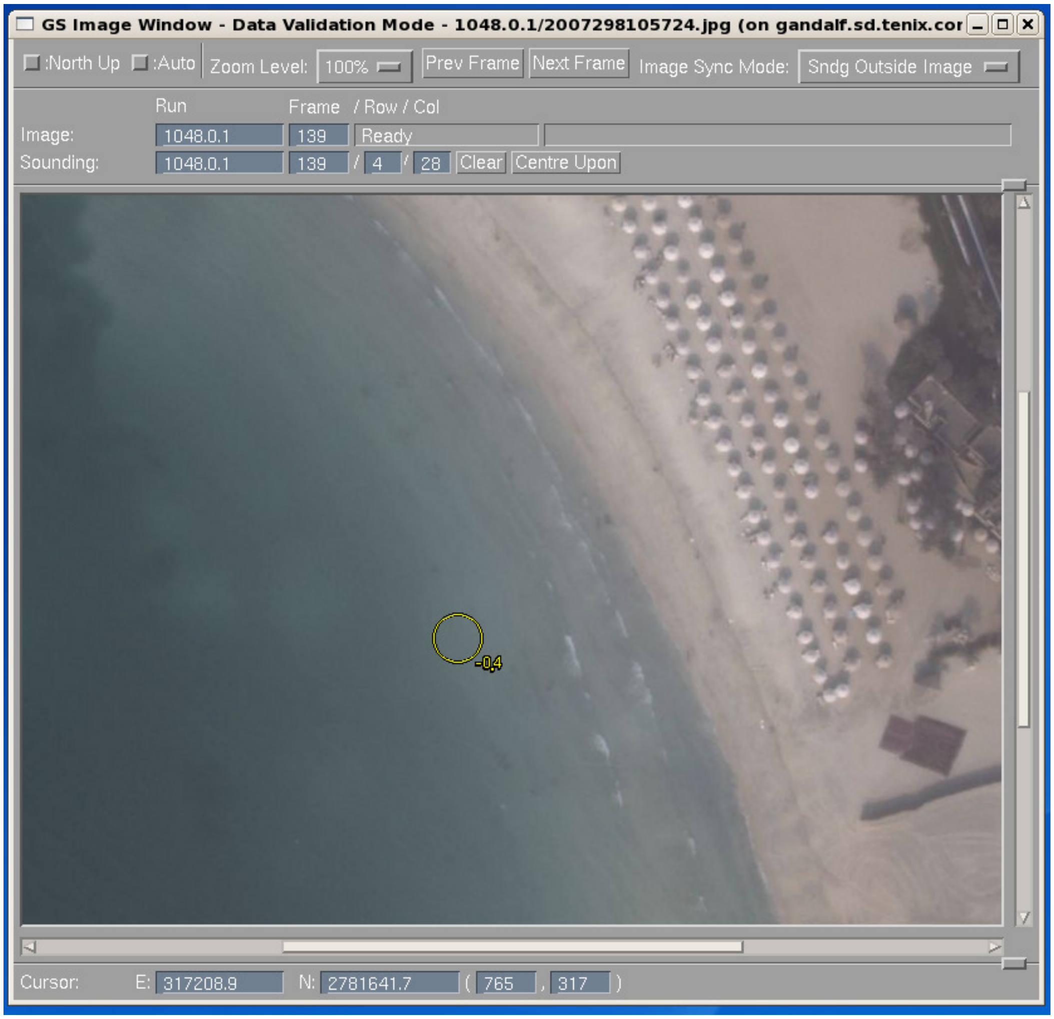

Figure 2

The GS Digital Image Window enables the operator to easily correlate features such as coastline, islands, islets, drying rocks, rocks awash, shallow rocks, kelp, beacons, buoys, boats, jetties, buildings and trees in the image with the data presented in the different GS displays. The quality of imagery and zoom functionality of the window even enables discernment of biological data artefacts, such as bird strikes and whale returns.

B.11.3 Checking

When a line has been validated it is passed to a checker. All edits made by the validator are marked on the line and logged in a validation log. The checker independently assesses the line and checks the validation edits.

B.11.4 Data visualisation

All validated and approved data is exported from the GS in a defined ASCII format for spatial presentation and checking. The position, depth, run and other relevant information are extracted from the line-based data for use in the generation of TIN models and gridded data sets. Both of these are used to produce contour plots, sun-illuminated colour banded images and coverage

check plots. Anomalies found in these plots are reported back to the checkers for remedial action in the GS.

A number of software packages are used to produce these QC products namely:

- Generic Mapping Tools (GMT)
- Visualisation Tool Kit (VTK)
- Fledermaus
- Geocomp Terramodel
- MicroStation
- ArcGIS

B.11.5 Approval

In the final phase an IHO Category A qualified Hydrographic Surveyor reviews each line and approves the data for delivery. All actions in validation, checking and approval are logged on appropriate forms and the procedures used have been certified as conforming to ISO-9001 Quality Assurance standards.

B.11.6 Audit trail

All actions in validation, checking and approval are logged on appropriate forms and the procedures used have been certified as conforming to ISO-9001 Quality Assurance standards. In addition, all operator actions are logged by the GS.

B.11.7 Tagging of Soundings in the GS

During data processing on the GS, the operators have the ability to assign S-57 and user-defined tags to gaps and features in the data. This enables accurate delineation and attribution of features using the S-57 feature file.

All actions in validation, checking and approval are logged on appropriate forms and the procedures used have been certified as conforming to ISO-9001 Quality Assurance standards. In addition, all operator actions are logged by the GS.

B.11.8 Database management and survey line identification

Run Numbers	Density (m)	Remarks
50 - 72	5x5	Cross lines.
100 - 207	5x5	Mainlines - Galway Bay.
230 - 268	5x5	Mainlines – Aran Islands.
500 - 634	5x5	Mainlines – Lough Foyle
635 - 641	5x5	Additional Mainlines –Galway Bay
700 - 705	5x5	Coverage Lines – Miscellaneous Areas
1000 - 1084	5x5	Additional Mainlines –Donegal
1100 - 1353	5x5	Mainlines – Tralee Bay
1400 - 1429	5x5	Mainlines – Ocean Buoy
1450 – 1478	5x5	Additional Mainlines –Tralee Bay
1500 – 1511	5x5	Additional Mainlines –Tralee Bay
1800 – 1951	5x5	Mainlines – Blacksod Bay

Table 2 - Database line planning and numbering

B.11.9 Line identifiers

Line identifiers within the LADS Mk II system uniquely define a specific line and are made up of 4 fields separated with a point '.' as follows:

(Items in <> are the generic names for the fields.)

<LineNumber>.<Section>.<Sequence>.<Child>

eg. 498.1.2.3

Maximum fields are 100000.99.99.9

LineNumber – Range 1..100000
This field uniquely defines the line and is chosen by the operator when defining a line.

Section – Range 0..99
This field denotes the section of the line.

Zero indicates the whole original line. When the line or part of the line is reflown the section number is incremented. Thus:

- 498.0.x.x is the original line
- 498.1.x.x is the first refly
- 498.2.x.x is the second refly.

Sequence – Range 1..99
This field denotes the number of times the logged data for the specific <LineNumber>.<Section> has been processed. Each time a line is processed by the Sortie Run Process (SRP) function the GS allocates a new sequence number for the line. Thus:

- 498.0.1.x is the first processing of the original line
- 498.0.2.x is the second processing of the original line
- 498.1.1.x is the first processing of the first refly
- 498.1.2.x is the second processing of the first refly.

Child – Range 1..9
This field denotes the segment (or child section) of a <LineNumber> .<Section>.<Sequence>.

Hydrographic surveyors divide lines into ACCEPTED, REJECTED or ANOMALOUS segments during the Line Validation process, these segments are given sequential child numbers. Thus:

498.0.1.1 – is the first child (segment) of the first processing of the original line.
This provides the mechanism of ensuring only ACCEPTED data is output for products.

B.11.10 Software versions

The following software versions were used during survey operations.

System	Version	Remarks
Airborne System	AS 9.0.4	
Airborne System GPS Receiving System	-	Fugro OmniStar Virtual Base Station Service<br>Fugro OmniStar 3500LR Receiver<br>Ashtech GG24 GPS Receiver
GPS Base Station Receiver	-	Ashtech Z12
GPS Airborne Receiver	-	Ashtech Z12
GPS Logging	5.6.0	Ashtech Datalogger Software
GPS Processing	8.1.2110	Novatel GrafNAV 8.1 (Precise Differential GPS Navigation Trajectory Software)
Ground System	E8.5/010	Version in use at Mission Start - May 2008
Visualisation and QC	10.42<br>3.3.1<br>4.2.2<br>6.5.18<br>9.2<br>08.09.04.51	Terramodel<br>Generic Mapping Tools (GMT)<br>Visualisation Tool Kit (VTK)<br>Fledermaus<br>ArcGIS<br>MicroStation V8 XM

Table 3 – Software versions

B.11.11 Processing parameters

Each survey line is processed with a specific set of processing parameters, with the set used for the line recorded on the Survey Line History Sheet for the line. Full details are recorded in the Survey Data Management Folder held by TLC.

B.12 Data output

The data is delivered in XYZ, ASCII format based on the sheet limits clashed at a 3 metre radial range. All files have been written to DVD (Juliet format)

B.12.1 File Naming Conventions

All file prefixes are named using the following convention.

<AreaId>	: 3 alphanumeric character field specifying the specific project area (GAL – Galway Bay, TRA – Tralee Bay, BLS – Blacksod Bay, LOF – Lough Foyle)
<SoundingDensity >	: 1 numeric character specifying the sounding density – one of 2,3,4,5,6<br>e.g. 5 indicates 5 x 5 metre spot spacing
<ClashStatus>	: 1 alphanumeric character from C (clashed), U (unclashed).
<%Coverage >	: 1 numeric character indicating percentage coverage in flown lines<br>e.g. 1 indicates 100%, 2 indicates 200% etc
<ExportType>	: 1 alpha character indicating purpose of export. Delivered files will have one of I (intermediate delivery), F(Final delivery), S(Fairsheet data), ALL (entire survey area, LAT for LAT dataset)
<Version>	: character alpha specifying export type e.g. _01(XYZ), _02 (CAF)
<Extension>	: .XYZ for ASCII x,y,z (Eastings, Northings, Depth) files<br>.CAF for CARIS ASCII files

For example, the standard prefix for XYZ data of Galway Bay, surveyed at 5 x 5 metre spot spacing unclashed at 100% of entire area containing version type, the file name would be:

GAL5U1ALL_01.xyz

Enclosure:

1. LADS MkII Performance Verification Certificate
2. Specification for LADS MkII, CARIS Data Export Format
3. Specification for LADS MkII, XYZ ASCII Data Export Format

Enclosure 1: LADS MkII Performance Verification Certificate

Tenix

TenixLADSCorporation

LADS Mk II Performance Verification Certificate

The LADS Mk II System was flown for system performance verification over Gulf St Vincent, South Australia, Sorties 3 to 10, during March 2007.

A well-surveyed benchmark run was used to verify depth and position, with the latter by using submerged targets at known positions in the benchmark area. The system performance was verified as meeting the requirements of IHO Order 1 depth and horizontal accuracy.

Approving Authority: M. Penly 2 / 8 / 07

Mark Penly, Technical Manager

Quality System
Quality Endorsed Company
ISO9001 Lic20702

Enclosure 2: Specifications for LADS MkII, CARIS Data Export Format

SPECIFICATION - LADS CARIS EXPORT FORMAT

This is a controlled document.

Copy No:

Issue No: 1.00

LADS Mark II

Ground System Output Format Specification for CARIS

Document Number: LADS2A05.001.008

Commercial-in-Confidence

Authorised by: M. Penley

Date: 6-5-03

This document contains information which is the property of Tenix LADS Corporation and may not be reproduced, copied or disclosed in any form to a third party without the written permission of Tenix LADS Corporation.

Tenix LADS Corporation

Amendment Status

As necessary, authorised amendments will be issued to holders of this document. Amendments will take the form of replacement or additional pages.

The amendment status appears at the top right side of each page. For instance, Issue 1.00, where 1 is the Issue and 00 is the Amendment. The next amendment change would appear as 1.01.

Upon receipt, amendment pages are to be inserted in this document and superseded pages removed. For each amendment incorporated, the amendment number, date of incorporation and the signature of the amending officer must be entered in the table below.

Amendment Number	Date Incorporated	Entered By

Commercial-in-Confidence

List of Effective Pages

Page Number	Issue Status	Page Number	Issue Status
Title Page	1.00		
ii	1.00		
iii	1.00		
iv	1.00		
1	1.00		
2	1.00		
3	1.00		
4	1.00		
5	1.00		
6	1.00		
7	1.00		
8	1.00		
9	1.00		
10	1.00		
11	1.00		
12	1.00		
13	1.00		
14	1.00		
15	1.00		
16	1.00		
17	1.00		
18	1.00		
19	1.00		
20	1.00		
21	1.00		

Commercial-in-Confidence

CONTENTS

1. Introduction 1
1.1 Purpose 1
1.2 Scope 1
1.3 Definitions, Acronyms and Abbreviations 1
1.4 References 2
2. GS Output Format 3
2.1 Overview 3
2.2 Output Information 5
2.2.1 Header 5
2.2.2 Original/Output Spheroid 7
2.2.3 Original/Output Grid 10
2.2.4 Area Limits 11
2.2.5 Run Header 13
2.2.6 Scan Header 14
2.2.7 Sounding Entry (S, P, N) 15
2.3 ASCII Format Legend 18
2.4 Binary Waveform Format 19
2.4.1 Header 19
2.4.2 Scan Header 20
2.4.3 Waveform 21
2.5 Binary Format Legend 22

Commercial-in-Confidence

1. INTRODUCTION

1.1 PURPOSE

The purpose of this document is to specify the format produced by the **Output Caris Data Function** of the Laser Airborne Depth Sounder (LADS) Mk II Ground System (GS).

1.2 SCOPE

The document applies to the results generated by the **Output Caris Data Function** of the LADS Mk II Ground System.

1.3 DEFINITIONS, ACRONYMS AND ABBREVIATIONS

Definitions

Mission	A mission is defined as a continuous period of operation of the LADS Mk II System, with the objective of conducting a survey of an area of ocean defined by the customer. Individual survey flights are called sorties.
Easting & Northing	The aircraft position is expressed in metres North and East of the false origin on the Universal Transverse Mercator (UTM) Grid. This implies that a change in easting and northing represents a corresponding movement on the earth's surface expressed in metres. Note: The changes in eastings and northings are related to changes in latitude and longitude via complex translation equations.
Julian Day	The numerical day of the year i.e. January 1 is day 1 and February 28 is day 59.
Soundings	Soundings consist of depth information that results from laser events and, position information corresponding to GPS data. The waveform as seen on the displays is a composite of the Green and IR returns. The soundings, numbered 1 to 48 for each scan, are always numbered from the starboard side.
Survey Run	This is the part of the survey objective where depth soundings are taken.
Fairchart	Hardcopy plot of bathymetric survey data. The soundings appearing on the fairchart are the sub-set of soundings that have the field "Fairchart Selected" set to "Y".
No Bottom At (NBA)	These are secondary soundings where the seabed has not been detected by the Ground System, and a NBA depth has been assigned by a Hydrographic Survey Operator. The depth value assigned is the depth which, in the opinion of the Hydrographic Survey Operator has been swept clear by laser, with depths less than this being detected by the system

Commercial-in-Confidence

Acronyms

AS	Airborne System
GS	Ground System
GPS	Global Positioning System
LADS	Laser Airborne Depth Sounder
UTC	Universal Time Coordinated
NBA	No Bottom At
UTM	Universal Transverse Mercator
NBD	No Bottom Detected

Abbreviations

Nil

1.4 REFERENCES

Glossary of LADS Terminology 0006A00005

Commercial-in-Confidence

2. GS OUTPUT FORMAT

2.1 OVERVIEW

When the **Output CARIS Data Function** is run the results take the form of two files per run:

1 An ASCII file with CAF(CARIS ASCII Format) extension,
2 A Binary file with CBF (CARIS Binary Format) extension.

Each file name has the following structure:

- a 1-12 character prefix (prompted for in the GS),
- followed by an underscore, and then
- 7-14 digits that describe in the following order; the run number (1-4 digits), an underscore, run segment (1-2 digits), an underscore, run sequence (1-2 digits), an underscore and run child (1-2 digits).

The maximum length of the filenames including the extension is 30 characters.

For example, if the operator enters a filename of OTWAY, then for run 1020.0.1.2, the two output files will be called:

"OTWAY_1020_0_1_2.CAF", and

"OTWAY_1020_0_1_2.CBF".

A GS tableau option allows for the CAF files to be kept together in a single file or spread over one file per run. In the case of the single file the filename will be of the form "OTWAY.CAF". Binary files will always be separate.

The structure of an ASCII file is shown in Figure 1-1 below, with the components being described in the following sections.

Commercial-in-Confidence

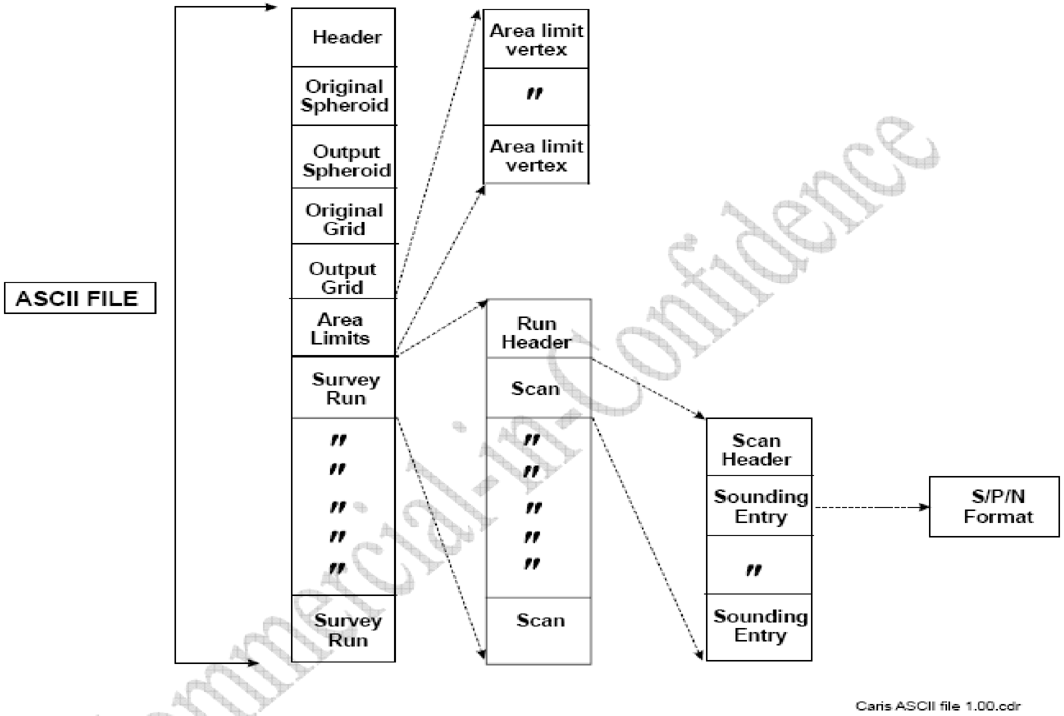

Figure 1-1 Structure of the ASCII file from the Output CARIS Data Function

Commercial-in-Confidence

2.2 OUTPUT INFORMATION

2.2.1 Header

The information associated with the **Header** output is listed below.

Name	Format	Range	Comments
Header identifier	X(3)	HCA	Header of CARIS ASCII format
Specification Issue	F(5,2)		Issue number of the specification
Mission title	X(40)		
Mission ident number	D(3)	1.. 999	
Time of data output	D(7)		Julian date, formatted as dddyyyy
Data scope	X(1)	S,P,A	S – All secondary soundings,<br>P – All Primary soundings.<br>A – All Primary soundings, including soundings where the sea bed was not found (NBD)
NBA included	X(1)	Y, N	This flag indicates that soundings marked as "No Bottom At" have been included.
Clash Range Radial	D(3)	0 .. 550	Represents the minimum distance in metres between soundings. Soundings contained in the fairchart are identified with the "fairchart selected" flag in the sounding data. If no reduction processing was performed this value will be 0, and the "fairchart selected" flag will be set true for all soundings.
Position transform applied	X(1)	Y, N	This flag indicates that positions used in this data have been transformed to a spheroid or grid different to that used to collect the data. The values for the original and output spheroid/grid are detailed below.

Table 1 Header Output Information

Commercial-in-Confidence

The format of the information associated with the **Header** output is as shown below:

Format
X(3) ^F(5,2) ^X(40) ^D(3) ^D(7) ^X(1) ^X(1) ^D(3) ^X(1)←

Table 2 Format of Header Information

Commercial-in-Confidence

2.2.2 Original/Output Spheroid

The information associated with the **Original/Output Spheroid** output is listed below.

Name	Format	Range	Comments
Spheroid entry identifier 1	X(2)	C1,D1	C – Original, D – Output.
Spheroid ident text	X(40)		
Spheroid entry identifier 2	X(2)	C2,D2	C – Original, D – Output.
Major semi axis	F(10,2)	6_300_000.0 .. 6_500_000.0	
Minor semi axis	F(10,2)	6_300_000.0 .. 6_500_000.0	
Flattening	F(9,6)	250.0 .. 350.0	
Eccentricity	F(14,12)	0.006 .. 0.0075	
Spheroid entry identifier 3	X(2)	C3,D3	C – Original, D – Output.
GPS X offset	F(8,2)	-1000.0 .. 1000.0	The following "GPS" prefixed fields represent the transformation parameters required to move from the WGS84 spheroid to this spheroid.
GPS Y offset	F(8,2)	-1000.0 .. 1000.0	
GPS Z offset	F(8,2)	-1000.0 .. 1000.0	
GPS X rotation	F(10,5)	-206.0 .. 206.0	Uses the Coordinate Axis Rotation sign convention. (ie. rotations effect the axis). A positive rotation is defined as clockwise when viewed from the origin along the axis. (eg for a given position, a positive rotation about the Z axis will result in the transformed position having a longitude with a smaller value)
GPS Y rotation	F(10,5)	-206.0 .. 206.0	see above

Commercial-in-Confidence

Name	Format	Range	Comments
GPS Z rotation	F(10,5)	-206.0 .. 206.0	see above
GPS Scale factor	F(8,5)	-1.0 .. 1.0	

Table 3 Output Information for Original/Output Spheroid

Commercial-in-Confidence

The format of the information associated with the **Original/Output Spheroid** output is as shown below:

**Format**
X(2) ∧X(40) ←
X(2) ∧F(10,2) ∧F(10,2) ∧F(9,6) ∧F(14,12) ←
X(2)∧F(8,2) ∧F(8,2) ∧F(8,2) ∧ F(10,5) ∧ F(10,5) ∧ F(10,5) ∧F(8,5) ←

Table 4 Format of Original/Output Spheroid Information

Commercial-in-Confidence

2.2.3 *Original/Output Grid*

The information associated with the **Original/Output Grid** output is listed below.

Name	Format	Range	Comments
Grid Entry Identifier	X(2)	F1,G1	F – Original, G – output
Grid ident text	X(20)		
Latitude of true origin	D(3)	-90 .. 90	
Central meridian longitude	D(4)	-180 .. 180	
Zone identifier	X(2)	1 .. 60, SP	1 .. 60 – UTM Zone identifier.<br>SP – identifies a non-standard (special) zone.
False origin easting	D(8)	-5_000_000 .. 5_000_000	
False origin northing	D(9)	-10_000_000 .. 20_000_000	
Central scale factor	F(7,4)	0.5 .. 1.5	

Table 5 Output Information for Original/Output Grid

The format of the information associated with the **Original/Output Grid** output is as shown below:

Format
X(2) ^X(20) ^D(3) ^D(4) ^X(2) ^D(8) ^D(9) ^F(7,4) ←

Table 6 Format of Original/Output Grid Information

Commercial-in-Confidence

2.2.4 Area Limits

The information associated with the **Area Limits** output is listed below.
There can be up to 10 vertices defining and area, with these vertices being numbered from 0-9. Lines are only output for vertices that have been defined. i.e. for a rectangle only 4 vertices are output.

The polygon points are ordered in a clockwise fashion, and the polygon is not closed geometrically. The coordinates are relative to the output spheroid and grid systems.

Name	Format	Range	Comments
Area limits identifier 1	X(2)	L0	Entry identifier for polygon point 1
Lat	F(12,8)	-90.0 .. 90.0	
Long	F(13,8)	-180.0 .. 180.0	
Easting	D(8)	-5_000_000 .. 5_000_000	
Northing	D(9)	-10_000_000 .. 20_000_000	
...			
Area limits identifier **n**	X(2)	L[**n**-1]	Entry identifier for polygon point **n**
Lat	F(12,8)	-90.0 .. 90.0	
Long	F(13,8)	-180.0 .. 180.0	
Easting	D(8)	-5_000_000 .. 5_000_000	
Northing	D(9)	-10_000_000 .. 20_000_000	
Where **n** = 1 .. 10			

Table 7 Output Information for Area Limits

Commercial-in-Confidence

The format of the information associated with the **Area Limits** output is as shown below:

Format
X(2) ^F(12,8) ^F(13,8) ^D(8) ^ D(9) ←

Table 8 Format of Area Limits Output

Commercial-in-Confidence

2.2.5 Run Header

The information associated with the **Run Header** output is listed below.

Name	Format	Range	Comment
Run header identifier	X(2)	R1	
Run identifier	X(14)		The run identifier has a format as follows:<br>Run.section.sequence.child (eg. 100.0.1.1)<br>Where<br>Run = run number<br>Section = section number of main run. Used when a section of the run is reflown.<br>Sequence = identifies the nth flown occurrence of the same run<br>Child = portion of run accepted manually by hydrographic selection
Date flown	D(7)		Julian Date, formatted as dddyyyy
Planned Track	D(3)	0 .. 360	The planned track of the run in degrees, expressed as a grid bearing
Status	X(9)	ACCEPTED, ANOMALOUS or REJECTED	The run status of the child run.

Table 9 Output Information for Run Header

The format of the information associated with the **Run Header** output is as shown below.

Format
X(2) ∧X(14) ∧D(7)∧D(3)∧X(9)←

Table 10 Format of Run Header Output

Commercial-in-Confidence

2.2.6 Scan Header

The information associated with the **Scan Header** output is listed below.

Name	Format	Range	Comment
Scan header identifier	X(2)	W1	
Scan Reference Position lat - output spheroid	F(12,8)	-90.0 .. 90.0	Corresponds to position of sounding at column 24 in the Output Spheroid. Expressed in degrees.
Scan Reference Position long - output spheroid	F(13,8)	-180.0 .. 180.0	Corresponds to position of sounding at column 24 in the Output Spheroid. Expressed in degrees.
Time - year	D(4)	0 .. 9999	
Time – Julian Day	D(3)	1 .. 366	
Time – Hour	D(2)	0 .. 23	
Time – Minute	D(2)	0 .. 59	
Time – Second	D(2)	0 .. 59	
Scan Row Number	D(2)	1 .. 18	The Scan Number can be considered as a time component, (1/18th) of a second
Tide Correction	F(6,2)	-20.00 .. 20.00	Represents the tide adjustment made to the observed depth to give the sounding Depth relative to the LAT datum.

Table 11 Output Information for Scan Header

The format of the information associated with the **Scan Header** output is as shown below.

Format
X(2) ^F(12,8) ^F(13,8) ^D(4) ^D(3) ^D(2) ^D(2) ^D(2) ^D(2) ^F(6,2) ←

Table 12 Format of Scan Header Output

Commercial-in-Confidence

2.2.7 Sounding Entry (S, P, N)

The information associated with the **Sounding Entry** output is listed below.

Name	Format	Range	Comments
Sounding identifier	X(1)	S,P,N,X	S - secondary sounding,<br>P - primary sounding,<br>N - NBA sounding,<br>X – NBD sounding.
Selected Depth Position lat - output spheroid	F(12,8)	-90.0 .. 90.0	Expressed in degrees.
Selected Depth Position long - output spheroid	F(13,8)	-180.0 .. 180.0	Expressed in degrees.
Selected Depth Position Easting - output spheroid	D(8)	-5_000_000 .. 5_000_000	
Selected Depth Position Northing - output spheroid	D(9)	-10_000_000 .. 20_000_000	
Contender Depth Position lat - output spheroid	F(12,8)	-90.0 .. 90.0	Expressed in degrees.<br>0.0 when no contender exists.
Contender Depth Position long - output spheroid	F(13,8)	-180.0 .. 180.0	Expressed in degrees.<br>0.0 when no contender exists.
Contender Depth Position Easting	D(8)	-5_000_000 .. 5_000_000	0 when no contender exists.

Commercial-in-Confidence

Name	Format	Range	Comments
- output spheroid			
Contender Depth Position Northing<br>- output spheroid	D(9)	-10_000_000 .. 20_000_000	0 when no contender exists.
Frame	D(4)	1..1749	Frame number
Row	D(2)	1..18	Scan number
Column	D(2)	1..48	Sounding number
Selected Depth	F(6,2)	-99.99 .. 99.99	Selected Depth to tide datum (includes tide correction) in metres<br>99.99 when no depth was detected
Contender Depth	F(6,2)	-99.99 .. 99.99	Contender Depth to tide datum (includes tide correction) in metres<br>99.99 when no depth was detected
Flag	D(1)	0..255	Validation flag from LADS Ground System (see Table 15 – Validation Flag bit values)
Comment	X(10)		Operator comment
Spare	X(10)		Spare field for future expansion

Table 13 Output Information for Sounding Entry

Commercial-in-Confidence

The format of the information associated with the **Sounding Entry** output is as shown below.

**Format**
X(1) ^F(12,8) ^F(13,8) ^D(8) ^D(9) ^ F(12,8) ^F(13,8) ^D(8) ^D(9) ^D(4) ^D(2) ^D(2) ^F(6,2) ^F(6,2) ^D(1) ^X(10) ^X(10)←

Table 14 Format of Sounding Entry Output

Bit 7	Bit 6	Bit 5	Bit 4	Bit 3	Bit 2	Bit 1	Bit 0
spare	Clashed	Converted NBD	Manual Secondary	Swapped Contenders	Significant Contender	Excessive Gradient	Depth Edited

Table 15 – Validation Flag bit values

Commercial-in-Confidence

2.3 ASCII FORMAT LEGEND

The legend for the symbols used in the format tables is listed below.

Symbol	Description	Comments
X	Text	
D(max_size)	Integer	Max_size represents the maximum number of characters allowed for the integer, including a leading minus sign if appropriate.
F(max_size,aft_digits)	Float	Max_size represents the maximum number of characters allowed for the float, including a leading minus sign if appropriate.<br>Aft_digits represents the number of digits after the decimal point.<br>Eg " -10.000" would be represented as F(7,3)
^	Field separator	May be comma, space, tab.
←	Line terminator	(may be <CR><LF>, <LF>, <CR>)

Table 16 ASCII Format Legend

Commercial-in-Confidence

2.4 BINARY WAVEFORM FORMAT

Raw waveform data is provided in a binary file in the following format.

2.4.1 Header

The information associated with the **Binary File Header** is listed below.

Name	Format	Minimum value	Maximum Value	Comments
Header identifier	ASCII(3)	HCB		Header identifier for Caris Binary file
Specification Issue major issue number	uchar	0	255	1
Specification Issue minor issue number	uchar	0	255	0
Mission Title	ASCII(40)	0	255	A string of 40 ASCII characters as per 0 Header. String is space padded.
Run Identifier	ushort	1	9 999	LADS run number range 1..9 999
Run Segment	uchar	0	99	LADS run segment number range 0..99
Run Sequence	uchar	0	99	LADS run sequence number range 0..99
Run Child	uchar	0	99	LADS run child number range 0..99

Table 17 Binary File Header

Commercial-in-Confidence

2.4.2 Scan Header

The information associated with the **Binary Scan Header** is listed below.

Name	Format	Minimum value	Maximum Value	Comments
Scan header identifier	ASCII(2)	W1		
Time - year	ushort	0	9999	
Time – Julian Day	ushort	1	366	
Time – Hour	uchar	0	23	
Time – Minute	uchar	0	59	
Time – Second	uchar	0	59	

Table 18 Binary Scan Header

Commercial-in-Confidence

2.4.3 Waveform

The information associated with the **Waveform** is listed below.

Name	Format	Minimum value	Maximum Value	Comments
Waveform identifier	ASCII(2)	WF		Waveform identifier
Frame	ushort	1	1 749	LADS frame number range 1..1 749
Row	uchar	1	18	LADS scan number range 1..18
Column	uchar	1	48	LADS sounding number range 1..48
Selected Depth Index	uchar	0	255	Index into the waveform indicating position of the selected depth
Contend Depth Index	uchar	0	255	Index into the waveform indicating position of the contending depth. 0 indicates no contender
Waveform Sample 1	uchar	0	255	1st sample of the digital waveform
Waveform Sample 2	uchar	0	255	2nd sample of the digital waveform
.<br>.<br>.				
Waveform Sample 120	uchar	0	255	120th sample of the digital waveform

Table 19 Binary Waveform

Commercial-in-Confidence

2.5 BINARY FORMAT LEGEND

The legend for additional symbols used in the format tables is shown below.

Symbol	Description	Minimum value	Maximum Value	Binary Size (bytes)
ASCII	ASCII character	0	255	1
ushort	Unsigned 16 bit Integer	0	65 535	2
uchar	Unsigned 8 bit Integer	0	255	1

Table 20– Binary Format Legend

Commercial-in-Confidence

Enclosure 3: Specifications for LADS MkII, XYZ ASCII Data Export Format

SPECIFICATION - LADS XYZ DATA EXPORT FORMAT

This is a controlled document.

Copy No:

Issue No: 1.00

Commercial-in-Confidence

LADS Mark II

Specification for the Ground System
XYZ Export Format

Document Number: LADS2A05.001.013

Authorised by: M. Penley

Date: 19 JAN 05

This document contains information which is the property of Tenix LADS Corporation and may not be reproduced, copied or disclosed in any form to a third party without the written permission of Tenix LADS Corporation.

Amendment Status

As necessary, authorised amendments will be issued to holders of this document. Amendments will take the form of replacement or additional pages.

The amendment status appears at the top right side of each page. For instance, Issue 1.00, where 1 is the Issue and 00 is the Amendment. The next amendment change would appear as 1.01.

Upon receipt, amendment pages are to be inserted in this document and superseded pages removed. For each amendment incorporated, the amendment number, date of incorporation and the signature of the amending officer must be entered in the table below.

Amendment Number	Date Incorporated	Entered By

Commercial-in-Confidence

List of Effective Pages

Page Number	Issue Status	Page Number	Issue Status
Title Page	1.00		
ii	1.00		
iii	1.00		
iv	1.00		
1	1.00		
2	1.00		
3	1.00		
4	1.00		
5	1.00		
6	1.00		
7	1.00		
8	1.00		

Commercial-in-Confidence

CONTENTS

1. Introduction 1
1.1 Purpose 1
1.2 Scope 1
1.3 Definitions, Acronyms and Abbreviations 1
1.3.1 Definitions 1
1.3.2 Acronyms 2
1.3.3 Abbreviations 2
1.4 References 2
2. Format Description 3
2.1 Overview 3
2.2 File Layout 3
2.2.1 File Contents 3
2.2.2 File Naming 3
2.3 Sounding Data File Format 4
2.3.1 Contents 4
2.3.2 Format 5
2.3.3 Line Format 6
3. Format Legend 8

Commercial-in-Confidence

1. INTRODUCTION

1.1 PURPOSE

The purpose of this document is to specify the format of the deliverable files of the XYZ export format, as produced by the LADS Mk II Ground System.

1.2 SCOPE

This document applies only to the description of the XYZ format.

1.3 DEFINITIONS, ACRONYMS AND ABBREVIATIONS

Definitions

Easting & Northing	The aircraft position is expressed in metres North and East of the false origin on the Universal Transverse Mercator (UTM) Grid. This implies that a change in easting and northing represents a corresponding movement on the earth's surface expressed in metres. Note: Changes in easting and northing are related to changes in latitude and longitude via complex translation equations.
Sounding	A Sounding is the result of a single fire of the laser, and represents the depth at a particular geographic position as measured by the LADS Mk II system.
Run	A single continuous pass of data collection by the LADS aircraft.
Fairsheet	Hardcopy plot of bathymetric survey data.
Julian Day	The numerical day of the year i.e. January 1 is day 1 and February 28 is day 59
Secondary/Primary Soundings	Secondary soundings have been accepted by a hydrographer, whereas Primary soundings have been rejected.
No Bottom At (NBA)	These are secondary soundings where the seabed has not been detected by the Ground System, and a NBA depth has been assigned by a Hydrographic Survey Operator. This depth value assigned is the depth which, in the opinion of the HSO has been swept clear by laser, with depths less than this being detected by the system

Commercial-in-Confidence

Acronyms

AS	Airborne System
CR	Carriage Return character
GPS	Global Positioning System
GS	Ground System
LADS	Laser Airborne Depth Sounder
LAT	Lowest Astronomical Tide
LF	Line feed character
NBA	No Bottom At
TMC	Transverse Mercator Coordinate
UTC	Universal Time Co-ordinated
UTM	Universal Transverse Mercator

Abbreviations

Nil

1.4 REFERENCES

Nil

Commercial-in-Confidence

2. FORMAT DESCRIPTION

2.1 OVERVIEW

The XYZ Export Format is a minimized format for the export of position and depth information from the LADS MkII Ground System. Positions are exported in TMC format (easting and northing), Geographic format (latitude and longitude), or both formats. Additional information such as tide, LADS sounding index, and clash reduction status is optionally exported in conjunction with position and depth.

2.2 FILE LAYOUT

2.2.1 File Contents

Each XYZ export set consists of a directory containing 2 files in ASCII format:

1. Sounding data file
2. Parameter/Option file

The Sounding data file contains the data in XYZ format. The contents of this file are described in the following sections.

The Parameters file contains the parameters and options that were used to generate the XYZ data. It is written in a free form text format, and contains the following information:

- Area Boundaries
- Export Options
- Sounding Reduction (Clash) Options
- Sounding Attribute Filter Options, used to determine the soundings that were exported
- Spheroid and Grid Information of the original and exported data
- LADS database from which the data was exported
- Time of data export
- Number of soundings exported

2.2.2 File Naming

The XYZ export directory has a name consisting of 12 standard ASCII characters.
The Sounding data file is named identically to the enclosing directory, but with the extension ".XYZ".
The Parameters file is named "PARAMS.DAT"

Commercial-in-Confidence

2.3 SOUNDING DATA FILE FORMAT

2.3.1 Contents

Each line in the file corresponds to one depth sounding, and consists of the data fields in the following table. A number of parameters determine the presence of some fields, the depth format and the field separator and terminator characters. These parameters are set within the GS upon the export of the sounding data, and are described in the following table.

Parameter	Effect
Position_Format	Determines whether sounding coordinates are represented in TMC format, Geographic (Lat/Long) format, or both
Depth Format	Determines whether drying soundings are signed positive or negative
Additional_Fields	Determines whether additional sounding information beyond XYZ information is required
Field Separator	Determines the character to use between fields
Line Terminator	Determines the character to use at the end of a line (i.e. between soundings)

Where applicable, the effect of the parameter on the presence of a particular field is indicated in the "When Present" column in the table below. The values of the parameters are featured in the in the Parameters file that is included with each data set. These parameters will be set according to the requirements of the customer or software application to which the data is to be delivered/used.

Commercial-in-Confidence

2.3.2 *Format*

Refer to the table at the end of the document for an explanation of the format descriptors.

Field Name	Format	Range	Description/Comments	When Present
TMC Easting	F(10,1)	-5_000_000.0 .. 5_000_000.0	TMC easting of the sounding, relative to the export Spheroid and Grid parameters.	Position_Format = '*TMC*' or '*BOTH*'
TMC Northing	F(11,1)	- 10_000_000.0 .. 20_000_000.0	TMC northing of the sounding, relative to the export Spheroid and Grid parameters.	Position_Format = '*TMC*' or '*BOTH*'
Latitude	F(12,8)	-90.0 .. 90.0	Latitude of the sounding, relative to the export Spheroid parameters. Expressed in degrees to 8 decimal places.	Position_Format = '*LAT_LONG*' or '*BOTH*'
Longitude	F(12,8)	-180.0 .. 180.0	Longitude of the sounding, relative to the export Spheroid parameters. Expressed in degrees to 8 decimal places.	Position_Format = '*LAT_LONG*' or '*BOTH*'
Depth	F(5,2)	-99.0 .. 99.9	Depth relative to the LAT datum. When the "Depths Positive" parameter is true, depths are positive and drying soundings are negative. When "Depths Positive" is false, the reverse case applies. Refer to the Parameters file for the value of this parameter.	
Year	I(4)	0 .. 9999	Year of sounding collection	Additional_Fields = '*TRUE*'
Julian day	I(3)	0 .. 366	Julian day of sounding collection	Additional_Fields = '*TRUE*'
Time	X(8)		Time of sounding collection. In format HH:MM:SS.	Additional_Fields = '*TRUE*'
Run Identifier	X(14)		LADS Run Identifier in which sounding was collected.	Additional_Fields = '*TRUE*'
Frame	I(4)	0 .. 1749	LADS Frame Identifier in which sounding was collected.	Additional_Fields = '*TRUE*'
Row	I(2)	1 .. 18	LADS Scan Identifier in which sounding was collected.	Additional_Fields = '*TRUE*'
Column	I(2)	1 .. 48	LADS Sounding Identifier in which sounding was collected.	Additional_Fields = '*TRUE*'
Tidal Correction	F(5,2)	-20.0 .. 40.0	Tide correction applied to the sounding. Tides are relative to the LAT datum. Tide values are subtracted from the observed depth value.	Additional_Fields = '*TRUE*'
Clash Flag	X(1)	'Y' or 'N'	'Y' – the sounding is clashed in, and so is included in the reduced dataset.<br>'N' – the sounding is clashed out, and so is excluded from the reduced dataset.	Additional_Fields = '*TRUE*'

Commercial-in-Confidence

2.3.3 Line Format

The format of a line is given below for each combination of the Position Format and Additional Fields parameters.

Position Format of TMC, No Additional Fields

FORMAT
F(10,1)^F(11,1)^F(5,2)←

Position Format of TMC, With Additional Fields

FORMAT
F(10,1)^F(11,1)^F(5,2)^I(4)^I(3)^X(8)^X(14)^I(4)^I(2)^I(2)^F(5,2)^X(1)←

Position Format of Lat/Long, No Additional Fields

FORMAT
F(12,8)^F(12,8)^F(5,2)←

Position Format of Lat/Long, With Additional Fields

FORMAT
F(12,8)^F(12,8)^F(5,2)^I(4)^I(3)^X(8)^X(14)^I(4)^I(2)^I(2)^F(5,2)^X(1)←

Position Format of TMC and Lat/Long, No Additional Fields

FORMAT
F(10,1)^F(11,1)^F(12,8)^F(12,8)^F(5,2)←

9

Commercial-in-Confidence

Position Format of TMC and Lat/Long, With Additional Fields

FORMAT
F(10,1)^F(11,1)^F(12,8)^F(12,8)^F(5,2)^I(4)^I(3)^X(8)^X(14)^I(4)^I(2)^I(2)^F(5,2)^X(1)←

Commercial-in-Confidence

3. FORMAT LEGEND

The legend for the symbols used in the format tables is shown below.

Symbol	Type	Comments
X(max)	Alphanumeric	"Max" represents the maximum characters in the field
D	Date	Dates are stored as an 8 digit integer, in the format:<br>CCYYMMDD<br>Where CC = century<br>YY = year<br>MM = month number<br>DD = day of month
I(max)	Integer	"Max" represents the maximum number of digits allowed for the integer, including a leading minus sign if appropriate. Eg. The smallest number in a field with format I(4) would be "-999"
F(fore_digits,aft_digits)	Float	fore_digits represents the maximum number of digits before the decimal point, including a leading minus sign if required.<br>Aft_digits represents the number of digits after the decimal point.<br>Eg " -10.000" would be represented as F(3,3)
^	Field separator	A space character (" ") or comma (",") is used to separate fields (Refer to 2.3.1 for when each is used)
←	Line terminator	A <CR> (carriage return) character, <LF> (linefeed) character, or <CR><LF> (carriage return, linefeed) is used to terminate lines. (Refer to 2.3.1 for when each is used)

Format Legend

Commercial-in-Confidence

Annex C. Geodetic Records

All collected data was post-processed relative to the European Terrestrial Reference System (ETRS89) during the application of the Kinematic GPS solution. All coordinates refer to the Universal Transverse Mercator projection, Northern Hemisphere (UTM N) in Zone 29 Central Meridian 9° West.

C.1 Geodetic Parameters

The following are the parameters for the Geodetic Datum used for data delivery.

**Spheroid**	Name	GRS80
	Semi Major Axis	6 378 137.0
	Semi Minor Axis	6 356 752.3141
	Compression	298.257222101
**Grid System**	Projection Type	Universal Transverse Mercator
	Zone	29 Northern Hemisphere
	Longitude of Central Meridian	9° West
	Scale Factor at Central Meridian	0.9996000
	Longitude of Grid Origin	000°00'00.00"E
	Latitude of Grid Origin	000°00'00.00"N
	False Easting	500 000.000 m
	False Northing	0 m

Table 1 – Geodetic Datum

C.2 Geodetic Control Stations

C.2.1 Fugro OmniStar Virtual Base Station Service

Throughout the survey, the real-time position of the LADS Mk II system was derived from an Ashtech GG24 GPS receiver. WADGPS corrections from the Fugro OmniStar Virtual Base Station (VBS) service were received using an OmniStar 3510LR system and applied to the raw GPS position as received by the Ashtech GG24 GPS receiver.

C.2.2 LADS Local GPS Base Station – Radisson SAS Hotel, Galway

A local GPS base station was established by LADS surveyors on 19 May at the Radisson SAS Hotel in Galway. This station was coordinated using static GPS methods. A check of the base station coordination was performed by Arrigan Geo-Surveyors using rapid-static GPS methods. The static GPS data was processed using Waypoint GrafNet software and constraining the station using IGS continually operating GPS stations nearby. Refer to enclosure 2 for the processing and adjustment report.

The derived ETRS89 coordinates for the local GPS base station are:

Latitude	Longitude	Easting	Northing	Ellipsoidal Height
53° 16' 26.58445" N	9° 02' 37.52701" E	497 082.046	5 902 758.068	85.180m

Table 2 – GPS local base station coordinates

The Ellipsoidal Height referred to in table 2 is given to the L1 phase centre of the GPS Antenna. This height is different to the height given in the LADS GPS Station Summary which refers the height of the top of the permanent pole, supporting a Microwave Transceiver, on to which the GPS antenna was attached. The LADS Station Summary is given in enclosure 1.

C.2.3 Static Position Check Control Marks – Galway International Airport

Arrigan Geo-Surveyors undertook the coordination of the static check marks at Galway International Airport using a combination of rapid static GPS and terrestrial methods on the 19 May 2008. The report of survey from Arrigan Geo-Surveyors is enclosed in Enclosure 2. Table 3 gives the coordinates of the static marks. Enclosure 3 shows the layout of the static position check marks and the laser source mark.

Mark	Easting	Northing
ST1	503 912.615	5 905 825.400
ST2	503 918.165	5 905 839.674
ST3	503 903.379	5 905 836.726

Table 3 – Static Position Check Coordinates

C.2.4 Derived Antenna Position for Static Position Check

For the static position check the aircraft was parked in the centre of the three control points. The position of the laser source was then plumbed down from the aircraft to the tarmac and marked with a nail. This position, the laser source mark, was derived by measuring the distances between it and the static position check marks and elementary trigonometry was used to derive the coordinate. To derive the antenna position the aircraft heading was determined using the AHRS Gyro Compass Alignment routine, this was found to be 165º. The fixed values of the Antenna to Laser Source Offset were applied to derive the coordinates of the aircraft GPS antenna. The derived ETRS89 coordinates for the laser source position during the static position check performed on 21 May 2008 were:

Easting	503 909.911
Northing	5 905 834.111

Table 4 – Laser source position coordinates

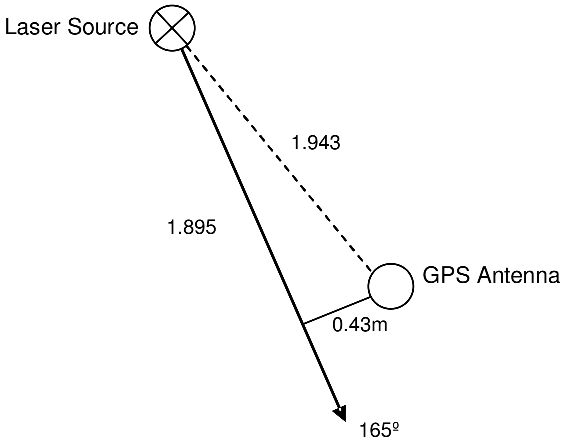

$\Delta E = 1.943.\sin(165-12.7847)° = 0.906m$

$\Delta N = 1.943.\cos(165-12.7847)° = -1.719m$

The derived ETRS89 coordinates for the GPS antenna position during the static position check performed on 21 May 2008 were:

Easting	503 910.817
Northing	5 905 832.392

Table 5 –GPS antenna coordinates

Enclosures:

1. LADS GPS Station Summary
2. GrafNet Processing Report and Network Adjustment Report

3. Static Position Check Triangle Diagram

4. Arrigans Coordination Report

Enclosure 1 LADS GPS Base Station Summary

LADS STATION SUMMARY

Station Name or Number<br>**GAL1**			Order
Location<br>**Radisson SAS Hotel, Galway, Ireland**			Positional Uncertainty
Country<br>**Ireland**	State<br>**County Galway**	District<br>**Galway**	
HORIZONTAL DATUM:	☐ AGD 66 ☐ AGD 84	☐ WGS 72 ☐ GDA 94	☐ WGS 84 ☒ Other (specify) ☐ ITRF 2000 **ETRF89**
VERTICAL DATUM:	☐ Australian Height Datum ☐ Sounding Datum	☐ Other (specify)	Reduced Level (metres)<br>**N / A**
ELLIPSOIDAL HEIGHT	☒ GRS80	☐ Other (specify)	Height (metres)<br>**84.150*** **
RECTANGULAR CO-ORDINATES	UTM / ZONE<br>**North / Zone 29**		Central Meridian<br>**9°W**
Latitude<br>**53° 16‘ 26.58445“S**	Longitude<br>**9° 2‘ 37.52701“E**		Convergence<br>**0° 2‘ 6.26“**
Easting<br>**497082.046mE**	Northing<br>**5902758.068mN**		N Measured
Station Established by<br>**BCM**	Establishing Survey Reference<br>**08_9nw_ireland**		Date<br>**19 May 2008**

Revisited Date				
Report Reference				
Revisiting Authority				
Reference Books		Probable Life of Mark<br>**5 years**		

Satellite Obstructions Polar Plot (If no masking, "Intentionally Left Blank")

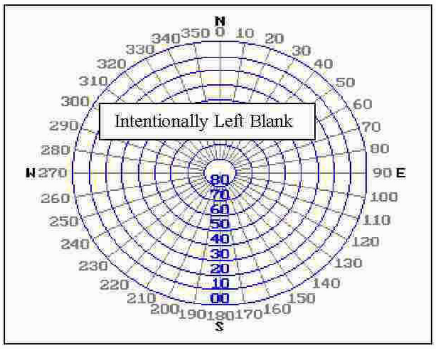

Location: Fixed on top of a permanent steel pole supporting a Microwave Transceiver. The pole is on the north western wing of the hotel on the roof of level 4, directly above room 474. The GPS data logger was located on level 4 in room 473.

Access: Access will need to be arranged through the hotel Engineers. Entrance to the roof is made via the 5th floor which is off limits to all but the executive guests to the hotel, hence an escort is required. The service lifts can be used to access the 5th floor by arrangement.

Description: No actual mark exists; the coordinated position is the logging phase centre of an AeroAntenna Technology
AT2775_42 L1/L2 GPS antenna.
*** The height refers to the top of the steel pole. See profile diagram over page.

Visibility: There are no obstructions to satellite visibility at the location of the antenna.

LS 5.01 Version 1 dated 14/11/06

PARTICULARS OF STATION MARK PART ONE

PLAN

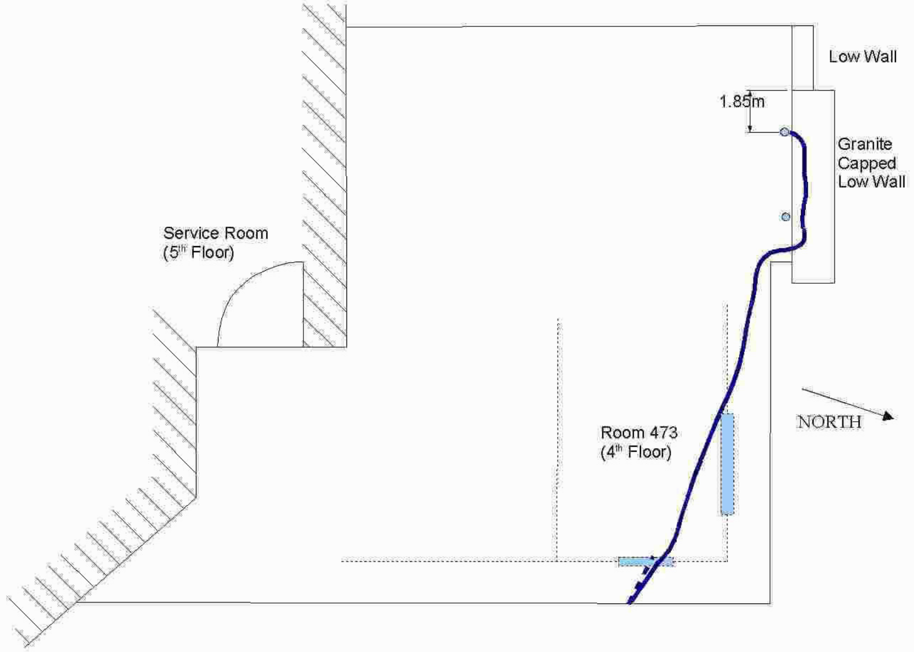

PROFILE:

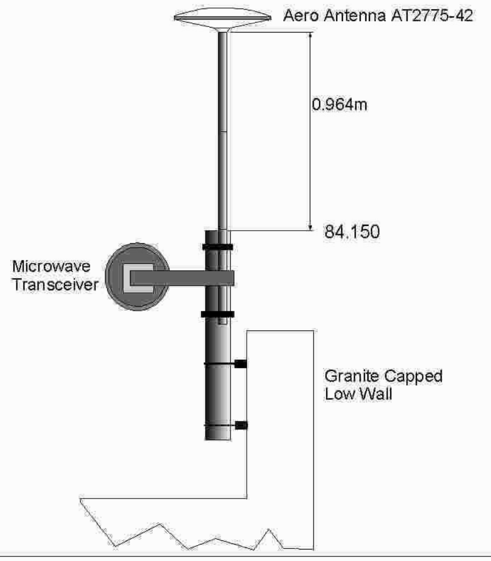

LS 5.01

Version 1 dated 14/11/06

PARTICULARS OF STATION MARK PART TWO
(To include View of Mark, View of Area and View of Chartlet).

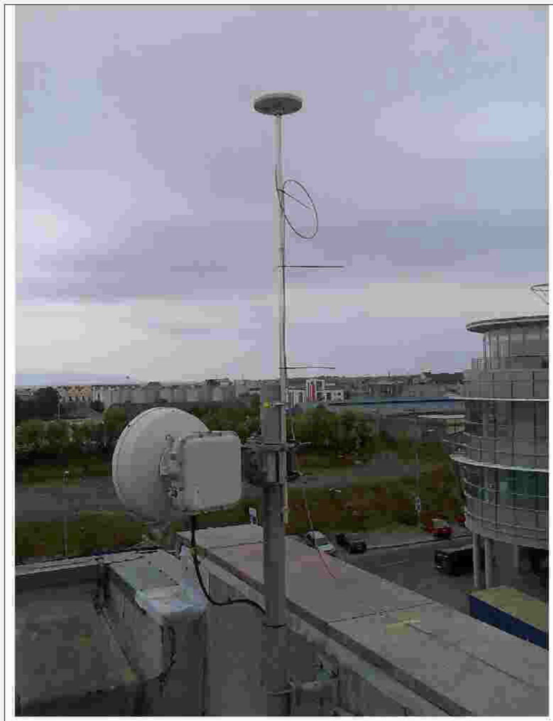

Compiled by: Tyson Hillyard	Date: 22 May 08
Checked by: Ben McWilliam	Date: 23 May 08

LS 5.01 Version 1 dated 14/11/06

Enclosure 2 GrafNet Processing Report and Network Adjustment Report

```
************************************************
* GrafNet - GRAPHIC GPS NETWORK PROCESSING     *
*           SOFTWARE PACKAGE                   *
*                                              *
* PROJECT REPORT                               *
*                                              *
* Copyright NovAtel Inc. (2006)                *
*                                              *
* Version: 7.60.2209                           *
*                                              *
* PROJECT: OSI.gnt                             *
************************************************
```

DATE: 5/22/2008 (m/d/y)

TIME: 10:33:10

```
**********************************************************************
      PROJECT INFORMATION
**********************************************************************
```

Datum: ETRS89

Grid: Irish Grid

Path: C:\Documents and Settings\tysonh\My Documents\DATABASES\08_9nw_ireland\Geodetics\Base Station Coordination\16HR\

```
**********************************************************************
      OBSERVATION FILES
**********************************************************************
```

Obs	File	ID	AntHgt	StartDate	StrtTime	Length	Int(s)	Antenna Info
1	.\ATHL.gpb	Athlone	0.110	05/20/2008	18:00:00	17:59:55	5.000	AOAD/M_T, MeasDist 0.000 m to mark/ARP
2	.\CSTB.gpb	CastleBa	0.110	05/20/2008	18:00:00	17:59:55	5.000	AOAD/M_T, MeasDist 0.000 m to mark/ARP
3	.\B16HRA08.gpb	GAL1	0.000	05/20/2008	19:01:15	16:15:00	15.000	Generic, MeasDist 0.000 m to PC
4	.\GLWY.gpb	Galway	0.110	05/20/2008	18:00:00	17:59:55	5.000	AOAD/M_T, MeasDist 0.000 m to mark/ARP
5	.\LMRK.gpb	Limerick	0.110	05/20/2008	18:00:00	17:59:55	5.000	AOAD/M_T, MeasDist 0.000 m to mark/ARP

****

CONTROL/CHECK POINTS

****

ID	Type	Latitude	Longitude	Grid-E(m)	Grid-N(m)	Ell-Hgt	OrthoHgt	SD-H(mm)	SD-Z(mm)
Athlone	3-D GCP	53 25 55.81991	-7 54 27.46160	206139.697	242454.814	114.082	-	5.00	5.00
CastleBa	3-D GCP	53 51 02.65602	-9 17 14.31657	115281.144	289806.666	111.150	-	5.00	5.00
Galway	3-D GCP	53 16 50.45125	-9 03 38.65035	129246.483	226115.343	86.506	-	5.00	5.00
Limerick	3-D GCP	52 40 26.35651	-8 34 32.51885	161057.427	158227.210	82.665	-	5.00	5.00

****

SESSION INFORMATION

****

Name	MObs	RObs	Length	Dist(km)	Status	Sol-Type	RMS(mm)	Rel.	SD(mm)
Athlone to GAL1 (1)	1	3	16:15:00	77.672	Good	L3-IonoFreeFloat	-	-	1.9
CastleBa to Athlone (1)	2	1	17:59:55	102.452	Good	L3-IonoFreeFloat	-	-	1.1
CastleBa to GAL1 (1)	2	3	16:15:00	66.182	Good	L3-IonoFreeFloat	-	-	1.8
Galway to Athlone (1)	4	1	17:59:55	78.607	Good	L3-IonoFreeFloat	-	-	1.0
Galway to CastleBa (1)	4	2	17:59:55	65.198	Good	L3-IonoFreeFloat	-	-	1.0
Galway to GAL1 (1)	4	3	16:15:00	1.352	Good	L1L2-NewFixed	6.3	-	0.5
Limerick to CastleBa (1)	5	2	17:59:55	139.302	Good	L3-IonoFreeFloat	-	-	1.3
Limerick to Athlone (1)	5	1	17:59:55	95.530	Good	L3-IonoFreeFloat	-	-	1.1
Limerick to GAL1 (1)	5	3	16:15:00	73.811	Good	L3-IonoFreeFloat	-	-	1.9
Limerick to Galway (1)	5	4	17:59:55	74.967	Good	L3-IonoFreeFloat	-	-	1.0

$-Reliabity or RMS failed statistics

```
             **************************************************
             * NETWORK - WEIGHTED GPS NETWORK ADJUSTMENT      *
             *                                                *
             * (c) Copyright NovAtel Inc., (2006)             *
             *                                                *
             * Version: 7.60.2209                             *
             *                                                *
             * FILE: C:\Documents and Settings\tysonh\My Documents\DATABASES\08_9nw_ireland\Geodetics\Base Station Coordination\16HR\OSI.net
             **************************************************

DATE(m/d/y): Thur.   5/22/08   TIME: 10:48:43

***************************************************************

  DATUM:            'ETRS89'
  GRID:             UTM, Zone 29
  SCALE_FACTOR:     14.9500
  CONFIDENCE LEVEL: 95.00 %   (Scale factor is 2.4479)

****************************************************************
    INPUT CONTROL/CHECK POINTS
****************************************************************

STA_ID     TYPE    -- LATITUDE --  -- LONGITUDE --  ELLHGT -   HZ-SD   V-SD
Athlone    GCP-3D  53 25 55.81991   -7 54 27.46160    114.082 0.00500 0.00500
CastleBa   GCP-3D  53 51 02.65602   -9 17 14.31657    111.151 0.00500 0.00500
Galway     GCP-3D  53 16 50.45125   -9 03 38.65035     86.506 0.00500 0.00500
Limerick   GCP-3D  52 40 26.35651   -8 34 32.51885     82.665 0.00500 0.00500
```

```
****************************************************************
    INPUT VECTORS
****************************************************************

SESSION NAME           VECTOR(m)   ------ Covariance (m) [unscaled] ------
                        DX/DY/DZ          standard deviations in brackets
Athlone to GAL1 (1)    2797.0385  1.0199e-006 (0.0010)
                     -76904.8461  2.6997e-008 1.4988e-006 (0.0012)
                     -10527.0963  5.9998e-007 -6.2140e-008 1.0192e-006 (0.0010)

CastleBa to Athlone (1) 50735.3067  3.5335e-007 (0.0006)
                      84614.4810  3.1998e-008 5.4310e-007 (0.0007)
                     -27615.7597  2.0991e-007 -9.9407e-009 3.5792e-007 (0.0006)

CastleBa to GAL1 (1)  53532.3440  9.3247e-007 (0.0010)
                       7709.6353  2.8474e-008 1.3564e-006 (0.0012)
                     -38142.8568  5.5049e-007 -6.7246e-008 9.4666e-007 (0.0010)

Galway to Athlone (1) -2035.5589  2.9320e-007 (0.0005)
                      77930.4138  2.7663e-008 4.5005e-007 (0.0007)
                      10084.8616  1.7302e-007 -6.1789e-009 2.9166e-007 (0.0005)

Galway to CastleBa (1) -52770.8658  2.6423e-007 (0.0005)
                      -6684.0673  2.5739e-008 4.0335e-007 (0.0006)
                      37700.6212  1.5579e-007 -9.3243e-009 2.6590e-007 (0.0005)

Galway to GAL1 (1)      761.4678  6.7921e-008 (0.0003)
                       1025.5697  -1.0461e-008 2.5279e-008 (0.0002)
                       -442.2423  4.7737e-008 -7.4254e-009 1.2680e-007 (0.0004)

Limerick to CastleBa (1) -111030.9422  4.8343e-007 (0.0007)
                     -30616.6246  4.5293e-008 7.4544e-007 (0.0009)
                      78357.6917  2.8458e-007 -1.5758e-008 4.8137e-007 (0.0007)

Limerick to Athlone (1) -60295.6354  3.3595e-007 (0.0006)
                      53997.8567  3.1678e-008 5.1863e-007 (0.0007)
                      50741.9319  1.9727e-007 -5.1997e-009 3.3006e-007 (0.0006)

Limerick to GAL1 (1)  -57498.5969  9.9295e-007 (0.0010)
                     -22906.9875  2.6065e-008 1.4602e-006 (0.0012)
                      40214.8367  5.8035e-007 -6.6268e-008 9.7925e-007 (0.0010)

Limerick to Galway (1) -58260.0764  2.8537e-007 (0.0005)
                     -23932.5574  2.6690e-008 4.3877e-007 (0.0007)
                      40657.0706  1.6713e-007 -8.0805e-009 2.8008e-007 (0.0005)
```

****

OUTPUT VECTOR RESIDUALS (East, North, Height - Local Level)

****

SESSION NAME	-- RE -- (m)	-- RN -- (m)	-- RH -- (m)	- PPM -	DIST (km)	- STD - (m)
Athlone to GAL1 (1)	0.0009	0.0037	-0.0100	0.138	77.7	0.0073
CastleBa to Athlone (1)	-0.0012	-0.0009	0.0039	0.040	102.5	0.0043
CastleBa to GAL1 (1)	-0.0006	0.0024	-0.0047	0.081	66.2	0.0070
Galway to Athlone (1)	-0.0007	0.0008	-0.0014	0.023	78.6	0.0039
Galway to CastleBa (1)	0.0006	0.0016	-0.0051	0.083	65.2	0.0037
Galway to GAL1 (1)	-0.0000	-0.0005	0.0018	1.389	1.4	0.0018
Limerick to CastleBa (1)	0.0016	0.0006	-0.0015	0.016	139.3	0.0051
Limerick to Athlone (1)	0.0001	-0.0002	0.0024	0.025	95.5	0.0042
Limerick to GAL1 (1)	-0.0009	0.0026	-0.0083	0.119	73.8	0.0072
Limerick to Galway (1)	0.0011	-0.0010	0.0035	0.051	75.0	0.0039
RMS	0.0009	0.0018	0.0051			

$ - This session is flagged as a 3-sigma outlier

****

CONTROL POINT RESIDUALS (ADJUSTMENT MADE)

****

STA. NAME	-- RE -- (m)	-- RN -- (m)	-- RH -- (m)
Athlone	0.0077	0.0042	-0.0242
CastleBa	-0.0109	-0.0163	0.0290
Galway	-0.0023	0.0103	-0.0134
Limerick	0.0059	0.0015	0.0086
RMS	0.0074	0.0099	0.0205

****

OUTPUT STATION COORDINATES (LAT/LONG/HT)

****

STA_ID	-- LATITUDE --	-- LONGITUDE --	- ELLHGT -
Athlone	53 25 55.82005	-7 54 27.46118	114.0578
CastleBa	53 51 02.65549	-9 17 14.31717	111.1795
GAL1	53 16 26.58445	-9 02 37.52701	85.1796
Galway	53 16 50.45158	-9 03 38.65047	86.4926
Limerick	52 40 26.35656	-8 34 32.51853	82.6736

```
*****************************************************************
    OUTPUT STATION COORDINATES (GRID)
*****************************************************************

STA_ID        - EASTING -   - NORTHING - - ELLHGT -
                   (m)            (m)         (m)
Athlone       572574.0944  5920903.5555   114.0578
CastleBa      481099.6564  5966953.1333   111.1795
GAL1          497082.0459  5902758.0683    85.1796
Galway        495950.4513  5903496.4362    86.4926
Limerick      528688.9089  5836089.8474    82.6736

*****************************************************************
    OUTPUT VARIANCE/COVARIANCE
*****************************************************************
                                                 2
STA_ID       SE/SN/SUP   --------- CX matrix (m )-----------
             (95.00 %)   (not scaled by confidence level)
                (m)           (ECEF, XYZ cartesian)
Athlone         0.0067   7.0366e-006
                0.0063   6.5108e-008 7.4429e-006
                0.0067   4.5157e-007 -2.0532e-008 7.0360e-006

CastleBa        0.0067   7.0828e-006
                0.0063   6.9373e-008 7.5080e-006
                0.0067   4.7738e-007 -3.0355e-008 7.0896e-006

GAL1            0.0066   7.4501e-006
                0.0064   -5.1692e-008 7.3862e-006
                0.0071   7.5543e-007 -9.1949e-008 7.8457e-006

Galway          0.0065   6.8574e-006
                0.0063   4.3389e-008 7.1430e-006
                0.0066   3.5163e-007 -2.3135e-008 6.8698e-006

Limerick        0.0067   7.0943e-006
                0.0063   6.9864e-008 7.5308e-006
                0.0067   4.8099e-007 -2.5293e-008 7.0850e-006

*****************************************************************
    VARIANCE FACTOR = 1.3264

    Note: Values < 1.0 indicate statistics are pessimistic, while
          values > 1.0 indicate optimistic statistics. Entering this
          value as the network adjustment scale factor will bring
          variance factor to one.
*****************************************************************
```

Enclosure 3 Static Position Check Mark Diagram

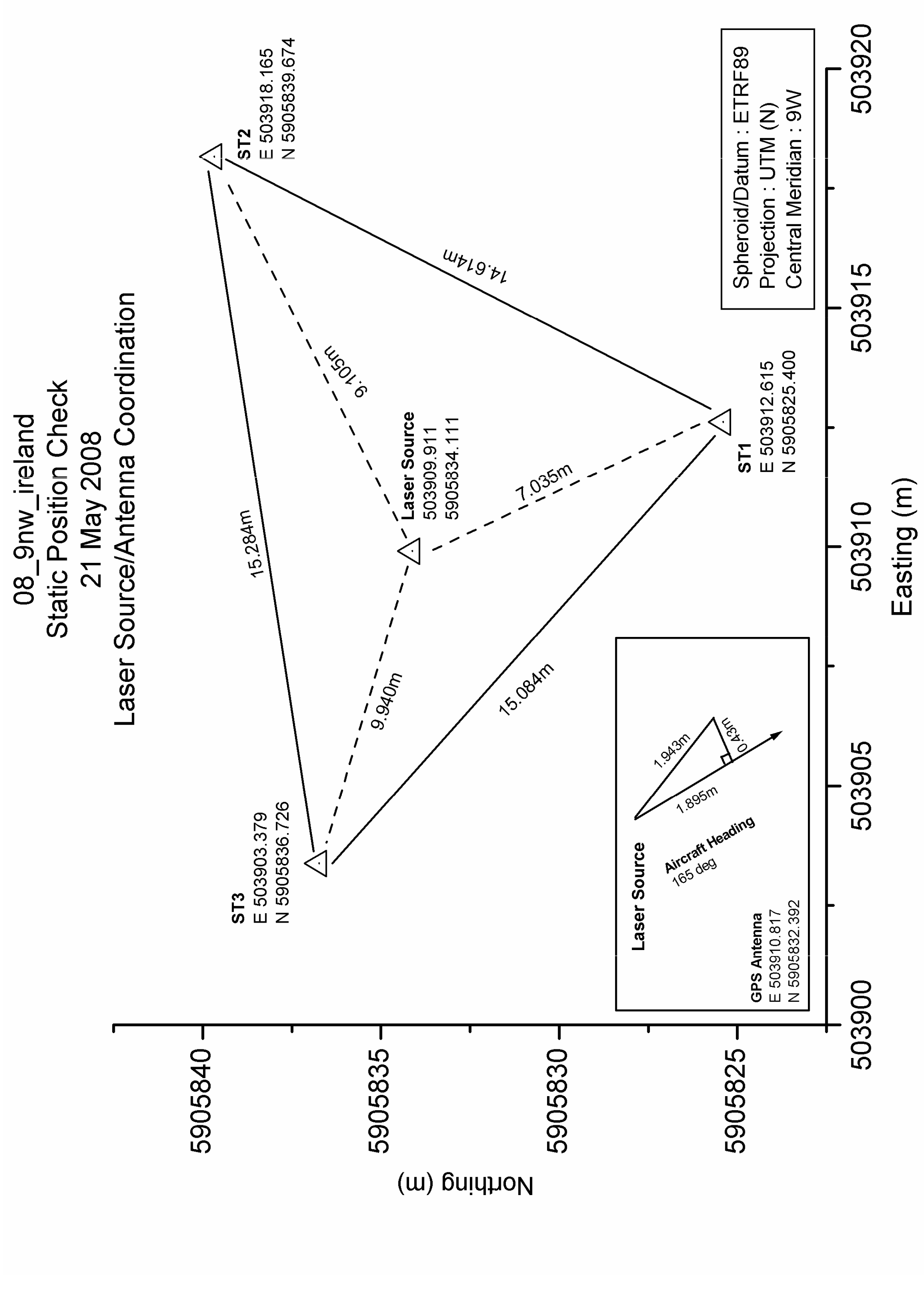

Enclosure 4 Arrigans Coordination Report.

Final Check Survey Radisson Hotel and Galway Airport.
Insertion of Station Points for Bathymetric Survey. 19.05.2008.

Name	Lat	Long	Elip Ht	Lat	Long	ITM East	ITM Nor	Lvl (M Hd)	ING East	ING Nor	Lvl (M Hd)	UTM Zone 29
GH1	53.30117793	-8.941631316	79.794	53ø18'04.24054"N	8ø56'29.87274"W	537234.384	728289.882	22.346	137270.061	228261.405	22.346	503889.826,5905776.544,22.346,Malin Head
GH2	53.30102793	-8.942541782	79.381	53ø18'03.70054"N	8ø56'33.15041"W	537173.489	728273.992	21.934	137209.153	228245.511	21.934	503829.169,5905759.794,21.934,Malin Head
Galway	53.2806809	-9.060736208	86.506	53ø16'50.45125"N	9ø03'38.65035"W	529261.694	726120.478	29.114	129295.663	226091.48	29.114	495950.460,5903496.417,29.114,Malin Head
RAD1	53.27405118	-9.043757455	84.19	53ø16'26.58425"N,	9ø02'37.52684"W	530383.161	725366.137	26.81	130417.368	225336.992	26.81	497082.046,5902758.053,26.810,Malin Head
RAD2	53.27359865	-9.044440569	72.067	53ø16'24.95512"N	9ø02'39.98605"W	530336.867	725316.454	14.688	130371.065	225287.298	14.688	497036.465,5902707.742,14.688,Malin Head
ST1			79.466	53ø18'05.822"N	8ø56'28.640"W	537257.856	728338.416	22.017	137293.547	228309.989	22.017	503912.615,5905825.400,22.017,Malin Head
ST2			79.298	53ø18'06.283"N	8ø56'28.339"W	537263.611	728352.626	21.849	137299.294	228324.162	21.849	503918.165,5905839.674,21.849,Malin Head
ST3			79.228	53ø18'06.188"N	8ø56'29.138"W	537248.781	728349.884	21.779	137284.461	228321.419	21.779	503903.379,5905836.726,21.779,Malin Head

Annex D. Position Fixing Systems and Position Fixing System Validation Results

D.1 Positioning Fixing Systems

Throughout the survey the real-time position of the LADS Mk II system was derived from an Ashtech GG24 GPS receiver, with corrections received via Fugro OmniStar Wide Area Differential GPS (WADGPS) using the Virtual Base Station (VBS) service.

The KGPS (L1/L2 carrier phase) position was obtained by simultaneous GPS data logging throughout each sortie with two Ashtech Z12 dual frequency GPS receivers; the reference receiver at the coordinated local GPS base station in Galway and the roving receiver onboard the aircraft. Following each flight the KGPS data was post-processed in GrafNav software before being imported into the Ground System where it was then applied to all soundings. The dynamic position check function in the Ground System was used to calculate statistics on the accuracy of the real-time WADGPS positioning.

D.2 GPS Base Station Coordinate Validation

Arrigan Geo-Surveyors of Galway provided a check on the LADS GPS Base station at the Radisson SAS Hotel. A short static session of 1 hour was used by Arrigan Geo-Surveyors to obtain the coordinates. The differences in the coordinates calculated by LADS and Arrigan are shown in table 1.

Source	Easting	Northing	Elevation	ΔE	ΔN	ΔEl
LADS Survey	497 082.046	5 902 758.068	85.18	-	-	-
Arrigan Surveyors	497 082.046	5 902 758.053	85.22	0.000	0.015	-0.040

Table 1- LADS GPS Base Station coordinate comparison

D.3 GPS Static Position Check

On 21 May 2008, a static position check of the LADS Mk II positioning system was undertaken using the derived aircraft GPS antenna position, as determined by the static position check control marks at Galway International Airport. Two observation sessions took place, one using Fugro OmniStar VBS WADGPS real-time corrections and the other using stand-alone GPS. Additionally, during these periods the roving dual frequency GPS receiver on the aircraft logged data simultaneously with the local GPS base station. Post-processing of this data provided KGPS (L1/L2 carrier phase) positions for the aircraft GPS antenna.

D.3.1 Session 1 – WADGPS

Session 1 used real-time differential corrections from the Fugro OmniStar VBS WADGPS service combined with stand-alone GPS from an Ashtech GG24 GPS receiver to provide a differentially corrected real-time position for the Airborne System. The local GPS base station and the roving receiver commenced recording GPS data a short time before AS logging commenced. The Fugro OmniStar VBS WADGPS corrected position was recorded on tape using the GPS manual logging function on the AS. This position was recorded for two hours.

D.3.2 Session 2 – GPS Only

Session 2 used no real-time differential corrections. The local GPS base station and the roving receiver began recording GPS data a short time before AS logging commenced. The AS was set to receive no differential corrections and this resulted in a stand-alone GPS position. The position data was recorded on tape using the manual logging function on the AS. This position was recorded for two hours.

D.3.3 Observations

The observation periods were as follows:

Session 1	Start Time (UTC)	Stop Time (UTC)	Logging Duration	Average Number of GPS Satellites
Airborne System - WADGPS	11:59:04	14:01:13	2 hr 02 min	11
GrafNav KGPS	11:54:27	14:01:31	2 hr 02 min	11
**Session 2**	**Start Time (UTC)**	**Stop Time (UTC)**	**Logging Duration**	**Average Number of GPS Satellites**
Airborne System - GPS Only	14:08:02	16:11:20	2 hr 03 min	9
GrafNav KGPS	14:04:31	16:13:26	2 hr 09 min	9

Table 2 Static Position Check Observation Sessions

The AS GPS observables were recorded manually every ten minutes. The easting, northing, height, PDOP, EHE, EVE and number of GPS satellites used were observed and recorded.

D.3.4 Processing

The KGPS positions were produced by processing the reference station file and the aircraft file with GrafNAV software. A KGPS position is produced by solving for the carrier phase ambiguity and using double differencing and forward and backward processing techniques. Both the stand-alone GPS file and the WADGPS file are produced in real-time on the AS and the solution was logged directly to tape. The files were then processed using Position Analysis Software on the GS.

D.3.5 Results

The final positions were exported to a commercial spreadsheet/graphical based software package where calculations of means and standard deviations were completed and scatter plots produced. The calculation of these results was completed in a commercial spreadsheet application, this sheet in enclosed in Enclosure 2.

Table 3 shows the comparison of different static GPS solutions for the position of the aircraft GPS antenna.

	Easting	Northing			
**Absolute Position**	503 910.817	5 905 832.392			
	**Easting ±σE**	**Northing ±σN**	**Δ East C–O (m)**	**Δ North C–O (m)**	**Absolute Accuracy (95% conf)**
**Session 1 - WADGPS**	503 911.573	5 905 832.548	-0.756	-0.156	2.728
	**+/-** 0.417	**+/-** 0.681			
**Session 1 - KGPS**	503 910.851	5 905 832.498	-0.034	-0.106	0.170
	**+/-** 0.016	**+/-** 0.018			
**Session 2 - GPS Only**	503 911.123	5 905 834.237	-0.306	-1.845	6.109
	**+/-** 1.054	**+/-** 1.372			
**Session 2 - KGPS**	503 910.849	5 905 832.503	-0.032	-0.111	0.170
	**+/-** 0.010	**+/-** 0.020			

Table 3 Static Position Check Results

Note: Absolute Accuracy (95% Confidence) = $2.45\,(\sigma E^2 + \sigma N^2)^{½} + (\Delta\,East^2 + \Delta\,North^2)^{½}$

The stated theoretical accuracy of each of the positioning systems has been compared against the absolute accuracy achieved during the static position check in the following table:

Positioning System	Baseline Distance (km)	Theoretical GPS Accuracy (m) (95% confidence)	Absolute Accuracy (m) (95% confidence)	Notes
Session 1 - WADGPS	N/A	N/A	2.728	1
Session 1 - KGPS	7.49	0.300	0.170	
Session 2 - Raw GPS	N/A	14.000	6.109	
Session 2 - KGPS	7.49	0.300	0.170	

Table 4 Theoretical GPS Positioning Accuracies Achieved

Notes:

1: WADGPS corrections supplied by the Fugro OmniStar VBS service were used in real-time to control the aircraft navigation during the flight. Accuracies achieved by this service will be similar to those achieved in the static position check.

A compilation of graphs illustrating the spread of solved positions for each positioning system is provided at Enclosure 1. These graphs show the mean point of recorded positions and the position of the actual antenna as determined by coordination of the laser source using resection techniques and applying the laser source - antenna offset.

D.3.6 Conclusion

The absolute accuracy of the logged WADGPS position solution based on a virtual base station within the survey area was consistent with previous results and was sufficient for the real-time positioning of the aircraft.

The KGPS position yielded a more accurate result and this positioning solution was subsequently applied to all survey data.

The position check of the three systems shows that there are no gross errors.

D.4 Dynamic Position Check

During each sortie, GPS data was logged both on the aircraft and at the base station which enabled a KGPS position solution to be determined. These positions were then compared to the position as determined by the real-time positioning system. For each survey line the mean difference and standard deviation have been calculated. Table 5 shows the mean and standard deviation of the difference in position between the real-time positioning system and the post-processed KGPS for each data collection sortie.

Sortie No.	Lines Flown	Max. Difference AS – KGPS (m)	Mean Difference AS – KGPS (m)	Overall Mean Standard Deviation (m)
4	29	2.47	0.96	0.19
5	36	2.59	0.99	0.19
6	10	2.02	0.99	0.16
7	35	3.22	1.05	0.17
8	25	2.34	0.91	0.24
9	10	1.98	1.22	0.23
10	10	2.45	1.02	0.17
11	37	2.90	1.08	0.19
12	12	2.16	0.95	0.14
13	38	2.52	1.11	0.17
14	9	1.91	0.81	0.14
15	33	1.86	0.83	0.16
16	31	2.21	0.83	0.17
17	18	2.57	1.05	0.23
18	31	2.28	0.96	0.18
19	52	4.23	0.88	0.19
20	40	2.07	0.87	0.19
Total	456	Max. Value 4.23	Mean Value 0.97	Mean Value 0.17

Table 5 Dynamic Position Check Results

These results show good agreement between the real-time position and the post-processed KGPS position. An extract from the dynamic GPS position check report for Sortie 5 is provided in Enclosure 3.

D.5 Navigation Check

Navigation checks were conducted over the GPS Base Station on the rooftop of the Radisson SAS Hotel in Galway on five occasions during the survey.

D.5.1 Navigation Check Results

The logged aircraft position over the base station during the position check was processed against the downward looking digital camera record to determine the difference in position at the time of overflight. This provided a gross error check of the aircraft positioning.

A position for the coordinated mark was placed on the digital camera image. The X, Y pixel values numbers for the coordinated mark on the image were then entered into the Ground System which combined them with the platform pitch and roll, aircraft position, aircraft heading and time over the mark to compute the actual offsets in eastings and northings in metres.

All offsets computed were assigned a confidence of 1 by the Ground System and given a hydrographic confidence of 1 by the System Operator responsible for the navigation position check. The table below shows the offsets achieved.

			∆ East (m)		∆ North (m)	
**Nav Check Name**	**No. of Sorties Flown**	**No. of Detections**	**Mean**	**Standard Deviation**	**Mean**	**Standard Deviation**
Radisson SAS Hotel GPS Base Station	5	5	0.8	0.8	-1.9	0.7

Table 6 Navigation Check Results

These results show that there were no gross errors in the navigation system for the survey.

Enclosures:

1. Static Position Check Graphs.
2. Static Position Check Calculation Sheet
3. Example Dynamic Position Check Report from Sortie 5.

Enclosure 1 – Static Position Check Graphs

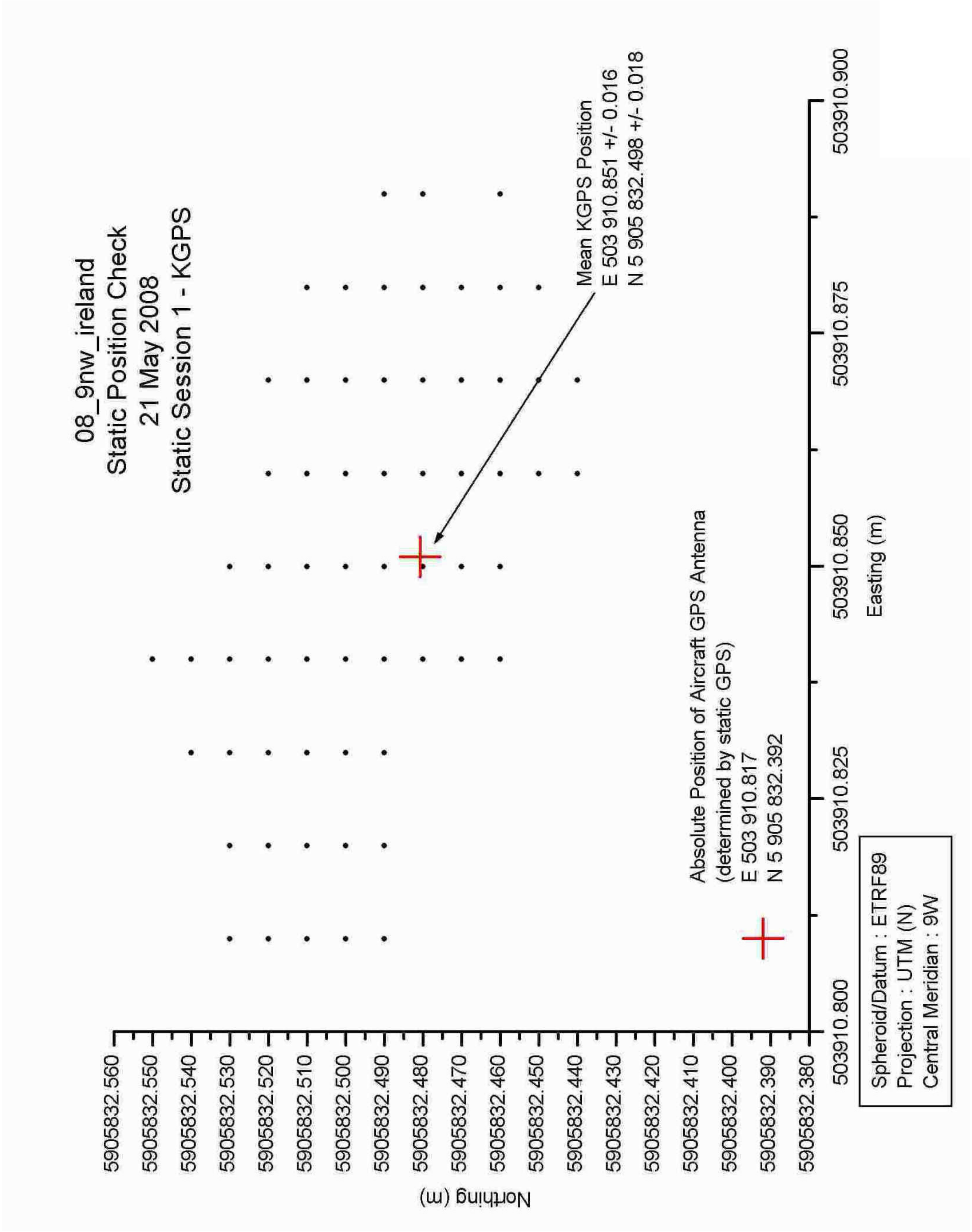

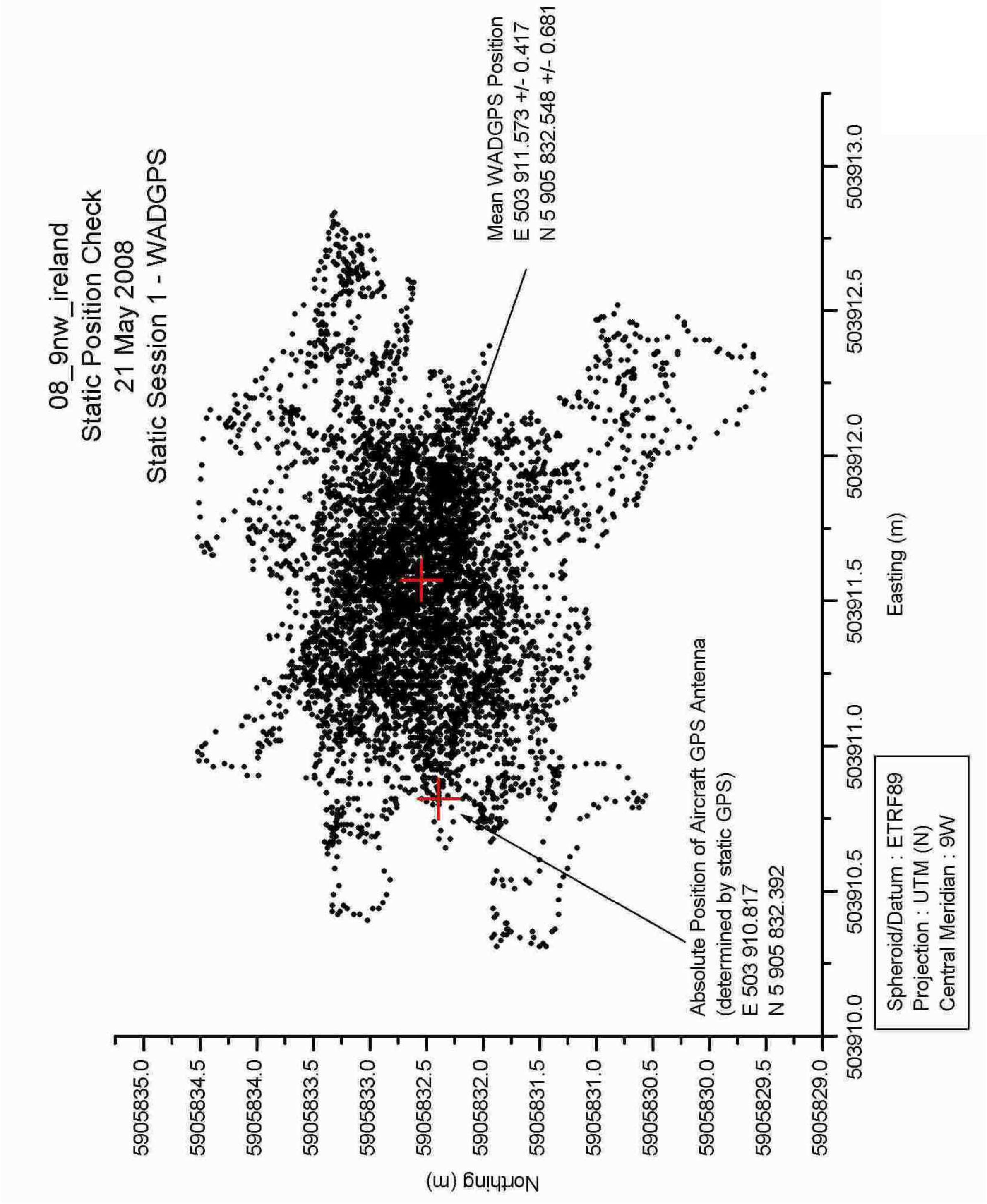
08_9nw_ireland
Static Position Check
21 May 2008
Static Session 1 - WADGPS
Mean WADGPS Position
E 503 911.573 +/- 0.417
N 5 905 832.548 +/- 0.681
Absolute Position of Aircraft GPS Antenna
(determined by static GPS)
E 503 910.817
N 5 905 832.392
Spheroid/Datum : ETRF89
Projection : UTM (N)
Central Meridian : 9W
Easting (m)
Northing (m)
503910.0
503910.5
503911.0
503911.5
503912.0
503912.5
503913.0
5905829.0
5905829.5
5905830.0
5905830.5
5905831.0
5905831.5
5905832.0
5905832.5
5905833.0
5905833.5
5905834.0
5905834.5
5905835.0

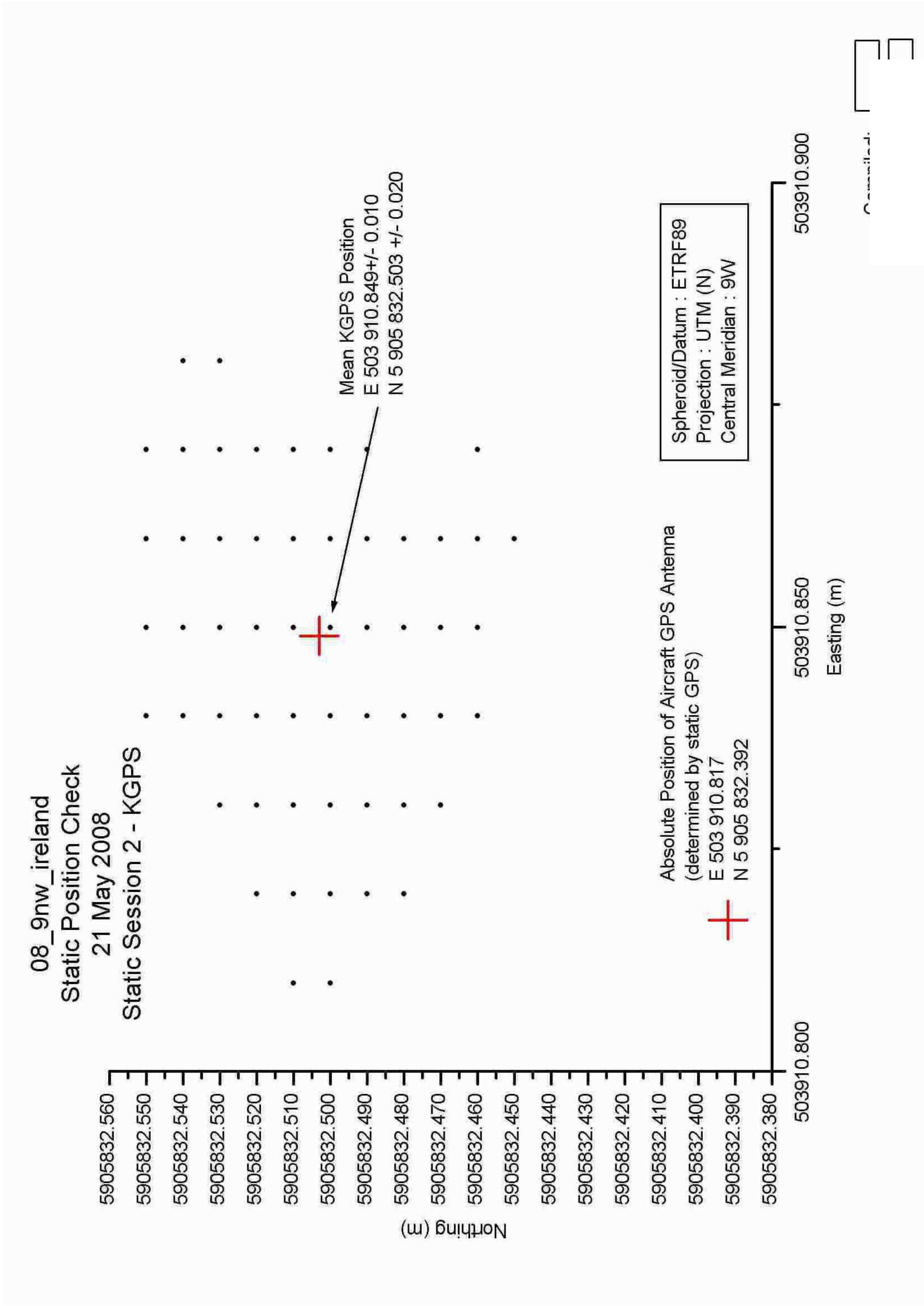
08_9nw_ireland
Static Position Check
21 May 2008
Static Session 2 - KGPS
Mean KGPS Position
E 503 910.849+/- 0.010
N 5 905 832.503 +/- 0.020
Absolute Position of Aircraft GPS Antenna
(determined by static GPS)
E 503 910.817
N 5 905 832.392
Spheroid/Datum : ETRF89
Projection : UTM (N)
Central Meridian : 9W
Northing (m)
5905832.560
5905832.550
5905832.540
5905832.530
5905832.520
5905832.510
5905832.500
5905832.490
5905832.480
5905832.470
5905832.460
5905832.450
5905832.440
5905832.430
5905832.420
5905832.410
5905832.400
5905832.390
5905832.380
503910.800
503910.850
503910.900
Easting (m)

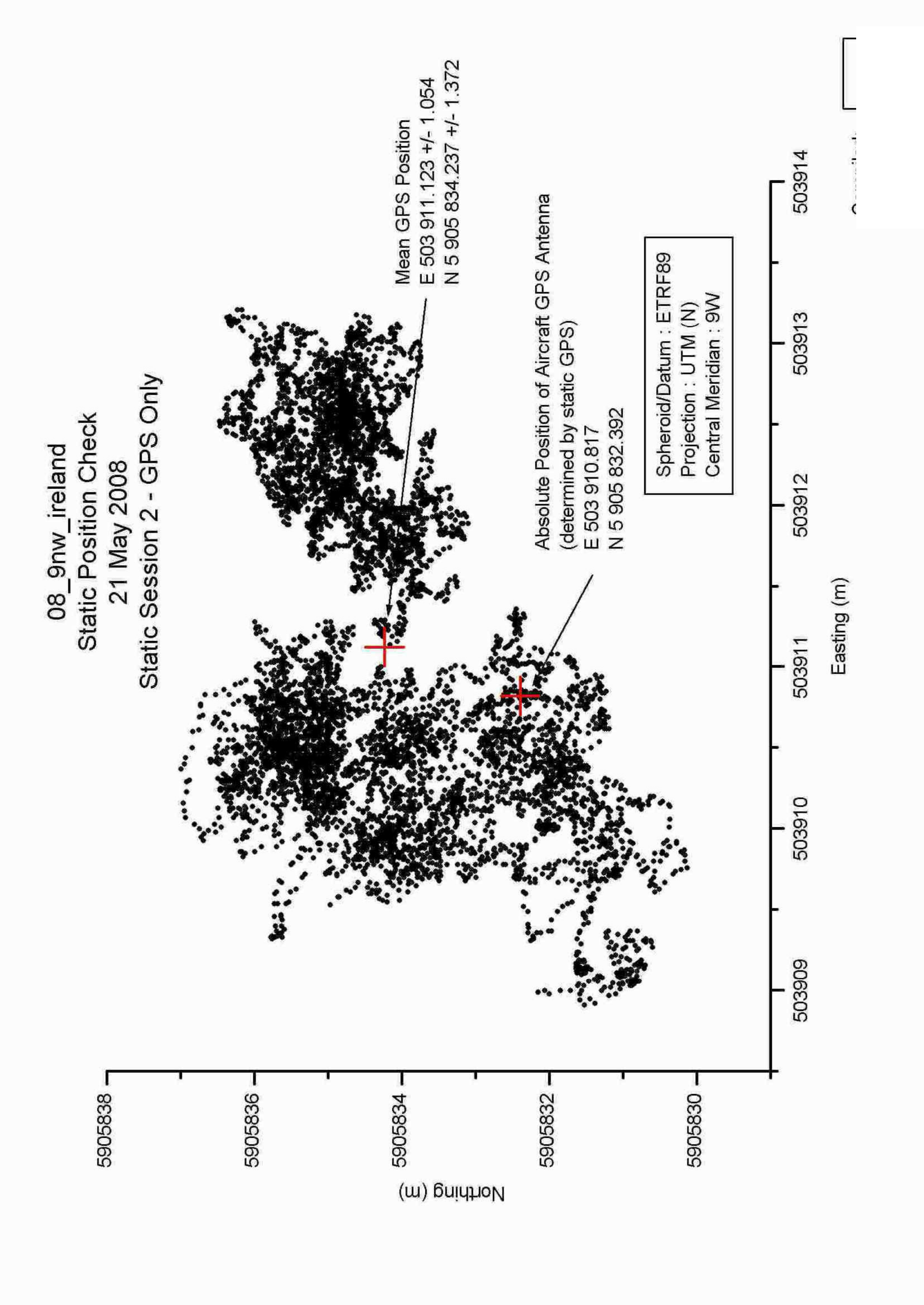
08_9nw_ireland
Static Position Check
21 May 2008
Static Session 2 - GPS Only
Mean GPS Position
E 503 911.123 +/- 1.054
N 5 905 834.237 +/- 1.372
Absolute Position of Aircraft GPS Antenna
(determined by static GPS)
E 503 910.817
N 5 905 832.392
Spheroid/Datum : ETRF89
Projection : UTM (N)
Central Meridian : 9W
503909
503910
503911
503912
503913
503914
Easting (m)
5905838
5905836
5905834
5905832
5905830
Northing (m)

Enclosure 2 – Static Position Check Calculation Sheet

Static Position Check Calculation Sheet

Survey Title: NW Ireland

Database Name: 08_9nw_ireland

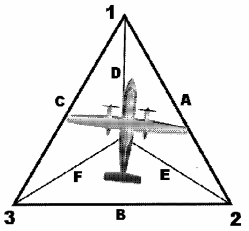

Segment	Taped (m)	Check
A	14.614	0.000
B	15.084	0.007
C	15.284	-0.031
D	9.105	
E	7.035	
F	9.940	

Aircraft Heading	165°

Point ID	EASTING	NORTHING	DATUM	SOURCE
1	503912.615	5905825.400	ETRF89	Arrigan
2	503903.379	5905836.726	ZONE	
3	503918.165	5905839.674	29N	
Laser Source	**503909.911**	**5905834.111**		
GPS Absolute	**503910.817**	**5905832.392**		

	Easting	Northing			
**Absolute Position**	503910.817	5905832.392			
	**Easting ±σE**	**Northing ±σN**	**Δ East C–O (m)**	**Δ North C–O (m)**	**Absolute Accuracy (95% conf)**
**Session 1 WADGPS**	503911.573 +/- 0.417	5905832.548 +/-0.681	-0.756	-0.156	2.728
**Session 1 KGPS**	503910.851 +/- 0.016	5905832.498 +/- 0.018	-0.034	-0.106	0.170
**Session 2 GPS Only**	503911.123 +/- 1.054	5905834.237 +/- 1.372	-0.306	-1.845	6.109
**Session 2 KGPS**	503910.849 +/- 0.010	5905832.503 +/- 0.020	-0.032	-0.111	0.170

Note: Absolute Accuracy (95% Confidence) = 2.45 (σE2 + σN2) ½ + (Δ East2 + Δ North2)½

Triangle Solutions

Side	Side Length	Squared	Cos Rule	Acos(rad)	DD	DMS	Angle	Check	
A	14.614	213.568996	-0.6336618937	2.2570739281	129.32081	129:19:15	a		TRI 1
E	7.035	49.491225	0.9280697566	0.3816005849	21.86410	21:51:51	e		
D	9.105	82.901025	0.8761797962	0.5029181405	28.81509	28:48:54	d	180.00000	
B	15.084	227.527056	-0.5665280310	2.1730827120	124.50847	124:30:30	b		TRI 2
F	9.94	98.803600	0.8397168277	0.5740347873	32.88977	32:53:23	f		
E	7.035	49.491225	0.9231984022	0.3944751543	22.60176	22:36:06	e	180.00000	
C	15.284	233.600656	-0.2867066816	1.8615837518	106.66089	106:39:39	c		TRI 3
D	9.105	82.901025	0.8211505062	0.6073723071	34.79987	34:48:00	d		
F	9.94	98.803600	0.7821816550	0.6726365947	38.53924	38:32:21	f	180.00000	
					360.49017	360:29:25	Check		

Join Calculation between Stat-Cal Triangle marks

Line	de	dn	dist	BRG	RawDegBRG	DegBRG	DegBRG
1-2	9.236	-11.326	14.614	-0.68410	-39.19617	320.80383	140.80383
2-3	-14.786	-2.948	15.077	1.37400	78.72435	78.72435	258.72435
3-1	5.55	14.274	15.315	0.37083	21.24702	21.24702	201.24702

Bearing Quadrant Calculations

Column	Row	Quadrant	Min	Max	AntiClockwise	Clockwise
1	2	2	90	180	140.80383	320.80383
2	2	3	180	270	258.72435	78.72435
1	1	1	0	90	21.24702	201.24702

Quadrant	Min	Max
1	0	90
2	90	180
3	180	270
4	270	360

Laser Source Coordinate Calculation

From P	NewRawBRG	NewBRG	dist	de	dn	New E	New N	
1	-17.29222	-17.29222	9.105	-2.706	8.693	503909.909	5905834.093	
1	342.66793	342.66793	9.105	-2.712	8.692	503909.903	5905834.092	
2	111.98874	111.98874	7.035	6.523	-2.634	503909.902	5905834.092	
2	111.61412	111.61412	7.035	6.540	-2.591	503909.919	5905834.135	
3	236.12259	236.12259	9.94	-8.253	-5.541	503909.912	5905834.133	
3	236.04689	236.04689	9.94	-8.245	-5.552	503909.920	5905834.122	
						503909.911	5905834.111	Average
						+/- 0.007	+/- 0.019	Std Deviation

GPS Antenna Coordinate Calculation

otenuse	Angle from heading	DD	NewBrgRaw	NewBRG	DMS	de	dn	GPS EAST	GPS NORTH
1.943	347:12:51	347.21417	512.21417	152.2141667	152:12:51	0.906	-1.719	**503910.817**	**5905832.392**

Enclosure 3 – Example Dynamic Position Check Report from Sortie 5

```
GS VERSION E8.5/010
POSITION ANALYSIS REPORT FOR SORTIE  5 FLOWN ON 145 2008 (24/05/08)
MISSION TITLE: NORTH WEST IRELAND 2008
RUN COUNT:  36
------------------------------------------------------------------
Statistics for Run 3.0.1
GPS Start Time 14:59:38 End Time 15:02:22

DGPS_HRMS                   Min=   0.000m  Max=   0.000m
KGPS_HRMS                   Min=   0.047m  Max=   0.047m
AS_EHE                      Min=   0.917m  Max=   0.958m
AS_PDOP                     Min=   1.70m   Max=   1.70m
POST_PROCESSED_PDOP         Min=   1.40m   Max=   1.40m
AS_SVS                      Min= 9         Max= 9
POST_PROCESSED_SVS          Min= 8         Max= 8
AS_LATENCY_OF_CORRECTIONS   Min=  4s       Max= 17s

Analysis of Reported GPS Position

Airborne System - DGPS (Sample Size: AS =  165 DGPS =  0)
Latitude    Min=  0.000m  Max=  0.000m  Mean=  0.000m   Stdev=  0.000m
Longitude   Min=  0.000m  Max=  0.000m  Mean=  0.000m   Stdev=  0.000m
RMS         Min=  0.000m  Max=  0.000m  Mean=  0.000m   Stdev=  0.000m

Airborne System - KGPS (Sample Size: AS =  165 KGPS =  165)
Latitude    Min=  0.190m  Max=  1.165m  Mean=  0.506m   Stdev=  0.194m
Longitude   Min= -0.414m  Max=  0.025m  Mean= -0.131m   Stdev=  0.091m
RMS         Min=  0.207m  Max=  1.169m  Mean=  0.533m   Stdev=  0.187m

DGPS - KGPS (Sample Size: DGPS =  0 KGPS =  165)
Latitude    Min=  0.000m  Max=  0.000m  Mean=  0.000m   Stdev=  0.000m
Longitude   Min=  0.000m  Max=  0.000m  Mean=  0.000m   Stdev=  0.000m
RMS         Min=  0.000m  Max=  0.000m  Mean=  0.000m   Stdev=  0.000m

DGPS - AS Height (Sample Size: AS =  165 DGPS =  0)
WGS84 Ht    Min=  0.000m  Max=  0.000m  Mean=  0.000m

KGPS - AS Height (Sample Size: AS =  165 KGPS =  165)
WGS84 Ht    Min= -2.967m  Max= -1.472m  Mean= -2.201m

Airborne System GPS Mode : diff
Airborne System Racal Basestation : 0100
```

```
-------------------------------------------------------------------
Statistics for Run 4.0.1
GPS Start Time 15:10:15 End Time 15:12:58

DGPS_HRMS                  Min=   0.000m  Max=   0.000m
KGPS_HRMS                  Min=   0.045m  Max=   0.046m
AS_EHE                     Min=   0.917m  Max=   1.000m
AS_PDOP                    Min=   1.80m   Max=   1.80m
POST_PROCESSED_PDOP        Min=   1.20m   Max=   1.20m
AS_SVS                     Min= 9         Max= 9
POST_PROCESSED_SVS         Min= 9         Max= 9
AS_LATENCY_OF_CORRECTIONS  Min=  7s       Max= 17s

Analysis of Reported GPS Position

Airborne System - DGPS (Sample Size: AS =  164 DGPS =  0)
Latitude    Min=  0.000m  Max=  0.000m  Mean=  0.000m   Stdev=  0.000m
Longitude   Min=  0.000m  Max=  0.000m  Mean=  0.000m   Stdev=  0.000m
RMS         Min=  0.000m  Max=  0.000m  Mean=  0.000m   Stdev=  0.000m

Airborne System - KGPS (Sample Size: AS =  164 KGPS =  164)
Latitude    Min= -0.701m  Max= -0.126m  Mean= -0.411m   Stdev=  0.133m
Longitude   Min=  0.084m  Max=  0.775m  Mean=  0.380m   Stdev=  0.178m
RMS         Min=  0.183m  Max=  0.893m  Mean=  0.574m   Stdev=  0.183m

DGPS - KGPS (Sample Size: DGPS =  0 KGPS =  164)
Latitude    Min=  0.000m  Max=  0.000m  Mean=  0.000m   Stdev=  0.000m
Longitude   Min=  0.000m  Max=  0.000m  Mean=  0.000m   Stdev=  0.000m
RMS         Min=  0.000m  Max=  0.000m  Mean=  0.000m   Stdev=  0.000m

DGPS - AS Height (Sample Size: AS =  164 DGPS =  0)
WGS84 Ht    Min=  0.000m  Max=  0.000m  Mean=  0.000m

KGPS - AS Height (Sample Size: AS =  164 KGPS =  164)
WGS84 Ht    Min= -2.586m  Max= -0.779m  Mean= -1.575m

Airborne System GPS Mode : diff
Airborne System Racal Basestation : 0100
```

Annex E. Vertical Datum, Tidal Model and Tide Stations

E.1 Vertical Datum

All depths were reduced to Malin Head Ordnance Datum (MHOD) for Galway Bay, Tralee Bay and Blacksod Bay, and to Belfast Lough Ordnance Datum (BLOD) for Lough Foyle, using corrections derived from observed tides.

A second set of digital data was generated reduced to Lowest Astronomical Tide (LAT) for all areas for subsequent delivery to UKHO for charting purposes. LAT connections were calculated using geodetic information and software supplied by the GSI. LAT connections at each gauge location are highlighted in Enclosure 4.

E.2 Tidal Models

The survey was carried out in five distinct regions. Areas surveyed included Tralee Bay, Galway Bay, Donegal Bay, Blacksod Bay and Lough Foyle. Separate tide models were created for each area. Reduction of tidal data for each of the survey areas is discussed separately below.

To enable an effective tide model to be established, a total of twelve tide stations were incorporated into twenty four tide areas which were used for the vertical control of soundings in each of the survey areas. Tide areas created within Donegal Bay have been excluded from this annex as LADS data collected in the region was not accepted due to water clarity issues.

The tidal model adopted for each area is discussed separately below:

GALWAY BAY

Tide Area 1: soundings were reduced using observed tides from the Galway Port tide gauge.

Tide Area 2: soundings were reduced using a linear interpolation between observed tides at the Galway Port and Inishmore tide gauges.

Tide Areas 3 & 4: soundings were reduced using observed tides from the Inishmore tide gauge.

Tide Area 5: soundings were reduced using a linear interpolation between observed tides at the Rossaveel and Inishmore tide gauges.

Tide Area 6: soundings were reduced using observed tides from the Rossaveel tide gauge.

Tide Area 7: soundings were reduced using a linear interpolation between observed tides at the Galway Port and Rossaveel tide gauges.

Tide Area 8: soundings were reduced using a planar interpolation between observed tides at the Galway Port, Inishmore and Rossaveel tide gauges.

Tide Area 21: soundings were reduced using observed tides from the Inishmore Ocean Buoy tide gauge. Note no data was actually collected in this area.

TRALEE BAY

Tide Area 18: soundings were reduced using observed tides from the Maheree's Pier tide gauge.

Tide Area 19&20: soundings were reduced using a linear interpolation between observed tides at the Maheree's Pier and Fenit Harbour tide gauges.

BLACKSOD BAY

Tide Area 22: soundings were reduced using observed tides from the Blacksod tide gauge.

LOUGH FOYLE

Tide Area 23: soundings were reduced using a linear interpolation between observed tides at the Greencastle and Derry Port tide gauges.

Tide Area 24: soundings were reduced using observed tides from the Greencastle tide gauge.

Chartlets of all areas and lists of coordinates of the tide stations and areas are presented in Enclosures 1 and 2.

E.3 Tidal Reduction

The reduction of soundings within the Ground System is undertaken by means of linear interpolation within tide areas containing two tide stations, planar interpolation within areas containing three stations, and where only one station is incorporated within an area the value from that single station is applied across the entire tidal area.

E.4 Provision of Observed Tide Data

Observed tidal data for the survey was sourced from permanently installed gauges of the Irish National Tide Gauge Network, temporary tide gauges installed by the Geological Survey of Ireland and five temporary tide gauges established by Tenix LADS Corporation surveyors.

Data from tide gauges located at Galway Port, Inishmore and Killybegs were sourced from the Irish National Tide Gauge Network. Tides were provided in UTC time format and reduced to the Malin Head Ordnance Datum (MHOD). The Inishmore Ocean buoy gauge was created within the Ground System to satisfy tide area requirements, for potential data collection in this area, however no data was collected and so this gauge site was not used.

Observed tides for Maheree's Pier, Blacksod Bay and Greencastle tide gauges were supplied by the Geological Survey of Ireland. Data was provided in UTC time and reduced to MHOD.

Tenix LADS Surveyors installed four Valeport 740c tide gauges at Fenit Harbour, Rossaveel, Mullaghmore and Derry Port; along with two bottom mounted Valeport MIDAS water level recorders located in Donegal Bay, all were in UTC time format. See Enclosure 3 for full details of each gauge inserted.

All gauges inserted by Tenix LADS Corporation were fully checked via 25 Hour Gauge / pole comparison and connected to MHOD via closed levelling run to local Survey Control Marks. A 25hr simultaneous logging session was conducted at Mount Charles Pier to transfer datum to the bottom mounted tide gauges.

Enclosures:

1. Chartlets defining Tide Stations and Areas.
2. Coordinate list of Tide Stations and Tide Areas.
3. LADS tide gauge comparisons and datum relationships.
4. LAT connections
5. National Tidal Centre BMTG Analysis

Enclosure 1 – Chartlets Defining Tide Stations and Areas

GALWAY BAY

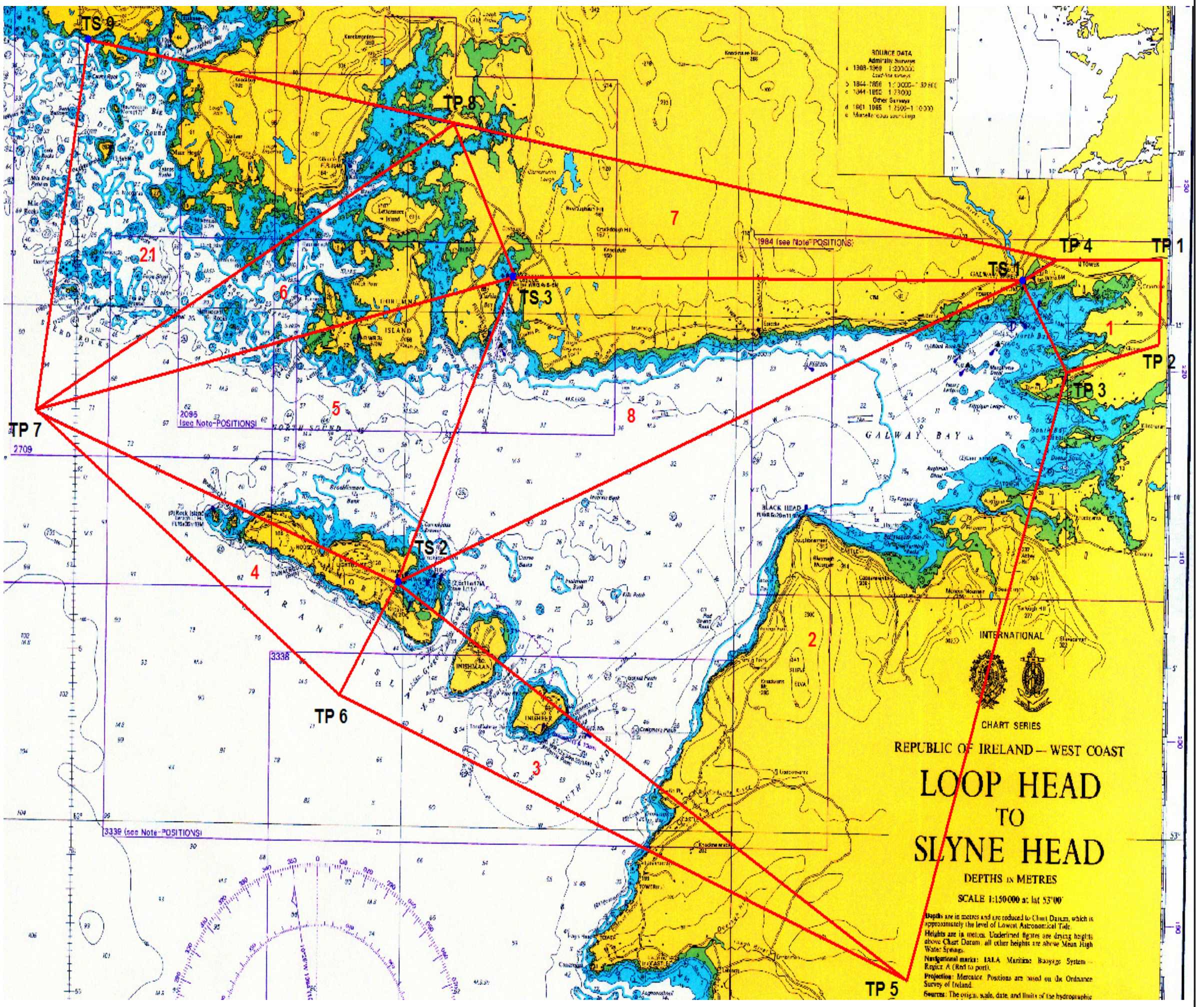

TRALEE BAY

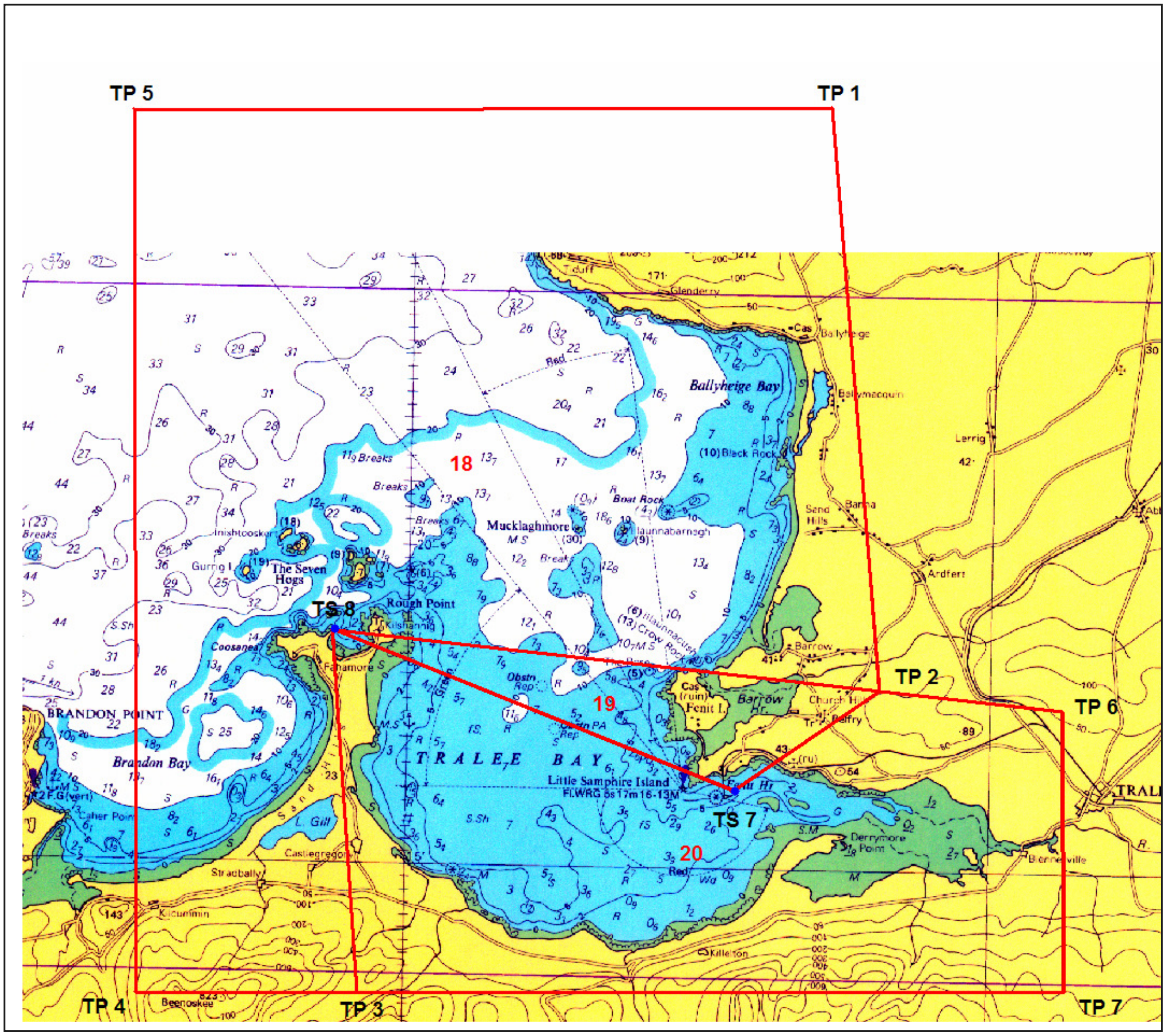
TP 5
TP 1
TP 2
TP 6
TP 7
TP 3
TP 4
TS 8
TS 7
18
19
20
Ballyheige Bay
Mucklaghmore
The Seven Hogs
Rough Point
Brandon Bay
BRANDON POINT
TRALEE BAY
Little Samphire Island
Fenit I.
Barrow
Derrymore Point
Ardfert
Castlegregory
Stradbally

BLACKSOD BAY

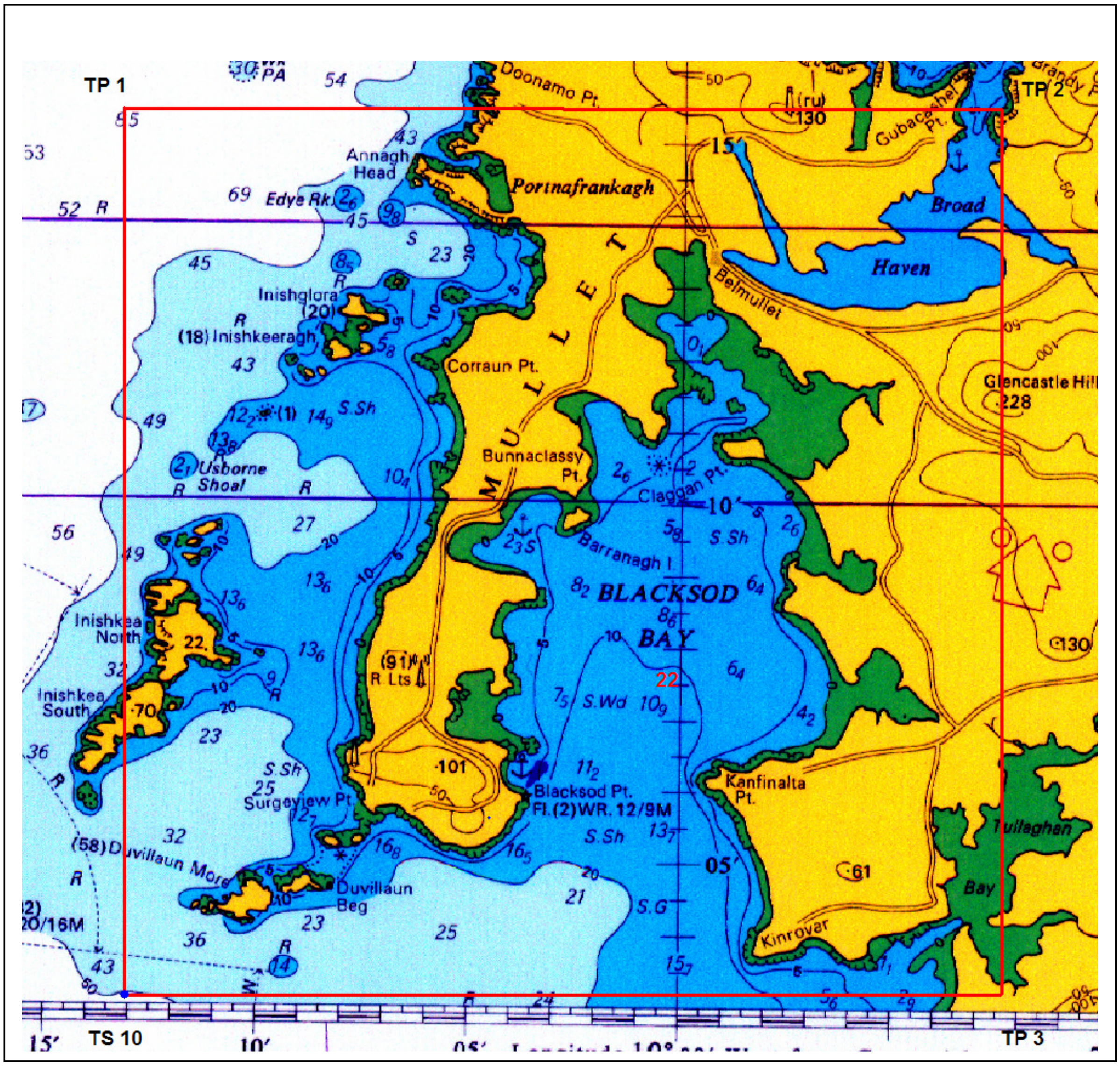

LOUGH FOYLE

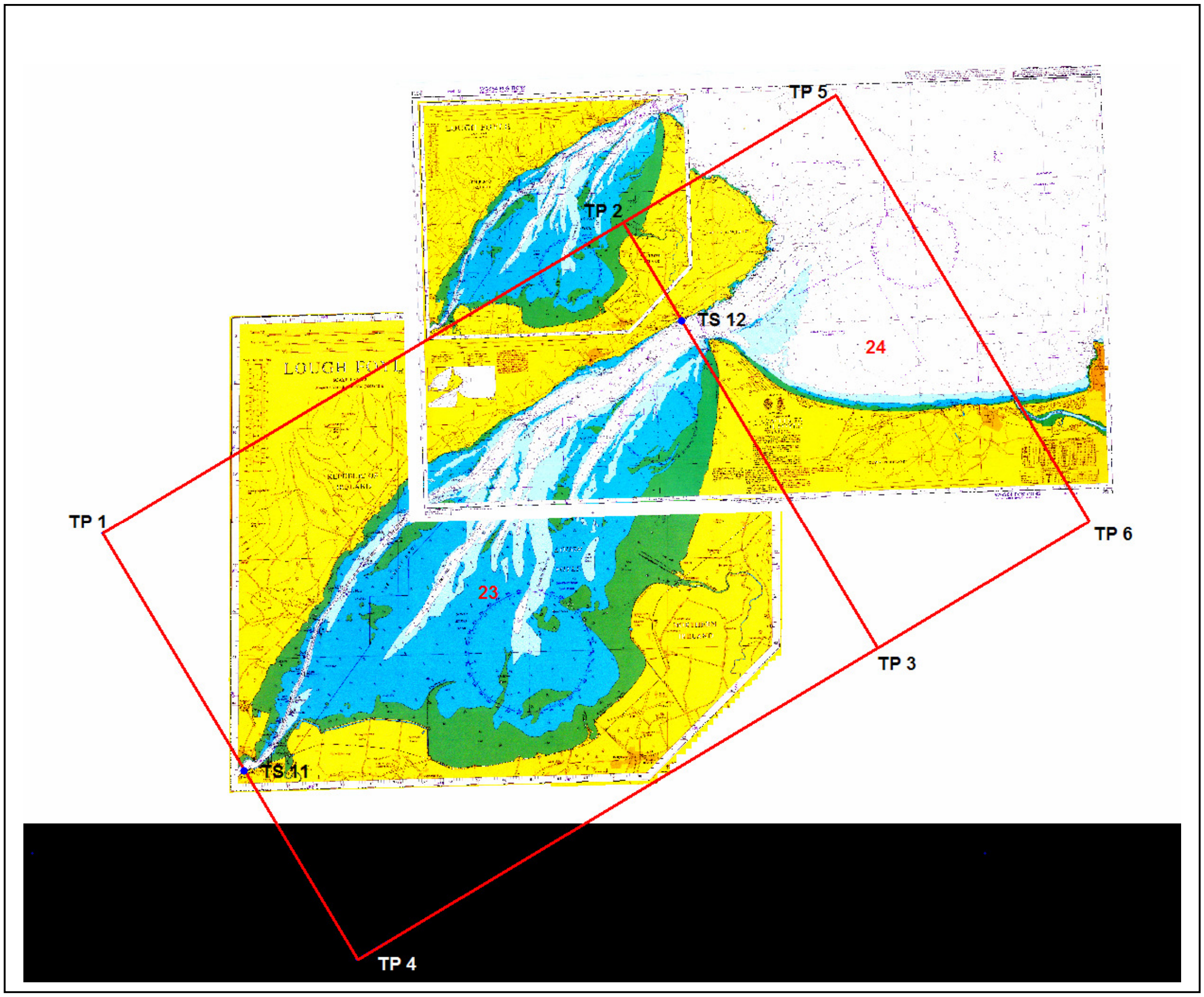

Enclosure 2 – List of Tide Stations and Tide Areas

TS	*Tide Station Name*	*Easting*	*Northing*	*Type*
TS 1	Galway Port	497 260	5 902 367	Observed
TS 2	Inishmore	455 013	5 885 889	Observed
TS 3	Rossaveel	462 908	5 902 580	Observed
TS 4	Mullaghmore	535 956	6 035 496	Observed
TS 5	Killybegs	535 639	6 054 695	Observed
TS 6	BMTG	550 503	6 050 943	Observed
TS 7	Fenit Harbour	441 195	5 791 475	Observed
TS 8	Maheree's Pier	429 598	5 796 177	Observed
TS 9	Inishmore Ocean Buoy (Inishmore tide gauge)	434 163	5 915 433	Observed
TS 10	Blacksod Bay	420 793	5 990 894	Observed
TS 11	Derry Port	610 819	6 100 648	Observed
TS 12	Greencastle	628 337	6 119 219	Observed

Note: Positions for all tide stations are those entered to construct the tide areas, not the actual geographic position.

GALWAY BAY

Tide Area	*Point Name*	*Easting*	*Northing*
1	TP 1	506 740	5 903 436
	TP 2	506 501	5 898 884
	TP 3	500 347	5 897 342
	TS 1	497 260	5 902 367
	TP 4	499 554	5 903 540
2	TS 1	497 260	5 902 367
	TP 3	500 347	5 897 342
	TP 5	489 441	5 864 360
	TS 2	455 013	5 885 889

Tide Area	*Point Name*	*Easting*	*Northing*
3	TS 2	455 013	5 885 889
	TP 5	489 441	5 864 360
	TP 6	451 069	5 879 847
4	TS 2	455 013	5 885 889
	TP 6	451 069	5 879 847
	TP 7	430 612	5 895 357
5	TS 3	462 908	5 902 580
	TS 2	455 013	5 885 889
	TP 7	430 612	5 895 357
6	TS 3	462 908	5 902 580
	TP 7	430 612	5 895 357
	TP 8	458 897	5 910 799
7	TS 1	497 260	5 902 367
	TS 3	462 908	5 902 580
	TP 8	458 897	5 910 799
	TP 4	499 554	5 903 540
8	TS 1	497 260	5 902 367
	TS 2	455 013	5 885 889
	TS 3	462 908	5 902 580
21	TS 9	434 163	5 915 433
	TP 7	430 612	5 895 357
	TP 8	458 897	5 910 799

TRALEE BAY

Tide Area	*Point Name*	*Easting*	*Northing*
18	TP 1	444 182	5 811 397
	TS 8	429 598	5 796 177
	TP 3	430 250	5 785 615
	TP 4	423 774	5 785 615
	TP 5	423 774	5 811 397
	TP2	445 503	5 794 409
19	TS 7	441 195	5 791 475
	TS 8	429 598	5 796 177
	TP2	445 503	5 794 409
20	TP 6	450 880	5 793 811
	TP 7	450 907	5 785 615
	TP 3	430 250	5 785 615
	TS 8	429 598	5 796 177
	TS 7	441 195	5 791 475
	TP2	445 503	5 794 409

BLACKSOD BAY

Tide Area	*Point Name*	*Easting*	*Northing*
22	TS 10	420 793	5 990 894
	TP 1	420 793	6 013 421
	TP 2	443 088	6 013 421
	TP 3	443 088	5 990 894

LOUGH FOYLE

Tide Area	*Point Name*	*Easting*	*Northing*
23	TS 11	610 819	6 100 648
	TP 1	604 939	6 110 506
	TP 2	626 029	6 123 084
	TS 12	628 334	6 119 219
	TP 3	636 304	6 105 856
	TP 4	615 214	6 093 278
24	TS 12	628 334	6 119 219
	TP 2	626 029	6 123 084
	TP 5	634 617	6 128 206
	TP 6	644 892	6 110 978
	TP 3	636 304	6 105 856

Enclosure 3 – LADS tide gauge comparisons and datum relationships

MULLAGHMORE

Tenix LADS surveyors installed a Valport 740c tide gauge and tide pole inside Mullaghmore Harbour on 16 May 2008. A 25-hour tide pole / gauge comparison was conducted and the pole was conducted via a closed levelling run between to a GSI Survey mark to enable MH datum to be recovered. Pole readings were taken every 30 minutes during rising and falling tide periods and at 10 minute intervals over the high and low water periods. These observed values were compared to the Valeport 10 minute logged readings and the height difference between the pole and gauge determined. The analysis also proved that the gauge was logging correctly in both time and range.

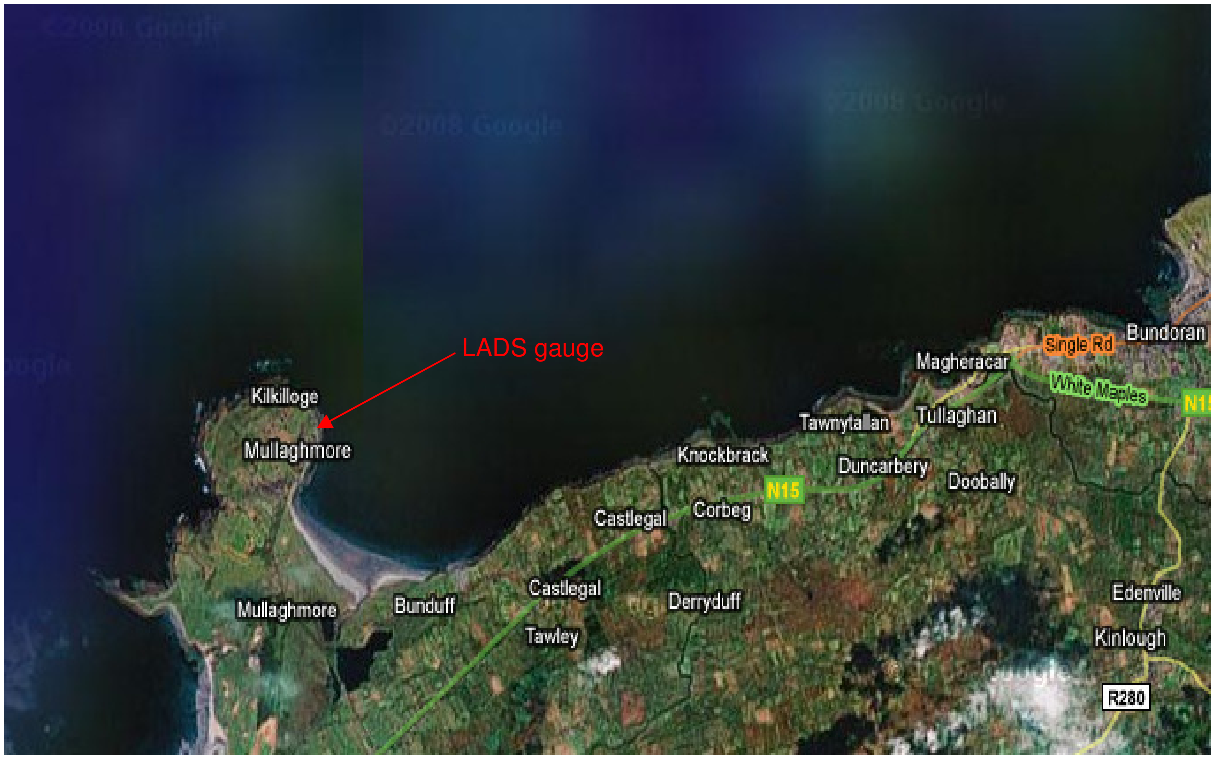

Location of LADS gauge at Mullaghmore Harbour

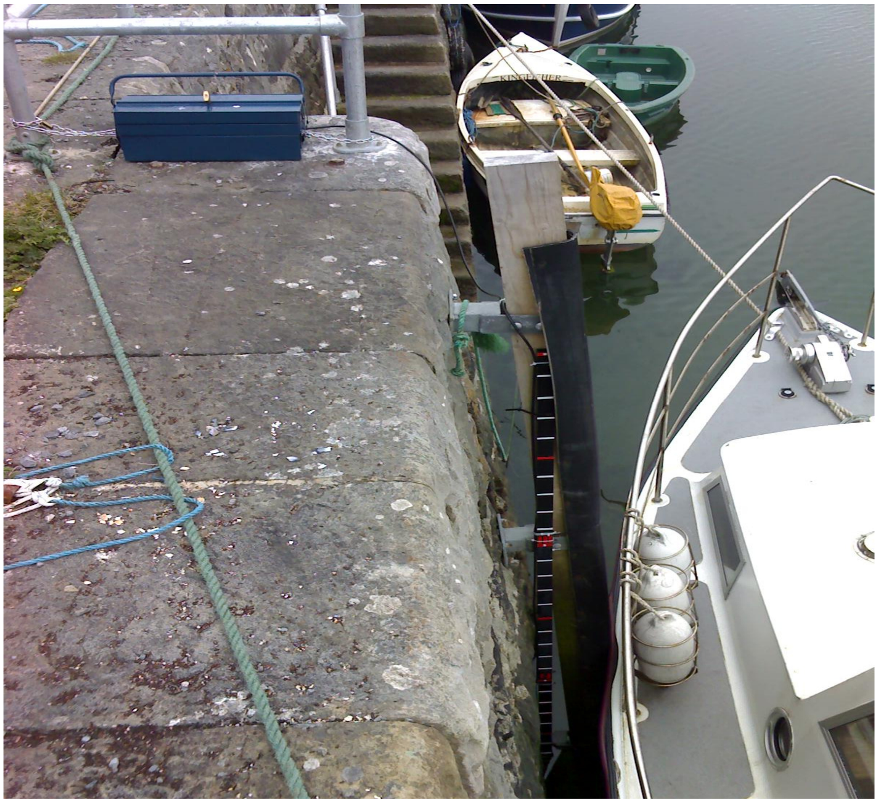

LADS gauge attached to fender on harbour wall

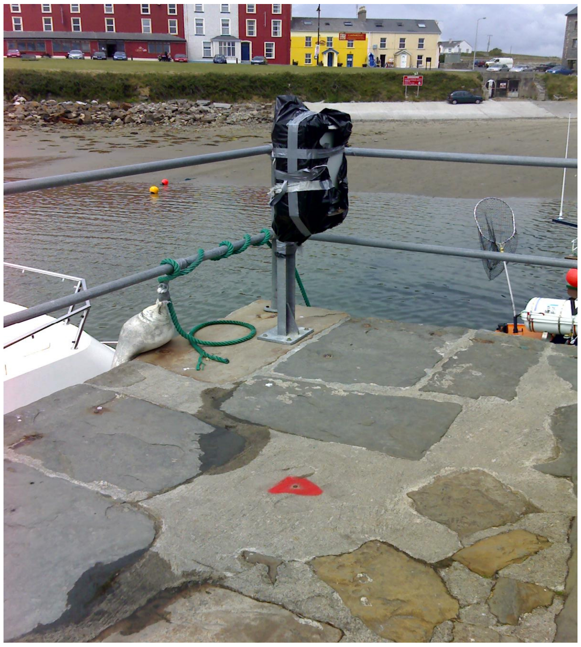

MULLAGHMORE SURVEY MARK DESCRIPTION

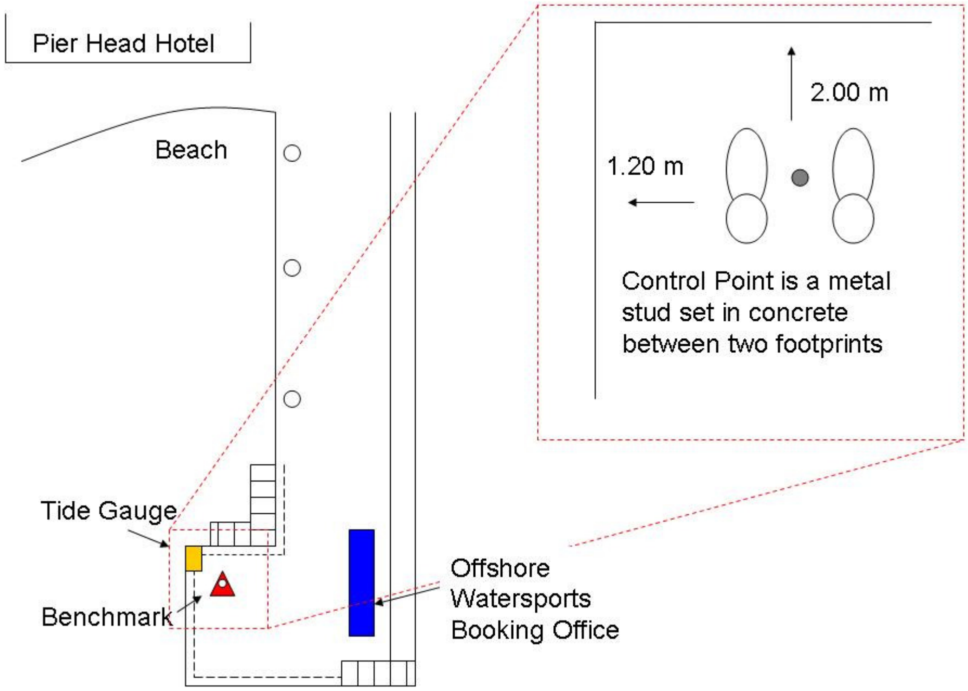

ETRF89_X (m)	ETRF89_Y (m)	ETRF89_Z (m)	E_ITM (m)	N_ITM (m)
3674876.351	-545748.949	5167099.003	571011.472	857572.816
		LAT (m)	E_ING (m)	N_ING (m)
		-2.12	171053.724	357572.189
ETRF89_LAT	ETRF89_LONG	ELLIPSOID_HGT (m)	HGT_ABOVE_GEOID (m)	HGT_ABOVE MALIN HEAD DATUM (m)
54° 27' 56.94656 N	8° 26' 49.73815 W	60.686	3.111	2.951

MULLAGHMORE 25 HOUR POLE / GAUGE COMPARISON

Date	Time (UTC)	LADS Gauge	LADS Pole	Gauge - Pole	LADS Gauge - 0.02m	Diff. LG - LP
16-May-08	19:00:00	1.16	1.15	0.01	1.14	-0.01
16-May-08	19:30:00	0.90	0.88	0.02	0.88	0.00
16-May-08	20:00:00	0.69	0.67	0.02	0.67	0.00
16-May-08	20:30:00	0.55	0.52	0.03	0.53	0.01
16-May-08	20:40:00	0.51	0.49	0.02	0.49	0.00
16-May-08	20:50:00	0.48	0.46	0.02	0.46	0.00
16-May-08	21:00:00	0.45	0.45	0.00	0.43	-0.02
16-May-08	21:10:00	0.41	0.4	0.01	0.39	-0.01
16-May-08	21:20:00	0.40	0.38	0.02	0.38	0.00
16-May-08	21:30:00	0.39	0.37	0.02	0.37	0.00
16-May-08	21:40:00	0.38	0.37	0.01	0.36	-0.01
16-May-08	21:50:00	0.39	0.38	0.01	0.37	-0.01
16-May-08	22:00:00	0.40	0.39	0.01	0.38	-0.01
16-May-08	22:10:00	0.42	0.4	0.02	0.40	0.00
16-May-08	22:20:00	0.46	0.45	0.01	0.44	-0.01
16-May-08	22:30:00	0.50	0.49	0.01	0.48	-0.01
16-May-08	23:00:00	0.66	0.63	0.03	0.64	0.01
16-May-08	23:30:00	0.87	0.88	-0.01	0.85	-0.03
17-May-08	00:00:00	1.13	1.12	0.01	1.11	-0.01
17-May-08	00:30:00	1.42	1.39	0.03	1.40	0.01
17-May-08	01:00:00	1.71	1.69	0.02	1.69	0.00
17-May-08	01:30:00	1.99	1.99	0.00	1.97	-0.02
17-May-08	02:00:00	2.23	2.2	0.03	2.21	0.01
17-May-08	02:30:00	2.42	2.38	0.04	2.40	0.02
17-May-08	03:00:00	2.57	2.54	0.03	2.55	0.01
17-May-08	03:30:00	2.64	2.61	0.03	2.62	0.01
17-May-08	04:00:00	2.66	2.63	0.03	2.64	0.01
17-May-08	04:10:00	2.64	2.61	0.03	2.62	0.01
17-May-08	04:20:00	2.63	2.61	0.02	2.61	0.00
17-May-08	04:30:00	2.61	2.58	0.03	2.59	0.01
17-May-08	05:00:00	2.50	2.49	0.01	2.48	-0.01
17-May-08	05:30:00	2.29	2.25	0.04	2.27	0.02
17-May-08	06:00:00	2.01	1.99	0.02	1.99	0.00
17-May-08	06:30:00	1.67	1.63	0.04	1.65	0.02
17-May-08	07:00:00	1.35	1.32	0.03	1.33	0.01
17-May-08	07:30:00	1.02	1	0.02	1.00	0.00
17-May-08	08:00:00	0.72	0.69	0.03	0.70	0.01
17-May-08	08:30:00	0.48	0.46	0.02	0.46	0.00
17-May-08	09:00:00	0.27	0.26	0.01	0.25	-0.01
17-May-08	09:10:00	0.22	0.21	0.01	0.20	-0.01
17-May-08	09:20:00	0.19	0.18	0.01	0.17	-0.01
17-May-08	09:30:00	0.15	0.13	0.02	0.13	0.00
17-May-08	09:40:00	0.11	0.09	0.02	0.09	0.00
17-May-08	09:50:00	0.10	0.09	0.01	0.08	-0.01
17-May-08	10:00:00	0.09	0.08	0.01	0.07	-0.01
17-May-08	10:10:00	0.09	0.08	0.01	0.07	-0.01

17-May-08	10:20:00	0.10	0.09	0.01	0.08	-0.01
17-May-08	10:30:00	0.12	0.1	0.02	0.10	0.00
17-May-08	10:40:00	0.13	0.11	0.02	0.11	0.00
17-May-08	10:50:00	0.19	0.18	0.01	0.17	-0.01
17-May-08	11:00:00	0.22	0.2	0.02	0.20	0.00
17-May-08	11:30:00	0.43	0.41	0.02	0.41	0.00
17-May-08	12:00:00	0.65	0.66	-0.01	0.63	-0.03
17-May-08	12:30:00	0.93	0.89	0.04	0.91	0.02
17-May-08	13:00:00	1.26	1.24	0.02	1.24	0.00
17-May-08	13:30:00	1.60	1.58	0.02	1.58	0.00
17-May-08	14:00:00	1.91	1.88	0.03	1.89	0.01
17-May-08	14:30:00	2.18	2.15	0.03	2.16	0.01
17-May-08	15:00:00	2.40	2.38	0.02	2.38	0.00
17-May-08	15:30:00	2.54	2.5	0.04	2.52	0.02
17-May-08	16:00:00	2.60	2.57	0.03	2.58	0.01
17-May-08	16:10:00	2.60	2.57	0.03	2.58	0.01
17-May-08	16:20:00	2.62	2.59	0.03	2.60	0.01
17-May-08	16:30:00	2.61	2.59	0.02	2.59	0.00
17-May-08	16:40:00	2.61	2.58	0.03	2.59	0.01
17-May-08	16:50:00	2.59	2.56	0.03	2.57	0.01
17-May-08	17:00:00	2.57	2.53	0.04	2.55	0.02
17-May-08	17:30:00	2.46	2.44	0.02	2.44	0.00
17-May-08	18:00:00	2.23	2.2	0.03	2.21	0.01
17-May-08	18:30:00	1.92	1.9	0.02	1.90	0.00
17-May-08	19:00:00	1.59	1.56	0.03	1.57	0.01
17-May-08	19:30:00	1.25	1.23	0.02	1.23	0.00
17-May-08	20:00:00	0.93	0.91	0.02	0.91	0.00
			Mean	0.02		0.00

Note:
1. LADS Gauge was placed on the 0.0 of the pole.
2. LADS Gauge observed to be reading 0.02m deep
3. Therefore subtract 0.02m from all gauge depths to get true reading.

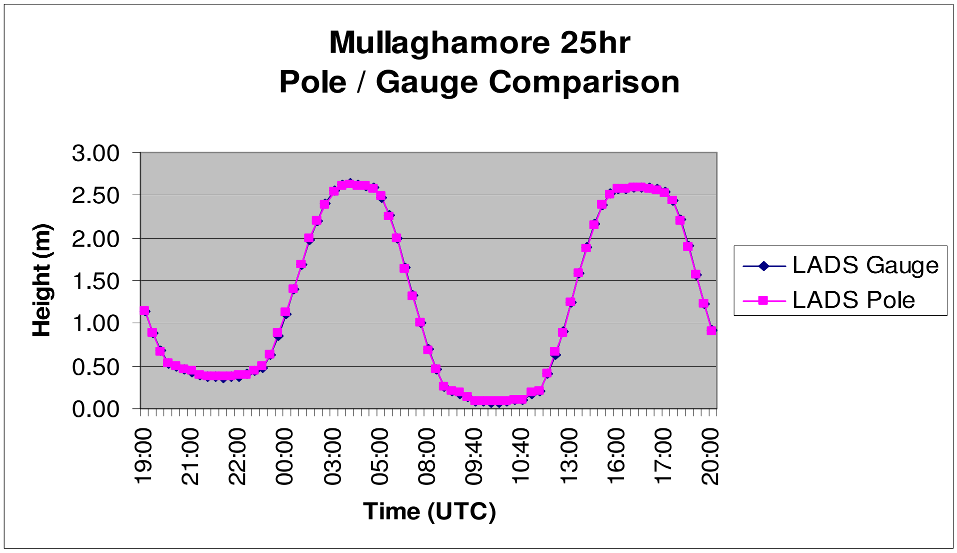

MULLAGHMORE LEVELLING DETAILS

LEVELLING OBSERVATION FORM

Survey	North West Ireland	Date	15May08	Observer	Ryan Wilkins
Unit	Tenix LADS Corp.	Time	1700	Staffman	Huw Thomas
Locality	Mullaghmore			Instrument and No.	5318758
Bench Marks	GSI Benchmark			Staff and No.	

Staff Station	Distance	Stadia Wire	Back Reading	Inter Reading Differences (=/<2 mm)		Forward Reading	Rise (Back>Fwd)	Fall (Back<Fwd)	Reduced Level	Remarks
Benchmark		T	1.741	0.199						
		M	1.542							
	39.6	B	1.345	0.197						
CP	37.7	T	1.545	0.151	0.188	1.656				
		M	1.394			1.468	0.074		0.074	
	30.2	B	1.243	0.151	0.189	1.279				
CP	31.8	T	1.610	0.103	0.159	1.673				
		M	1.507			1.514		-0.120	-0.046	
	20.6	B	1.404	0.103	0.159	1.355				
Tide Pole	16.3	T	1.988	0.084	0.082	1.956				
		M	1.904			1.874		-0.367	-0.413	Placed on 4m mark
	16.7	B	1.821	0.083	0.081	1.793				
CP	17.8	T	1.671	0.158	0.089	1.600				∴ Tide pole zero is 4.413m
		M	1.513			1.511	0.393		-0.020	below BM and 1.462m
	31.6	B	1.355	0.158	0.089	1.422				below Marlin Head
CP	33.1	T	1.666	0.186	0.165	1.584				
		M	1.480			1.419	0.094		0.074	
	37.4	B	1.292	0.188	0.166	1.253				
Benchmark	39.2	T			0.196	1.750				
		M				1.554		-0.074	0.000	
		B			0.196	1.358				
Check Totals	352.0		9.340	1.761	1.759	9.340	0.561	-0.561		Traverse Length (Kms) 0.352
				3.520						Allowable Misclosure 7.1
Height								0.000		Actual Misclosure 0.000

Reduced By	Huw Thomas	Checked By	N.Townsend

MULLAGHMORE DATUM CONNECTION

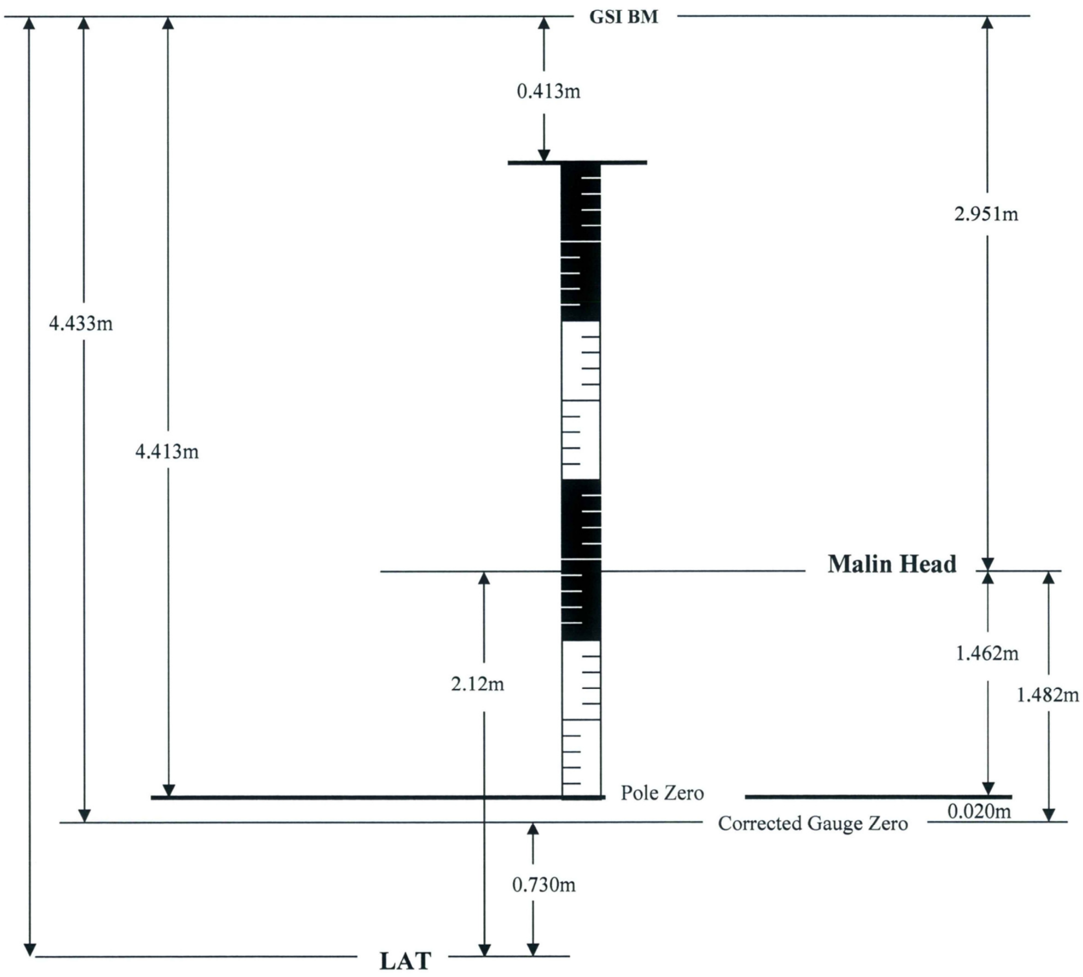

Subtract 1.482m from gauge readings before application for connection to Malin Head. Add 0.730m to gauge readings before application for connection to LAT

ROSSAVEEL

Tenix LADS surveyors installed a Valport 740c tide gauge and tide pole on Rossaveel Pier on 20 May 2008. A 25-hour tide pole / gauge comparison was conducted at the Rossaveel Pier on 20 – 21 May 2008. Tenix LADS surveyors installed a tide pole and gauge on the north western harbour wall and conducted a closed leveling run between the tide pole and a GSI Survey Mark (MO 125). Pole readings were taken every 30 minutes during rising and falling tide periods and at 10 minute intervals over the high and low water periods. These observed values were compared to the Valeport 10 minute logged readings and the height difference between the pole and gauge determined. The analysis also proved that the gauge was logging correctly in both time and range.

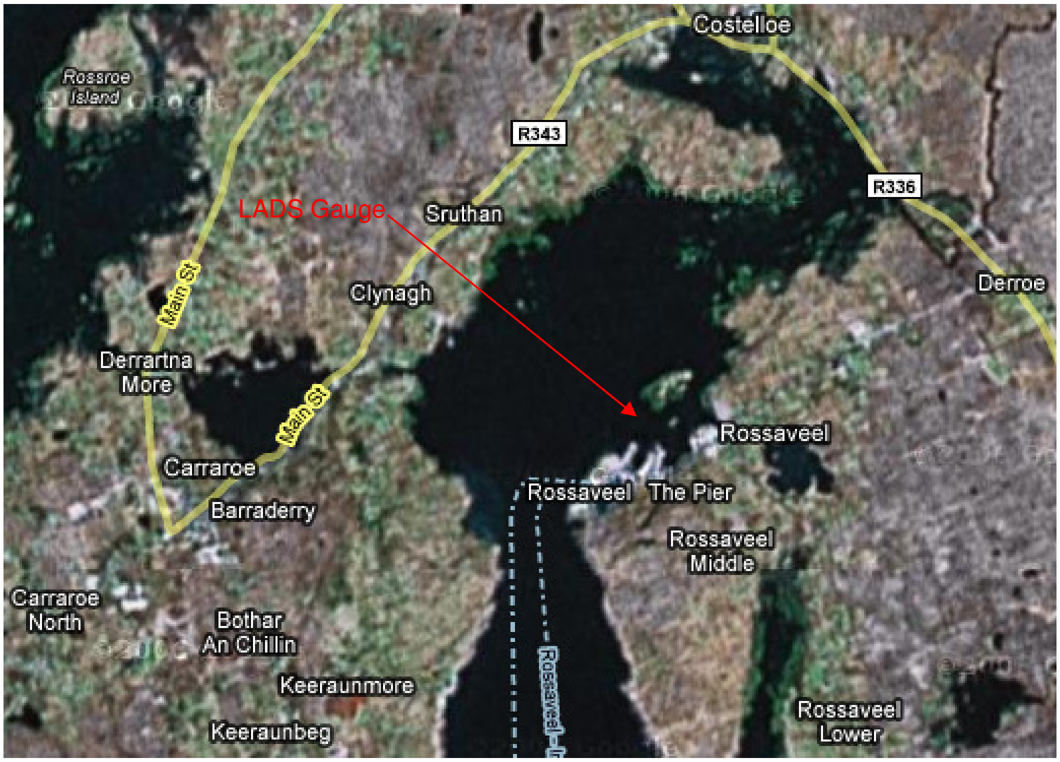

Location of LADS Gauge in Rossaveel

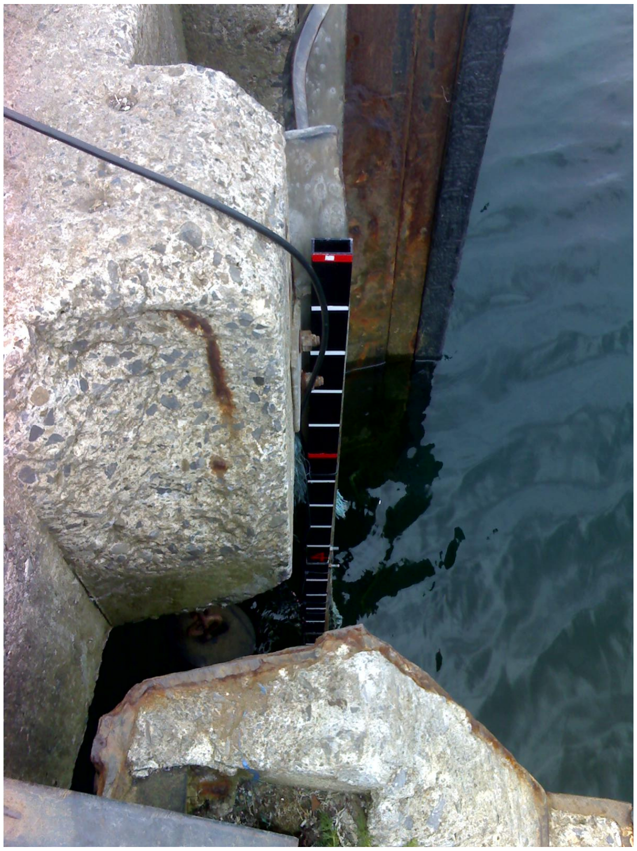

LADS gauge fixed to wharf ladder

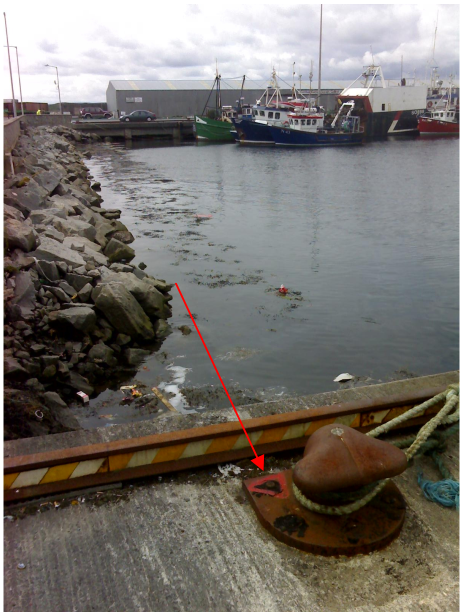

GSI Survey Mark MO 125

ROSSAVEEL SURVEY MARK DESCRIPTION

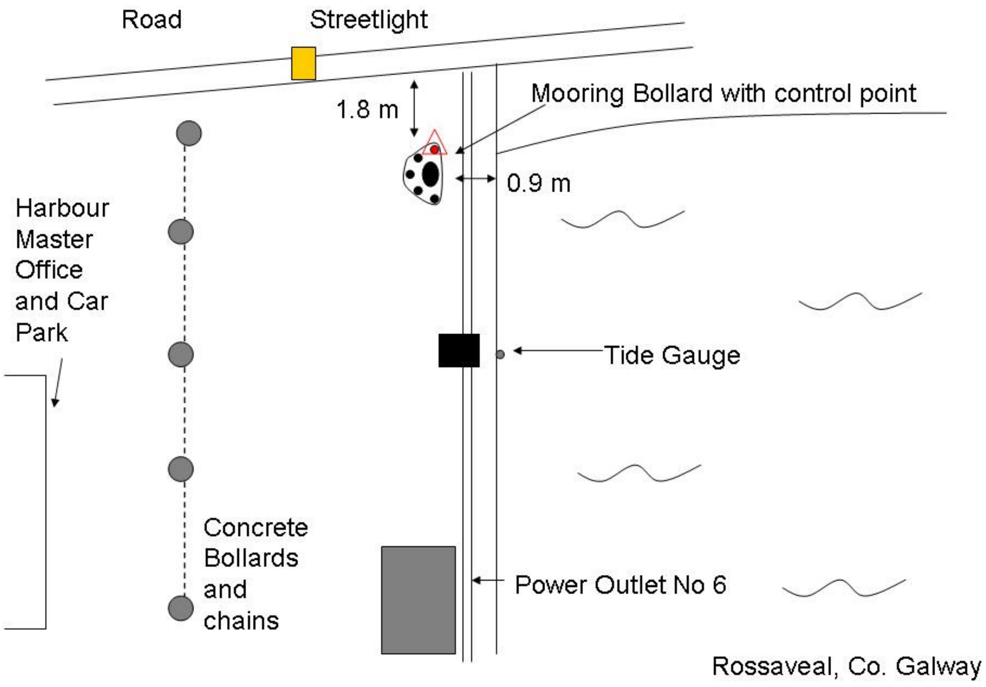

ETRF89_X (m)	ETRF89_Y (m)	ETRF89_Z (m)	E_ITM (m)	N_ITM (m)
3769914.703	-635057.78	5088355.419	495803.786	725104.681
		**LAT (m)**	**E_ING (m)**	**N_ING (m)**
		-2.68	95830.561	225075.25
**ETRF89_LAT**	**ETRF89_LONG**	**ELLIPSOID_HGT(m)**	**HGT_ABOVE_GEOID (m)**	**HGT_ABOVE MALIN HEAD DATUM (m)**
53 15 57.740 N	09 33 42.986 W	60.958	3.502	3.37

ROSSAVEEL 25 HOUR POLE / GAUGE COMPARISON

Date	Time (UTC)	LADS Gauge	LADS Pole	Gauge - Pole	LADS Gauge - 0.02m	Diff. LG - LP
20-May-08	12:00:00	1.97	1.96	0.01	1.95	-0.01
20-May-08	12:30:00	2.22	2.23	-0.01	2.20	-0.03
20-May-08	13:00:00	2.54	2.53	0.01	2.52	-0.01
20-May-08	13:30:00	2.90	2.89	0.01	2.88	-0.01
20-May-08	14:00:00	3.24	3.24	0.00	3.22	-0.02
20-May-08	14:30:00	3.62	3.63	-0.01	3.60	-0.03
20-May-08	15:00:00	3.98	3.98	0.00	3.96	-0.02
20-May-08	15:30:00	4.35	4.33	0.02	4.33	0.00
20-May-08	16:00:00	4.67	4.68	-0.01	4.65	-0.03
20-May-08	16:30:00	4.91	4.88	0.03	4.89	0.01
20-May-08	16:40:00	5.00	4.99	0.01	4.98	-0.01
20-May-08	16:50:00	5.05	5.02	0.03	5.03	0.01
20-May-08	17:00:00	5.09	5.06	0.03	5.07	0.01
20-May-08	17:10:00	5.11	5.09	0.02	5.09	0.00
20-May-08	17:20:00	5.13	5.1	0.03	5.11	0.01
20-May-08	17:30:00	5.13	5.09	0.04	5.11	0.02
20-May-08	17:40:00	5.10	5.06	0.04	5.08	0.02
20-May-08	17:50:00	5.07	5.04	0.03	5.05	0.01
20-May-08	18:00:00	5.03	5	0.03	5.01	0.01
20-May-08	18:30:00	4.79	4.79	0.00	4.77	-0.02
20-May-08	19:00:00	4.46	4.43	0.03	4.44	0.01
20-May-08	19:30:00	4.10	4.08	0.02	4.08	0.00
20-May-08	20:00:00	3.69	3.67	0.02	3.67	0.00
21-May-08	20:30:00	3.30	3.3	0.00	3.28	-0.02
21-May-08	21:00:00	2.94	2.92	0.02	2.92	0.00
21-May-08	21:30:00	2.52	2.5	0.02	2.50	0.00
21-May-08	22:00:00	2.13	2.1	0.03	2.11	0.01
21-May-08	22:30:00	1.85	1.83	0.02	1.83	0.00
21-May-08	23:00:00	1.66	1.64	0.02	1.64	0.00
21-May-08	23:10:00	1.65	1.63	0.02	1.63	0.00
21-May-08	23:20:00	1.63	1.61	0.02	1.61	0.00
21-May-08	23:30:00	1.60	1.59	0.01	1.58	-0.01
21-May-08	23:40:00	1.62	1.61	0.01	1.60	-0.01
21-May-08	23:50:00	1.68	1.68	0.00	1.66	-0.02
21-May-08	00:00:00	1.75	1.74	0.01	1.73	-0.01
21-May-08	00:30:00	1.94	1.96	-0.02	1.92	-0.04
21-May-08	01:00:00	2.25	2.23	0.02	2.23	0.00
21-May-08	01:30:00	2.59	2.59	0.00	2.57	-0.02
21-May-08	02:00:00	2.91	2.91	0.00	2.89	-0.02
21-May-08	02:30:00	3.26	3.26	0.00	3.24	-0.02
21-May-08	03:00:00	3.64	3.64	0.00	3.62	-0.02
21-May-08	03:30:00	3.97	3.99	-0.02	3.95	-0.04
21-May-08	04:00:00	4.31	4.3	0.01	4.29	-0.01
21-May-08	04:30:00	4.58	4.56	0.02	4.56	0.00
21-May-08	04:40:00	4.67	4.64	0.03	4.65	0.01
21-May-08	04:50:00	4.74	4.72	0.02	4.72	0.00
21-May-08	05:00:00	4.79	4.78	0.01	4.77	-0.01

21-May-08	05:10:00	4.83	4.81	0.02	4.81	0.00
21-May-08	05:20:00	4.89	4.87	0.02	4.87	0.00
21-May-08	05:30:00	4.93	4.91	0.02	4.91	0.00
21-May-08	05:40:00	4.95	4.94	0.01	4.93	-0.01
21-May-08	05:50:00	4.95	4.92	0.03	4.93	0.01
21-May-08	06:00:00	4.92	4.89	0.03	4.90	0.01
21-May-08	06:10:00	4.90	4.88	0.02	4.88	0.00
21-May-08	06:20:00	4.88	4.85	0.03	4.86	0.01
21-May-08	06:30:00	4.85	4.83	0.02	4.83	0.00
21-May-08	07:00:00	4.62	4.59	0.03	4.60	0.01
21-May-08	07:30:00	4.31	4.29	0.02	4.29	0.00
21-May-08	08:00:00	3.91	3.89	0.02	3.89	0.00
21-May-08	08:30:00	3.52	3.5	0.02	3.50	0.00
21-May-08	09:00:00	3.13	3.15	-0.02	3.11	-0.04
21-May-08	09:30:00	2.74	2.71	0.03	2.72	0.01
21-May-08	10:00:00	2.41	2.39	0.02	2.39	0.00
21-May-08	10:30:00	2.12	2.1	0.02	2.10	0.00
21-May-08	11:00:00	1.91	1.88	0.03	1.89	0.01
21-May-08	11:10:00	1.85	1.84	0.01	1.83	-0.01
21-May-08	11:20:00	1.83	1.82	0.01	1.81	-0.01
21-May-08	11:30:00	1.82	1.8	0.02	1.80	0.00
21-May-08	11:40:00	1.81	1.79	0.02	1.79	0.00
21-May-08	11:50:00	1.83	1.81	0.02	1.81	0.00
21-May-08	12:00:00	1.87	1.85	0.02	1.85	0.00
21-May-08	12:30:00	2.02	2.02	0.00	2.00	-0.02
21-May-08	13:00:00	2.18	2.19	-0.01	2.16	-0.03
			Mean	0.02		0.00

Note:

1. LADS Gauge was placed on the 0.0 of the pole.
2. LADS Gauge observed to be reading 0.02m deep
3. Therefore subtract 0.02m from all gauge depths to get true reading.

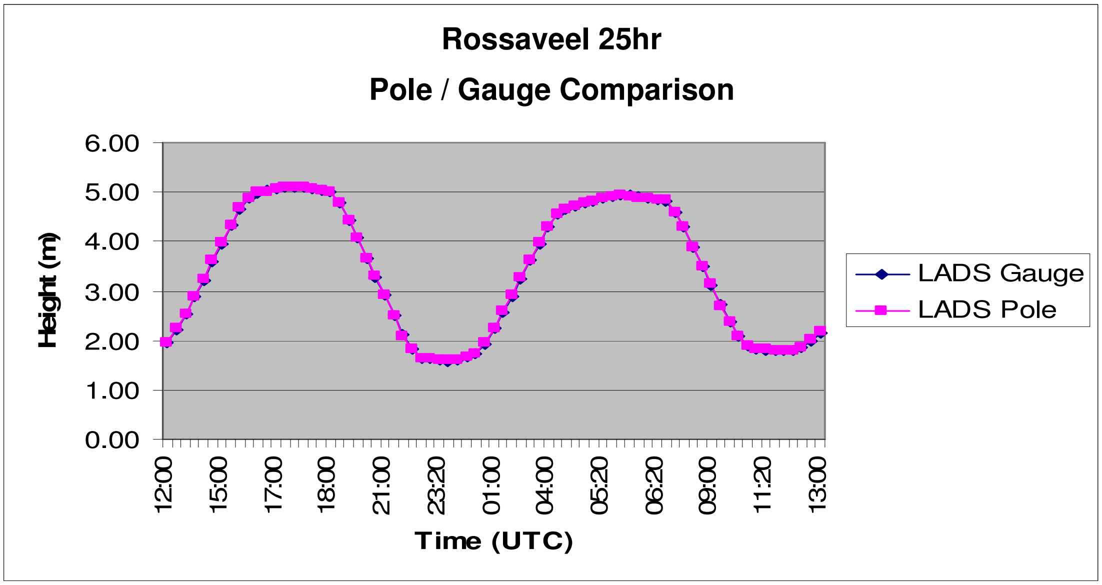

ROSSAVEEL LEVELLING DETAILS

LEVELLING OBSERVATION FORM

Survey	North West Ireland	Date	20May08	Observer	Tyson Hillyard
Unit	Tenix LADS Corp.	Time	1500	Staffman	Ryan Wilkins
Locality	Rossaveel			Instrument and No.	5318758
Bench Marks	M0125			Staff and No.	

Staff Station	Distance	Stadia Wire	Back Reading	Inter Reading Differences (=/<2 mm)		Forward Reading	Rise (Back>Fwd)	Fall (Back<Fwd)	Reduced Level	Remarks
		T	1.672	0.053						
Benchmark		M	1.619							
	10.5	B	1.567	0.052						
	13.6	T	1.630	0.067	0.068	1.572				
CP		M	1.563			1.504	0.115		0.115	
	13.5	B	1.495	0.068	0.068	1.436				
	13.2	T	1.695	0.069	0.067	1.566				
CP		M	1.626			1.499	0.064		0.179	
	13.7	B	1.558	0.068	0.065	1.434				
	14.7	T	1.413	0.073	0.073	1.697				
CP		M	1.340			1.624	0.002		0.181	
	14.7	B	1.266	0.074	0.074	1.550				
	14.3	T	1.655	0.075	0.072	1.679				
CP		M	1.580			1.607		-0.267	-0.086	
	15.0	B	1.505	0.075	0.071	1.536				
	13.5	T	1.614	0.067	0.068	1.639				
CP		M	1.547			1.571	0.009		-0.077	
	13.3	B	1.481	0.066	0.067	1.504				
	13.5	T	1.627	0.072	0.067	1.621				
CP		M	1.555			1.554		-0.007	-0.084	
	14.3	B	1.484	0.071	0.068	1.486				
	13.5	T	1.593	0.053	0.068	1.599				
CP		M	1.540			1.531	0.024		-0.060	
	10.6	B	1.487	0.053	0.067	1.464				
	10.0	T	3.153	0.05	0.050	3.158				
Tide Pole		M	3.103			3.108		-1.568	-1.628	Staff placed at 5 m
	10.0	B	3.053	0.05	0.050	3.058				
	10.5	T	1.620	0.071	0.053	1.589				∴ Tide pole zero is
CP		M	1.549			1.536	1.567		-0.061	6.628m below BM and
	14.4	B	1.476	0.073	0.052	1.484				3.258m below Malin Head
	15.2	T	1.592	0.080	0.076	1.655				
CP		M	1.512			1.579		-0.030	-0.091	
	16.0	B	1.432	0.080	0.076	1.503				
	15.8	T	1.611	0.076	0.079	1.594				
CP		M	1.535			1.515		-0.003	-0.094	
	15.2	B	1.459	0.076	0.079	1.436				
	16.3	T	1.857	0.082	0.082	1.612				
CP		M	1.775			1.530	0.005		-0.089	
	16.3	B	1.694	0.081	0.081	1.449				
	16.3	T	1.584	0.076	0.082	1.569				
CP		M	1.508			1.487	0.288		0.199	
	15.2	B	1.432	0.076	0.081	1.406				
	16.8	T	1.600	0.075	0.084	1.673				
CP		M	1.525			1.589		-0.081	0.118	
	15.0	B	1.450	0.075	0.084	1.505				
	17.0	T			0.085	1.730				
Benchmark		M				1.645		-0.120	-0.002	
		B			0.085	1.560				
Check Totals	421.9		24.877	2.077	2.142	24.879	2.074	-2.076		Traverse Length (Kms) 0.4219
				4.219						Allowable Misclosure 7.8
Height						-0.002		-0.002		Actual Misclosure 2.0

Reduced By	Huw Thomas	Checked By	N.Townsend

LS 4.05 Version 2 dated 16/04/98

ROSSAVEEL DATUM CONNECTION

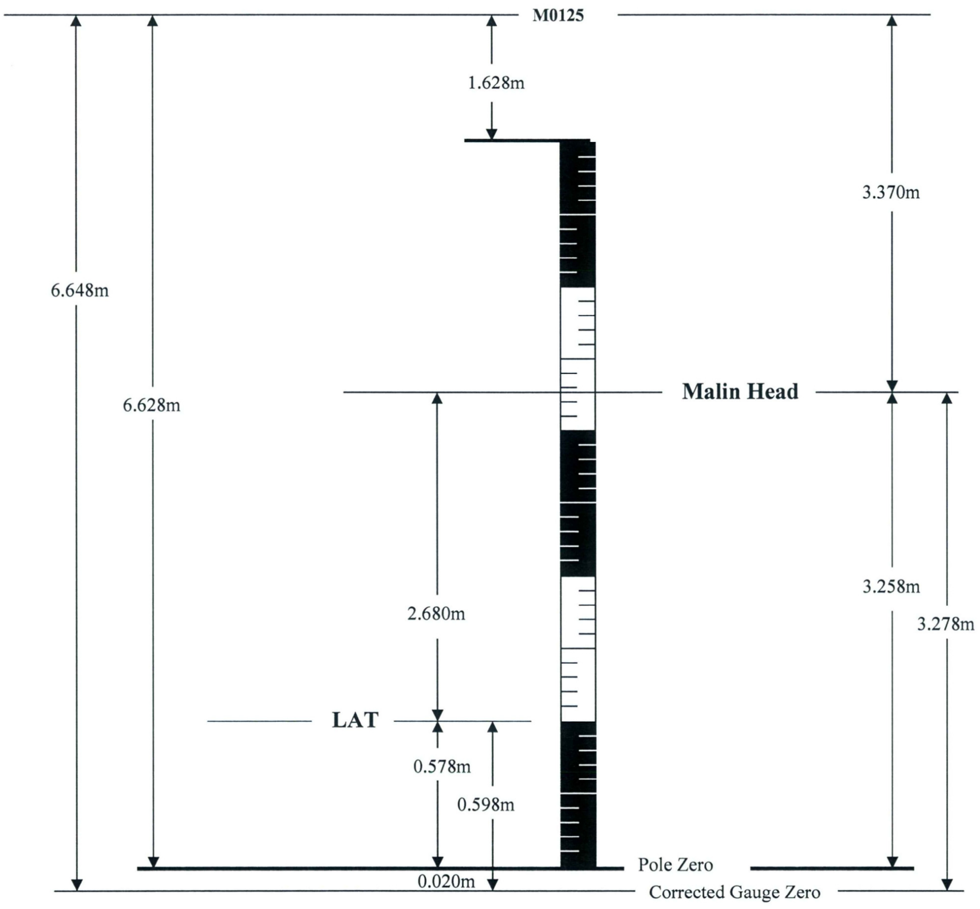

Subtract 3.278m from gauge readings before application for connection to Malin Head. Subtract 0.598 from gauge readings before application for connection to LAT

FENIT HARBOUR

The Valeport 740c tide gauge established at Mullaghmore Harbour was recovered and placed at Fenit Harbour on 29 March 2008. A spot check was conducted to ensure the gauge was operating correctly. LADS surveyors installed a tide pole and gauge on the southern arm of the harbour and conducted a closed leveling run between the tide pole and a GSI Survey Mark (MO 112). Pole readings were taken every 10 minutes over a 3 hour period. These observed values were compared to the Valeport 10 minute logged readings and the height difference between the pole and gauge determined. This analysis confirmed the gauge was operating correctly after being removed from Mullaghmore Harbour.

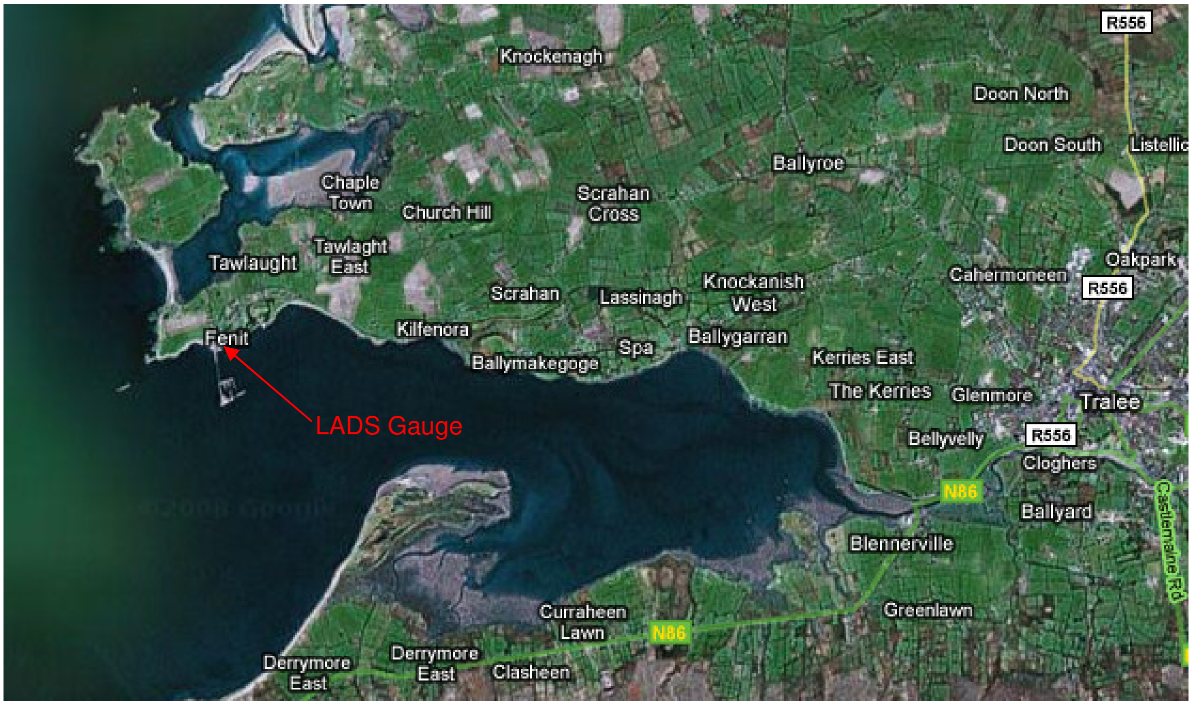

LADS gauge location at Fenit Harbour

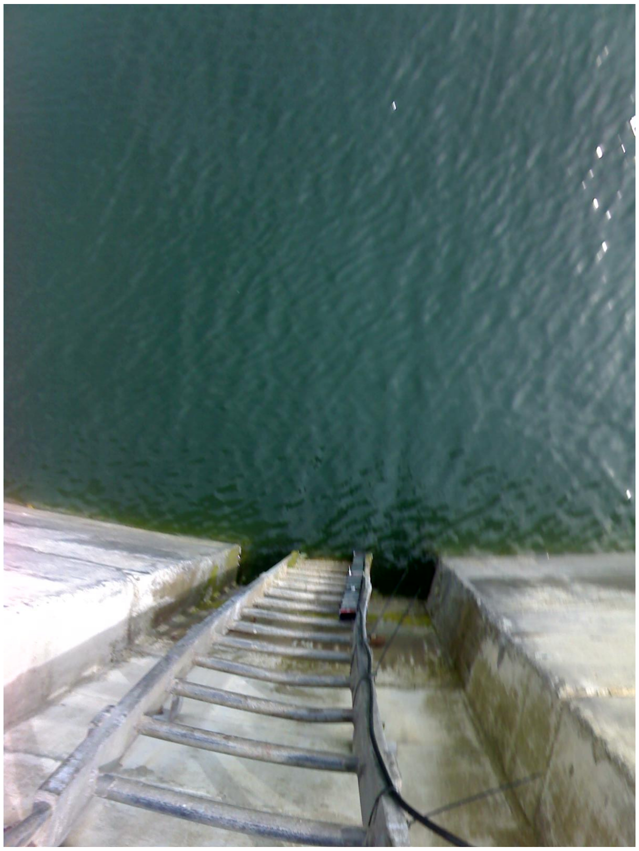

LADS gauge fixed to wharf ladder

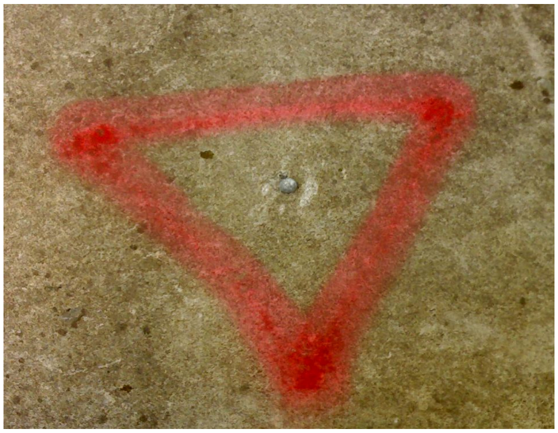

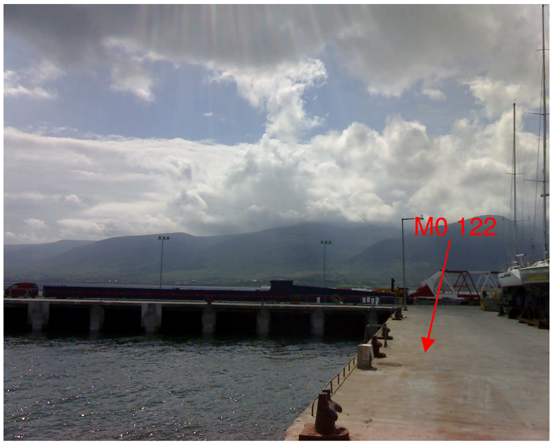

GSI Survey Mark MO 122

FENIT HARBOUR SPOT CHECK

Date	Time (UTC)	LADS Gauge	LADS Pole	Gauge - Pole
29-May-08	10:00	3.236549	3.23	0.01
29-May-08	10:10	3.276825	3.26	0.02
29-May-08	10:20	3.338865	3.32	0.02
29-May-08	10:30	3.394392	3.35	0.04
29-May-08	10:40	3.45073	3.4	0.05
29-May-08	10:50	3.501574	3.45	0.05
29-May-08	11:00	3.549976	3.5	0.05
29-May-08	11:10	3.606306	3.56	0.05
29-May-08	11:20	3.655924	3.61	0.05
29-May-08	11:30	3.673411	3.65	0.02
29-May-08	11:40	3.672191	3.7	-0.03
29-May-08	11:50	3.695779	3.7	0.00
29-May-08	12:00	3.680935	3.63	0.05
29-May-08	12:10	3.668531	3.61	0.06
29-May-08	12:20	3.637623	3.59	0.05
29-May-08	12:30	3.600612	3.58	0.02
29-May-08	12:40	3.546722	3.52	0.03
29-May-08	12:50	3.498117	3.47	0.03
29-May-08	13:00	3.447345	3.42	0.03

Swell effected readings

Average diff without swell affected readings	0.02
Average diff with swell affected readings	0.03

Note:

1. Mullaghmore gauge installed at Fenit Harbour.
2. Gauge observed to be reading 0.02m deep at Mullaghmore.
3. Gauge observed to be reading 0.02m deeper at Fenit Harbour.
4. Gauge operating correctly, subtract 0.02m from gauge depths to get true reading

FENIT HARBOUR SURVEY MARK DESCRIPTION

GSI GPS Control Station Description

**Station Name**	Fenit	**County**	Kerry
**GSI_ID**	M0112		

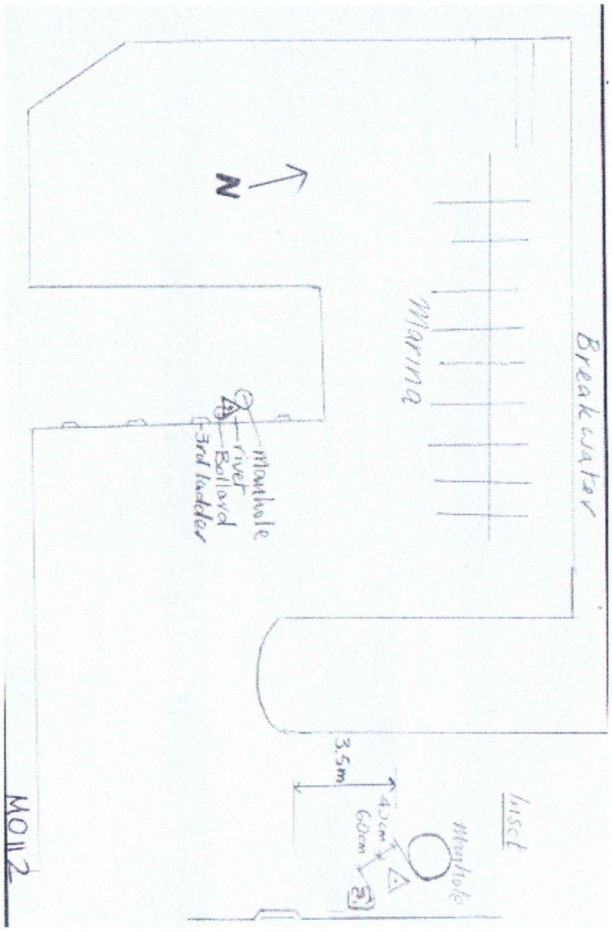

Description of Station Site

Rivet set in central pier, Fenit Harbour. Rivet is 3.5 meters north of 3rd ladder adjacent to manhole.

**Ladder Available?**	Yes
**Does the pier dry out?**	No
**Boats alongside?**	No
**Is the pier exposed?**	No
**Is permission required?**	Yes

Suitability for a tide gauge

GOOD

Local Contact:

Keith Parrott, Kerry CoCo. Tel: 066 7121111

Comments:

There are no comments for this station

Link to photo:

There is no photo available

Control Point Coordinates

TRF89_X (m)	ETRF89_Y (m)	ETRF89_Z (m)	E_ITM (m)	N_ITM (m)
3853460	-669884.5843	5021314.919	472939.985	614813.889

LAT (m)	E_ING (m)	N_ING (m)
-2.41	72962.049	114760.669

ETRF89_LAT	ETRF89_LONG	ELLIPSOID_HGT(m)	HGT_ABOVE_GEOID (m)	HGT_ABOVE MALIN HEAD DATUM (m)
52 16 13.1	9 51 42.235761 W	62.3258	4.101	3.905

FENIT HARBOUR LEVELLING DETAILS

LEVELLING OBSERVATION FORM

Survey	North West Ireland 2008	Date	13Jun08	Observer	Huw Thomas
Unit	Tenix LADS Corp.	Time	1300	Staffman	Ryan Wilkins
Locality	Fenit Harbour			Instrument and No.	5318758
Bench Marks	M0112			Staff and No.	

Staff Station	Distance	Stadia Wire	Back Reading	Inter Reading Differences (=/<2 mm)		Forward Reading	Rise (Back>Fwd)	Fall (Back<Fwd)	Reduced Level	Remarks
PM		T	1.520	0.055						
		M	1.465							
	10.9	B	1.411	0.054						
pole	9.1	T	3.112	0.045	0.046	3.140				
		M	3.067			3.094		-1.629	-1.629	At 5m mark
	9.1	B	3.021	0.046	0.045	3.049				
PM	10.9	T			0.055	1.493				∴Tide pole zero is 6.629m
		M				1.438	1.629			below BM and 2.724m
		B			0.054	1.384				below Malin Head
Check Totals	40.0		4.532	0.200	0.200	4.532	1.629	-1.629		Traverse Length (Kms) 0.04
				0.400						Allowable Misclosure 2.4
Height										Actual Misclosure 0.000

Reduced By	Huw Thomas	Checked By	N.Townsend

FENIT HARBOUR DATUM CONNECTION

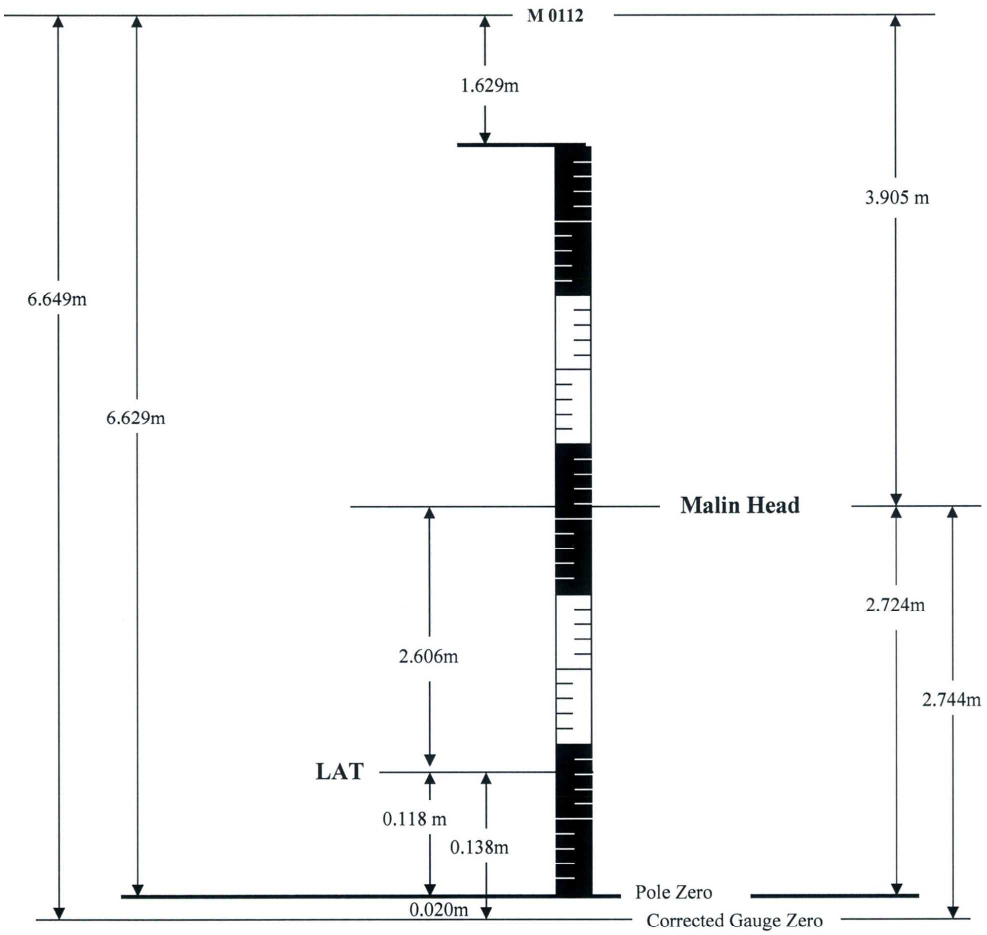

Subtract 2.744m from gauge readings before application for connection to Malin Head. Subtract 0.138 m from gauge readings before application for connection to LAT

DERRY PORT

Tenix LADS surveyors installed a Valport 740c tide gauge and tide pole at Derry Port on 23 May 2008. A 25-hour tide pole / gauge comparison was conducted at the Derry Port in Lisahawley on 23 – 24 May 2008. Tenix LADS surveyors installed a tide pole and gauge adjacent to the Harbour pilot pontoon and conducted a closed leveling run between the tide pole and a GSI Survey Mark (2008_05). Pole readings were taken every 30 minutes during rising and falling tide periods and at 10 minute intervals over the high and low water periods. These observed values were compared to the Valeport 10 minute logged readings and the height difference between the pole and gauge determined. The analysis also proved that the gauge was logging correctly in both time and range.

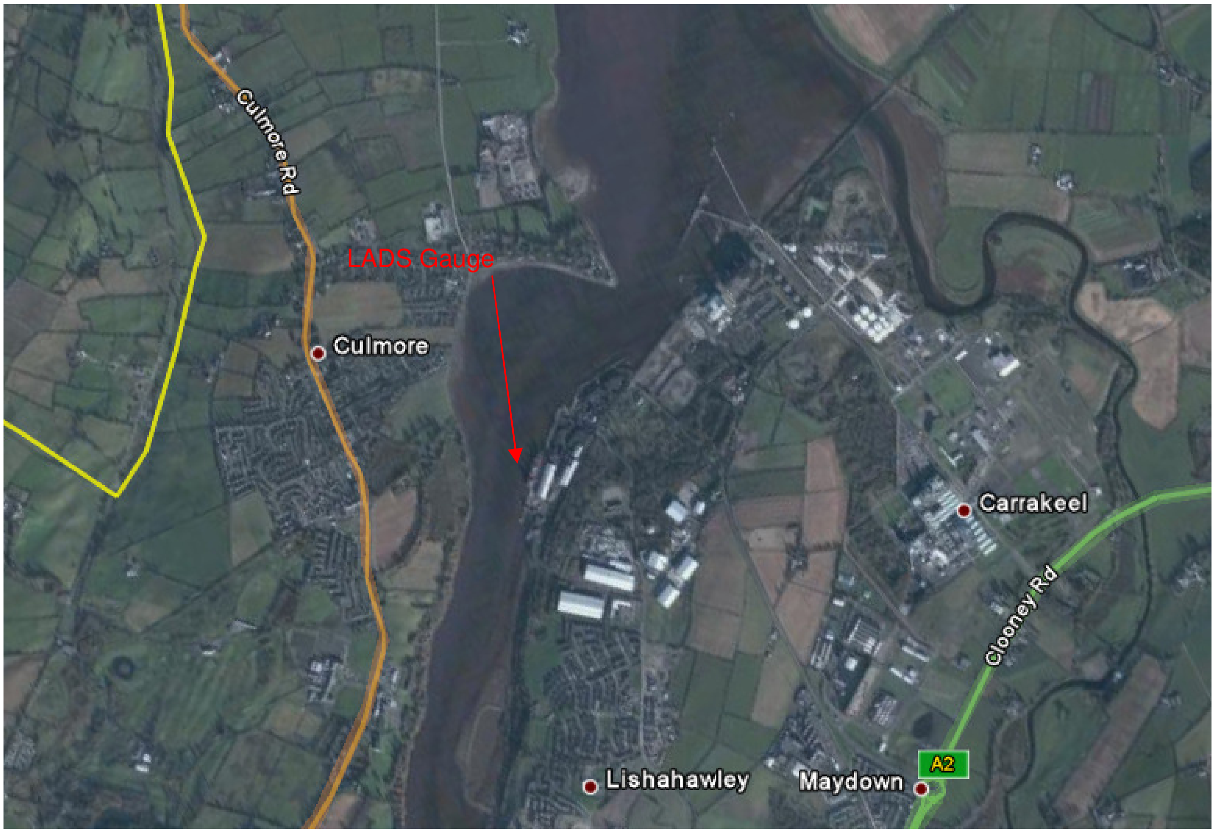

LADS Gauge location at the Derry Port

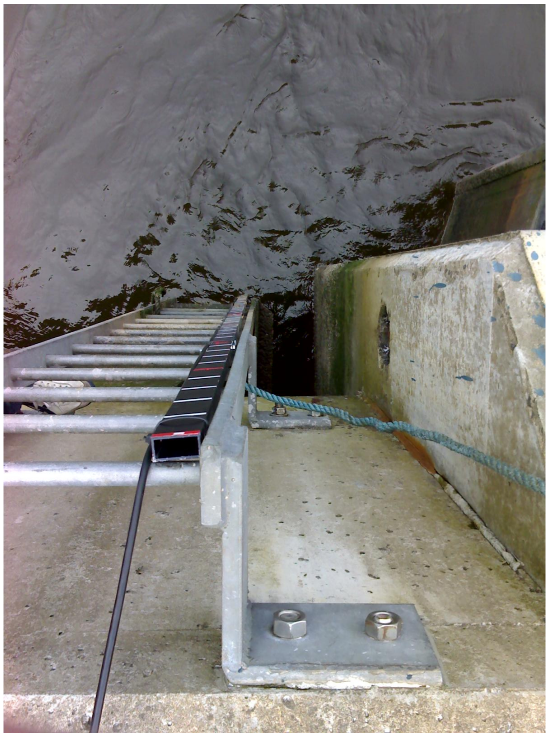

LADS gauge fixed to wharf ladder

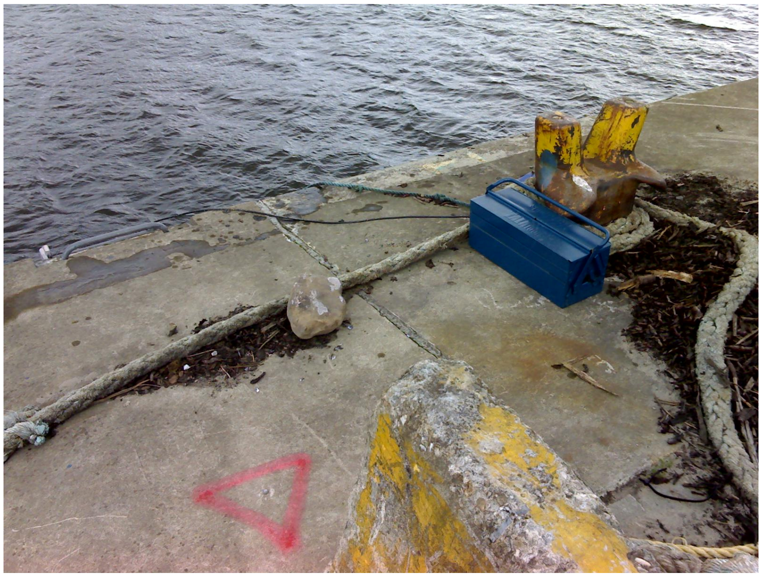

GSI Survey Mark 2008_05

DERRY PORT SURVEY MARK DESCRIPTION

GSI GPS Control Station Description

Station Name	Port of Derry	County	Derry
GSI_ID (year_number)	2008_05		

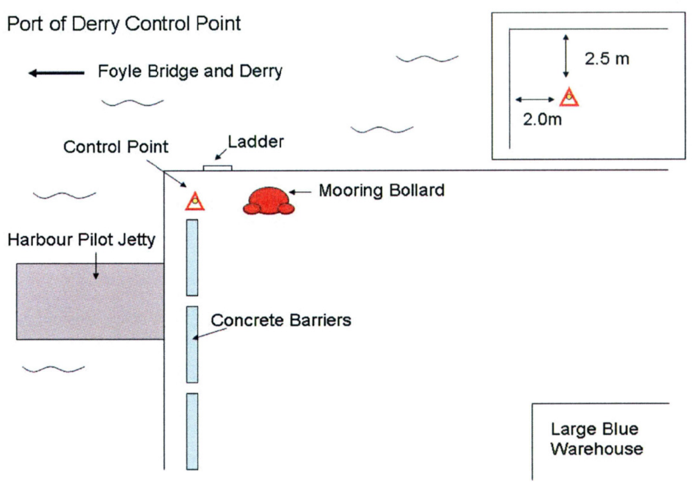

Description of Station Site

Rivet outlined with red triangle set in concrete 2.5 m and 2 m from edge of eastern end of pier in the Port of Derry. Harbour pilot jetty adjacent.

Ladder Available? Yes
Does the pier dry out? No
Boats alongside? Occasionally
Is the pier exposed? No
Is permission required? Yes, HM
Suitability for a tide gauge
GOOD, attached to ladder

Local Contact:

Port of Derry Harbour master

Comments:

On Lough Foyle. Radar gauge from Loughs and Rivers agency adjacent. Sheltered. Locations below are refernced to Northern Irish datum at Belfast Lough as Grid Inquest cannot convert to Republic of Ireland datum at Malin Head.

Link to photo:

There is no photo available

Control Point Coordinates

ETRF89_X (m)	ETRF89_Y (m)	ETRF89_Z (m)		E_NITM (m)	N_NITM (m)
3633541.861	-463261.578	5204003.211		646930.093	921663.265
			LAT	E_IG (NI) (m)	N_IG (NI) (m)
			-0.94 m (at mouth of L. Foyle)	246988.036	421676.929
ETRF89_LAT	ETRF89_LONG	ELLIPSOID_HGT(m)	HGT_ABOVE GEOID (m)		HGT_ABOVE Belfast Lough DATUM (m)
55° 02' 24.97886" N	7° 15' 56.79198"	60.4031 m	3.239		3.159

DERRY PORT 25 HOUR POLE / GAUGE COMPARISON

Date	Time (UTC)	LADS Gauge	LADS Pole	Gauge - Pole	LADS Gauge - 0.12m	Diff. LG - LP
23-May-08	13:30:00	2.299619	2.17	0.13	2.18	0.01
23-May-08	14:00:00	2.138081	2.02	0.12	2.02	0.00
23-May-08	14:10:00	2.098769	1.98	0.12	1.98	0.00
23-May-08	14:20:00	2.053108	1.93	0.12	1.93	0.00
23-May-08	14:30:00	2.000689	1.88	0.12	1.88	0.00
23-May-08	14:40:00	1.964035	1.83	0.13	1.84	0.01
23-May-08	14:50:00	1.933114	1.8	0.13	1.81	0.01
23-May-08	15:00:00	1.890316	1.77	0.12	1.77	0.00
23-May-08	15:10:00	1.856731	1.74	0.12	1.74	0.00
23-May-08	15:20:00	1.83359	1.71	0.12	1.71	0.00
23-May-08	15:30:00	1.811063	1.69	0.12	1.69	0.00
23-May-08	15:40:00	1.805738	1.68	0.13	1.69	0.01
23-May-08	15:50:00	1.800619	1.68	0.12	1.68	0.00
23-May-08	16:00:00	1.810244	1.69	0.12	1.69	0.00
23-May-08	16:10:00	1.846082	1.72	0.13	1.73	0.01
23-May-08	16:20:00	1.901374	1.79	0.11	1.78	-0.01
23-May-08	16:30:00	1.940896	1.82	0.12	1.82	0.00
23-May-08	17:00:00	2.082389	1.96	0.12	1.96	0.00
23-May-08	17:30:00	2.248847	2.13	0.12	2.13	0.00
23-May-08	18:00:00	2.444355	2.33	0.11	2.32	-0.01
23-May-08	18:30:00	2.635335	2.52	0.12	2.52	0.00
23-May-08	19:00:00	2.821585	2.69	0.13	2.70	0.01
23-May-08	19:30:00	3.014156	2.9	0.11	2.89	-0.01
23-May-08	20:00:00	3.187264	3.06	0.13	3.07	0.01
23-May-08	20:30:00	3.351966	3.23	0.12	3.23	0.00
23-May-08	21:00:00	3.476964	3.37	0.11	3.36	-0.01
23-May-08	21:30:00	3.569222	3.45	0.12	3.45	0.00
23-May-08	21:40:00	3.581291	3.48	0.10	3.46	-0.02
23-May-08	21:50:00	3.578222	3.48	0.10	3.46	-0.02
23-May-08	22:00:00	3.566767	3.44	0.13	3.45	0.01
23-May-08	22:10:00	3.54897	3.43	0.12	3.43	0.00
23-May-08	22:20:00	3.529946	3.42	0.11	3.41	-0.01
23-May-08	22:30:00	3.500284	3.37	0.13	3.38	0.01
23-May-08	23:00:00	3.391246	3.28	0.11	3.27	-0.01
23-May-08	23:30:00	3.253147	3.13	0.12	3.13	0.00
24-May-08	00:00:00	3.099894	2.97	0.13	2.98	0.01
24-May-08	00:30:00	2.925138	2.8	0.13	2.81	0.01
24-May-08	01:00:00	2.753637	2.63	0.12	2.63	0.00
24-May-08	01:30:00	2.61978	2.5	0.12	2.50	0.00
24-May-08	02:00:00	2.49635	2.38	0.12	2.38	0.00
24-May-08	02:30:00	2.354076	2.2	0.15	2.23	0.03
24-May-08	03:00:00	2.227759	2.11	0.12	2.11	0.00
24-May-08	03:30:00	2.124773	1.99	0.13	2.00	0.01
24-May-08	04:00:00	2.05147	1.93	0.12	1.93	0.00
24-May-08	04:10:00	2.029356	1.9	0.13	1.91	0.01
24-May-08	04:20:00	2.008675	1.89	0.12	1.89	0.00
24-May-08	04:30:00	1.999665	1.88	0.12	1.88	0.00
24-May-08	04:40:00	2.000279	1.88	0.12	1.88	0.00

24-May-08	04:50:00	2.021575	1.9	0.12	1.90	0.00
24-May-08	05:00:00	2.037956	1.9	0.14	1.92	0.02
24-May-08	05:10:00	2.068465	1.95	0.12	1.95	0.00
24-May-08	05:20:00	2.10614	2	0.11	1.99	-0.01
24-May-08	05:30:00	2.141972	2.04	0.10	2.02	-0.02
24-May-08	06:00:00	2.252941	2.15	0.10	2.13	-0.02
24-May-08	06:30:00	2.388469	2.29	0.10	2.27	-0.02
24-May-08	07:00:00	2.530944	2.42	0.11	2.41	-0.01
24-May-08	07:30:00	2.694488	2.59	0.10	2.57	-0.02
24-May-08	08:00:00	2.846758	2.74	0.11	2.73	-0.01
24-May-08	08:30:00	2.992055	2.88	0.11	2.87	-0.01
24-May-08	09:00:00	3.114218	3	0.11	2.99	-0.01
24-May-08	09:30:00	3.220206	3.09	0.13	3.10	0.01
24-May-08	10:00:00	3.279335	3.16	0.12	3.16	0.00
24-May-08	10:10:00	3.280972	3.14	0.14	3.16	0.02
24-May-08	10:20:00	3.27463	3.16	0.11	3.15	-0.01
24-May-08	10:30:00	3.265014	3.12	0.15	3.15	0.03
24-May-08	10:40:00	3.237188	3.1	0.14	3.12	0.02
24-May-08	10:50:00	3.221025	3.09	0.13	3.10	0.01
24-May-08	11:00:00	3.183582	3.06	0.12	3.06	0.00
24-May-08	11:30:00	3.05856	2.94	0.12	2.94	0.00
24-May-08	12:00:00	2.91245	2.79	0.12	2.79	0.00
24-May-08	12:30:00	2.753433	2.63	0.12	2.63	0.00
24-May-08	13:00:00	2.592761	2.46	0.13	2.47	0.01
24-May-08	13:30:00	2.450496	2.33	0.12	2.33	0.00
24-May-08	14:00:00	2.312927	2.19	0.12	2.19	0.00
24-May-08	14:30:00	2.212608	2.08	0.13	2.09	0.01
			Mean	0.12		0.00

Note:

1. LADS Gauge was placed on the 0.0 of the pole.
2. LADS Gauge observed to be reading 0.12m deep
3. Therefore subtract 0.12m from all gauge depths to get true reading.

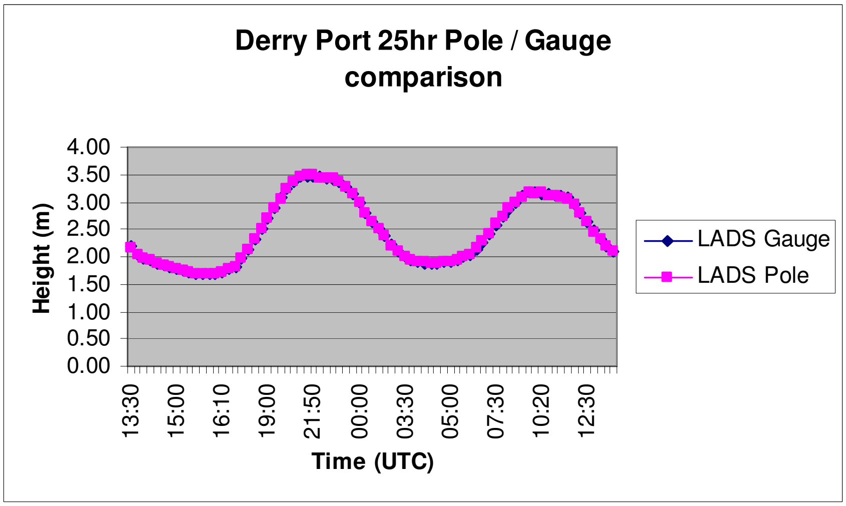

Tenix®

DERRY PORT LEVELLING DETAILS

LEVELLING OBSERVATION FORM

Survey	North West Ireland 2008	Date	23May08	Observer	Huw Thomas
Unit	Tenix LADS Corp.	Time	1400	Staffman	Ryan Wilkins
Locality	Derry Port			Instrument and No.	5318758
Bench Marks	2008_05			Staff and No.	

Staff Station	Distance	Stadia Wire	Back Reading	Inter Reading Differences (=/<2 mm)		Forward Reading	Rise (Back>Fwd)	Fall (Back<Fwd)	Reduced Level	Remarks
PM		T	1.483	0.020						
		M	1.463							
	4.1	B	1.442	0.021						
Tide Pole	4.1	T	2.219	0.020	0.021	2.201				
		M	2.199			2.180		-0.717	-0.717	At 5m mark
	3.9	B	2.180	0.019	0.020	2.160				
PM	3.8	T			0.019	1.501				∴ Tide pole zero is
		M				1.482	0.717			5.717m below BM and
		B			0.019	1.463				2.558m below Belfast Lough
Check Totals	15.9		3.662	0.080<br>0.159	0.079	3.662	0.717	-0.717		Traverse Length (Kms) 0.0159<br>Allowable Misclosure 1.5
Height										Actual Misclosure 0.000

Reduced By	Huw Thomas	Checked By	N.Townsend

DERRY PORT DATUM CONNECTION

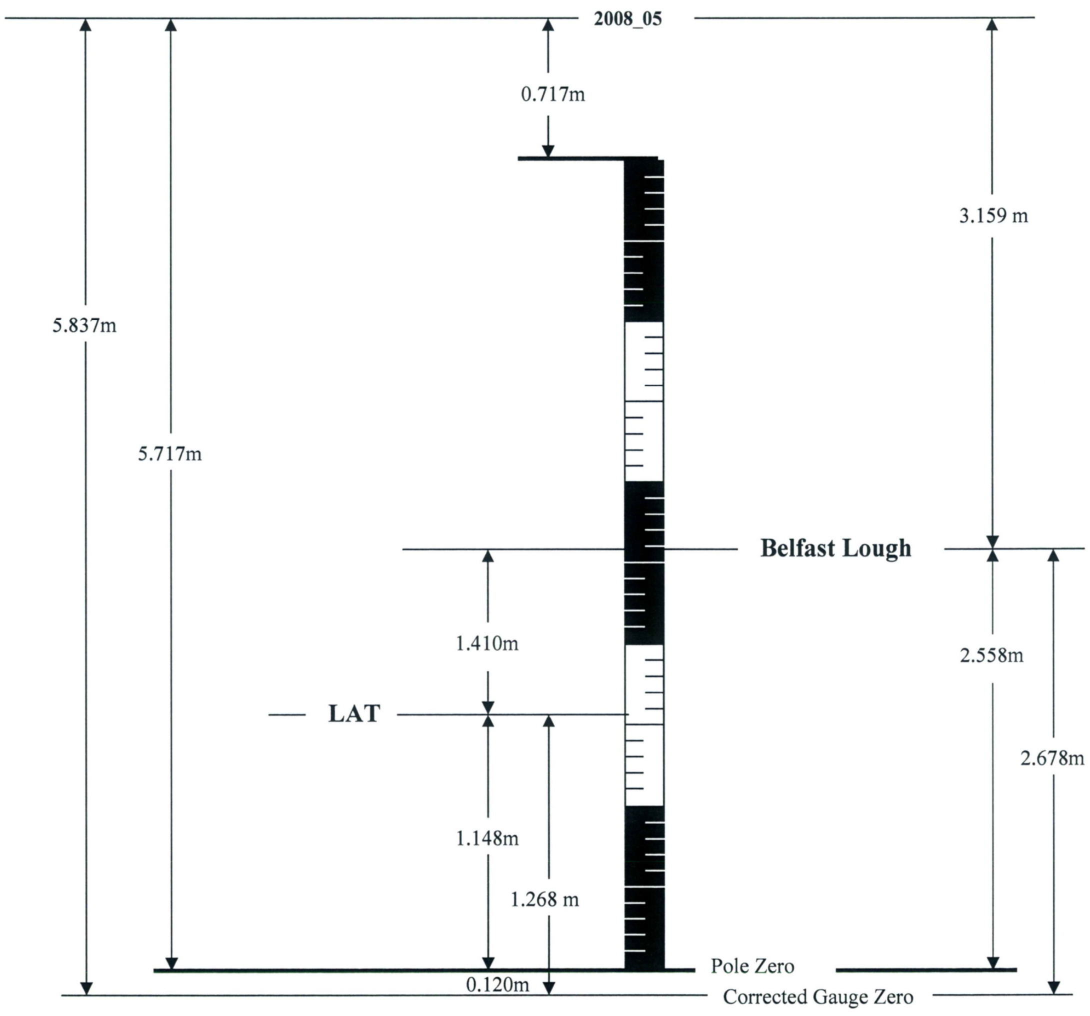

Subtract 2.678m from gauge readings before application for connection to Belfast Lough. Subtract 1.268m from gauge readings before application for connection to LAT

DONEGAL BAY BOTTOM MOUNTED TIDE GAUGE

Two Valeport MIDAS water level recorders were placed in Donegal Bay on 16 May 2008. A local fishing charter based out of Mullaghmore was used to ferry Tenix LADS surveyors to a location approximately 1nm south east of the Mount Charles Pier. The two gauges were secured inside lobster pots and left for a duration of thirty days. A full Tidal analysis was conducted on the BMTG data by the Bureau of Meteorology's National Tidal Center and can be seen in Enclosure 5.
A 25-hour simultaneous logging session was conducted at the Mount Charles Pier on 14 June 2008 to enable the transfer of sounding datum to the gauges. Tenix LADS surveyors installed a tide pole and gauge on the north eastern face of the pier and conducted a closed leveling run between the tide pole and a GSI Survey Mark. Spot checks were conducted to ensure the gauges was operating correctly, it was then left to log for the 25hr period. An analysis was conducted on the data collected from the Mount Charles Pier gauge and the BMTG to transfer sounding datum.

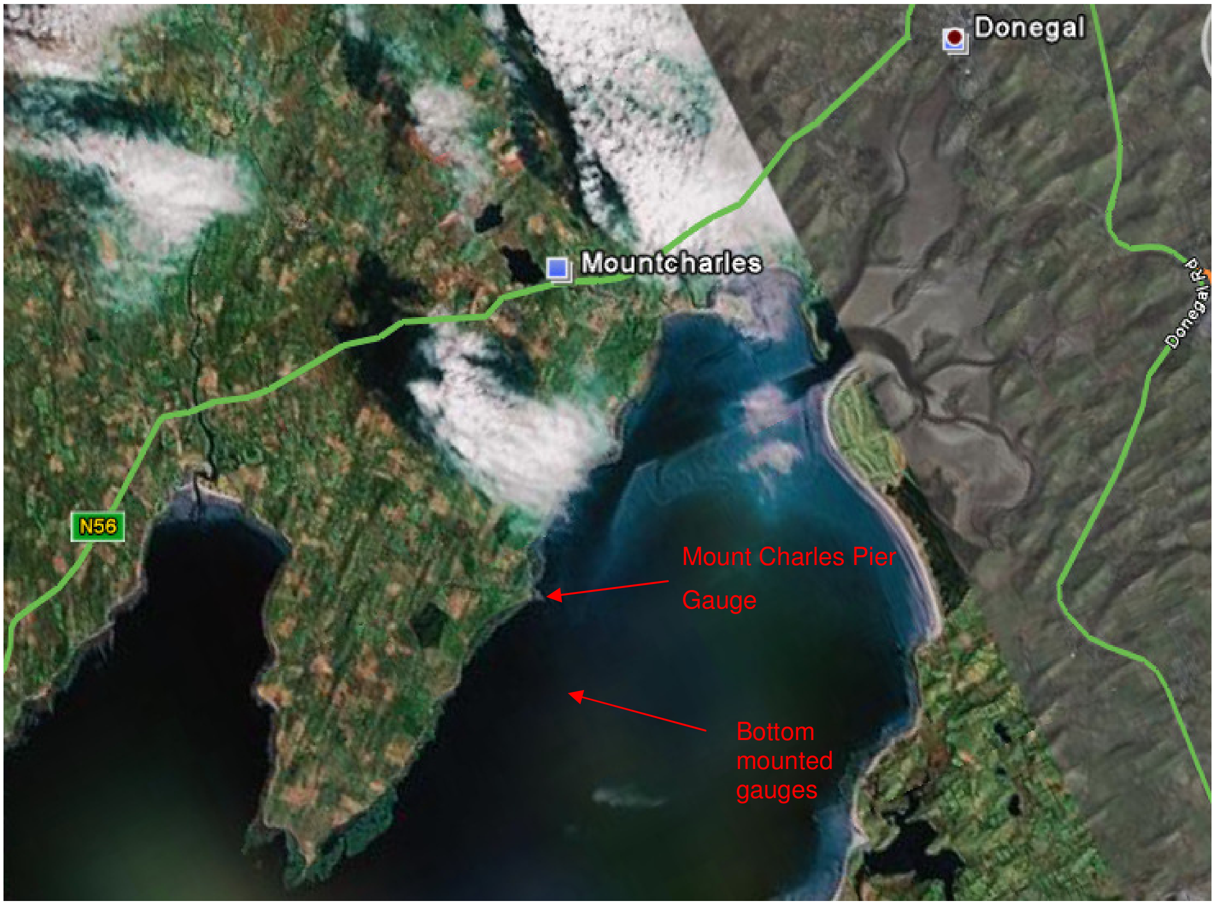

Location of Mount Charles and bottom mounted gauges

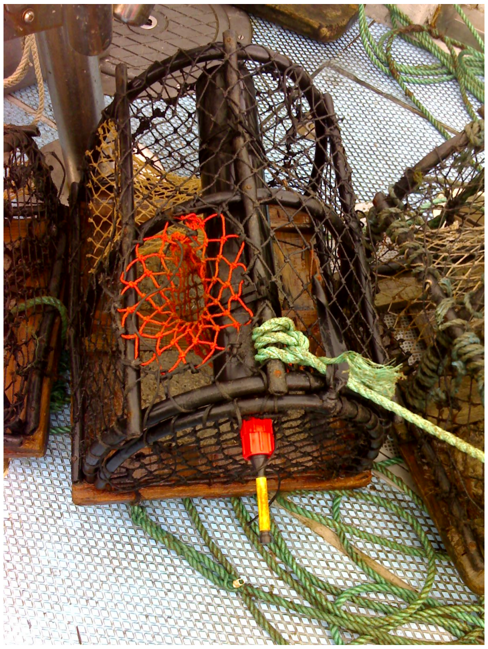

LADS BMTG in lobster pot

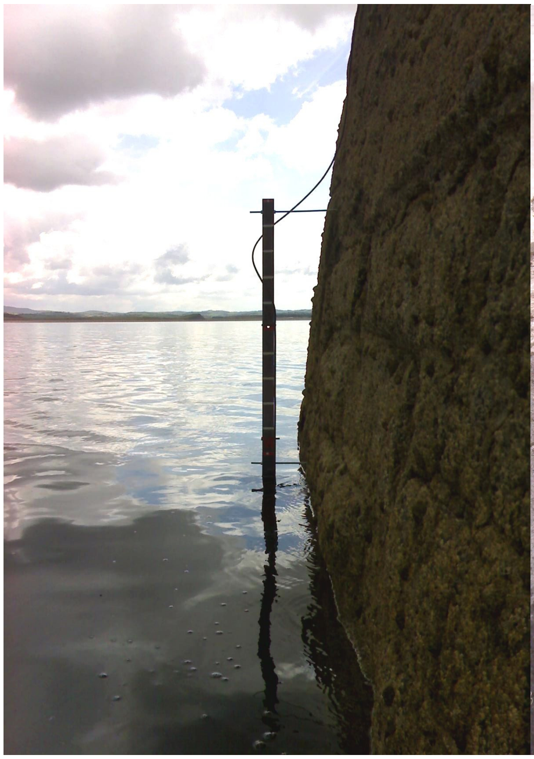

LADS gauge fixed to Mount Charles Pier

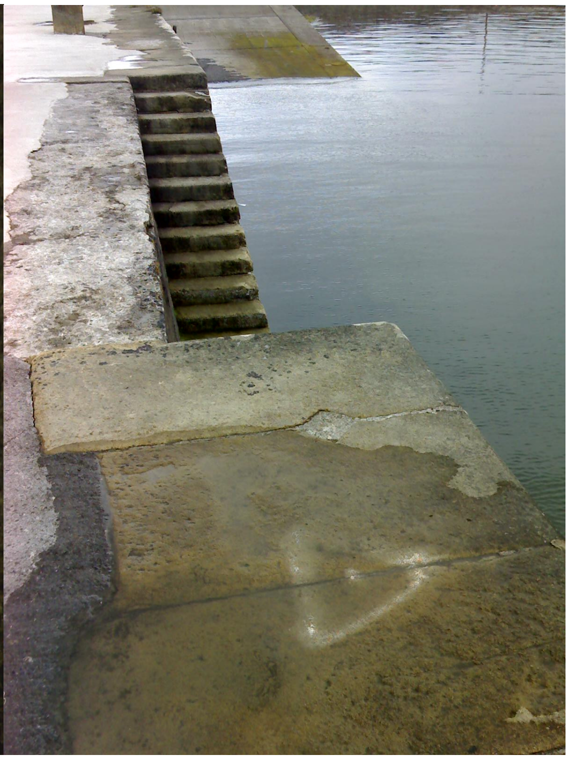

GSI Survey Mark

MOUNT CHARLES SURVEY MARK DESCRIPTION

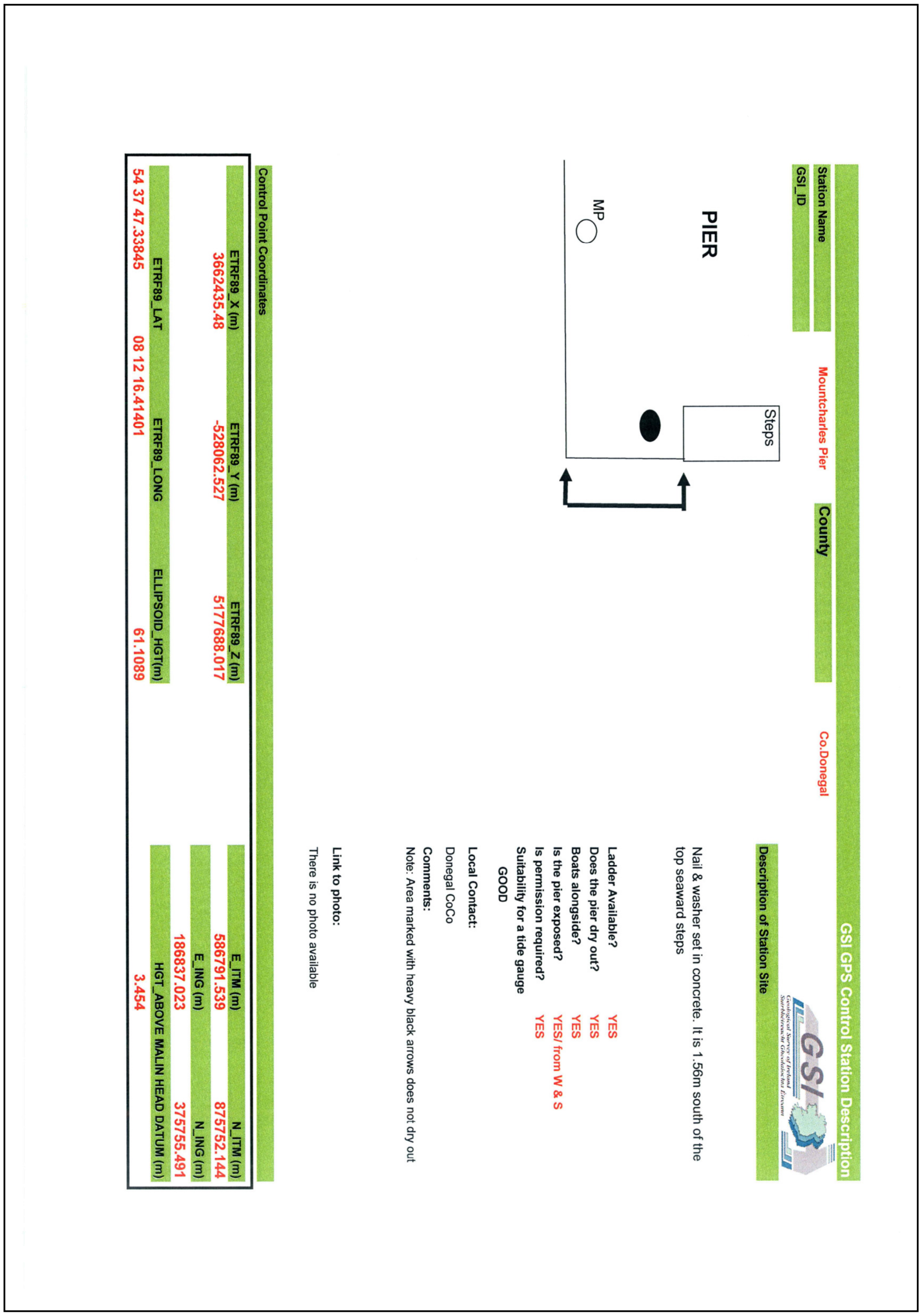

GSI GPS Control Station Description

Station Name	Mountcharles Pier	County	Co.Donegal
GSI_ID			

Description of Station Site

Nail & washer set in concrete. It is 1.56m south of the top seaward steps

**Ladder Available?**	YES
**Does the pier dry out?**	YES
**Boats alongside?**	YES
**Is the pier exposed?**	YES/ from W & S
**Is permission required?**	YES
**Suitability for a tide gauge**	

GOOD

Local Contact:
Donegal CoCo

Comments:
Note: Area marked with heavy black arrows does not dry out

Link to photo:
There is no photo available

Control Point Coordinates

ETRF89_X (m)	ETRF89_Y (m)	ETRF89_Z (m)	E_ITM (m)	N_ITM (m)
3662435.48	-528062.527	5177688.017	586791.539	875752.144

E_ING (m)	N_ING (m)
186837.023	375755.491

ETRF89_LAT	ETRF89_LONG	ELLIPSOID_HGT(m)	HGT_ABOVE MALIN HEAD DATUM (m)
54 37 47.33845	08 12 16.41401	61.1089	3.454

MOUNT CHARLES LEVELLING DETAILS

LEVELLING OBSERVATION FORM

Survey	NW Ireland 2008	Date	15Jun08	Observer	Huw Thomas
Unit	Tenix LADS Corp.	Time	1200	Staffman	Ryan Wilkins
Locality	Mt Charles			Instrument and No.	5318758
Bench Marks	GSI Benchmark			Staff and No.	

Staff Station	Distance	Stadia Wire	Back Reading	Inter Reading Differences (=/<2 mm)		Forward Reading	Rise (Back>Fwd)	Fall (Back<Fwd)	Reduced Level	Remarks
BM		T	1.689	0.032	i					
		M	1.657							
	6.5	B	1.624	0.033						
pole	8.8	T	4.917	0.045	0.044	4.886				
		M	4.872			4.842		-3.185	-3.185	Placed at 2m mark
	9.0	B	4.827	0.045	0.044	4.798				
BM	6.3	T			0.031	1.718				
		M				1.687	3.185			
		B			0.032	1.655				
Check Totals	30.6		6.529	0.155	0.151	6.529	3.185	-3.185		Traverse Length (Kms) 0.0306
				0.306						Allowable Misclosure 2.1
Height										Actual Misclosure 0.000

Reduced By	Huw Thomas	Checked By	N.Townsend

MOUNT CHARLES / BMTG DATUM CONNECTION

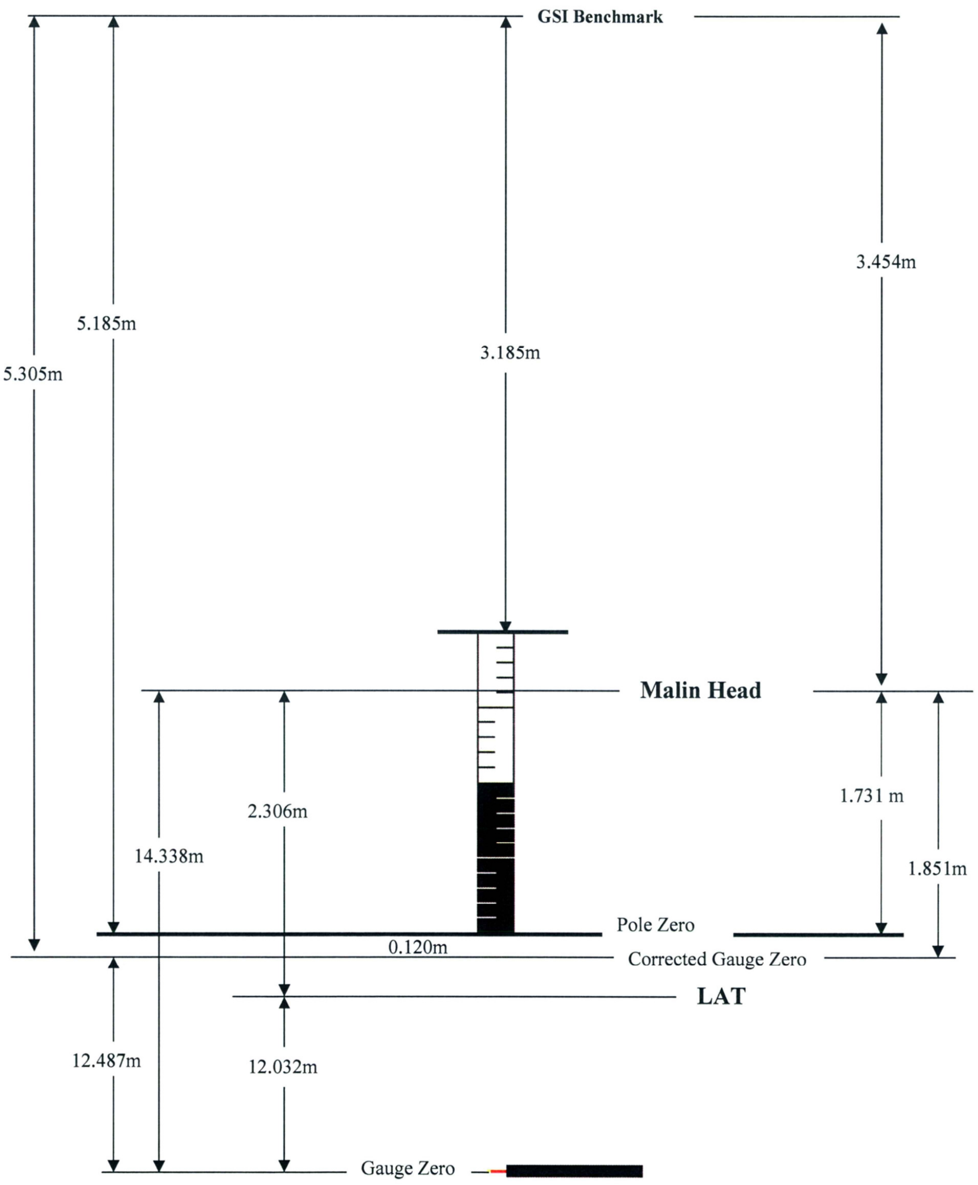

Gauge transferred from Derry Port, 0.120m correction defined during 25hr observation, adopted at Mt Charles. Subtract 1.851m from gauge readings before application for connection to Malin Head. Subtract 14.338m from BMTG readings before application for connection to Malin Head. Subtract 12.032m from BMTG readings before application for connection to LAT

MOUNT CHARLES / BMTG 25HR OBSERVATION

Date	Time (UTC)	Mt Charles Raw	BMTG	Atmospheric Pressure	Donegal BMTG (Corrected for pressure)	Mt Charles / BMTG difference	Mt Charles (Malin Head)	BMTG (Malin Head)	
14-Jun-08	11:40:00	1.448712	24.115	10.15	13.965	12.516288	-0.402288	-0.373	-0.029288
14-Jun-08	11:50:00	1.532038	24.196	10.15	14.046	12.513962	-0.318962	-0.292	-0.026962
14-Jun-08	12:00:00	1.608636	24.275	10.15	14.125	12.516364	-0.242364	-0.213	-0.029364
14-Jun-08	12:10:00	1.690729	24.352	10.15	14.202	12.511271	-0.160271	-0.136	-0.024271
14-Jun-08	12:20:00	1.768538	24.431	10.15	14.281	12.512462	-0.082462	-0.057	-0.025462
14-Jun-08	12:30:00	1.848582	24.515	10.15	14.365	12.516418	-0.002418	0.027	-0.029418
14-Jun-08	12:40:00	1.937582	24.601	10.15	14.451	12.513418	0.086582	0.113	-0.026418
14-Jun-08	12:50:00	2.024334	24.677	10.15	14.527	12.502666	0.173334	0.189	-0.015666
14-Jun-08	13:00:00	2.09703	24.743	10.15	14.593	12.49597	0.24603	0.255	-0.00897
14-Jun-08	13:10:00	2.155876	24.807	10.15	14.657	12.501124	0.304876	0.319	-0.014124
14-Jun-08	13:20:00	2.2137	24.863	10.15	14.713	12.4993	0.3627	0.375	-0.0123
14-Jun-08	13:30:00	2.273762	24.92	10.15	14.77	12.496238	0.422762	0.432	-0.009238
14-Jun-08	13:40:00	2.332395	24.97	10.15	14.82	12.487605	0.481395	0.482	-0.000605
14-Jun-08	13:50:00	2.377182	25.009	10.15	14.859	12.481818	0.526182	0.521	0.005182
14-Jun-08	14:00:00	2.400796	25.038	10.15	14.888	12.487204	0.549796	0.55	-0.000204
14-Jun-08	14:10:00	2.43581	25.08	10.15	14.93	12.49419	0.58481	0.592	-0.00719
14-Jun-08	14:20:00	2.486699	25.127	10.15	14.977	12.490301	0.635699	0.639	-0.003301
14-Jun-08	14:30:00	2.532905	25.161	10.15	15.011	12.478095	0.681905	0.673	0.008905
14-Jun-08	14:40:00	2.539825	25.177	10.15	15.027	12.487175	0.688825	0.689	-0.000175
14-Jun-08	14:50:00	2.545525	25.18	10.15	15.03	12.484475	0.694525	0.692	0.002525
14-Jun-08	15:00:00	2.548985	25.18	10.15	15.03	12.481015	0.697985	0.692	0.005985
14-Jun-08	15:10:00	2.54756	25.177	10.15	15.027	12.47944	0.69656	0.689	0.00756
14-Jun-08	15:20:00	2.535958	25.171	10.15	15.021	12.485042	0.684958	0.683	0.001958
14-Jun-08	15:30:00	2.535144	25.171	10.15	15.021	12.485856	0.684144	0.683	0.001144
14-Jun-08	15:40:00	2.535754	25.167	10.15	15.017	12.481246	0.684754	0.679	0.005754

14-Jun-08	15:50:00	2.523949	25.152	10.15	15.002	12.478051	0.672949	0.664	0.008949
14-Jun-08	16:00:00	2.483646	25.121	10.15	14.971	12.487354	0.632646	0.633	-0.000354
14-Jun-08	16:10:00	2.446598	25.082	10.15	14.932	12.485402	0.595598	0.594	0.001598
14-Jun-08	16:20:00	2.410364	25.041	10.15	14.891	12.480636	0.559364	0.553	0.006364
14-Jun-08	16:30:00	2.359471	24.986	10.15	14.836	12.476529	0.508471	0.498	0.010471
14-Jun-08	16:40:00	2.305929	24.924	10.15	14.774	12.468071	0.454929	0.436	0.018929
14-Jun-08	16:50:00	2.241594	24.862	10.15	14.712	12.470406	0.390594	0.374	0.016594
14-Jun-08	17:00:00	2.178069	24.796	10.15	14.646	12.467931	0.327069	0.308	0.019069
14-Jun-08	17:10:00	2.104564	24.72	10.15	14.57	12.465436	0.253564	0.232	0.021564
14-Jun-08	17:20:00	2.018836	24.634	10.15	14.484	12.465164	0.167836	0.146	0.021836
14-Jun-08	17:30:00	1.936971	24.553	10.15	14.403	12.466029	0.085971	0.065	0.020971
14-Jun-08	17:40:00	1.853063	24.473	10.15	14.323	12.469937	0.002063	-0.015	0.017063
14-Jun-08	17:50:00	1.783814	24.392	10.15	14.242	12.458186	-0.067186	-0.096	0.028814
14-Jun-08	18:00:00	1.697247	24.3	10.15	14.15	12.452753	-0.153753	-0.188	0.034247
14-Jun-08	18:10:00	1.604562	24.207	10.15	14.057	12.452438	-0.246438	-0.281	0.034562
14-Jun-08	18:20:00	1.518796	24.125	10.15	13.975	12.456204	-0.332204	-0.363	0.030796
14-Jun-08	18:30:00	1.442804	24.051	10.15	13.901	12.458196	-0.408196	-0.437	0.028804
14-Jun-08	18:40:00	1.376179	23.987	10.15	13.837	12.460821	-0.474821	-0.501	0.026179
14-Jun-08	18:50:00	1.305474	23.924	10.15	13.774	12.468526	-0.545526	-0.564	0.018474
14-Jun-08	19:00:00	1.24149	23.866	10.15	13.716	12.47451	-0.60951	-0.622	0.01249
14-Jun-08	19:10:00	1.180152	23.812	10.15	13.662	12.481848	-0.670848	-0.676	0.005152
14-Jun-08	19:20:00	1.134707	23.77	10.15	13.62	12.485293	-0.716293	-0.718	0.001707
14-Jun-08	19:30:00	1.093743	23.734	10.15	13.584	12.490257	-0.757257	-0.754	-0.003257
14-Jun-08	19:40:00	1.058077	23.69	10.15	13.54	12.481923	-0.792923	-0.798	0.005077
14-Jun-08	19:50:00	1.014665	23.643	10.15	13.493	12.478335	-0.836335	-0.845	0.008665
14-Jun-08	20:00:00	0.9698243	23.598	10.15	13.448	12.4781757	-0.8811757	-0.89	0.0088243
14-Jun-08	20:10:00	0.9315045	23.549	10.15	13.399	12.4674955	-0.9194955	-0.939	0.0195045
14-Jun-08	20:20:00	0.8895144	23.512	10.15	13.362	12.4724856	-0.9614856	-0.976	0.0145144
14-Jun-08	20:30:00	0.8513958	23.48	10.15	13.33	12.4786042	-0.9996042	-1.008	0.0083958
14-Jun-08	20:40:00	0.8279535	23.471	10.15	13.321	12.4930465	-1.0230465	-1.017	-0.0060465
14-Jun-08	20:50:00	0.817557	23.474	10.15	13.324	12.506443	-1.033443	-1.014	-0.019443
14-Jun-08	21:00:00	0.8179648	23.479	10.15	13.329	12.5110352	-1.0330352	-1.009	-0.0240352
14-Jun-08	21:10:00	0.8224494	23.483	10.15	13.333	12.5105506	-1.0285506	-1.005	-0.0235506

14-Jun-08	21:20:00	0.8369229	23.485	10.15	13.335	12.4980771	-1.0140771	-1.003	-0.0110771
14-Jun-08	21:30:00	0.8558805	23.492	10.15	13.342	12.4861195	-0.9951195	-0.996	0.0008805
14-Jun-08	21:40:00	0.8734112	23.499	10.15	13.349	12.4755888	-0.9775888	-0.989	0.0114112
14-Jun-08	21:50:00	0.8893106	23.515	10.15	13.365	12.4756894	-0.9616894	-0.973	0.0113106
14-Jun-08	22:00:00	0.9125481	23.544	10.15	13.394	12.4814519	-0.9384519	-0.944	0.0055481
14-Jun-08	22:10:00	0.9474034	23.585	10.15	13.435	12.4875966	-0.9035966	-0.903	-0.0005966
14-Jun-08	22:20:00	0.9802194	23.623	10.15	13.473	12.4927806	-0.8707806	-0.865	-0.0057806
14-Jun-08	22:30:00	1.016703	23.669	10.15	13.519	12.502297	-0.834297	-0.819	-0.015297
14-Jun-08	22:40:00	1.064599	23.726	10.14	13.586	12.521401	-0.786401	-0.752	-0.034401
14-Jun-08	22:50:00	1.128389	23.786	10.14	13.646	12.517611	-0.722611	-0.692	-0.030611
14-Jun-08	23:00:00	1.197066	23.855	10.14	13.715	12.517934	-0.653934	-0.623	-0.030934
14-Jun-08	23:10:00	1.264313	23.926	10.14	13.786	12.521687	-0.586687	-0.552	-0.034687
14-Jun-08	23:20:00	1.342966	23.993	10.14	13.853	12.510034	-0.508034	-0.485	-0.023034
14-Jun-08	23:30:00	1.41265	24.058	10.14	13.918	12.50535	-0.43835	-0.42	-0.01835
14-Jun-08	23:40:00	1.481717	24.133	10.14	13.993	12.511283	-0.369283	-0.345	-0.024283
14-Jun-08	23:50:00	1.560152	24.214	10.14	14.074	12.513848	-0.290848	-0.264	-0.026848
15-Jun-08	00:00:00	1.648155	24.306	10.14	14.166	12.517845	-0.202845	-0.172	-0.030845
15-Jun-08	00:10:00	1.736152	24.396	10.14	14.256	12.519848	-0.114848	-0.082	-0.032848
15-Jun-08	00:20:00	1.823531	24.473	10.14	14.333	12.509469	-0.027469	-0.005	-0.022469
15-Jun-08	00:30:00	1.90072	24.546	10.14	14.406	12.50528	0.04972	0.068	-0.01828
15-Jun-08	00:40:00	1.968129	24.61	10.14	14.47	12.501871	0.117129	0.132	-0.014871
15-Jun-08	00:50:00	2.040421	24.672	10.14	14.532	12.491579	0.189421	0.194	-0.004579
15-Jun-08	01:00:00	2.100695	24.727	10.14	14.587	12.486305	0.249695	0.249	0.000695
15-Jun-08	01:10:00	2.150175	24.781	10.14	14.641	12.490825	0.299175	0.303	-0.003825
15-Jun-08	01:20:00	2.204742	24.837	10.14	14.697	12.492258	0.353742	0.359	-0.005258
15-Jun-08	01:30:00	2.26175	24.883	10.14	14.743	12.48125	0.41075	0.405	0.00575
15-Jun-08	01:40:00	2.304504	24.927	10.14	14.787	12.482496	0.453504	0.449	0.004504
15-Jun-08	01:50:00	2.347663	24.973	10.14	14.833	12.485337	0.496663	0.495	0.001663
15-Jun-08	02:00:00	2.38105	25.008	10.14	14.868	12.48695	0.53005	0.53	5E-05
15-Jun-08	02:10:00	2.419321	25.049	10.14	14.909	12.489679	0.568321	0.571	-0.002679
15-Jun-08	02:20:00	2.4696	25.094	10.14	14.954	12.4844	0.6186	0.616	0.0026
15-Jun-08	02:30:00	2.515196	25.137	10.14	14.997	12.481804	0.664196	0.659	0.005196
15-Jun-08	02:40:00	2.551427	25.174	10.13	15.044	12.492573	0.700427	0.706	-0.005573

15-Jun-08	02:50:00	2.573613	25.201	10.13	15.071	12.497387	0.722613	0.733	-0.010387
15-Jun-08	03:00:00	2.60394	25.227	10.13	15.097	12.49306	0.75294	0.759	-0.00606
15-Jun-08	03:10:00	2.632841	25.247	10.13	15.117	12.484159	0.781841	0.779	0.002841
15-Jun-08	03:20:00	2.641797	25.261	10.13	15.131	12.489203	0.790797	0.793	-0.002203
15-Jun-08	03:30:00	2.649938	25.266	10.13	15.136	12.486062	0.798938	0.798	0.000938
15-Jun-08	03:40:00	2.644646	25.256	10.13	15.126	12.481354	0.793646	0.788	0.005646
15-Jun-08	03:50:00	2.630603	25.247	10.13	15.117	12.486397	0.779603	0.779	0.000603
15-Jun-08	04:00:00	2.611471	25.22	10.13	15.09	12.478529	0.760471	0.752	0.008471
15-Jun-08	04:10:00	2.584197	25.188	10.13	15.058	12.473803	0.733197	0.72	0.013197
15-Jun-08	04:20:00	2.544507	25.156	10.13	15.026	12.481493	0.693507	0.688	0.005507
15-Jun-08	04:30:00	2.511736	25.11	10.13	14.98	12.468264	0.660736	0.642	0.018736
15-Jun-08	04:40:00	2.44843	25.054	10.13	14.924	12.47557	0.59743	0.586	0.01143
15-Jun-08	04:50:00	2.375146	24.979	10.13	14.849	12.473854	0.524146	0.511	0.013146
15-Jun-08	05:00:00	2.305521	24.902	10.13	14.772	12.466479	0.454521	0.434	0.020521
15-Jun-08	05:10:00	2.22836	24.823	10.13	14.693	12.46464	0.37736	0.355	0.02236
15-Jun-08	05:20:00	2.140808	24.735	10.13	14.605	12.464192	0.289808	0.267	0.022808
15-Jun-08	05:30:00	2.048363	24.639	10.13	14.509	12.460637	0.197363	0.171	0.026363
15-Jun-08	05:40:00	1.962223	24.546	10.13	14.416	12.453777	0.111223	0.078	0.033223
15-Jun-08	05:50:00	1.86793	24.454	10.13	14.324	12.45607	0.01693	-0.014	0.03093
15-Jun-08	06:00:00	1.778722	24.365	10.13	14.235	12.456278	-0.072278	-0.103	0.030722
15-Jun-08	06:10:00	1.692969	24.276	10.13	14.146	12.453031	-0.158031	-0.192	0.033969
15-Jun-08	06:20:00	1.608025	24.197	10.13	14.067	12.458975	-0.242975	-0.271	0.028025
15-Jun-08	06:30:00	1.53265	24.129	10.13	13.999	12.46635	-0.31835	-0.339	0.02065
15-Jun-08	06:40:00	1.467048	24.06	10.13	13.93	12.462952	-0.383952	-0.408	0.024048
15-Jun-08	06:50:00	1.394924	23.988	10.13	13.858	12.463076	-0.456076	-0.48	0.023924
15-Jun-08	07:00:00	1.318515	23.899	10.13	13.769	12.450485	-0.532485	-0.569	0.036515
15-Jun-08	07:10:00	1.230894	23.812	10.13	13.682	12.451106	-0.620106	-0.656	0.035894
15-Jun-08	07:20:00	1.156105	23.736	10.13	13.606	12.449895	-0.694895	-0.732	0.037105
15-Jun-08	07:30:00	1.082126	23.671	10.13	13.541	12.458874	-0.768874	-0.797	0.028126
15-Jun-08	07:40:00	1.015684	23.612	10.13	13.482	12.466316	-0.835316	-0.856	0.020684
15-Jun-08	07:50:00	0.9518876	23.553	10.13	13.423	12.4711124	-0.8991124	-0.915	0.0158876
15-Jun-08	08:00:00	0.8897183	23.484	10.13	13.354	12.4642817	-0.9612817	-0.984	0.0227183
15-Jun-08	08:10:00	0.8202071	23.414	10.13	13.284	12.4637929	-1.0307929	-1.054	0.0232071

15-Jun-08	08:20:00	0.7531381	23.353	10.13	13.223	12.4698619	-1.0978619	-1.115	0.0171381
15-Jun-08	08:30:00	0.6966665	23.298	10.13	13.168	12.4713335	-1.1543335	-1.17	0.0156665
15-Jun-08	08:40:00	0.647328	23.257	10.13	13.127	12.479672	-1.203672	-1.211	0.007328
15-Jun-08	08:50:00	0.6014535	23.218	10.13	13.088	12.4865465	-1.2495465	-1.25	0.0004535
15-Jun-08	09:00:00	0.5688306	23.184	10.13	13.054	12.4851694	-1.2821694	-1.284	0.0018306
15-Jun-08	09:10:00	0.5362068	23.158	10.13	13.028	12.4917932	-1.3147932	-1.31	-0.0047932
15-Jun-08	09:20:00	0.5182633	23.146	10.13	13.016	12.4977367	-1.3327367	-1.322	-0.0107367
15-Jun-08	09:30:00	0.5150008	23.14	10.13	13.01	12.4949992	-1.3359992	-1.328	-0.0079992
15-Jun-08	09:40:00	0.5082718	23.145	10.13	13.015	12.5067282	-1.3427282	-1.323	-0.0197282
15-Jun-08	09:50:00	0.527235	23.162	10.13	13.032	12.504765	-1.323765	-1.306	-0.017765
15-Jun-08	10:00:00	0.558432	23.188	10.13	13.058	12.499568	-1.292568	-1.28	-0.012568
15-Jun-08	10:10:00	0.5937058	23.214	10.13	13.084	12.4902942	-1.2572942	-1.254	-0.0032942
15-Jun-08	10:20:00	0.6224542	23.257	10.13	13.127	12.5045458	-1.2285458	-1.211	-0.0175458
15-Jun-08	10:30:00	0.6715899	23.303	10.13	13.173	12.5014101	-1.1794101	-1.165	-0.0144101
15-Jun-08	10:40:00	0.7260238	23.351	10.13	13.221	12.4949762	-1.1249762	-1.117	-0.0079762
15-Jun-08	10:50:00	0.7794361	23.408	10.13	13.278	12.4985639	-1.0715639	-1.06	-0.0115639
15-Jun-08	11:00:00	0.8299919	23.466	10.13	13.336	12.5060081	-1.0210081	-1.002	-0.0190081
15-Jun-08	11:10:00	0.8939989	23.526	10.13	13.396	12.5020011	-0.9570011	-0.942	-0.0150011
15-Jun-08	11:20:00	0.9577985	23.585	10.13	13.455	12.4972015	-0.8932015	-0.883	-0.0102015
15-Jun-08	11:30:00	1.022614	23.65	10.13	13.52	12.497386	-0.828386	-0.818	-0.010386
15-Jun-08	11:40:00	1.084164	23.716	10.13	13.586	12.501836	-0.766836	-0.752	-0.014836
15-Jun-08	11:50:00	1.152029	23.787	10.13	13.657	12.504971	-0.698971	-0.681	-0.017971
15-Jun-08	12:00:00	1.227022	23.874	10.13	13.744	12.516978	-0.623978	-0.594	-0.029978
15-Jun-08	12:10:00	1.315051	23.965	10.13	13.835	12.519949	-0.535949	-0.503	-0.032949
15-Jun-08	12:20:00	1.405315	24.054	10.13	13.924	12.518685	-0.445685	-0.414	-0.031685
15-Jun-08	12:30:00	1.494349	24.146	10.13	14.016	12.521651	-0.356651	-0.322	-0.034651
15-Jun-08	12:40:00	1.584598	24.234	10.13	14.104	12.519402	-0.266402	-0.234	-0.032402
					AVERAGE	12.48656305			0.00043695
					ST DEV	0.018879491			

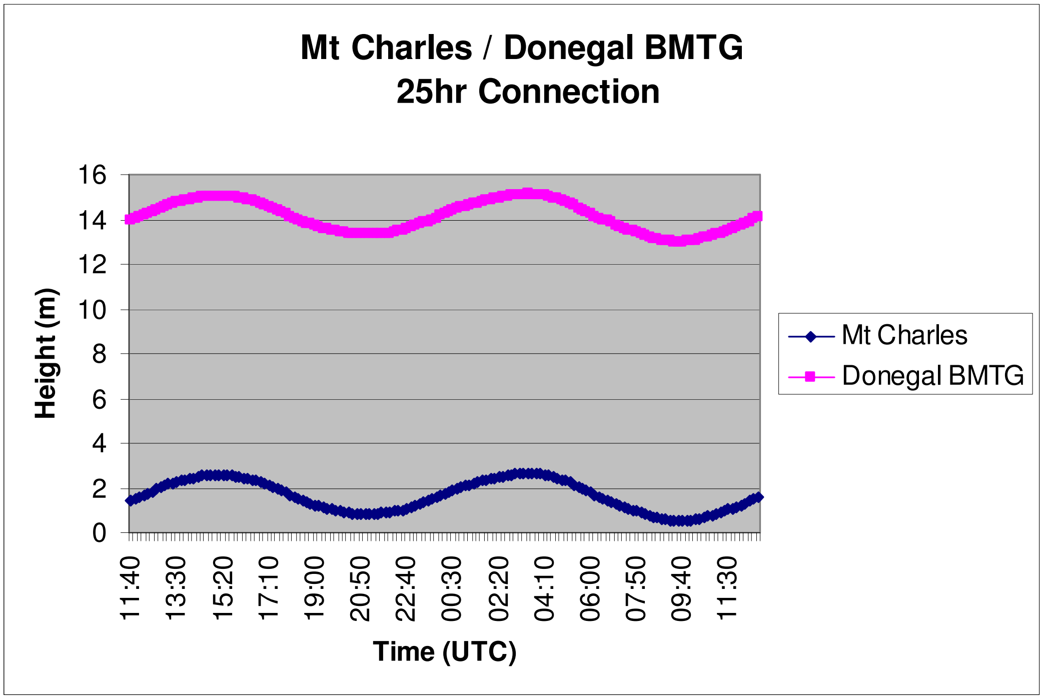
Mt Charles / Donegal BMTG
25hr Connection
Height (m)
16
14
12
10
8
6
4
2
0
11:40
13:30
15:20
17:10
19:00
20:50
22:40
00:30
02:20
04:10
06:00
07:50
09:40
11:30
Time (UTC)
Mt Charles
Donegal BMTG

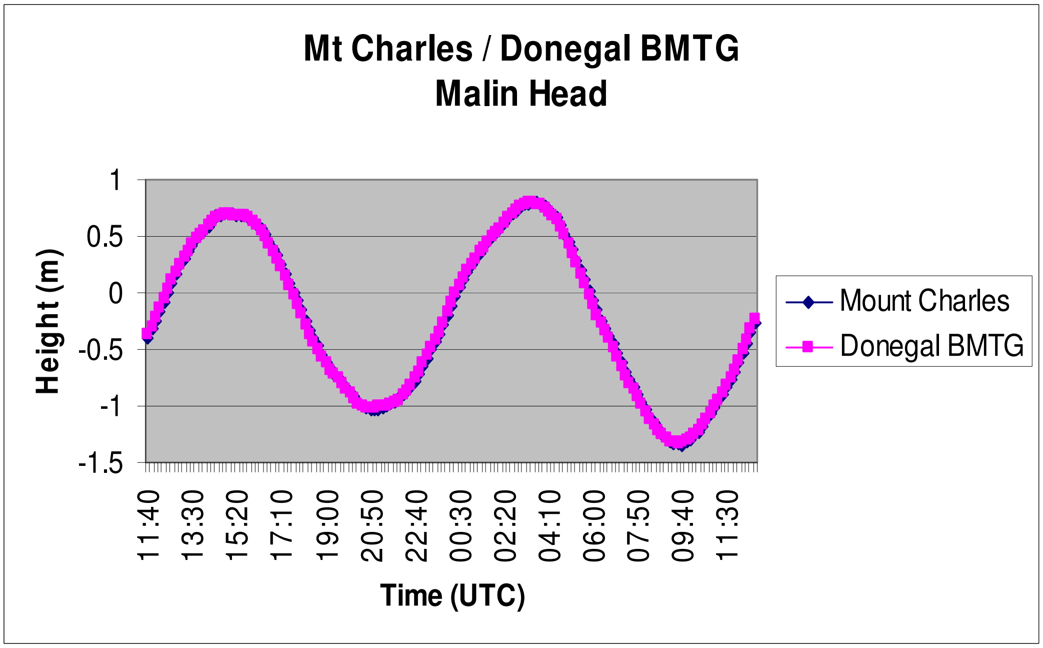
Mt Charles / Donegal BMTG
Malin Head
Height (m)
1
0.5
0
-0.5
-1
-1.5
11:40
13:30
15:20
17:10
19:00
20:50
22:40
00:30
02:20
04:10
06:00
07:50
09:40
11:30
Time (UTC)
Mount Charles
Donegal BMTG

Enclosure 4– LAT CONNECTIONS

NW Ireland 2008 - Datum Connection Summary

Survey Area	Tide Gauge	GSI Point Number	Easting ETRF 89	Northing ETRF 89	Latitude ETRF 89	Longitudes ETRF 89	Ellp Height	Height Above Malin Head	Geoid Height (GH)	MHd / GH Sep.	PolPred LAT below MSL (GH)	LAT below Marlin Hd
Galway Bay	Rossaveal LADS Gauge	Ross_HM	462520.349	5902013.125	53° 15' 57.740" N	09° 33' 42.986" W	60.958	3.370	3.502	0.132	2.68	2.81
	Rossaveal BLOM Gauge	at MHd datum	461980.415	5901716.401	53° 15' 48.0" N	09° 34' 12.0" W	57.592	0.000	0.13	0.13	2.68	2.81
	Galway Harbour INTG	Gauge Zero	496795.046	5902183.956	53° 16' 08.0" N	09° 02' 53.0" W	57.370	0.000	0.195	0.195	2.88	3.08
	Inishmore Island INTG	Gauge Zero	455363.267	5885580.271	53° 07' 04.0" N	09° 40' 01.0" W	57.922	0.000	0.146	0.146	2.60	2.75
Tralee Bay	Fenit Harbour LADS Gauge	M0112	441199.169	5791454.564	52° 16' 13.157178"N	09° 51' 42.235761"W	62.3258	3.905	4.101	0.196	2.41	2.606
	Fahamore Pier GSI Gauge	2008_09	429557.670	5796064.88	52° 18' 37.42437"N	10° 01' 59.79295"W	61.4973	2.912	3.116	0.204	2.41	2.614
Donegal Bay	Mullaghmore Hb LADS Gauge		535833.136	6035491.553	54° 27' 56.94656"N	08° 26' 49.73815" W	60.686	2.951	3.111	0.160	2.12	2.280
	Killybegs Harbour INTG	Gauge Zero	539049.916	6054498.491	54° 38' 11.0" N	08° 23' 42.0" W	57.788	0.000	0.140	0.140	2.10	2.240
	Mount Charles Pier LADS Gauge		551350.012	6053889.67	54° 37' 47.33845" N	08° 12' 16.41401" W	61.1089	3.454	3.600	0.146	2.16	2.306
	Donegal BMTG LADS Gauge	Gauge Zero	550287.592	6051744.904	54° 36' 38.340" N	08° 13' 16.980" W	57.648	0.000	0.146	0.146	2.16	2.306
	Donegal TG BLOM Gauge	at MHd datum	551750.642	6054007.409	54° 37' 51.0" N	08° 11' 54.0" W	57.652	0.000	0.145	0.145	2.16	2.305
	Inishcrone GSI Gauge		493580.272	6008026.369	54° 13' 12.72723" N	09° 05' 54.44662" W	60.832	2.758	2.916	0.158	2.05	2.208
Blacksod Bay	Blacksod Pier GSI Gauge		430682.527	5995178.966	54° 06' 00.379" N	10° 03' 36.165" W	61.812	3.379	3.506	0.127	2.27	2.397
Sligo	Sligo BLOM Gauge	at MHd datum	523631.344	6019557.722	54° 19' 24.0" N	08° 38' 12.0" W	57.819	0.000	0.174	0.174	2.09	2.264
**Survey Area**	**Tide Gauge**	**GSI Point Number**	**Easting ETRF 89**	**Northing ETRF 89**	**Latitude ETRF 89**	**Longitudes ETRF 89**	**Ellp Height**	**Height Above Belfast Lough**	**Geoid Height (GH)**	**Belfast Lough / GH Sep.**	**VORF LAT below MSL (GH)**	**LAT below Belfast Lough**
Lough Foyle	Greencastle GSI Gauge		628189.824	6119100.449	55° 12' 06.543042" N	6° 59' 08.816259" W	59.7847	2.887	2.939	0.052	1.33	1.382
	Lisahawley LADS Gauge	2008_05	610818.851	6100647.527	55° 02' 24.97886" N	7° 15' 56.79198" W	60.4031	3.159	3.239	0.080	1.33	1.410

Supplied by GSI
Calculated using Grid Inquest Program
Calculated using PolPred suppied by GSI

Calculated using GEIOD_RI software supplied by GSI
Calculated from above figures

Red - Supplied by GSI
Blue - Calculated by Grid Inquest
Light Blue - Calculated using PolPred supplied by GSI

Green - Calculated using GEOID_RI supplied by GSI
Black - Calculated from above figures

21/10/08

Enclosure 5 – NATIONAL TIDAL CENTER BMTG ANALYSIS

Sea Level Analysis for Donegal Bay, Ireland 2008

This analysis for TenixLADS of sea level data recorded by pressure gauge deployed in Donegal Bay (54° 36.6′ N, 8° 13.3′) was completed on the 3rd of July 2008. The sea level observations were analysed to derive tidal constituents and the tidal planes including mean sea level and Lowest Astronomical Tide (LAT).

This report details the results of the analysis, including a table of the tidal constituents and plots of the observations and residuals. Accompanying the report is a csv file of the ten-minute observation, prediction and residual data.

The data set received on the 1st of July consisted of two spreadsheet files; the first consisting of the 10-minute total pressures (in decibars) and temperature, and the second the hourly air pressure. The air pressure data was interpolated to 10-minute values then subtracted from the total pressures, which were then converted to sea levels using the algorithm presented in UNESCO Technical Papers in Marine Science No 44.

The initial harmonic tidal analysis was performed on 743 hours of data resolving 37 constituents. The constituents were used to determine mean sea level. The sea level observations were reduced to mean sea level and the data re-analysed. The tidal planes were then re-calculated with respect to mean sea level and are presented below.

To verify the analysis, 6-minute Donegal Bay predictions were compared with 6-minute tide gauge data obtained from nearby port of Killybegs (see below). The Killybegs sea level data was obtained from the Marine Institute web page (http://www.marine.ie/home/services/operational/oceanography/IrishTidesPorts.html?gauge=Killybegs%20Port).

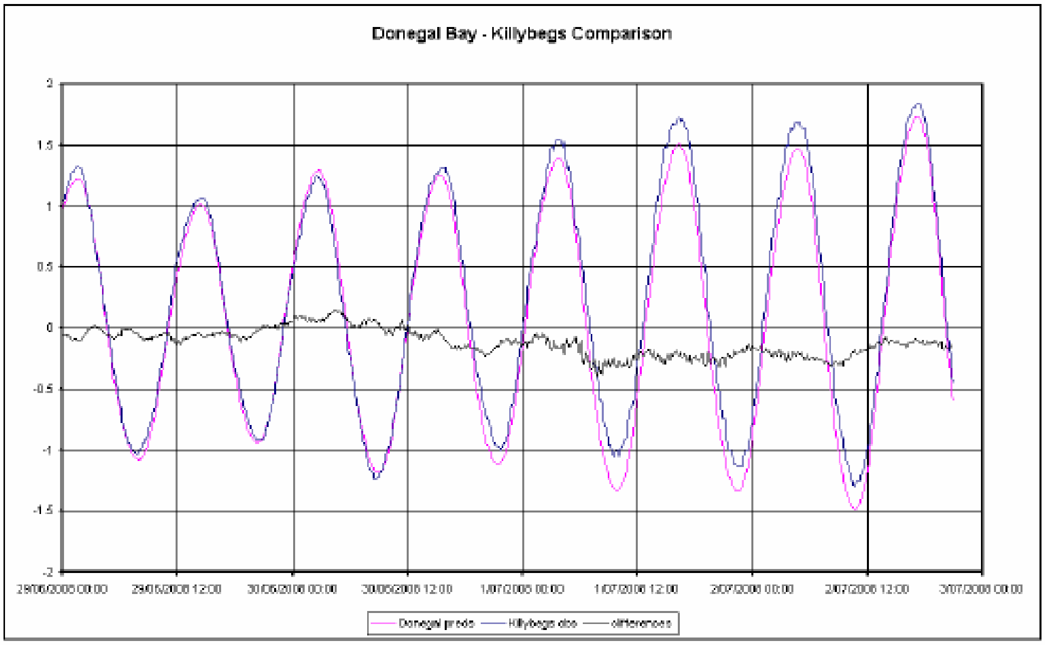

From the analysis LAT is 2.244 metres below mean sea level. The tide type is classified using the Form Factor : F = (K1 + O1) / (M2 + S2).

**value of F**	**category**
0 - 0.25	semidiurnal
0.25 - 1.5	mixed, mainly semidiurnal
1.5 - 3	mixed, mainly diurnal
> 3	diurnal

This analysis returned a form factor of 0.145. Being less than 0.25, the tide at Donegal Bay is classified as semidiurnal. The applicable tidal planes and levels for this classification are:

Highest Astronomical Tide (HAT) = 2.321
Mean High Water Spring (MHWS) = 1.674
Mean High Water Neap (MHWN) = 0.771
Mean Sea Level (MSL) = 0.000
Mean Low Water Neap (MLWN) = -0.771
Mean Low Water Spring (MLWS) = -1.674
Indian Spring Low Water (ISLW) = -1.917
Lowest Astronomical Tide (LAT) = -2.244

MONTHLY SEA LEVELS FOR DONEGAL BAY, IRELAND - 2008

Mth	Year	Gaps	Good	Minimum	Maximum	Mean	St Devn
1	2008	4464	0				
2	2008	4176	0				
3	2008	4464	0				
4	2008	4320	0				
5	2008	2248	2216	-1.398	1.618	0.022	0.832
6	2008	2076	2244	-1.791	1.897	-0.015	0.969
Totals		21748	4460				

```
Mean sea level =  0.003  (Average monthly means = 0.003)

Maximum recorded level of  1.897 metres at 1910 hours 05/06/2008
Minimum recorded level of -1.791 metres at 0210 hours 07/06/2008
Standard deviation of the observations =  0.9034 metres
Skewness                               =  0.0882
```

DONEGAL BAY - 2008 SEA LEVEL ANALYSIS - OBSERVATIONS REDUCED TO MSL

Mean	: residuals	0.000	observations	0.003
Mean of absolute value	: residuals	0.046	observations	0.791
Standard deviation	: residuals	0.056	observations	0.906

Distribution of residuals

From	To	Number	Percentage
-0.20	-0.10	144	3.25 %
-0.10	0.00	2140	48.24 %
0.00	0.10	1987	44.79 %
0.10	0.20	165	3.72 %

Number of values = 4436 , zeros = 33 and gaps = 19732

99999

DONEGAL BAY, IRELAND - 2008 ANALYSIS Lat 54° 37′ N Long 008° 13′ W Time zone 0000

Data in metres from 1-Jan-2008 to 16-Jun-2008
Analysis of 2-Jul-08 for 37 constituents on 31 days of data
Sample Correlation Coefficient is 0.9981
Standard Devn of the Residuals is 0.0562
Zero Frequency Level is 0.0002 metres (MSL)
Prediction Datum is MSL, 2.244 metres above LAT

Name	No.	Sigma	H (m)	g (deg)	Name	No.	Sigma	H (m)	g (deg)
Mm	3	0.544374	0.0295	252.8784	T2	35	29.958933	0.0267	193.5468
Mf	5	1.098033	0.0537	162.8314	S2	36	30.000000	0.4512	193.5468
SIGMA1	7	12.927139	0.0073	237.1270	K2	38	30.082137	0.1227	193.5468
Q1	8	13.398660	0.0398	295.6709	2SM2	41	31.015895	0.0038	56.1967
O1	10	13.943035	0.1048	351.7560	MO3	42	42.927139	0.0058	36.9424
M1	12	14.492052	0.0053	193.0832	M3	43	43.476156	0.0149	54.3044
P1	15	14.958931	0.0459	128.7964	SO3	44	43.943035	0.0041	154.6089
S1	16	15.000000	0.0016	128.7964	SK3	46	45.041068	0.0059	179.4363
K1	17	15.041068	0.1385	128.7964	MN4	47	57.423833	0.0105	346.9826
J1	21	15.585443	0.0164	208.6218	M4	48	57.968208	0.0257	33.1144
OO1	23	16.139101	0.0008	176.7158	SN4	49	58.439729	0.0022	214.8543
MNS2	25	27.423833	0.0173	81.3986	MS4	50	58.984104	0.0107	98.5145
2N2	26	27.895354	0.0324	136.0301	S4	52	60.000000	0.0015	158.8513
MEU2	27	27.968208	0.0511	89.3581	2MN6	54	86.407938	0.0173	195.2879
N2	28	28.439729	0.2453	136.0301	M6	55	86.952312	0.0274	228.8668
NEU2	29	28.512583	0.0465	136.0301	MSN6	56	87.423833	0.0045	114.2769
M2	31	28.984104	1.2227	156.9025	2MS6	57	87.968208	0.0145	292.2035
LAMDA2	33	29.455625	0.0084	177.5217	2SM6	59	88.984104	0.0042	326.7943
L2	34	29.528478	0.0311	177.5217					

COPYRIGHT COMMONWEALTH OF AUSTRALIA 2008 BUREAU OF METEOROLOGY NATIONAL TIDAL CENTRE

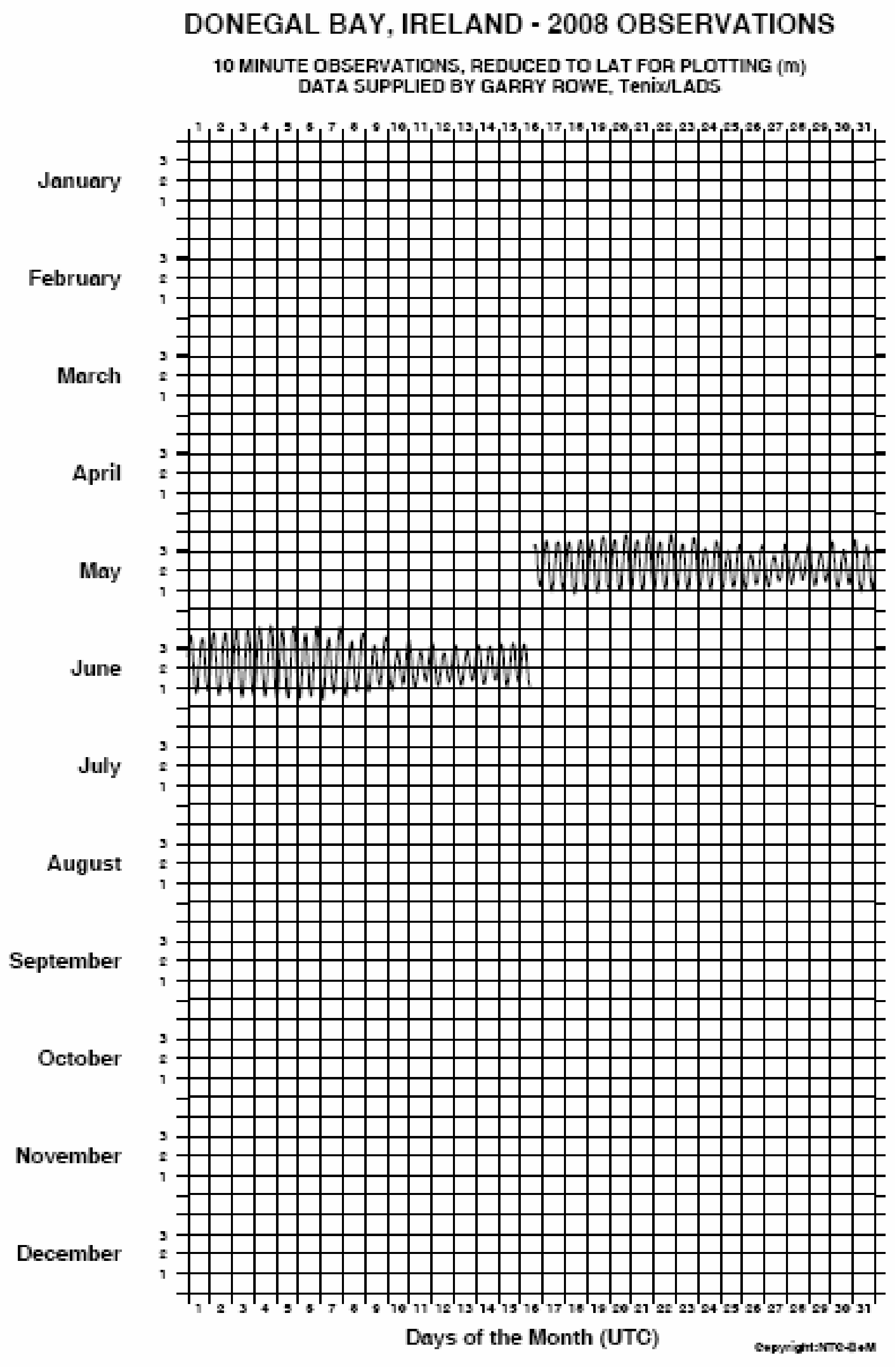
DONEGAL BAY, IRELAND - 2008 OBSERVATIONS
10 MINUTE OBSERVATIONS, REDUCED TO LAT FOR PLOTTING (m)
DATA SUPPLIED BY GARRY ROWE, Tenix/LADS
January
February
March
April
May
June
July
August
September
October
November
December
Days of the Month (UTC)
Copyright:NTC-BoM

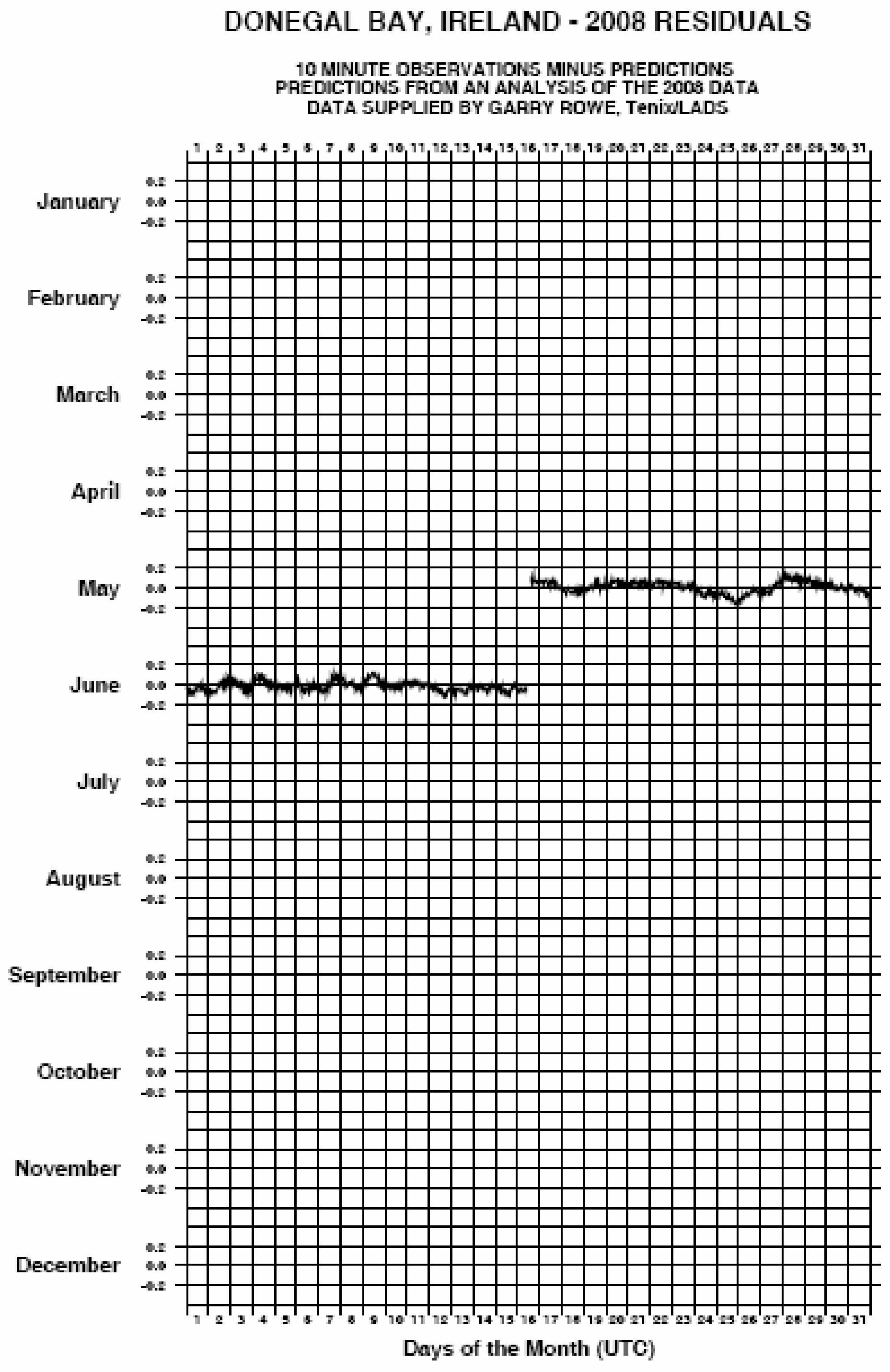
DONEGAL BAY, IRELAND - 2008 RESIDUALS
10 MINUTE OBSERVATIONS MINUS PREDICTIONS
PREDICTIONS FROM AN ANALYSIS OF THE 2008 DATA
DATA SUPPLIED BY GARRY ROWE, Tenix/LADS
January
February
March
April
May
June
July
August
September
October
November
December
Days of the Month (UTC)

Annex F. Calibration – Benchmark and Cross Line comparison results

F.1 General

Depth benchmarks and cross lines are used as the primary means of checking the quality of the depth data. A depth benchmark is typically a flat area of seabed which is re-surveyed on each sortie to check for correct system operation and correct application of tides in that area; the depth benchmark results can also be used in the assessment of the precision of the survey.

Cross lines are compared against main survey lines, and are used to check correct system operation and the tidal model throughout the survey area.

F.2 Benchmark Comparison Function

The LADS data is compared against the gridded benchmark surface in the Ground System and statistics are generated which include the number of points compared, the Mean Depth Difference (MDD) and the Standard Deviation (SD) between the data sets. The comparison compares the secondary data against the benchmark surface, and as this data is unedited it may contain noise normally removed during the validation process which is flagged as the shoalest and deepest differences.

F.3 Benchmarks

One benchmark areas were selected north of the Aran Islands, near the aircraft's base at Galway airport, at the start of the survey; however the highly variable water and weather conditions meant that no meaningful data was collected over these benchmarks on subsequent sorites. In addition, the wide spread of the geographical locations of each of the 5 survey areas undertaken meant that there was little opportunity to obtain repeat observations at the one location.

As a result no meaningful benchmark comparisons were calculated during this survey and a historical values of 0.15 metres (68% Confidence), gained from over 10 years of trials and survey operations, has been adopted for the system accuracy and been used in the calculation of the overall vertical accuracy of the depths.

The observed overlap between lines indicated that the LADS Mk II system operated correctly, and that the tidal model was consistent, and that tides were correctly applied.

F.4 Cross Line Comparisons

Fifteen cross lines were flown across the survey areas. Comparisons were made with main survey lines for analysis. Details are tabled below and the comparison summaries are provided at Enclosure 2.

F.4.1 Mean Depth Differences (MDD) and Standard Deviation (SD)

The averages of the Mean Depth Differences and Standard Deviation for each cross line run are as follows:

Run No.	Run Line Intersections	Comparisons	Mean Confidence	Average MDD	Average SD
50.0.1	5	6873	7.5	-0.09 +/- 0.09	0.18 +/- 0.03
51.0.1	28	43780	7.5	0.01 +/- 0.09	0.11 +/- 0.07
52.0.1	4	3946	7.1	0.04 +/- 0.01	0.25 +/- 0.06
53.1.1	9	8693	6.7	0.11 +/- 0.08	0.41 +/- 0.13
54.0.1	3	4275	7.6	-0.08 +/- 0.04	0.10 +/- 0.01
55.0.1	8	10815	6.8	0.03 +/- 0.06	0.35 +/- 0.03
56.1.1	3	2946	5.5	0.08 +/- 0.13	0.33 +/- 0.09
57.0.1	30	43983	7.3	0.01 +/- 0.13	0.23 +/- 0.19
65.0.1	31	51782	7.5	-0.03 +/- 0.05	0.09 +/- 0.04
66.0.1	25	40703	7.7	-0.04 +/- 0.05	0.08 +/- 0.04
68.0.1	3	5066	6.5	0.02 +/- 0.05	0.14 +/- 0.02
69.0.1	61	86772	7.3	0.13 +/- 0.14	0.08 +/- 0.05
70.0.1	30	50995	7.3	0.13 +/- 0.13	0.06 +/- 0.02
72.0.1	50	86986	7.8	0.03 +/- 0.03	0.08 +/- 0.05
**Total**	**290**	**447615**	**Average = 7.15**	**Average = 0.03 +/- 0.08**	**Average = 0.18+/- 0.06**

F.4.2 Cross Line Comparison Results

The mean of the average mean depth differences of 0.03 metres and the mean of the average standard deviations of 0.18 metres was generated from the intersection of 290 runs and the comparison of 447615 individual depths.

The final number of accepted comparisons was affected by the rejection of unacceptable values from this statistical analysis as a result of three main poor quality indications:

- Insufficient comparisons - intersections with less than 500 comparisons were rejected.
- Poor C0 confidence value - comparisons with a Subsurface Confidence less than 6 were rejected.
- Poor C1 confidence value – comparisons with a Near Neighbour Confidence of less than 4 were rejected.
- All comparisons containing topographic data was also rejected as this data contains artefacts such as trees and man made structures which influence the results.

The cross line comparisons are generally good in depths < 20 metres, however there is a increase in noise in the steep coastlines with large kelp forests; this was particularly true along the north shore of Galway Bay. This does not reflect deterioration in system performance, rather the complex nature of the seabed along these coastlines.

Details are attached as Enclosure 1.

Enclosure:

1. Cross Line Comparisons

Enclosure 1 – Cross Line Comparisons

Line 50.0.1

Reference Run No.	Target Run No.	Sortie No.	Number of Comps.	MDD	SD	CW	C0	C1	C2	Remarks
50.0.1	254.0.1.1	5	21	0.22	0.26	5.7	4.9	2.9	6	Rejected, Insufficient Comps
50.0.1	255.0.1.2	15	800	-0.15	0.18	7.5	7.4	7.3	7.8	
50.0.1	257.0.1.1	15	1668	-0.13	0.16	7.9	8.7	8.9	8.8	
50.0.1	258.0.1.1	5	1833	0.05	0.14	7.9	8.6	8.9	8.7	
50.0.1	259.0.1.1	15	1675	-0.17	0.2	7.5	8.9	7.5	7.9	
50.0.1	260.0.1.4	5	897	-0.03	0.22	6.7	7.3	7.2	8.1	
50.0.1	261.0.1.1	15	289	-0.11	0.31	7.2	7.8	8	8.1	Rejected, Insufficient Comps

Total Number of Comps.	Arithmetic Mean	Arithmetic Mean	Mean Confidence
**6873**	**-0.09 +/- 0.09**	**0.18 +/- 0.03**	**7.5**

Line 51.0.1

Reference Run No.	Target Run No.	Sortie No.	Number of Comps.	MDD	SD	CW	C0	C1	C2	Remarks
51.0.1	230.0.1.2	4	1580	-0.08	0.18	7.8	7.5	8.7	7.7	
51.0.1	232.0.1.2	4	1792	-0.07	0.15	8.2	8.3	8.8	8.4	
51.0.1	234.0.1.2	4	1816	-0.13	0.12	8.4	8.6	8.8	8.6	
51.0.1	236.0.1.4	4	1830	-0.08	0.12	8.3	8.5	8.8	8.5	
51.0.1	238.0.1.1	4	1620	-0.06	0.17	7.6	7.3	8.4	7.5	
51.0.1	239.0.1.1	7	448	0.19	0.2	6.6	5.3	4.5	6.9	Rejected, Insufficient Comps
51.0.1	240.0.1.4	4	1733	-0.06	0.17	7.7	7.2	8.6	7.5	
51.0.1	241.0.1.1	7	1483	0.15	0.18	6.9	6.6	6.9	7.6	
51.0.1	242.0.1.1	4	1805	-0.11	0.12	7.2	8.7	7.3	8.5	
51.0.1	243.0.1.1	7	1740	-0.08	0.3	7.6	8.4	8.3	8.3	
51.0.1	244.0.1.1	4	1104	-0.13	0.31	6	7.4	6.5	7.9	
51.0.1	245.1.1.2	14	132	0.14	0.21	2.6	7.7	2.9	7.8	Rejected, Insufficient Comps
51.0.1	246.0.1.1	4	357	-0.01	0.31	4.2	8	4.4	8.2	Rejected, Insufficient Comps
51.0.1	249.1.1.1	13	709	-0.04	0.09	7.7	8.7	8.4	8.8	
51.0.1	250.0.1.1	5	1548	0.04	0.08	8	8.9	8.6	8.9	
51.0.1	251.0.1.2	15	1770	0.03	0.06	8.3	9	8.9	9	
51.0.1	252.0.1.1	5	1744	0.01	0.05	6.9	9	7.8	9	
51.0.1	253.0.1.1	15	1847	0.02	0.05	8.7	9	9	9	
51.0.1	254.0.1.1	5	1793	-0.02	0.05	8	9	9	9	
51.0.1	255.0.1.2	15	1731	0.02	0.06	8	9	9	9	
51.0.1	256.0.1.3	5	1760	0	0.05	5.2	9	5.2	9	
51.0.1	257.0.1.1	15	1781	0.1	0.06	8.9	9	8.9	8.9	
51.0.1	258.0.1.1	5	1779	0	0.04	8	9	9	9	
51.0.1	259.0.1.1	15	1756	0.11	0.06	7.3	9	7.7	9	
51.0.1	260.0.1.4	5	1727	-0.01	0.04	6.7	9	7.6	9	
51.0.1	261.0.1.1	15	1757	0.24	0.11	7.6	9	8.5	9	
51.0.1	262.0.1.1	5	1713	0.06	0.04	9	9	9	9	

51.0.1	263.0.1.3	15	922	0.22	0.11	7.4	9	8.4	9	
51.0.1	264.0.1.1	5	579	0.05	0.06	4.2	9	4.7	9	
51.0.1	265.0.1.1	15	119	0.03	0.14	6.3	9	7.1	8.4	Rejected, Insufficient Comps
51.0.1	266.0.1.6	5	1021	-0.02	0.13	8	9	9	8.5	
51.0.1	268.0.1.2	5	1340	-0.02	0.11	5.9	9	6.7	8.5	

Total Number of Comps.	Arithmetic Mean	Arithmetic Mean	Mean Confidence
**43780**	**0.01 +/- 0.09**	**0.11 +/- 0.07**	**7.5**

Line 52.0.1

Reference Run No.	Target Run No.	Sortie No.	Number of Comps.	MDD	SD	CW	C0	C1	C2	Remarks
52.0.1	238.0.1.1	4	312	-0.05	0.25	6.3	5.5	2.8	6.9	Rejected, Insufficient Comps
52.0.1	239.0.1.1	7	16	0.09	0.21	1.3	4.4	0.2	6.7	Rejected, Insufficient Comps
52.0.1	248.0.2.1	5	527	0.01	0.35	4.5	4.9	2.2	4.8	Rejected, Low C0 & C1
52.0.1	249.1.1.1	13	139	-0.06	0.34	3	5	0.6	5.7	Rejected, Insufficient Comps
52.0.1	250.0.1.1	5	705	0.04	0.26	6.4	6.9	4.1	7.2	
52.0.1	251.0.1.2	15	71	0.07	0.17	5.7	5.5	1.6	6.5	Rejected, Insufficient Comps
52.0.1	252.0.1.1	5	1194	0.05	0.33	6.7	6	6.5	6.8	
52.0.1	254.0.1.1	5	1532	0.02	0.19	7.8	6.4	8.8	7.4	
52.0.1	256.0.1.1	5	515	0.03	0.2	7.4	7.4	8.3	8	

Total Number of Comps.	Arithmetic Mean	Arithmetic Mean	Mean Confidence
**3946**	**0.04 +/- 0.01**	**0.25 +/- 0.06**	**7.1**

Line 53.1.1

Reference Run No.	Target Run No.	Sortie No.	Number of Comps.	MDD	SD	CW	C0	C1	C2	Remarks
53.1.1	185.0.1.2	11	3	-0.06	0.22	6	8.7	1	7.3	Rejected, Insufficient Comps
53.1.1	186.0.1.1	4	421	0.1	0.42	2.8	7.7	2.4	7.4	Rejected, Insufficient Comps
53.1.1	186.0.2.3	4	425	0.04	0.35	6.9	6.9	5	7.2	Rejected, Insufficient Comps
53.1.1	187.0.1.4	11	990	0.05	0.44	6.8	5.9	7	7.4	Rejected, Low C0
53.1.1	188.0.1.1	4	922	0.09	0.58	6.5	7.2	6.4	6.9	
53.1.1	189.0.1.2	11	849	0.2	0.64	7	6.3	7.2	7.7	
53.1.1	190.0.2.2	5	1039	0.2	0.49	7	6.6	8	7.8	
53.1.1	191.0.1.1	7	1091	0.16	0.29	7.7	6.6	8.9	7.8	
53.1.1	192.0.1.2	5	1338	0.06	0.3	5.3	6.9	5.9	7.8	
53.1.1	193.0.1.1	7	854	0.11	0.37	6.1	7	6.8	7.8	
53.1.1	194.0.1.2	5	1093	0.08	0.33	7.3	7.6	8.3	8.2	
53.1.1	195.0.1.2	7	615	0.18	0.34	6.2	7.4	7	8.2	
53.1.1	196.0.1.1	5	327	0.16	0.35	0.6	8.6	0.7	7.9	Rejected, Insufficient Comps
53.1.1	205.0.1.1	9	490	0.14	0.4	0.7	9	0.7	8.9	Rejected, Insufficient Comps
53.1.1	206.0.1.1	7	892	-0.06	0.35	7.2	8.9	6.6	8.5	

Total Number of Comps.	Arithmetic Mean	Arithmetic Mean	Mean Confidence
**8693**	**0.11 +/- 0.08**	**0.41 +/- 0.13**	**6.7**

Line 54.0.1

Reference Run No.	Target Run No.	Sortie No.	Number of Comps.	MDD	SD	CW	C0	C1	C2	Remarks
54.0.1	635.0.1.1	9	1715	-0.1	0.1	6.4	8.9	7	8.9	
54.0.1	637.0.1.1	9	1698	-0.04	0.1	8.3	9	8.9	9	
54.0.1	639.0.1.1	9	862	-0.11	0.11	8	9	9	9	

Total Number of Comps.	Arithmetic Mean	Arithmetic Mean	Mean Confidence
**4275**	**-0.08 +/- 0.04**	**0.10 +/- 0.01**	**7.6**

Line 55.0.1

Reference Run No.	Target Run No.	Sortie No.	Number of Comps.	MDD	SD	CW	C0	C1	C2	Remarks
55.0.1	189.0.1.2	11	89	-0.12	0.35	6.7	7.4	4.9	7.4	Rejected, Insufficient Comps
55.0.1	190.0.2.2	5	434	-0.01	0.26	7	7.7	5.8	7.8	Rejected, Insufficient Comps
55.0.1	191.0.1.1	7	876	-0.08	0.36	7.1	7.4	7.2	7.2	
55.0.1	192.0.1.2	5	1133	0.11	0.3	6.8	7.1	6.1	7.1	
55.0.1	193.0.1.1	7	1589	0.01	0.36	7	7.2	7.9	7.1	
55.0.1	194.0.1.2	5	1574	0.05	0.35	6.5	6.4	6.9	6.6	
55.0.1	195.0.1.2	7	1720	0.07	0.34	7.1	7.5	8.1	7.3	
55.0.1	196.0.1.1	5	1646	0.04	0.34	7.2	8.1	7.7	7.3	
55.0.1	197.0.1.1	11	1265	0.08	0.39	5.6	7.3	6.4	7.9	
55.0.1	198.0.1.2	5	1012	-0.04	0.34	6.8	7.5	7.7	7.5	
55.0.1	199.0.1.1	11	205	-0.11	0.32	6.3	7.9	7	8.2	Rejected, Insufficient Comps

Total Number of Comps.	Arithmetic Mean	Arithmetic Mean	Mean Confidence
**10815**	**0.03 +/- 0.06**	**0.35 +/- 0.03**	**6.8**

Line 56.1.1

Reference Run No.	Target Run No.	Sortie No.	Number of Comps.	MDD	SD	CW	C0	C1	C2	Remarks
56.1.1	200.0.1.1	6	968	-0.04	0.27	7	5.5	7.3	7.1	Rejected, Low C0
56.1.1	203.0.1.1	11	125	0.06	0.55	5.8	5.1	1.9	7	Rejected, Insufficient Comps
56.1.1	204.0.1.1	7	442	-0.11	0.39	5.3	5.4	4	5.6	Rejected, Insufficient Comps
56.1.1	205.0.1.1	9	1394	0.07	0.3	1.2	7.2	1.3	7.6	Rejected, Low C1
56.1.1	206.0.1.3	7	1171	-0.04	0.43	0.5	6.6	0.6	6.1	Rejected, Low C1
56.1.1	170.0.1.1	4	902	0.01	0.29	6.6	5.9	6.2	6.6	
56.1.1	171.0.1.1	11	1025	0.23	0.43	4.9	6.1	5.1	7.4	
56.1.1	172.0.1.1	4	1019	0.01	0.27	4.9	6.2	5.7	7.4	
56.1.1	173.0.1.1	11	83	0.03	0.18	0.2	7.1	0.2	8.3	Rejected, Insufficient Comps

Total Number of Comps.	Arithmetic Mean	Arithmetic Mean	Mean Confidence
**2946**	**0.08 +/- 0.13**	**0.33 +/- 0.09**	**5.5**

Line 57.0.1

Reference Run No.	Target Run No.	Sortie No.	Number of Comps.	MDD	SD	CW	C0	C1	C2	Remarks
57.0.1	107.0.1.1	15	556	-0.22	0.18	7.7	8.5	8.7	8.6	
57.0.1	109.0.1.1	15	1467	-0.17	0.17	7.8	8.5	8.7	8.5	
57.0.1	111.0.1.1	15	1534	-0.17	0.15	7.8	8.3	8.7	8.5	
57.0.1	112.0.1.2	5	58	0.1	0.15	6.9	6.3	6.7	6.8	Rejected, Insufficient Comps
57.0.1	113.0.1.1	15	1876	-0.16	0.13	8.4	8.6	8.8	8.7	
57.0.1	114.0.1.1	5	1780	0.04	0.14	7.7	7.2	8.5	7.4	
57.0.1	115.0.1.1	7	1692	0.17	0.16	0.7	5.8	0.6	6.9	Rejected, Low C0 & C1
57.0.1	116.0.1.1	5	969	0.13	0.18	6.3	5.7	3.7	6.2	Rejected, Low C0 & C1
57.0.1	117.0.1.2	7	165	0.21	0.22	5.1	4.9	1.1	6.8	Rejected, Insufficient Comps
57.0.1	118.0.1.1	5	269	0	0.25	6.1	4.9	2.8	6.2	Rejected, Insufficient Comps
57.0.1	120.0.1.1	5	817	0.04	0.24	6.6	5.2	4.3	6.2	Rejected, Low C0
57.0.1	121.0.1.1	11	910	0.11	0.17	7.3	6.3	7.4	7.2	
57.0.1	138.0.1.1	6	189	0.1	0.23	5.5	5	2.2	6.6	Rejected, Insufficient Comps
57.0.1	138.1.1.1	15	105	0.04	0.3	5.9	4.8	1.9	6.4	Rejected, Insufficient Comps
57.0.1	139.0.1.1	12	964	0	0.2	6.9	6	5.3	7	
57.0.1	140.1.1.1	15	591	0.1	0.23	6	5.3	2.7	6.9	Rejected, Low C0 & C1
57.0.1	141.0.1.1	12	906	0.01	0.22	6.5	6.1	4.1	7	
57.0.1	142.0.1.3	7	1012	0	0.28	4.1	4.9	3.4	6.5	Rejected, Low C0 & C1
57.0.1	143.0.1.1	12	16	0.2	0.41	1.6	6.4	1.2	7.1	Rejected, Insufficient Comps
57.0.1	144.0.1.3	7	1536	-0.02	0.25	4.6	5	4.6	6.4	Rejected, Low C0
57.0.1	144.1.1.1	15	171	-0.01	0.2	7.1	6.3	6.6	7.2	Rejected, Insufficient Comps
57.0.1	146.0.1.2	7	1924	0.02	0.14	7.6	6.9	8.4	7.2	
57.0.1	146.1.1.1	15	1812	0.13	0.16	7.4	6.6	7.6	7.5	
57.0.1	148.0.1.2	7	1637	-0.05	0.26	7.1	6.8	7.8	7.5	
57.0.1	150.0.1.2	7	1625	0	0.29	6.9	7.2	7.5	7.9	
57.0.1	152.0.1.3	7	220	-0.07	0.29	5.9	5.6	7	7.1	Rejected, Insufficient Comps
57.0.1	154.0.1.1	7	1730	-0.03	0.22	7.2	7.4	8.2	7.8	
57.0.1	156.0.1.1	7	1828	0.03	0.13	8	8.9	8.9	8.9	

57.0.1	158.0.1.1	7	1973	0.1	0.05	7.7	9	8.7	9	
57.0.1	160.0.1.1	7	1118	0.12	0.23	7.7	9	7.5	8.6	
57.0.1	161.0.1.1	15	1466	-0.37	0.28	7.3	9	8.2	8.9	
57.0.1	162.0.1.1	7	1001	0.03	0.47	6.5	9	5.8	8.6	
57.0.1	163.0.1.3	15	1703	-0.1	0.3	6.9	8.9	7.7	8.9	
57.0.1	165.0.1.3	15	1428	0.06	0.07	7.9	9	8.9	9	
57.0.1	166.0.1.3	7	1220	0.23	0.08	7.8	9	8.8	8.6	
57.0.1	167.0.1.2	15	1682	-0.03	0.21	7.1	8.8	7.9	8.9	
57.0.1	168.0.1.2	7	659	0.16	1.15	6.8	8.9	7.7	8.5	
57.0.1	173.0.1.1	11	2196	0.01	0.27	7.4	8.1	8.5	8.3	
57.0.1	175.0.1.1	11	1415	0.09	0.25	7.1	7.6	8.1	8.3	
57.0.1	176.0.1.1	4	1982	-0.05	0.23	7.2	8.2	8.2	7.9	
57.0.1	177.0.1.1	11	1022	0.25	0.24	6.6	8.9	7.4	8.9	
57.0.1	180.0.1.1	5	2041	0	0.13	6.3	9	7.1	8.8	
57.0.1	181.0.1.1	11	1280	0.11	0.17	6.3	9	7.1	8.9	
57.0.1	182.0.1.1	5	1248	0.07	0.22	7.6	9	7.8	8.6	

**Total Number of Comps.**	**Arithmetic Mean**	**Arithmetic Mean**	**Mean Confidence**
**43983**	**0.01 +/- 0.13**	**0.23 +/- 0.19**	**7.3**

Line 65.0.1

Reference Run No.	Target Run No.	Sortie No.	Number of Comps.	MDD	SD	CW	C0	C1	C2	Remarks
65.0.1	504.0.1.2	18	403	-0.13	0.07	4.8	8.1	5.4	8	Rejected, Insufficient Comps
65.0.1	505.0.1.1	20	1339	0.04	0.07	7	8.8	7.8	8.8	
65.0.1	507.0.1.1	20	1109	0.07	0.12	7.7	8.1	6.8	8.3	
65.0.1	509.0.1.1	20	1199	-0.04	0.13	7.7	8.6	7.7	8.5	
65.0.1	511.0.1.1	20	1807	0.02	0.11	8	9	9	9	
65.0.1	513.0.1.1	20	1498	0.05	0.2	7.7	7.9	7.4	8.3	
65.0.1	515.0.1.5	20	35	0	0.24	5.7	5.2	2	7.1	
65.0.1	516.0.1.2	18	773	-0.17	0.18	6.9	6.1	5.7	6.9	
65.0.1	518.0.1.2	18	1865	-0.12	0.12	7.8	8.9	8.8	8.7	
65.0.1	519.0.1.1	20	1815	-0.03	0.11	7.8	8.8	8.8	8.7	
65.0.1	521.0.1.1	20	1915	-0.06	0.1	7.8	8.2	8.8	8.7	
65.0.1	522.0.1.1	18	1818	-0.08	0.09	7.3	8.6	8.2	7.7	
65.0.1	523.0.1.1	20	1736	-0.05	0.09	7.6	7.9	8.9	8.4	
65.0.1	524.0.1.1	18	1730	-0.06	0.07	7.5	9	8.4	8.3	
65.0.1	525.0.1.1	20	1798	-0.03	0.1	7.8	8	8.8	8.4	
65.0.1	526.0.1.1	18	1794	-0.04	0.08	7.5	8.7	8.4	8	
65.0.1	527.0.1.3	20	1827	0.01	0.1	7.8	8.5	8.8	8.5	
65.0.1	528.0.1.1	18	1758	-0.01	0.12	7.7	9	8.5	8.7	
65.0.1	529.0.1.1	20	1806	0	0.11	7.9	8	8.9	8.2	
65.0.1	530.0.1.1	18	1747	0	0.1	7.8	9	8.8	8.8	
65.0.1	532.0.1.1	18	1768	-0.06	0.06	7.9	9	8.9	8.9	
65.0.1	534.0.1.3	18	1790	0.02	0.06	7.9	9	8.9	8.8	
65.0.1	536.0.1.4	18	1712	-0.04	0.07	6.3	9	7.1	8.9	
65.0.1	538.0.1.2	18	1733	-0.01	0.04	8	9	9	9	
65.0.1	540.0.1.2	18	1651	0	0.05	7.8	8.9	8.8	9	
65.0.1	544.0.1.1	18	1725	-0.04	0.08	7.9	8.2	8.9	8.2	
65.0.1	546.0.1.1	18	1731	-0.04	0.06	7.9	8.9	8.9	8.6	

65.0.1	548.0.1.1	18	1787	-0.04	0.05	6.4	8.5	7.2	8.6	
65.0.1	550.0.1.1	18	1660	-0.04	0.06	4.8	8.1	5.4	8.7	
65.0.1	552.0.1.6	18	1765	-0.04	0.08	2.7	6.8	3	8.1	Rejected, Low C1
65.0.1	554.0.1.2	20	629	-0.12	0.11	0.5	4.8	0.6	6.8	Rejected, Low C0 & C1
65.0.1	555.0.1.2	20	1707	-0.01	0.1	7.5	6.4	8.7	7.8	
65.0.1	557.0.1.1	20	1758	0.08	0.08	0.6	6.9	0.7	8	Rejected, Low C1
65.0.1	559.0.1.3	20	1786	0.05	0.09	2.9	6.1	3.4	7.6	Rejected, Low C1
65.0.1	560.0.1.2	20	1599	-0.11	0.1	4.7	5.1	5.7	7.1	Rejected, Low C0
65.0.1	561.0.1.2	20	1764	0.03	0.09	7.6	6.4	8.7	7.8	
65.0.1	562.0.1.1	20	1648	-0.1	0.11	6.4	6.5	7.3	7.9	
65.0.1	563.0.1.1	20	7	-0.11	0.12	7.6	5.6	9	7.6	Rejected, Insufficient Comps
65.0.1	1638.0.1.1	18	1772	-0.03	0.08	8	7.9	9	8	

Total Number of Comps.	Arithmetic Mean	Arithmetic Mean	Mean Confidence
**51782**	**-0.03 +/- 0.05**	**0.09 +/- 0.04**	**7.5**

Line 66.0.1

Reference Run No.	Target Run No.	Sortie No.	Number of Comps.	MDD	SD	CW	C0	C1	C2	Remarks
66.0.1	504.0.1.2	18	615	0.01	0.1	7.7	9	9	8	
66.0.1	505.0.1.1	20	1280	-0.08	0.18	7.1	9	8	9	
66.0.1	507.0.1.1	20	1829	-0.06	0.13	7.6	9	9	9	
66.0.1	508.0.1.2	18	924	0	0.11	7.2	6	7	7	
66.0.1	509.0.1.1	20	1784	-0.04	0.1	7.8	9	9	9	
66.0.1	511.0.1.1	20	1104	-0.09	0.16	7.4	7	7	8	
66.0.1	517.0.1.1	20	743	0.08	0.21	6.2	5	4	7	Rejected, Low C0 & C1
66.0.1	519.0.1.1	20	1877	0.06	0.14	7.7	8	9	8	
66.0.1	521.0.1.1	20	1706	-0.02	0.09	8	9	9	9	
66.0.1	522.0.1.1	18	1715	-0.1	0.06	8	9	9	9	
66.0.1	523.0.1.1	20	1789	-0.03	0.06	8	9	9	9	
66.0.1	524.0.1.1	18	1842	-0.1	0.07	8	9	9	9	
66.0.1	525.0.1.1	20	1745	0.02	0.08	8	9	9	9	
66.0.1	526.0.1.1	18	1843	-0.09	0.05	8	9	9	9	
66.0.1	527.0.1.1	20	1773	0.05	0.06	8	9	9	9	
66.0.1	528.0.1.1	18	1748	-0.07	0.06	8	9	9	9	
66.0.1	529.0.1.1	20	1797	0.01	0.06	8	9	9	9	
66.0.1	530.0.1.1	18	1844	-0.08	0.06	8	9	9	9	
66.0.1	532.0.1.1	18	1749	-0.07	0.05	8	9	9	9	
66.0.1	534.0.1.1	18	1799	-0.11	0.09	8	9	9	9	
66.0.1	536.0.1.4	18	1819	-0.08	0.06	7	9	8	9	
66.0.1	538.0.1.2	18	1920	-0.07	0.06	7.7	9	9	9	
66.0.1	540.0.1.2	18	1914	-0.08	0.06	7.9	9	9	9	
66.0.1	542.0.1.4	18	1830	-0.03	0.06	7.9	9	9	9	
66.0.1	543.0.1.5	20	1768	0.05	0.05	0.6	9	1	9	Rejected, Low C1
66.0.1	545.0.1.8	20	1837	0.04	0.05	1.9	9	2	9	Rejected, Low C1

66.0.1	546.0.1.1	18	1772	-0.06	0.07	7.7	9	9	8	
66.0.1	548.0.1.1	18	685	0	0.06	5.9	9	7	9	

Total Number of Comps.	Arithmetic Mean	Arithmetic Mean	Mean Confidence
**40703**	**-0.04 +/- 0.05**	**0.08 +/- 0.04**	**7.7**

Line 68.0.1

Reference Run No.	Target Run No.	Sortie No.	Number of Comps.	MDD	SD	CW	C0	C1	C2	Remarks
68.0.1	1148.0.1.2	8	1570	0.03	0.15	7.3	7	8	7	
68.0.1	1150.0.1.2	8	1632	0.07	0.16	7.2	6	7	7	
68.0.1	1174.1.2.1	13	1864	-0.03	0.12	5.1	9	6	9	

Total Number of Comps.	Arithmetic Mean	Arithmetic Mean	Mean Confidence
**5066**	**0.02 +/- 0.05**	**0.14 +/- 0.02**	**6.5**

Line 69.0.1

Reference Run No.	Target Run No.	Sortie No.	Number of Comps.	MDD	SD	CW	C0	C1	C2	Remarks
69.0.1	1203.0.1.1	13	1804	-0.05	0.19	7.7	8	9	8	
69.0.1	1206.0.1.1	13	506	-0.15	0.5	3.9	5	2	5	Rejected, Poor C0. Poor C1
69.0.1	1208.0.1.1	13	926	-0.08	0.37	4.3	5	2	4	Rejected, Poor C0. Poor C1
69.0.1	1210.0.1.1	13	1487	-0.02	0.31	5.3	5	5	6	Rejected, Poor C1
69.0.1	1212.0.1.1	13	1570	-0.09	0.26	6.2	5	6	6	Rejected, Poor C1
69.0.1	1214.0.1.1	13	1208	-0.1	0.36	5.2	5	4	5	Rejected, Poor C1
69.0.1	1216.0.1.1	13	1586	-0.07	0.21	6.8	6	7	7	
69.0.1	1218.0.1.1	13	1643	-0.12	0.17	7.4	7	8	8	
69.0.1	1220.0.1.1	13	1618	-0.11	0.14	7.2	7	8	7	
69.0.1	1222.0.1.1	13	1187	0.01	0.24	6.8	6	7	7	Rejected, Poor C0
69.0.1	1224.0.1.1	13	1655	-0.03	0.18	6.8	7	7	7	
69.0.1	1226.0.1.1	13	1802	0	0.12	7.3	8	8	8	
69.0.1	1250.0.1.2	13	1905	-0.07	0.11	0	9	0	9	Rejected, Poor C1
69.0.1	1252.0.1.2	13	1658	-0.12	0.11	7.7	9	9	9	
69.0.1	1254.0.1.1	13	1735	-0.12	0.15	7.5	8	8	8	
69.0.1	1256.0.1.1	9	1849	0.17	0.12	7.5	8	9	8	
69.0.1	1258.0.1.4	9	1610	0.2	0.19	6.3	7	7	8	
69.0.1	1260.0.1.4	9	1300	0.15	0.23	5.7	6	7	7	Rejected, Poor C0
69.0.1	1261.0.1.3	17	1509	-0.02	0.23	6.3	7	7	7	
69.0.1	1262.0.1.2	9	1751	0.26	0.13	6.5	8	7	8	
69.0.1	1263.0.1.2	17	1808	0.2	0.11	6.7	9	8	8	
69.0.1	1264.0.1.1	8	1761	0.23	0.07	7.2	9	8	9	

69.0.1	1265.0.1.1	17	1808	0.21	0.06	7.7	9	9	9	
69.0.1	1266.0.1.1	8	1792	0.23	0.05	7.5	9	8	9	
69.0.1	1267.0.1.2	17	1786	0.24	0.05	7.2	9	8	9	
69.0.1	1268.0.1.1	8	1847	0.23	0.05	7.5	9	8	9	
69.0.1	1269.0.1.3	17	1829	0.27	0.04	8	9	9	9	
69.0.1	1270.0.1.1	8	1862	0.21	0.05	7.1	9	8	9	
69.0.1	1271.0.1.2	17	1805	0.25	0.05	7.8	9	9	9	
69.0.1	1272.0.1.1	8	1833	0.23	0.04	7	9	8	9	
69.0.1	1273.0.1.2	17	1779	0.22	0.06	7.6	9	9	9	
69.0.1	1274.0.1.1	8	1759	0.23	0.05	7.4	9	8	9	
69.0.1	1275.0.1.2	17	1802	0.26	0.06	7.1	9	8	9	
69.0.1	1276.0.1.1	8	1706	0.21	0.05	7.2	9	8	9	
69.0.1	1277.0.1.2	17	1801	0.25	0.05	7.4	9	8	9	
69.0.1	1278.0.1.2	8	1838	0.18	0.04	4.9	9	6	9	
69.0.1	1280.0.1.2	8	90	0.06	0.05	7.9	9	9	9	Rejected, too few comparisons
69.0.1	1282.0.1.3	8	1829	0.19	0.06	8	9	9	9	
69.0.1	1284.0.1.2	8	1778	0.19	0.06	8	9	9	9	
69.0.1	1284.1.1.2	16	1777	0.25	0.05	7.2	9	8	9	
69.0.1	1300.0.1.2	8	1764	0.2	0.06	7.6	9	9	9	
69.0.1	1301.0.1.2	17	1775	0.27	0.06	8	9	9	9	
69.0.1	1302.0.1.2	8	1770	0.24	0.05	7.7	9	9	9	
69.0.1	1304.0.1.2	8	1778	0.21	0.05	7.2	9	8	9	
69.0.1	1306.0.1.4	8	1782	0.2	0.04	7.1	9	8	9	
69.0.1	1308.0.1.2	8	1757	0.21	0.05	7.7	9	9	9	
69.0.1	1310.0.1.2	8	1689	0.27	0.05	6.5	9	7	9	

69.0.1	1312.0.1.2	8	1697	0.26	0.05	7.8	9	9	9	
69.0.1	1314.0.1.1	8	1688	0.26	0.06	7.5	9	8	9	
69.0.1	1316.0.1.1	8	1661	0.31	0.06	8	9	9	9	
69.0.1	1318.0.1.2	10	1684	0.08	0.06	2.1	9	2	9	Rejected, Poor C1
69.0.1	1320.0.1.1	13	1646	-0.01	0.06	7.7	9	9	9	
69.0.1	1322.0.1.1	13	1674	0.03	0.06	7.6	9	9	9	
69.0.1	1324.0.1.1	13	1704	0	0.05	7.6	9	9	9	
69.0.1	1326.0.1.1	13	1609	0	0.05	6.6	9	8	9	
69.0.1	1328.0.1.1	13	1808	0.03	0.06	7.8	9	9	9	
69.0.1	1330.0.1.1	13	1708	-0.01	0.05	6.6	9	7	9	
69.0.1	1332.0.1.1	13	1620	0.01	0.04	6	9	7	9	
69.0.1	1334.0.2.2	16	1334	-0.2	0.1	7.3	9	8	9	
69.0.1	1336.0.1.1	16	1888	0.17	0.19	8	9	9	9	
69.0.1	1456.0.1.1	16	46	0.25	0.19	3.3	5	1	5	Rejected, too few comparisons

**Total Number of Comps.**	**Arithmetic Mean**	**Arithmetic Mean**	**Mean Confidence**
**86772**	**0.13 +/- 0.14**	**0.08 +/- 0.05**	**7.3**

Line 70.0.1

Reference Run No.	Target Run No.	Sortie No.	Number of Comps.	MDD	SD	CW	C0	C1	C2	Remarks
70.0.1	1282.0.1.3	8	1767	0.17	0.06	8	9	9	9	
70.0.1	1284.0.1.2	8	1773	0.13	0.05	8	9	9	9	
70.0.1	1300.0.1.2	8	1765	0.19	0.05	7.6	9	9	9	
70.0.1	1301.0.1.2	17	1734	0.33	0.05	8	9	9	9	
70.0.1	1302.0.1.2	8	1702	0.18	0.05	7.7	9	9	9	
70.0.1	1304.0.1.2	8	1765	0.21	0.04	7.2	9	8	9	
70.0.1	1306.0.1.4	8	1750	0.19	0.06	7.1	9	8	9	
70.0.1	1308.0.1.2	8	1769	0.22	0.05	7.7	9	9	9	
70.0.1	1310.0.1.2	8	1721	0.23	0.05	6.6	9	7	9	
70.0.1	1312.0.1.2	8	1691	0.18	0.05	7.8	9	9	9	
70.0.1	1314.0.1.1	8	1658	0.17	0.05	7.5	9	8	9	
70.0.1	1316.0.1.1	8	1695	0.22	0.07	8	9	9	9	
70.0.1	1318.0.1.2	10	1757	0.12	0.04	2.4	9	3	9	Rejected, Poor C1
70.0.1	1320.0.1.1	13	1820	0	0.04	7.7	9	9	9	
70.0.1	1322.0.1.1	13	1758	-0.01	0.04	7.6	9	9	9	
70.0.1	1324.0.1.1	13	1748	0.02	0.07	7.6	9	9	9	
70.0.1	1326.0.1.1	13	1766	0.02	0.08	6.7	9	8	9	
70.0.1	1328.0.1.1	13	1704	0.04	0.06	7.8	9	9	9	
70.0.1	1330.0.1.1	13	1754	0.01	0.05	6.6	9	8	9	
70.0.1	1332.0.1.1	13	1632	0.01	0.06	6.1	9	7	9	
70.0.1	1334.0.1.4	16	1661	0.19	0.09	4.4	9	5	6	
70.0.1	1334.0.2.2	16	1573	0.28	0.11	7	8	8	7	
70.0.1	1336.0.1.1	16	1724	0.42	0.11	7.7	7	9	7	
70.0.1	1338.0.1.1	16	1661	0.39	0.1	7.4	8	8	8	
70.0.1	1340.0.1.1	13	1736	0	0.05	8	9	9	9	
70.0.1	1340.0.2.2	13	1735	-0.05	0.05	7.8	9	9	9	
70.0.1	1342.0.1.1	13	1668	0.03	0.06	7.4	9	8	9	
70.0.1	1344.0.1.1	13	1688	0	0.05	7.5	9	8	9	

70.0.1	1346.0.1.3	13	1685	0.01	0.05	7.9	9	9	9	
70.0.1	1348.0.1.1	13	1655	-0.02	0.05	6.9	9	8	9	
70.0.1	1350.0.1.1	13	1237	-0.01	0.06	6.4	9	7	9	

Total Number of Comps.	Arithmetic Mean	Arithmetic Mean	Mean Confidence
**50995**	**0.13 +/- 0.13**	**0.06 +/- 0.02**	**7.3**

Line 72.0.1

Reference Run No.	Target Run No.	Sortie No.	Number of Comps.	MDD	SD	CW	C0	C1	C2	Remarks
72.0.1	1850.0.1.2	19	1848	0.07	0.05	8	9	9	9	
72.0.1	1852.0.1.3	19	1812	0.06	0.04	6.3	9	7	9	
72.0.1	1854.0.1.2	19	1801	0.05	0.05	7.9	9	9	9	
72.0.1	1856.0.1.3	19	1754	0.04	0.05	7.2	9	8	9	
72.0.1	1858.0.1.2	19	1758	0.05	0.04	8	9	9	9	
72.0.1	1860.0.1.2	19	1752	0.06	0.04	6.7	9	8	9	
72.0.1	1862.0.1.2	19	1759	0.05	0.05	8	9	9	9	
72.0.1	1864.0.1.2	19	1829	0.05	0.04	8	9	9	9	
72.0.1	1868.0.1.2	19	1789	0.08	0.05	8	9	9	9	
72.0.1	1870.0.1.1	19	1764	0.08	0.05	8	9	9	9	
72.0.1	1872.0.1.1	19	1755	0.08	0.06	8	9	9	9	
72.0.1	1874.0.1.1	19	1788	0.05	0.05	8	9	9	9	
72.0.1	1876.0.1.1	19	1738	0.04	0.05	8	9	9	9	
72.0.1	1878.0.1.1	19	1742	0.04	0.05	8	9	9	9	
72.0.1	1880.0.1.1	19	1780	0.06	0.04	8	9	9	9	
72.0.1	1882.0.1.1	19	1736	0.05	0.05	8	9	9	9	
72.0.1	1884.0.1.1	19	1686	0.05	0.05	8	9	9	9	
72.0.1	1886.0.1.1	19	1791	0.05	0.05	8	9	9	9	
72.0.1	1888.0.1.1	19	1694	0.05	0.05	8	9	9	9	
72.0.1	1890.0.1.1	19	1748	0.05	0.05	8	9	9	9	
72.0.1	1892.0.1.1	19	1712	0.02	0.05	8	9	9	9	
72.0.1	1894.0.1.1	19	1725	0.03	0.05	8	9	9	9	
72.0.1	1896.0.1.1	19	1723	0.02	0.06	8	9	9	9	
72.0.1	1898.0.1.1	19	1709	0.01	0.05	8	9	9	9	
72.0.1	1900.0.1.1	19	1779	0.03	0.06	8	9	9	9	
72.0.1	1902.0.1.1	19	1751	0.01	0.06	8	9	9	9	
72.0.1	1904.0.1.1	19	1741	-0.01	0.06	8	9	9	9	
72.0.1	1905.0.1.1	19	1750	0	0.06	9	9	9	9	

72.0.1	1907.0.1.1	19	1754	-0.02	0.06	8.2	9	9	9	
72.0.1	1909.0.1.1	19	1747	-0.04	0.06	8	9	9	9	
72.0.1	1911.0.1.1	19	1741	-0.04	0.06	8.3	9	9	9	
72.0.1	1913.0.1.1	19	1751	0.02	0.06	8	9	9	9	
72.0.1	1915.0.1.1	19	1706	0.01	0.06	8	9	9	9	
72.0.1	1917.0.1.1	19	1797	0.02	0.06	8	9	9	9	
72.0.1	1919.0.1.1	19	1804	0.02	0.05	8	9	9	9	
72.0.1	1921.0.1.1	19	1638	0.04	0.19	6.4	7	7	7	
72.0.1	1923.0.1.1	19	1567	-0.04	0.29	5.6	5	7	7	Rejected, Low C0
72.0.1	1925.0.1.1	19	1205	0	0.22	6.5	7	7	8	
72.0.1	1927.0.1.1	19	1695	0	0.2	7.4	8	8	8	
72.0.1	1929.0.1.1	19	1678	-0.03	0.08	7.9	9	9	9	
72.0.1	1931.0.1.1	19	1753	0.02	0.11	7.8	9	9	9	
72.0.1	1933.0.1.1	19	1718	0.05	0.13	7.8	8	9	9	
72.0.1	1935.0.1.1	19	1720	0.05	0.19	7.5	8	9	8	
72.0.1	1937.0.1.1	19	1869	-0.04	0.15	7.2	8	8	8	
72.0.1	1939.0.1.1	19	1649	0.02	0.11	7.9	9	9	9	
72.0.1	1941.0.1.1	19	1774	0.02	0.1	8	9	9	9	
72.0.1	1943.0.1.1	19	1722	0.05	0.15	7.6	8	9	8	
72.0.1	1945.0.1.1	19	1789	0.06	0.16	7.8	8	9	8	
72.0.1	1947.0.1.1	19	1734	0.07	0.15	8	8	9	9	
72.0.1	1949.0.1.1	19	1900	0.06	0.19	7.7	6	9	7	
72.0.1	1951.0.1.1	19	1628	0.08	0.21	7.4	6	9	7	

Total Number of Comps.	Arithmetic Mean	Arithmetic Mean	Mean Confidence
**86986**	**0.03 +/- 0.03**	**0.08 +/- 0.05**	**7.8**

Annex G. Vertical and Horizontal Accuracy of Soundings

G.1 Horizontal Accuracy of Soundings

G.1.1 Theoretical Accuracy

The theoretical accuracy of the positioning systems is related to the distance of the roving GPS receiver from the base station. The relationship between baseline distance and theoretical accuracy was provided by Thales GeoSolutions UK for the Ashtech GG24 WADGPS receiver and is based on empirical data using LandStar corrections. The Thales LandStar correction service was withdrawn by Fugro in late 2004 and replaced with Fugro OmniStar corrections using the Virtual Base Station service.

G.1.2 Fugro OmniStar Wide Area Real-Time DGPS solution

Empirical tests undertaken by Thales GeoSolutions U.K. have detailed the horizontal standard deviation of positions obtained by the Ashtech GG24 WADGPS receiver with LandStar DGPS corrections at varying baseline distances. The expected error has been determined to be 1.01m + 1.4ppm. This standard deviation defines the theoretical repeatability of position fixes at various ranges. In 2004 the LandStar WADGPS service was withdrawn and replaced with the Fugro OmniStar system, which provides for a virtual station based on the real-time raw GPS feed that is then used in the generation of a solution from the nearest five base stations. Previous Thales advice also stated that the user should also be aware of the following: "DGPS systems are single frequency systems: the DGPS corrections are corrections to the user's pseudo-ranges, which are only available on the L1 frequency. Due to this, ionospheric delays cannot be corrected for, and this will cause a bias in the position. This bias is typically about 20-25 centimetres per 100 kilometres (2.5ppm), and will be in the same direction as the 'baseline' between reference station and user. Therefore if corrections are taken from a DGPS station due north, the position will be consistently out in latitude. This is a general limitation of DGPS systems, since they are single frequency systems". By adopting a virtual base station in the position of the aircraft and generating a wide area solution for this position based on up to five fixed base stations, much of this error is reduced.

No accuracy figures for the modelled Fugro OmniStar corrections are available; however given that the modelled solution is calculated for the real-time position based on observations at up to five base stations, the achieved accuracies for the real-time system during operations should be similar to the observed absolute accuracy obtained during the static position check conducted under similar conditions.

The accuracy of the WADGPS position was found to be 2.73m (95% confidence) as determined by the static position check on 21 May 2008. This is consistent with previous results and is sufficient for the real-time positioning of the aircraft. In any event, more accurate post-processed KGPS positions were applied to soundings during post-processing.

G.1.3 Post-Processed Novatel GrafNav Dual Frequency KGPS Solution

The theoretical accuracy of the post-processed GrafNav positional data has been determined from the GrafNav Software User's Manual and through consultation with Novatel. For a PDOP of less than 5 the following GrafNav data processing accuracy has been quoted:

L1/L2 Carrier Phase, float ambiguities

fwd / backwd processing (KGPS) = 0.3 metres + 1ppm (worst case)

For this survey the maximum baseline distance between the local GPS base station (Radisson SAS Hotel, Galway) and the aircraft was approximately 250km. Therefore the expected accuracy of the post-processed solutions are:

L1/L2 carrier phase (KGPS) = 0.55 metres

G.1.4 Practical Accuracy

The actual performance of the positioning solutions were checked by:

a. Static position check

b. Dynamic position check

G.1.5 Static Position Check

Static position checks were conducted for the following GPS positions:

a. WADGPS: Fugro OmniStar Wide Area Differential GPS (real-time)

b. KGPS: Forward and backward processed L1/L2 carrier phase, float ambiguities (off-line)

c. Raw GPS: Stand-alone GPS receiver (real-time)

The static position check results are enclosed at Annex D.

G.1.6 Dynamic Position Monitoring

During the survey, GPS data was logged on the aircraft and at the local base station, which enabled post-processing to produce KGPS result files (off-line). These result files were then compared to the position as determined by the real-time WADGPS on the AS. For each survey line, the mean difference and standard deviation have been calculated. The dynamic position check results for each sortie are referenced in the paragraph 'WADGPS – Fugro OmniStar' in Annex D.

G.1.7 Accuracy of Position

The total expected error of the LADS Mk II system is a combination of the following errors:

a. GPS errors (Egps), as previously stated, have a theoretical maximum of ± 0.55 metres (95% confidence - KGPS).

b. Errors in assigning frame centre reference positions from GPS fixes (Eframe ref) have been assessed as ± 0.66 metres (95% confidence).

c. Platform and laser positioning errors (Eplat, this includes such errors as gimbal angles, optical alignment, AHRS angles, AHRS mount, Optical Coupler mount, Scanner mount, Laser output, Laser mount, Major, Minor and Delta scan mirrors, timing and aircraft height). The resultant error in position has been assessed as ± 1.3 metres (95% confidence).

d. Position errors of detecting objects due to the distance between laser spots (Espot). With a 5x5m laser spot spacing it is considered the worst case for the position is ± 2.5 metres based on the sample interval (95 % confidence).

e. Sea surface errors (Esurface) due to swell. These are variable and dependant on the angle of incidence of the laser beam at the air/sea boundary, the depth of water and sea state.

They have been assessed and are tabled below.

Depth (m)	Sea State 1	Sea State 2	Sea State 3	Sea State 4
5	0	0.03	0.31	0.55
10	0.01	0.06	0.62	1.10
15	0.01	0.09	0.93	1.65
20	0.02	0.12	1.24	2.20
30	0.04	0.18	1.86	3.30

Table 1 – Sea surface errors due to sea state

Total Expected Error = $((Egps)^2 + (Eframe\ Ref)^2 + (Eplat)^2 + (Espot)^2 + (Esurface)^2)\ ½$

In the following scenario, at 250 kilometres from the local KGPS base station at the Radisson SAS Hotel, Galway, in a depth of 20 metres, with sea state 3, the total error is expected to be:

Total Expected Error = $((0.55^2 + (0.66)^2 + (1.3)^2 + (2.5)^2 + (1.24)^2)^{½}$

= 3.2 metres at the 95% confidence level

Analysing the positional data obtained from both the static and dynamic position checks it has been concluded that during the survey IHO Order-1 precision for position was achieved.

G.1.8 GPS Positional Accuracy - Summary

G.1.8.1 Static Position Check

Radisson SAS Hotel, Galway

Absolute accuracy of real-time WADGPS Virtual Base Station Service = 2.73 metres

Absolute accuracy of GrafNav post-processed KGPS (4.0 km baseline) = 0.17 metres

G.1.8.2 Dynamic Position Check

All Areas

Mean value of range distances over all lines of survey	
between WADGPS and GrafNAV KGPS	= 0.97 metres
Maximum value of range distance, over all lines of survey	
between WADGPS and GrafNAV KGPS position	= 4.23 metres

G.1.9 LADS Mk II System Positional Accuracy

G.1.9.1 Theoretical Accuracy

Maximum (depth = 30m, sea state 3, baseline 250 km)	= 3.2 metres
IHO Order-1 Horizontal Accuracy – 30m	
(95% confidence)	= 5 metres + 5% of the depth
	= 6.5 metres
Survey Horizontal Accuracy	
(95% confidence)	= better than 4.0 metres

G.2 Vertical accuracy of soundings

G.2.1 LADS Mk II System Accuracy

A standard deviation of 0.15 metres (68% confidence) is the historical average for the LADS system obtained from historical benchmark data collected during previous surveys and during trials. This value has been adopted as the LADS MkII system accuracy for this survey.

G.2.2 Tides and Tidal Models

Tides were observed from multiple tide gauges throughout all survey areas, and the range of the observed tides was small. Thus the residual error due to tides is small and an accuracy value of 0.10m (68% confidence) was given to the observed tides used for the survey.

G.2.3 Swell

Swell had a small affect on survey operations in all the survey areas. The swell never exceeded 1 metres in most areas and 2 metres in the exposed parts of the Tralee Bay and Galway Bay areas. An allowance of 0.15 metres (68% confidence) has been allowed for the residual affects of swell and sea state.

G.2.4 Water Clarity

Water clarity was variable throughout the survey area and where possible data affected by varying water clarity has been removed and re-flown. An allowance of 0.15 metres (68% confidence) for the affects of degraded water clarity has been included in the accuracy model.

G.2.5 Accuracy of Soundings

An assessment of the total survey accuracy can be determined by combining the errors due to the LADS Mk II system, tidal model, swell and water clarity. These are combined using a Gaussian model as follows:

σ^2 Survey = σ^2 LADS Mk II System + σ^2 Water Clarity + σ^2 Residual Swell + σ^2 Tidal Model

and 95% confidence limit = 1.96σ [for a single dimensional distribution]

Average Depth	LADS Mk II σ total	Water Clarity σ	Residual Swell σ	Tidal Model σ	Survey Accuracy (68% conf.)	Survey Accuracy (95% conf.)	IHO Order-1
20 m	0.15	0.15	0.15	0.10	0.278	0.55 m	0.56 m

Table 2 – Theoretical Survey Accuracy

The theoretical assessment of accuracy is consistent with IHO Order-1 depth accuracy. The achieved accuracy of soundings is expected not to exceed 0.55 metres (95% confidence) down to depths of 20 metres.

Tenix

Annex H. Summary of Survey Activities

Consecutive Flight No	AS Flight No	GS Sortie No	Date Flown	Engine On	Wheels Up	Galway Bay		Donegal Bay		Tralee Bay		Lough Foyle		Oceano Buoy		Blacksod Bay		Wheels Down	Engine off	Time Lost - System	Time Lost - Weather (Air)	Total Time Lost - Air	On Task	Transit / BM / NAVCAL Time	Total Flight Hours	Airport Holding / Time on Ground	Time Lost - Weather (Ground)	Time Lost - System (Ground)	Remarks
						On Task	Off Task	On Task	Off Task	On Task	Off Task	On Task	Off Task	On Task	Off Task	On Task	Off Task												
1	1192	4	22-May-08	13:33	13:39	13:55<br>15:09	14:09<br>18:23											18:56	18:58			0:00	3:28	1:57	5:25				Initial sortie to Galway Bay, calm sea conditons.
2	1193	5	24-May-08	15:24	15:33	15:16	19:56											21:09	21:11			0:00	4:40	1:07	5:47				Good sortie to Galway Bay.
3	1194	6	25-May-08	15:35	15:43	17:12	17:28	15:35	16:22									18:45	18:48			0:00	1:03	2:10	3:13		3:47		Sortie to Donegal Bay and Galway Bay, strong easterly winds caused turbulence.
4	1195	7	27-May-08	10:01	10:10	10:52	15:39											15:53	15:57			0:00	4:47	1:09	5:56				Sortie to Galway Bay, 100% coverage completed.
5	1196	8,9	29-May-08	9:39<br>16:18	9:48<br>16:22	17:55	18:23			10:25<br>16:59	15:14<br>17:40							15:27<br>18:51	15:31<br>18:57			0:00	5:58	2:33	8:31				Good sortie to Tralee Bay and Galway Bay.
6	1197	10,11	31-May-08	12:34<br>16:38	12:39<br>16:43	16:10	19:53	13:19	14:17	12:17	12:25							15:55<br>21:10	16:00<br>21:15			0:00	4:49	3:14	8:03				Sortie to Tralee, Donegal and Galway, 200% completed.
7	1198	12	1-Jun-08	13:25	13:33	13:17	14:40											15:52	15:57			0:00	1:23	1:09	2:32		4:28		Sortie aborted due to Fog
8	1199	13	2-Jun-08	12:54	13:00	18:01	18:13			13:38	17:45							18:33	18:36			0:00	4:19	1:23	5:42				Sortie to Tralee and Galway Bay.
9	1200	14	3-Jun-08	9:54	10:01	11:39	12:18	10:37	11:02									12:34	12:37			0:00	1:04	1:39	2:43		4:17		Sortie to Donegal Bay and Galway Bay, aborted due to wind / sea state.
10	1201	15	5-Jun-08	16:10	16:18	17:44	21:52							17:30<br>20:26	17:44<br>20:52			22:00	22:07			0:00	4:48	1:09	5:57				Sortie to Galway Bay and Oceanographic Buoy Area
11	1202	16, 17	6-Jun-08	11:55<br>17:33	12:03<br>17:38					12:31<br>18:03	16:33<br>21:14							16:46<br>21:34	16:48<br>21:38			0:00	7:13	1:45	8:58				Two sorties conducted in Tralee Bay
12	1203	18	12-Jun-08	9:40<br>11:50	9:48<br>11:55							12:24	17:18					10:20<br>17:50	10:30<br>17:58			0:00	4:54	2:04	6:58				Transit to Londonderry, refuel. Sortie to Lough Foyle.
13	1204	20	13-Jun-08	11:41<br>17:57	11:47<br>18:02							12:48<br>18:34	16:55<br>20:06					17:19<br>20:39	17:26<br>20:43			0:00	5:39	2:52	8:31				Sortie to Lough Foyle, refuelling stop in Londonderry. Second sortie conducted to Lough Foyle.
14	1205	19	14-Jun-08	12:24	12:34											12:59	19:13	19:30	19:35			0:00	6:14	0:57	7:11				Sortie to Blacksod Bay
																						0:00	0:00	0:00	0:00				
																						0:00	0:00	0:00	0:00				
																						0:00	0:00	0:00	0:00				
																						0:00	0:00	0:00	0:00				
						Total:	23:44	Total:	2:10	Total:	16:58	Total:	10:33	Total:	0:40	Total:	6:14			0:00	0:00	0:00	60:19	25:08	85:27	0:00	12:32	0:00	
						Sorties	10	Sorties	3	Sorties	6	Sorties	3	Sorties	2	Sorties	1												

Annex I Personnel

The following personnel were involved in the collection and processing of all survey data:

Name	Position	Dates
Nigel Townsend	Charge Surveyor (IHO Cat A) / Project Manager / Field Party Leader	18-May-08 – 15-June-08
Ben McWilliam	Flight Coordinator / Surveyor / Data Checker	18-May-08 – 16-June-08
Adam Wiggins	Flight Operator / GIS Analyst / Data Checker	20-May-08 – 11-June-08
Tyson Hillyard	Flight Operator/Coordinator / Surveyor / Geodetics / Data Processor	18-May-08 – 11-June-08
Huw Thomas	Surveyor / Tides Coordinator / Data Processor	13-May-08 – 18-June-08
Ryan Wilkins	Surveyor / Tides Assistant / Data Processor	13-May-08 – 18-June-08
Kirsty Watson	GIS Analyst / Flight Operator/Coordinator / Data Processor	20-May-08 – 15-June-08

Additional support in the Survey Office was provided by:

Mark Sinclair	Survey Program Director
Roderick Curtin	Principal QC Analyst
Tom Gostlow	GIS Analyst / Data Checker
Vince Sicari	GIS Analyst / Data Processor
Georgia Lotsos	GIS Analyst / Data Processor
Phuong Le	Surveyor / Data Processor

Annex J. List of Objects and Obstructions Detected

J.1 Objects and Obstructions

Galway Bay

Object	Point	Surveyed Latitude (ETRF89)	Surveyed Longitude (ETRF89)	Easting	Northing	Run Number	Frame/Row/ Column	Remarks
Fish Farm No 1	1	53˚13.70	9˚42.63	452 566	5 897 907	198.0.1	66/7/2	Fish Farm, appears in video and data, eastings and northings are approximate.
	2	53˚14.70	9˚42.59	452 620	5 897 910	198.0.1	66/18/3	Fish Farm, appears in video and data, eastings and northings are approximate.
	3	53˚13.67	9˚42.63	452 567	5 897 854	196.0.1	322/18/18	Fish Farm, appears in video and data, eastings and northings are approximate.
	4	53˚13.67	9˚42.59	452 618	5 897 855	196.0.1	322/8/18	Fish Farm, appears in video and data, eastings and northings are approximate.
	5	53˚13.64	9˚42.63	452 565	5 897 799	196.0.1	322/18/29	Fish Farm, appears in video and data, eastings and northings are approximate.
	6	53˚13.64	9˚42.58	452 620	5 897 803	195.0.1	318/7/8	Fish Farm, appears in video, eastings and northings are approximate.
	7	53˚13.61	9˚42.63	452 567	5 897 743	196.0.1	322/17/40	Fish Farm, appears in video and data, eastings and northings are approximate.

Object	Point	Surveyed Latitude (ETRF89)	Surveyed Longitude (ETRF89)	Easting	Northing	Run Number	Frame/Row/ Column	Remarks
	8	53˚13.61	9˚42.59	452 618	5 897 748	196.0.1	322/7/39	Fish Farm, appears in video, eastings and northings are approximate.
Fish Farm No 2	1	53˚14.92	9˚43.56	451 562	5 900 183	53.1.1	74/2/46	Fish Farm, appears in video. Outside of survey area, located at end of x-tie.
Fish Farm No 3	1	53˚15.11	9˚43.57	451 555	5 900 539	53.0.1	70/17/48	Fish Farm, appears in video. Outside of survey area, located at end of x-tie.

Lough Foyle

Object	Point	Surveyed Latitude (ETRF89)	Surveyed Longitude (ETRF89)	Easting	Northing	Run Number	Frame/Row/ Column	Remarks
Charted Obstruction No 1	1	55˚13.00	6˚56.29	631 170	6 120 846	505.0.1	44/14/31	Obstruction confirmed, Recommend update position to survey position
	2	55˚12.99	6˚56.30	631163	6120828	505.0.1	44/17/33	Obstruction confirmed, Recommend update position to survey position
Charted Obstruction No 2	1	55˚10.38	7˚03.34	623828	6115770	512.0.1	144/6/10	Obstruction confirmed, Recommend update position to survey position

Fish Farm – Galway Bay Area

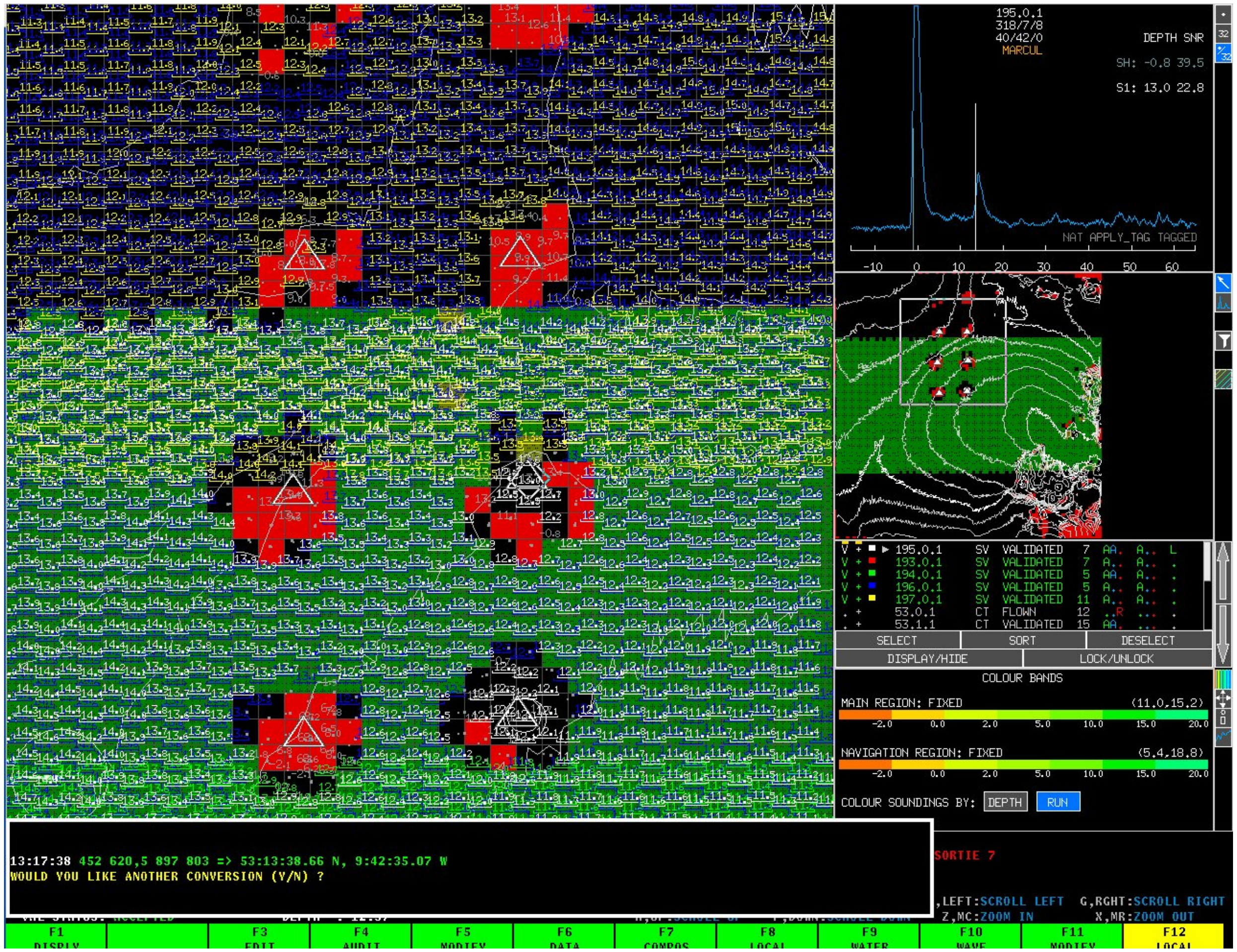

Charted Obstruction No 1

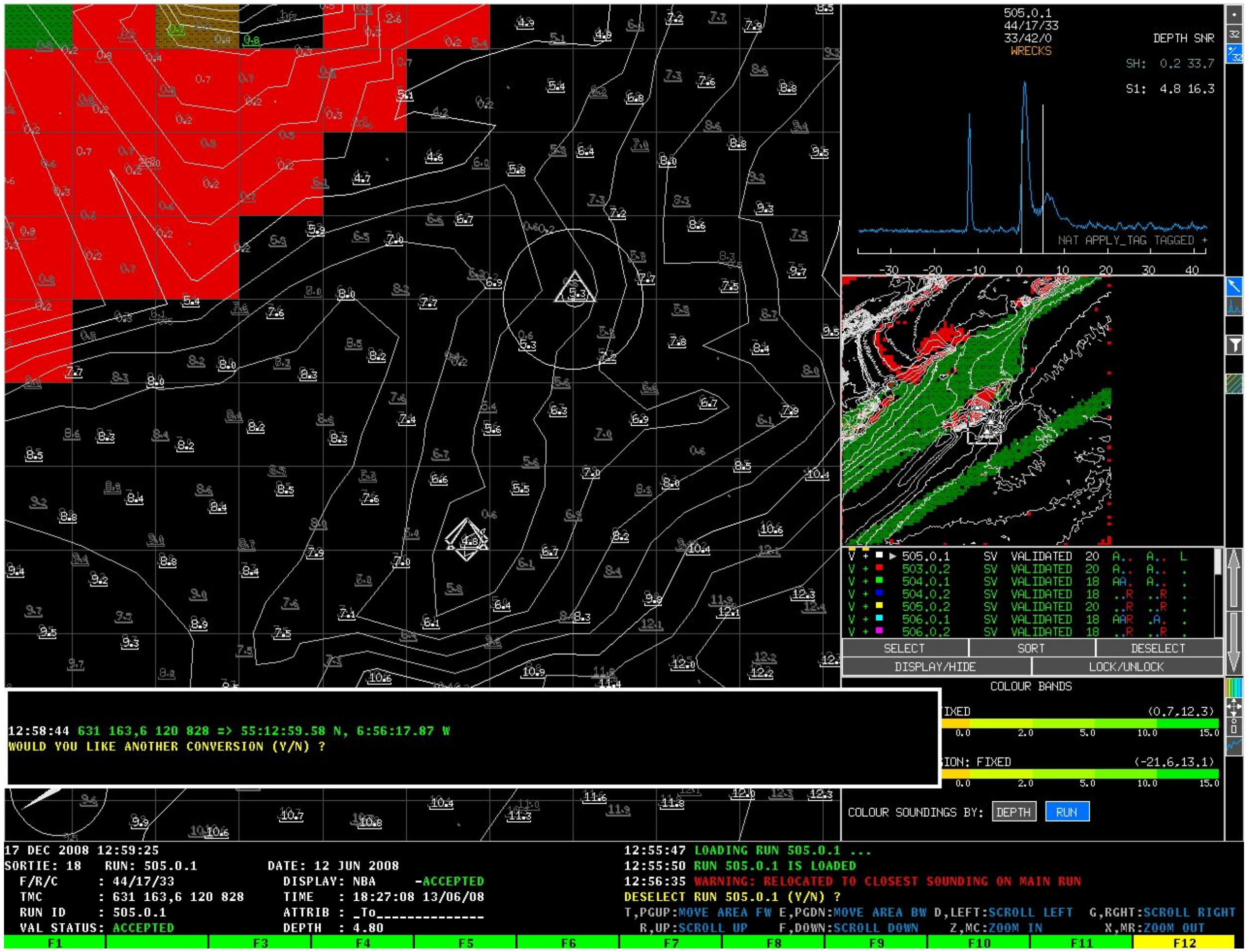

Charted Obstruction No 2

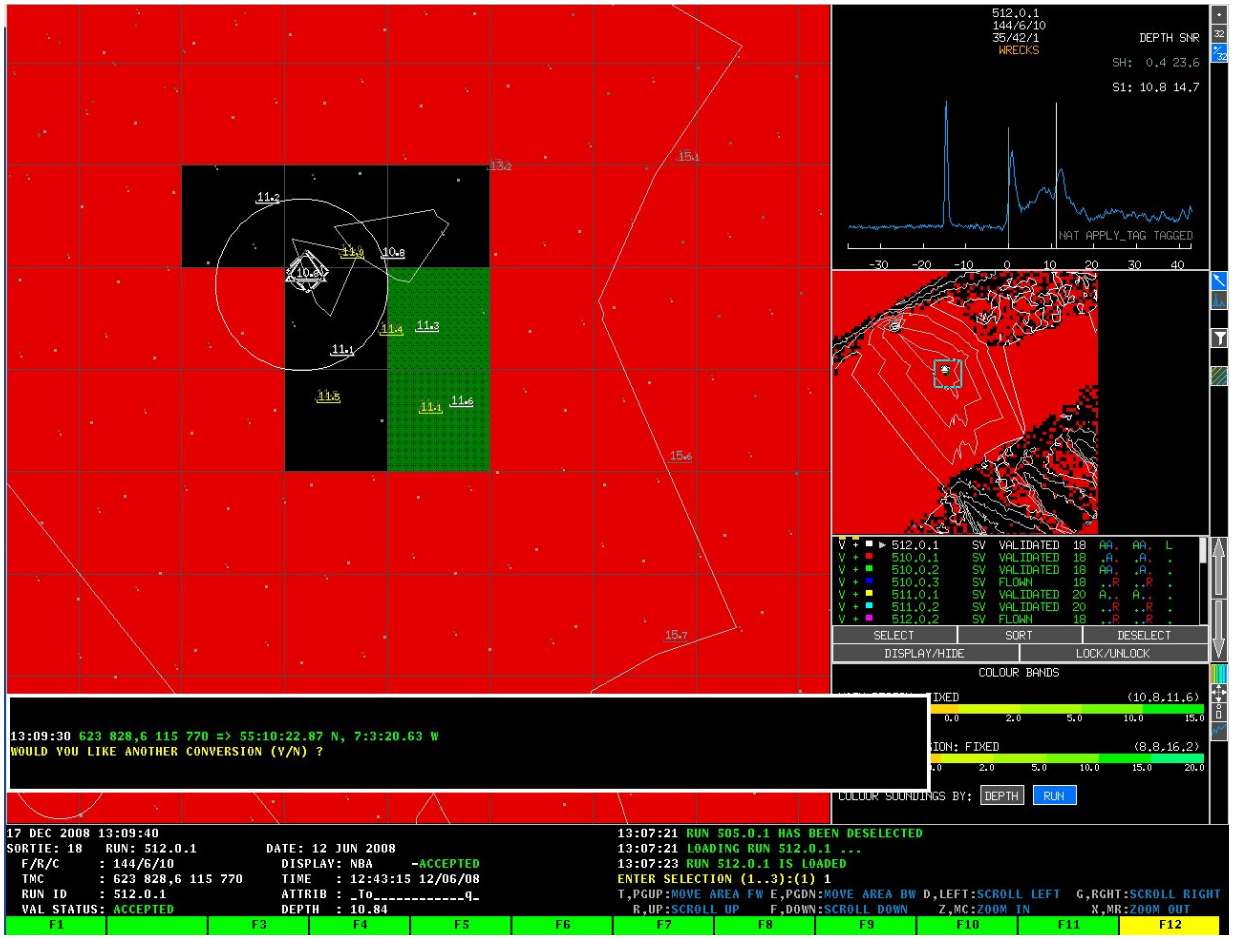

Annex K. List of Buoys and Beacons

K.1 Beacons

The following beacons were detected by the system and have been retained in the dataset.

Easting	Northing	Latitude (N)	Longitude (W)	Depth	Line	Frame	Row	Column	Remarks
452164.1	5898551.9	53º14.046	009º43.001	-6.27	204.0.1.1	389	15	36	Kiggaul Bay Light (NW Coast Galway Bay)
497889.3	5900682.9	53º15.324	009º01.898	-12.47	140.1.1.1	106	4	7	Leverets Tower, North Bay, Galway Bay
455745.7	5884356.5	53º06.409	009º39.664	-4.71	266.0.1.6	169	6	40	Beacon, Killeany Bay, Inishmore Island
455702.1	5884450.3	53º06.459	009º39.704	-2.13	266.0.1.6	170	6	27	Beacon, Killeany Bay, Inishmore Island
455673.0	5884322.3	53º06.390	009º39.729	-2.19	268.0.1.2	152	7	38	Beacon, Killeany Bay, Inishmore Island
455732.4	5884300.7	53º06.378	009º39.675	-2.48	268.0.1.2	153	2	40	Beacon, Killeany Bay, Inishmore Island
455741.6	5884230.9	53º06.341	009º39.666	-5.03	268.0.1.2	153	9	28	Beacon, Killeany Bay, Inishmore Island
631144.7	6120869.6	55º13.016	006º56.314	-5.56	504.0.1.2	42	11	46	Metal Man Beacon, North Channel Lough Foyle
625098.4	6116929.8	55º10.987	007º02.117	-13.28	507.0.1.1	124	1	42	Moville Beacon, Moville Bank's Lough Foyle
619884.9	6113057.8	55º08.978	007º07.125	-5.73	515.0.1.5	198	8	12	Redcastle NE Beacon, Lough Foyle
619347.8	6112652.9	55º08.767	007º07.641	-5.56	515.0.1.5	205	13	27	Redcastle Beacon, Lough Foyle
618309.3	6112145.5	55º08.509	007º08.631	-4.98	515.0.1.5	218	6	8	Argus Beacon, Lough Foyle
617259.5	6111383.6	55º08.113	007º09.637	-5.03	515.0.1.5	232	10	31	The Perch Beacon, Lough Foyle
618424.0	6111988.0	55º08.423	007º08.527	-4.75	516.0.1.2	217	5	27	Drung Beacon, Lough Foyle
629382.9	6118453.8	55º11.742	006º58.041	-4.99	517.0.1.1	73	14	20	Magiligan Beacon, Lough Foyle
619117.2	6112305	55º08.583	007º07.867	-5.3	517.0.1.3	209	15	23	Vances Beacon, Lough Foyle
625699.7	6115935.8	55º10.442	007º01.577	-5.45	519.0.1.1	121	6	33	Glenburnie Beacon, McKinney's Bank, Lough Foyle

Easting	Northing	Latitude (N)	Longitude (W)	Depth	Line	Frame	Row	Column	Remarks
617467.0	6111150.7	55º07.985	007º09.448	-4.73	519.0.1.1	228	17	12	White Castle Beacon, Lough Foyle
616338.5	6110342.9	55º07.566	007º10.529	-5.66	519.0.1.1	244	5	36	Carrowkeel Beacon, Lough Foyle
615545.2	6109418.2	55º07.078	007º11.298	-4.66	523.0.1.1	246	10	34	Cabry Beacon, Lough Foyle
615686.6	6109335.1	55º07.032	007º11.167	-5.71	525.0.1.1	247	8	22	Quigleys Beacon, Lough Foyle
614869.6	6108159.3	55º06.409	007º11.963	-4.61	530.0.1.1	254	5	41	Lepers Point Beacon, Lough Foyle
614727.3	6108255.2	55º06.463	007º12.095	-5.43	530.0.1.1	255	5	9	Greenbank Beacon, Lough Foyle
613886.2	6107081.6	55º05.842	007º12.914	-4.68	536.0.1.2	39	4	47	Aught Beacon, Lough Foyle
614074.0	6106959.5	55º05.774	007º12.740	-5.36	536.0.1.2	40	1	4	Ture Beacon, Lough Foyle
613722.5	6106595.3	55º05.582	007º13.079	-5.19	538.0.1.2	35	12	19	Drumskellan Beacon, Lough Foyle
613849.4	6106560.8	55º05.562	007º12.961	-5.04	540.0.1.2	40	6	41	Longfield Beacon, Lough Foyle
613454.5	6105589.1	55º05.044	007º13.355	-4.96	546.0.1.1	26	15	35	Claddagh Beacon, Lough Foyle
613240.7	6104907.0	55º04.679	007º13.572	-4.8	551.0.1.2	18	18	38	Crummin Beacon, Lough Foyle
613359.4	6104858.2	55º04.651	007º13.462	-5.13	552.0.1.2	20	2	36	Willsboro Beacon, Lough Foyle

K.2 Buoy

The following buoys were observed in the video, but have been removed from the datasets.

Easting	Northing	Latitude (N)	Longitude (W)	Depth	Line	Frame	Row	Column	Remarks
431391.8	5994955.0	54º05.891	010º02.949	17.31	1923.0.1.1	14	3	16	Blacksod Bay East Cardinal Buoy
495262.9	5898759.6	53º14.286	009º04.259	13.72	133.0.1.1	68	4	27	Cockle Rock North Cardinal Buoy , North Bay, Galway Bay
498797.1	5900375.5	53º15.158	009º01.082	9.28	133.0.1.3	111	2	14	Peter Rock South Cardinal Buoy , North Bay, Galway Bay
627270.1	6117543.1	55º11.284	007º00.055	14.14	513.0.1.1	98	15	37	Mckinney's Buoy, Lough Foyle
622034.2	6114911.4	55º09.945	007º05.055	6.59	509.0.1.1	165	2	35	Clare Buoy, Lough Foyle
622041.0	6114898.5	55º09.938	007º05.049	5.43	509.0.1.1	165	2	38	Clare Buoy, Lough Foyle
624047.9	6116029.7	55º10.518	007º03.130	13.49	511.0.1.1	140	2	9	Saltpans Buoy, Lough Foyle

Annex L. Daily Survey Logs

Survey Title : Ireland_07 **Database :**

Date : 13-May-08 **Julian Day :** 134

Weather in Survey Area

Cloud :	3	*octas*	High	*feet*	Sea State :	1-2	
Wind :	E	*degrees*	10 to 15	*speed*	Visibility :	Good	
Swell :	SW	*degrees*	0.5	*metres*	Temperature :	15 to 22	°C

Weather Comments: Slightly cool conditions in the afternoon with an easterly breeze evident.

General Comments:

Field Team: Total: 2 Survey: 2 Other:

Survey: HDT, RJW

Other:

Remarks:

Boat Operations: Vessel Name: Consecutive Trip:

Departure Time : Return Time: Sail Time:

On Task : Off Task: Time on task:

Weather Downtime : - Technical Downtime: Boat Downtime:

Crew:

Boat Activities:

Remarks / Defects : -

Survey Issues:

**Field Team Stats:**	**Boat Ops (hours):**	Daily	Cumul.	**Processing (Hours):**	Daily	Cumul.	**Other (Hr):** Setup	Daily	Cumul.

Equipment / Logistics

Remarks :

Narrative: Tides party arrived in Galway approximately 1500 and transited to Mullaghamore Harbour. Tide equipment not yet delivered, expected to arrive on the 14th.

24hr Forecast: Day Off

Survey Title : Ireland_07 **Database :**

Date : 14-May-08 **Julian Day :** 135

Weather in Survey Area

Cloud :	2	*octas*	High	*feet*	Sea State :	1-2	
Wind :	E	*degrees*	5 to 10	*speed*	Visibility :	Good	
Swell :	SW	*degrees*	0.5	*metres*	Temperature :	15 to 25	°C

Weather Comments: Cool and calm in the morning, winds slightly strengthening in the afternoon

General Comments: Day Off

Field Team: Total: 2 Survey: 2 Other:

Survey: HDT, RJW

Other:

Remarks:

Boat Operations: Vessel Name: Consecutive Trip:

Departure Time : Return Time: Sail Time:

On Task : Off Task: Time on task:

Weather Downtime : - Technical Downtime: Boat Downtime:

Crew:

Boat Activities:

Remarks / Defects : -

Survey Issues:

**Field Team Stats:**	**Boat Ops (hours):**	Daily	Cumul.	**Processing (Hours):**	Daily	Cumul.	**Other (Hr):** Setup	Daily	Cumul.

Equipment / Logistics

Remarks :

Narrative: Rest day. Tide equipment still not delivered. Contact made with Shenkers Logistics, equipment to be delivered around midday on the 15th

24hr Forecast: Take delivery of tide equipment, deploy bottom mounted gauges in Donegal Bay

Survey Title : Ireland_07 **Database :**

Date : 15-May-08 **Julian Day :** 136

Weather in Survey Area

Cloud :	3	*octas*	High	*feet*	Sea State :	1-2	
Wind :	E	*degrees*	10 to 15	*speed*	Visibility :	Good	
Swell :	SW	*degrees*	0.5	*metres*	Temperature :	15 to 22	°C

Weather Comments: Similar conditions to yesterday, calm in the morning with the easterly breeze strengthening in the evening

General Comments: Boat operations suspended, not all tide equipment delivered

Field Team: Total: 2 Survey: 2 Other:

Survey: HDT, RJW

Other:

Remarks:

Boat Operations: Vessel Name: Consecutive Trip:

Departure Time : Return Time: Sail Time:

On Task : Off Task: Time on task:

Weather Downtime : - Technical Downtime: Boat Downtime:

Crew:

Boat Activities:

Remarks / Defects : -

Survey Issues:

**Field Team Stats:**	**Boat Ops (hours):**	Daily	Cumul.	**Processing (Hours):**	Daily	Cumul.	**Other (Hr):** Setup	Daily	Cumul.
								20	20

Equipment / Logistics

Remarks :

Narrative: Bottom mounted gauges missing from equipment delivered to the hotel. BMTG's to be couriered to Mullaghamore by midday on the 16th. Boat rescheduled for 1400 on the 16th

24hr Forecast: Deploy bottom mounted gauges in Donegal Bay, assemble Wharf mounted gauge in Mullaghamore Harbour and commence 25hr observation session.

Survey Title : Ireland_07 **Database :**

Date : 16-May-08 **Julian Day :** 137

Weather in Survey Area

Cloud :	6	octas	High	feet	Sea State :	1-2	
Wind :	E-SW	degrees	10 to 15	speed	Visibility :	Good	
Swell :	SW	degrees	0.5	metres	Temperature :	15 to 22	°C

Weather Comments: calm easterly breeze in the morning with winds slightly strengthening and swinging to a south-westerly in the afternoon

General Comments: BMTG's deployed, 25Hr comparison initiated in Mullaghamore Harbour

Field Team: Total: 2 Survey: 2 Other:

Survey: HDT, RJW

Other:

Remarks:

Boat Operations: Vessel Name: Prospector 1 Consecutive Trip:

Departure Time : 1400 Return Time: 1745 Sail Time: 3hr 45m

On Task : Off Task: Time on task:

Weather Downtime : - Technical Downtime: Boat Downtime:

Crew: Peter Power + deckhand, HDT, RJW

Boat Activities: Deploy BMTG's in Donegal Bay

Remarks / Defects : -

Survey Issues:

**Field Team Stats:**	**Boat Ops (hours):**	Daily	Cumul.	**Processing (Hours):**	Daily	Cumul.	**Other (Hr):**	Daily	Cumul.
		8					Setup	12	32

Equipment / Logistics

Remarks :

Narrative: Bottom mounted gauges picked up from courier in Sligo.
Tides party rendezvoused with Skipper of "Prospector 1" at 1400 to deploy BMTG'S in Donegal Bay. A Valeport Wharf mounted gauge was then setup in Mullaghamore harbour with 25hr observations beginning at 1900.

24hr Forecast: Complete 25hr observation session, conduct tide gauge datum connection.

Survey Title : Ireland_08 **Database :**

Date : 17-May-08 **Julian Day :** 138

Weather in Survey Area

Cloud :	7	octas	High	feet	Sea State :	1-2	
Wind :	E	degrees	10 to 15	speed	Visibility :	Good	
Swell :	SW	degrees	0.5	metres	Temperature :	10-15	°C

Weather Comments: Cool cloudy conditions throughout the day, easterly breeze strengthing in the evening

General Comments: complete 25hr comparsion and datum connection

Field Team: Total: 2 Survey: 2 Other:

Survey: HDT, RJW

Other:

Remarks:

Boat Operations: Vessel Name: Consecutive Trip:

Departure Time : Return Time: Sail Time:

On Task : Off Task: Time on task:

Weather Downtime : - Technical Downtime: Boat Downtime:

Crew:

Boat Activities:

Remarks / Defects :

Survey Issues:

**Field Team Stats:**	**Boat Ops (hours):**	Daily	Cumul.	**Processing (Hours):**	Daily	Cumul.	**Other (Hr):** Setup	Daily	Cumul.
								20	52

Equipment / Logistics

Remarks :

Narrative: 25hr pole - gauge comparion continued throughout the day concluding at approximately 2100.A benchmark connection was also carried out during the afternoon.

24hr Forecast: Transit to Mount Charles pier to conduct 25 hr datum connection to BMTG's placed in Donegal Bay

DAILY SURVEY LOG

Survey Title : Ireland 2008 **Database :** 08_07NW Ireland

Date : Sunday 18 May 2008 **Julian Day :** 188

Weather in Survey Area

Cloud : 5/8 *octas* 2000 *feet* Sea State : 1

Wind : ESE *degrees* 16 *speed* Visibility : good

Swell : *degrees* *metres* Rain: < 5mm

Weather Comments: Overcast, cool, passing showers during afternoon

General Comments: Waiting for freight, LCL, access to airfield. attempting to conduct gauge/gauge comparison at Donegal Harbour.

Data Collection Cons flight: Sortie Number : AS GS

Time On Task : Downtime Env Downtime Sys

Flight Time: Aircraft (Weather Holding / Aircraft AOG):

Crew: Pilot Duty Hours:

Navcals : Pilot Flying Hours:

Benchmarks :

Survey Lines :

Remarks :

Progress:

No Planned flight - aircraft still in Saudi

**Environmental Relies Flown:**	**Cloud:**	Daily	Cumul.	**Swell:**	Daily	Cumul.	**Coverage:**	Daily	Cumul.
		0	0		0	0		0	0
**System Reflies Flown:**	**KF - Tilt:**	Daily	Cumul.	**Sea Surface:**	Daily	Cumul.	**Other Cause:**	Daily	Cumul.
		0	0		0	0		0	0

Survey Issues

Data Processing:

Attempted 25 Hr Gauge/Gauge comparison at Salt Hill Pier to connect BMTG to Marlin Hd Datum, unable to secure gauge or pole, returned to Galway whilst permission is obtained to attach clamps to wharf.

**Survey Team Stats:**		**Flying Ops (hours):**	Daily	Cumul.	**Pre Val (Hours):**	Daily	Cumul.	**Validation (Hours):**	Daily	Cumul.
			0	0		0	0		0	
**Survey Team**	**Nº**	**GPS / Tides (Hours):**	Daily	Cumul.	**Planning (Hours):**	Daily	Cumul.	**Other (Hours):**	Daily	Cumul.
	5		20	72		10	10	*Da y off*	20	395

Equipment / Logistics (to include aircraft and airborne system defects / changes)

Remarks : Obtained access to Field Office, set up in preparation for arrival of equipment.

**Laser SRL No:**	2001	**Green RX SRL No:**	2002

Narrative: (Customer/Personnel related issues & general comments)

A quite day due to delayed arrival of LCL from Saudi. Unable to get access to freight in Galway until Monday, Tides party returned to Galway John Sincock arrived in Field.

24hr Forecast: Receive freight, coordinate GPS base station, check marks Gain access to airfield

DAILY SURVEY LOG

Survey Title : Ireland 2008 **Database :** 08_07NW Ireland

Date : Monday 19 May 2008 **Julian Day :** 189

Weather in Survey Area

Cloud :	6/8	octas		feet	Sea State :	3
Wind :	SSE	degrees	15 -22	speed	Visibility :	good
Swell :	nil	degrees	Nil	metres		

Weather Comments: Overcast, winds developing from SSE, passing showers developing.

General Comments: Chasing freight, local surveyor conducting coordination of GPS base station and airport check marks.

Data Collection Cons flight: Sortie Number : AS GS

Time On Task : Downtime Env Downtime Sys

Flight Time: Aircraft (Weather Holding / Aircraft AOG):

Crew: Pilot Duty Hours:

Navcals : Pilot Flying Hours:

Benchmarks :

Survey Lines :

Remarks : LCL on Transit from Saudi

Progress:

Environmental Relies Flown:	Cloud:	Daily	Cumul.	Swell:	Daily	Cumul.	Coverage:	Daily	Cumul.
		0	0		0	0		0	0
System Reflies Flown:	KF - Tilt:	Daily	Cumul.	Sea Surface:	Daily	Cumul.	Other Cause:	Daily	Cumul.
		0	0		0	0		0	0

Survey Issues

Data Processing:

GPS freight still not available. Local surveyor used to coordinate GSP Base station and Check Marks

Tides Party day off

Survey Team Stats:		Flying Ops (hours):	Daily	Cumul.	Pre Val (Hours):	Daily	Cumul.	Validation (Hours):	Daily	Cumul.
			0	0		0	0		0	40
Survey Team	Nº	GPS / Tides (Hours):	Daily	Cumul.	Planning (Hours):	Daily	Cumul.	Other (Hours):	Daily	Cumul.
	5		20	92		10	20	Da y off	30	70

Equipment / Logistics (to include aircraft and airborne system defects / changes)

Remarks : Freight still not available. Logistics manager arrived in country.

**Laser SRL No:**	2001	**Green RX SRL No:**	2002

Narrative: (Customer/Personnel related issues & general comments)

Freight located in Shannon, cleared customs but cannot be delivered until tomorrow.

Gavin Siviour, 1 x LAME, Adam Wiggins arrived in field

24hr Forecast: LCL due to arrive, freight due to be delivered.

Strong Winds (25 to 40 knots) and rain (30mm) forecast for next two days.

DAILY SURVEY LOG

Survey Title : Ireland 2008 **Database :** 08_07NW Ireland

Date : Tuesday 20 May 2008 **Julian Day :** 190

Weather in Survey Area

Cloud : 6/8 octas feet Sea State : 3
Wind : SSE degrees 15 -22 speed Visibility : Moderate
Swell : nil degrees Nil metres

Weather Comments: Overcast, winds developing from SSE, passing showers developing.

General Comments: Install Tide station at Rossaveel, receive LCL on arrival establish GPS Base station.

Data Collection Cons flight: Sortie Number : AS GS

Time On Task : Downtime WX Downtime Sys

Flight Time: Aircraft (Weather Holding / Aircraft AOG):

Crew: Pilot Duty Hours: 11:00

Navcals : Pilot Flying Hours:

Benchmarks :

Survey Lines :

LCL on Transit from Saudi

Remarks / Defect

Progress: LCL arrived in Galway at 1600 local

Environmental Reflies Flown:	Cloud:	Daily	Cumul.	Swell:	Daily	Cumul.	Coverage:	Daily	Cumul.
		0	0		0	0		7	7
System Reflies Flown:	KF - Tilt:	Daily	Cumul.	Sea Surface:	Daily	Cumul.	Other Cause:	Daily	Cumul.
		0	0		0	0	Platform	3	3

Survey Issues

Data Processing:

Tide gauge at Rossaveel established, commenced 25 Hr Comparison.
GPS Base station established on top of Hotel, commenced 16 Hr check obs.

Survey Team Stats:		Flying Ops (hours):	Daily	Cumul.	Pre Val (Hours):	Daily	Cumul.	Validation (Hours):	Daily	Cumul.
			0	0		0	0		0	40
Survey Team	Nº 7	GPS / Tides (Hours):	Daily 40	Cumul. 132	Planning (Hours):	Daily 10	Cumul. 30	Other (Hours): Travel / DO	Daily 20	Cumul. 90

Equipment / Logistics (to include aircraft and airborne system defects / changes)

Remarks : AS Spares arrived in Galway. LCL arrived in Galway.
Field office set up with the exception of Bilbo (Gandlaf lost in freight)

Laser SRL No: 2001 **Green RX SRL No:** 2002

Narrative: (Customer/Personnel related issues & general comments)

Survey infrastructure now in place to commence survey operations.
LCL arrived in Galway as planned, operations at airport difficult due to number of aircraft movements and busy airport staff.

24hr Forecast: Conduct Static Position checks, complete 25 hour obs.
Weather forecast remain marginal.

DAILY SURVEY LOG

Survey Title : Ireland 2008 **Database :** 08_07NW Ireland

Date : Wednesday 21 May 2008 **Julian Day :** 191

Weather in Survey Area

Cloud : 8/8 octas — feet — Sea State : 3
Wind : SSE degrees — 20 - 30 speed — Visibility : Poor
Swell : nil degrees — Nil metres

Weather Comments: Overcast, strong from SSE, passing showers and rain.

General Comments: Pilot Rest Day, Conduct static GPS Checks, Complete office set up.

Data Collection Cons flight: Sortie Number : AS GS

Time On Task : Downtime WX Downtime Sys

Flight Time: Aircraft (Weather Holding / Aircraft AOG):

Crew: Pilot Duty Hours:

Navcals : Pilot Flying Hours:

Benchmarks :

Survey Lines :

LCL on Transit from Saudi

Remarks / Defect Progress:

**Environmental Reflies Flown:**	**Cloud:**	Daily	Cumul.	**Swell:**	Daily	Cumul.	**Coverage:**	Daily	Cumul.		
		0	0		0	0		9	16		
**System Reflies Flown:**	**KF - Tilt:**	Daily	Cumul.	**Sea Surface:**	Daily	Cumul.	**Other Cause:**	Daily	Cumul.		
		0	0		0	0	Platform	1	4		

Survey Issues

Data Processing:

Completed 25 Hour comparison at Rossaveel; Completed static GPS check.

**Survey Team Stats:**		**Flying Ops (hours):**	Daily	Cumul.	**Pre Val (Hours):**	Daily	Cumul.	**Validation (Hours):**	Daily	Cumul.
			0	0		0	0		0	40
**Survey Team**	**Nº**	**GPS / Tides (Hours):**	Daily	Cumul.	**Planning (Hours):**	Daily	Cumul.	**Other (Hours):**	Daily	Cumul.
	7		50	182		10	40		10	100

Equipment / Logistics (to include aircraft and airborne system defects / changes)

Remarks : Bilbo set to work. Still missing large amount of stores.

**Laser SRL No:**	2001	**Green RX SRL No:**	2002

Narrative: (Customer/Personnel related issues & general comments)

checks on Tidal network and GPS base systems completed.
Operations at airport remain difficult, hard to get time with airport management.

24hr Forecast: Afternoon flight to Galway Bay, Donegal Bay
Weather forecast is not good, flight may be cancelled.

DAILY SURVEY LOG

Survey Title : Ireland 2008 **Database :** 08_07NW Ireland

Date : Thursday 22 May 2008 **Julian Day :** 143

Weather in Survey Area

Cloud :	3	octas	1500	feet	Sea State :	0/1
Wind :	Var	degrees	<5	speed	Visibility :	Moderate
Swell :	NE	degrees	0.5	metres	Rain:	yes

Weather Comments: Heavy showers and low cloud in area, light and variable winds, heavy low cloud and mist in Donegal Bay

General Comments: Initial shakedown flight to Galway and Donegal Bay

Data Collection Cons flight: 1 Sortie Number : AS 1192 GS 22

Time On Task : 2:28 Downtime WX 1:00 Downtime Sys Nil

Flight Time: 5:51 Aircraft (Weather Holding / Aircraft AOG): Nil

Crew: PJ, DH, NAT, BCM, AMW Pilot Duty Hours: 7:30

Navcals : 1 Pilot Flying Hours: 5:51

Benchmarks : 2

Survey Lines : Galway - 27 x mainline, 2 x refly lines (calm seas)

Remarks / Defects : Tape Dive B U/S, repaired on return to Galway

Progress: Commenced Galway Area

Environmental Reflies Flown:	Cloud:	Daily	Cumul.	Swell:	Daily	Cumul.	Coverage:	Daily	Cumul.
		0	0		0	0		0	0
System Reflies Flown:	KF - Tilt:	Daily	Cumul.	Sea Surface:	Daily	Cumul.	Other Cause:	Daily	Cumul.
		0	0		0	0	Calm Cond.	2	2

Survey Issues

Data Processing:

Tide Party departed to install a gauge at Loch Foyle.

GPS data processed and checked.

Survey Team Stats:		Flying Ops (hours):	Daily	Cumul.	Pre Val (Hours):	Daily	Cumul.	Validation (Hours):	Daily	Cumul.
			30	30		0	0			40
Survey Team	N°	GPS / Tides (Hours):	Daily	Cumul.	Planning (Hours):	Daily	Cumul.	Other (Hours):	Daily	Cumul.
	7		30	212		10	50			100

Equipment / Logistics (to include aircraft and airborne system defects / changes)

Remarks : GPS, Media Freight turned up

DLT Drive B developed a fault on boot up, rectified on return, Drive A worked.

Laser SRL No: 2001 **Green RX SRL No:** 2002

Narrative: (Customer/Personnel related issues & general comments)

A semi-Successful first flight, rain and calm conditions made operations difficult. Some good data collected to 20+ metres in parts in Galway Bay and on the north side of Aran Islands.

24hr Forecast: No planned flying due to forecast low cloud and calm conditions. Establish tide gauge at Loch Foyle.

DAILY SURVEY LOG

Survey Title : Ireland 2008 **Database :** 08_07NW Ireland

Date : Friday 23 May 2008 **Julian Day :** 144

Weather in Survey Area

Cloud :	6/8	*octas*	1500	*feet*	Sea State :	1
Wind :	ENE	*degrees*	5	*speed*	Visibility :	Moderate
Swell :	Nil	*degrees*	-	*metres*	Rain:	Nil

Weather Comments: Calm conditions in between passing showers

General Comments: No Planned flight due to calm conditions and need to preserve Pilot Hours for Neap tide period.

Data Collection Cons flight: Sortie Number : AS GS

Time On Task : Downtime Envir Downtime Sys

Flight Time: Aircraft (Weather Holding / Aircraft AOG):

Crew: Pilot Duty Hours:

Navcals : Pilot Flying Hours:

Benchmarks :

Survey Lines : No Planned flight

Remarks / Defect

Progress:

Environmental Reflies Flown:	Cloud:	Daily	Cumul.	Swell:	Daily	Cumul.	Coverage:	Daily	Cumul.
		0	0		0	0		0	0
System Reflies Flown:	KF - Tilt:	Daily	Cumul.	Sea Surface:	Daily	Cumul.	Other Cause:	Daily	Cumul.
		0	0		0	0		0	2

Survey Issues

Data Processing:

Preval sortie 4, process GPS static Check results.

Tides Party installed Tide gauge at LondonDerry

Survey Team Stats:		Flying Ops (hours):	Daily	Cumul.	Pre Val (Hours):	Daily	Cumul.	Validation (Hours):	Daily	Cumul.
			0	30		30	30			40
Survey Team	Nº	GPS / Tides (Hours):	Daily	Cumul.	Planning (Hours):	Daily	Cumul.	Other (Hours):	Daily	Cumul.
	7		30	242		10	60	*Travel*	10	110

Equipment / Logistics (to include aircraft and airborne system defects / changes)

Remarks : Nil issues

Laser SRL No:	2001	Green RX SRL No:	2002

Narrative: (Customer/Personnel related issues & general comments)

Non flying day, marginal conditions, Pilot rest day to guarantee ops over neap tide period.

UK Student Oliver Wilson arrived on site.

24hr Forecast: Afternoon flight to Galway Bay.

DAILY SURVEY LOG

Survey Title : Ireland 2008 **Database :** 08_07NW Ireland

Date : Saturday 24 May 2008 **Julian Day :** 145

Weather in Survey Area

Cloud :	3	octas	5000	feet	Sea State :	3
Wind :	NE	degrees	25	speed	Visibility :	Haze
Swell :	90	degrees	1.5	metres	Rain:	Nil

Weather Comments: Strong breeze from the NE, poor visibility especially during morning, improved dur9ing afternoon.

General Comments: Planned afternoon flight to Galway Bay

Data Collection Cons flight: 2 Sortie Number : AS 1193 GS 5

Time On Task : 4:40 Downtime WX Nil Downtime Sys Nil

Flight Time: 5:47 Aircraft (Weather Holding / Aircraft OG):

Crew: PJ, DH, BCM, AMW, Oliva Wilson Pilot Duty Hours: 6:15

Navcals : 1 Pilot Flying Hours: 5:47

Benchmarks : 2

Survey Lines : Galway - 31 x mainline, 3 x cross lines

Remarks / Defects : Nil Defects

Progress: Galway Bay - 40% flown

Environmental Reflies Flown:	Cloud:	Daily	Cumul.	Swell:	Daily	Cumul.	Coverage:	Daily	Cumul.
		0	0		0	0		0	0
**System Reflies Flown:**	**KF - Tilt:**	Daily	Cumul.	**Sea Surface:**	Daily	Cumul.	**Other Cause:**	Daily	Cumul.
		0	0		0	0		0	2

Survey Issues

Data Processing:

Processing Sortie 4

Conducting Grafnav logging trial

Survey Team Stats:		Flying Ops (hours):	Daily	Cumul.	Pre Val (Hours):	Daily	Cumul.	Validation (Hours):	Daily	Cumul.
			20	50		10	40			40
Survey Team	Nº 7	GPS / Tides (Hours):	Daily 20	Cumul. 262	Planning (Hours):	Daily	Cumul. 60	Other (Hours): Day off	Daily 20	Cumul. 130

Equipment / Logistics (to include aircraft and airborne system defects / changes)

Remarks : Nil issues, LAME change over

Laser SRL No: 2001 **Green RX SRL No:** 2002

Narrative: (Customer/Personnel related issues & general comments)

A good flight to Galway Bay, good water conditions, Aran Islands completed at 100%, good progress made on Galway north coast coverage.

24hr Forecast: Afternoon flight to Donegal / Galway Bay

DAILY SURVEY LOG

Survey Title : Ireland 2008 **Database :** 08_07NW Ireland

Date : Sunday 25 May 2008 **Julian Day :** 146

Weather in Survey Area

Cloud :	4	*octas*	2000	*feet*	Sea State :	3
Wind :	ENE	*degrees*	30	*speed*	Visibility :	Good
Swell :		*degrees*	0.5	*metres*	Rain:	Nil

Weather Comments: Weather conditions fine, mostly clear, strong ENE caused turbulence in all survey areas

General Comments: Planned afternoon flight to Donegal / Galway Bay

Data Collection Cons flight: 3 Sortie Number : AS 1194 GS 6

Time On Task : 1:03 Downtime WX 3:47 Downtime Sys

Flight Time: 3:13 Aircraft (Weather Holding / Aircraft AOG):

Crew: PJ, DH, BCM, AMW Pilot Duty Hours: 4:45

Navcals : 1 Pilot Flying Hours: 3:13

Benchmarks : 2 x Donegal

Survey Lines : Donegal Bay - 6 Main lines
Galway Bay - 2 Mainlines

Remarks / Defects : Flight aborted due to Strong Easterly winds.

Progress: Galway Bay - 40% flown

**Environmental Reflies Flown:**	**Cloud:**	Daily	Cumul.	**Swell:**	Daily	Cumul.	**Coverage:**	Daily	Cumul.
		0	0		0	0		0	0
**System Reflies Flown:**	**KF - Tilt:**	Daily	Cumul.	**Sea Surface:**	Daily	Cumul.	**Other Cause:**	Daily	Cumul.
		0	0		0	0		0	2

Survey Issues
Data Processing:

Preval of Sortie 5

Conducted Boat Recce of Lough Foyle, strong easterly winds, reduced water clarity from previous recces.

**Survey Team Stats:**		**Flying Ops (hours):**	Daily	Cumul.	**Pre Val (Hours):**	Daily	Cumul.	**Validation (Hours):**	Daily	Cumul.
			20	70		20	60			40
**Survey Team**	**N°**	**GPS / Tides (Hours):**	Daily	Cumul.	**Planning (Hours):**	Daily	Cumul.	**Other (Hours):**	Daily	Cumul.
	7		25	287		5	65			130

Equipment / Logistics (to include aircraft and airborne system defects / changes)
Remarks :

Laser SRL No: 2001 **Green RX SRL No:** 2002

Narrative: (Customer/Personnel related issues & general comments)

Flight was aborted due to turbulence caused by strong easterly winds

Disappointing secchi results in Lough Foyle.

Olivia Wilson viewed office processes.

24hr Forecast: No Planned flight due to forecast strong easterly winds

No flight approval received for Northern Ireland, no flight to Lough Foyle.

DAILY SURVEY LOG

Survey Title : Ireland 2008 **Database :** 08_07NW Ireland

Date : Monday 26 May 2008 **Julian Day :** 147

Weather in Survey Area

Cloud : 4 *octas* *feet* Sea State : 3

Wind : NE *degrees* 25 -30 *speed* Visibility : good

Swell : nil *degrees* Nil *metres*

Weather Comments: Strong NE winds causing turbulence developing.

General Comments: No flight planned due to forecast winds.

Data Collection Cons flight: Sortie Number : AS GS

Time On Task : Downtime Env Downtime Sys

Flight Time: Aircraft (Weather Holding / Aircraft AOG):

Crew: Pilot Duty Hours:

Navcals : Pilot Flying Hours:

Benchmarks :

Survey Lines :

Remarks : No Planned flight - Weather

Progress:

Environmental Relies Flown:	Cloud:	Daily	Cumul.	Swell:	Daily	Cumul.	Coverage:	Daily	Cumul.
		0	0		0	0		0	0
System Reflies Flown:	KF - Tilt:	Daily	Cumul.	Sea Surface:	Daily	Cumul.	Other Cause:	Daily	Cumul.
		0	0		0	0		0	2

Survey Issues

Data Processing:

Preval - sortie 5

Process Tides Data

Survey Team Stats:		Flying Ops (hours):	Daily	Cumul.	Pre Val (Hours):	Daily	Cumul.	Validation (Hours):	Daily	Cumul.
			0	70		40	110		0	0
Survey Team	Nº	GPS / Tides (Hours):	Daily	Cumul.	Planning (Hours):	Daily	Cumul.	Other (Hours):	Daily	Cumul.
	7		20	307		10	75			110

Equipment / Logistics (to include aircraft and airborne system defects / changes)

Remarks : Nil issues

Laser SRL No:	2001	Green RX SRL No:	2002

Narrative: (Customer/Personnel related issues & general comments)

Strong NE winds forecast, cause turbulence over hills, conditions unsuitable for operations in all areas. No Flight Approval for Northern Ireland work.
Tim sollart arrived

24hr Forecast: Day time flight to Donegal Bay.

DAILY SURVEY LOG

Survey Title : Ireland 2008 **Database :** 08_07NW Ireland

Date : Tuesday 27 May 2008 **Julian Day :** 148

Weather in Survey Area

Cloud :	4 octas	3000	feet	Sea State :	3
Wind :	E degrees	30	speed	Visibility :	Moderate
Swell :	nil degrees	Nil	metres		

Weather Comments: Slight drop in the strong NE winds, rain forecast for the afternoon.

General Comments: Planned flight to Donegal Bay / Galway Bay

Data Collection Cons flight: 4 Sortie Number : AS 1195 GS 7

Time On Task : 4:47 Downtime WX Nil Downtime Sys Nil

Flight Time: 5:56 Aircraft (Weather Holding / Aircraft AOG): Nil

Crew: TS, DH, BCM, TPH, RJW Pilot Duty Hours: 7:45

Navcals : 1 Pilot Flying Hours: 5:56

Benchmarks : 2

Survey Lines : Galway - 32 x mainline

Remarks / Defects :

Progress: Galway Bay - 59% flown

Environmental Reflies Flown:	Cloud:	Daily	Cumul.	Swell:	Daily	Cumul.	Coverage:	Daily	Cumul.
		0	0		0	0		0	0
System Reflies Flown:	KF - Tilt:	Daily	Cumul.	Sea Surface:	Daily	Cumul.	Other Cause:	Daily	Cumul.
		0	0		0	0		0	2

Survey Issues

Data Processing:

Preval of Sortie 6 completed.

Rosaveel Tide gauge downloaded.

Survey Team Stats:		Flying Ops (hours):	Daily	Cumul.	Pre Val (Hours):	Daily	Cumul.	Validation (Hours):	Daily	Cumul.
			30	100		20	130		0	0
Survey Team	Nº	GPS / Tides (Hours):	Daily	Cumul.	Planning (Hours):	Daily	Cumul.	Other (Hours):	Daily	Cumul.
	7		10	317		10	85	Travel / DO		110

Equipment / Logistics (to include aircraft and airborne system defects / changes)

Remarks : Nil issues, still awaiting Gandalf

Laser SRL No: 2001 **Green RX SRL No:** 2002

Narrative: (Customer/Personnel related issues & general comments)

Conditions still marginal with strong E winds causing turbulence in all areas, Flight achieved to Galway Bay, conditions in Donegal Bay not suitable for Operations. Need to consider activating WX alternative - Tralee Bay.

24hr Forecast: Day time sortie, weather permitting.

DAILY SURVEY LOG

Survey Title : Ireland 2008 **Database :** 08_07NW Ireland

Date : Wednesday 28 May 2008 **Julian Day :** 149

Weather in Survey Area

Cloud :	Sct / Bkn	octas	1200 to 2500	feet	Sea State :	0
Wind :	W	degrees	4	speed	Visibility :	Moderate
Swell :	nil	degrees	Nil	metres		

Weather Comments: Winds dropped overnight, low cloud and mist during morning, lifted during afternoon.

General Comments: Planned flight cancelled due to weather conditions

Data Collection Cons flight: Sortie Number : AS GS

Time On Task : Downtime WX Downtime Sys

Flight Time: Aircraft (Weather Holding / Aircraft AOG):

Crew: Pilot Duty Hours:

Navcals : Pilot Flying Hours:

Benchmarks :

Survey Lines :

Flight cancelled due to weather

Remarks / Defect

Progress:

Environmental Reflies Flown:	Cloud:	Daily	Cumul.	Swell:	Daily	Cumul.	Coverage:	Daily	Cumul.
		0	0		0	0		0	0
**System Reflies Flown:**	**KF - Tilt:**	Daily	Cumul.	**Sea Surface:**	Daily	Cumul.	**Other Cause:**	Daily	Cumul.
		0	0		0	0	Platform	0	2

Survey Issues

Data Processing:

Preval Sortie 7

Survey Team Stats:		Flying Ops (hours):	Daily	Cumul.	Pre Val (Hours):	Daily	Cumul.	Validation (Hours):	Daily	Cumul.
			0	100		20	150		0	0
Survey Team	Nº	GPS / Tides (Hours):	Daily	Cumul.	Planning (Hours):	Daily	Cumul.	Other (Hours):	Daily	Cumul.
	7		25	342		5	90		20	130

Equipment / Logistics (to include aircraft and airborne system defects / changes)

Remarks : Still waiting for Frodo to arrive in Ireland.
Digital Camera identified as U/S on previous sortie, replaced with spare.

Laser SRL No: 2001 **Green RX SRL No:** 2002

Narrative: (Customer/Personnel related issues & general comments)

Very poor conditions during morning, flight cancelled.
Conditions improved during afternoon, forecast good for tomorrow.
Shifted Tide Gauge from Mullagmore to Tralee Bay

24hr Forecast: Planned Flight to Donegal / Tralee Bay

DAILY SURVEY LOG

Survey Title : Ireland 2008 **Database :** 08_07NW Ireland

Date : Thursday 29 May 2008 **Julian Day :** 150

Weather in Survey Area

Cloud :	3	*octas*	2400	*feet*	Sea State :	0/1
Wind :	SE	*degrees*	10	*speed*	Visibility :	Good
Swell :	NE	*degrees*	0.5	*metres*	Rain:	yes

Weather Comments: Passing showers, calm patches during the day

General Comments: Long day planned with two sorties to Tralee Bay with a fuelling stop in-between.

Data Collection Cons flight: 5 Sortie Number : AS 1195 GS 8,9

Time On Task : 5:58 Downtime WX Nil Downtime Sys Nil

Flight Time: 8:31 Aircraft (Weather Holding / Aircraft AOG): Nil

Crew: PJ, DH, NAT, BCM, AMW Pilot Duty Hours: 11:00

Navcals : 1 Pilot Flying Hours: 8:31

Benchmarks :

Survey Lines : Tralee - 29 x Mainlines
Galway - 5 x Mainlines

Remarks / Defects : Refueled at Kerry Airport

Progress: Galway Bay - 59% flown
Tralee Commenced

Environmental Reflies Flown:	Cloud:	Daily	Cumul.	Swell:	Daily	Cumul.	Coverage:	Daily	Cumul.
		0	0		0	0		0	0
System Reflies Flown:	KF - Tilt:	Daily	Cumul.	Sea Surface:	Daily	Cumul.	Other Cause:	Daily	Cumul.
		0	0		0	0		0	2

Survey Issues

Data Processing:

QC flights 1 - 4

Install tide gauge in Tralee Bay.

Survey Team Stats:		Flying Ops (hours):	Daily	Cumul.	Pre Val (Hours):	Daily	Cumul.	Validation (Hours):	Daily	Cumul.
			30	130		20	170			0
Survey Team	N°	GPS / Tides (Hours):	Daily	Cumul.	Planning (Hours):	Daily	Cumul.	Other (Hours):	Daily	Cumul.
	7		20	362			90			130

Equipment / Logistics (to include aircraft and airborne system defects / changes)

Remarks : Still waiting for Frodo

Laser SRL No: 2001 **Green RX SRL No:** 2002

Narrative: (Customer/Personnel related issues & general comments)

A good flight to Tralee, large area collected in entrance to Bay. Stopped to refuel at Kerry airport, then conducted a second short sortie.
Tide gauge installed in Tralee Bay.

24hr Forecast: Planned flight to Tralee / Galway Areas

Survey Title : Ireland 2008 **Database :** 08_9nw_ireland

Date : Friday 30 May 2008 **Julian Day :** 151

Weather in Survey Area

Cloud :	3	*octas*	2000	*feet*	Sea State :	2
Wind :	S	*degrees*	4	*speed*	Visibility :	Var
Swell :	NW	*degrees*	1.0	*metres*	Rain:	Y

Weather Comments:

General Comments: Flight cancelled due to weather

Data Collection Cons flight: Sortie Number : AS GS

Time On Task : Downtime WX Downtime Sys

Flight Time: Aircraft (Weather Holding / Aircraft AOG):

Crew: Pilot Duty Hours:

Navcals :

Benchmarks :

Survey Lines :

Flight cancelled due to weather

Remarks / Defects :

Progress:

		Daily	Cumul.		Daily	Cumul.		Daily	Cumul.
**Environmental Reflies Flown:**	**Cloud:**	0	0	**Swell:**	0	0	**Coverage:**	0	0
**System Reflies Flown:**	**KF - Tilt:**	0	0	**Sea Surface:**	0	0	**Other Cause:** Platform	0	2

Survey Issues

Data Processing:

Preval Sortie 8 and 9

**Survey Team Stats:**			Daily	Cumul.		Daily	Cumul.		Daily	Cumul.
		**Flying Ops (hours):**	0	130	**Pre Val (Hours):**	30	200	**Validation (Hours):**	0	0
**Survey Team**	**N°** 7	**GPS/Tides (Hours):**	30	392	**[illegible]nning (Hours):**	10	100	**Other (Hours):** Day Off	10	140

Equipment / Logistics (to include aircraft and airborne system defects / changes)

Remarks : Nil issues

**Laser SRL No:**	2001	**Green RX SRL No:**	2002

Narrative: (Customer/Personnel related issues & general comments)

General Day Off

24hr Forecast: Planned Flight to Tralee / Galway Areas

DAILY SURVEY LOG

Survey Title : Ireland 2008 **Database :** 08_07NW Ireland

Date : Saturday 31 May 2008 **Julian Day :** 152

Weather in Survey Area

Cloud :	3-4	octas	5000	feet	Sea State :	3
Wind :	SW	degrees	<10	speed	Visibility :	Varies
Swell :	W	degrees	1.5	metres	Rain:	Nil

Weather Comments: Fog off Tralee, Storms along west coast, calm conditions with slight sea breeze

General Comments: Afternoon flight attempted to all areas

Data Collection Cons flight: 6 Sortie Number : AS 1197 GS 10,11

Time On Task : 4:49 Downtime WX Nil Downtime Sys Nil

Flight Time: 8:03 Aircraft (Weather Holding / Aircraft OG):

Crew: TS, DH, TPH, KJW, RJW Pilot Duty Hours: 10:45

Navcals : 1 Pilot Flying Hours: 8:03

Benchmarks :

Survey Lines : Galway - 22 x mainline, 6 x Addnl coverage Lines

Remarks / Defects : Refueled at Galway to extend flight

Progress: Galway Bay - 76% flown
Tralee Bay - 29% flown

Environmental Reflies Flown:	Cloud:	Daily	Cumul.	Swell:	Daily	Cumul.	Coverage:	Daily	Cumul.
		0	0		0	0		5	5
**System Reflies Flown:**	**KF - Tilt:**	Daily	Cumul.	**Sea Surface:**	Daily	Cumul.	**Other Cause:**	Daily	Cumul.
		0	0		0	0		0	2

Survey Issues

Data Processing:

Preval Sortie 8,9 completed
Coverage plots generated

Survey Team Stats:		Flying Ops (hours):	Daily	Cumul.	Pre Val (Hours):	Daily	Cumul.	Validation (Hours):	Daily	Cumul.
			30	160		20	220			0
Survey Team	Nº	GPS / Tides (Hours):	Daily	Cumul.	Planning (Hours):	Daily	Cumul.	Other (Hours):	Daily	Cumul.
	7		10	402		10	110	Day off	0	140

Equipment / Logistics (to include aircraft and airborne system defects / changes)

Remarks : Nil issues

**Laser SRL No:**	2001	**Green RX SRL No:**	2002

Narrative: (Customer/Personnel related issues & general comments)

Along flight to all three areas, fog in Tralee, dirty water in Donegal Bay, stopped to refuel in Galway prior to a full sortie in Galway Bay conducting 200% coverage along the north coast of Galway Bay.

24hr Forecast: Afternoon flight to Donegal / Tralee Bay

DAILY SURVEY LOG

Survey Title : Ireland 2008 **Database :** 08_07NW Ireland

Date : Sunday 01 June 2008 **Julian Day :** 153

Weather in Survey Area

Cloud :	4	*octas*	4000	*feet*	Sea State :	2
Wind :	NE	*degrees*	11	*speed*	Visibility :	Fog
Swell :		*degrees*	0.5	*meters*	Rain:	Nil

Weather Comments: Heavy Fog off coastline

General Comments: Planned afternoon flight Galway Bay to try and complete inshore 200% areas.

Data Collection Cons flight: 7 Sortie Number : AS 1198 GS 12

Time On Task : 1:23 Downtime WX 4:28 Downtime Sys

Flight Time: 2:32 Aircraft (Weather Holding / Aircraft AOG):

Crew: TS, DH, TPH, KJW Pilot Duty Hours: 4:00

Navcals : Pilot Flying Hours: 2:32

Benchmarks : 2

Survey Lines : Galway Bay - 5 Mainlines, 5 x Cross Ties

Remarks / Defects : Flight aborted due to Fog in Tralee and Galway Bay, dirty water in Donegal

Progress: Galway Bay - 76% flown
Tralee Bay - 29% flown

**Environmental Reflies Flown:**	**Cloud:**	Daily	Cumul.	**Swell:**	Daily	Cumul.	**Coverage:**	Daily	Cumul.
		0	0		0	0		0	5
**System Reflies Flown:**	**KF - Tilt:**	Daily	Cumul.	**Sea Surface:**	Daily	Cumul.	**Other Cause:**	Daily	Cumul.
		0	0		0	0		0	2

Survey Issues

Data Processing:

Preval of Sortie 5

Conducted Boat Recce of Lough Foyle, strong easterly winds, reduced water clarity from previous recces.

**Survey Team Stats:**		**Flying Ops (hours):**	Daily	Cumul.	**Pre Val (Hours):**	Daily	Cumul.	**Validation (Hours):**	Daily	Cumul.
			20	180		20	240			0
**Survey Team**	**N°**	**GPS / Tides (Hours):**	Daily	Cumul.	**Planning (Hours):**	Daily	Cumul.	**Other (Hours):**	Daily	Cumul.
	7		10	412			110		20	160

Equipment / Logistics (to include aircraft and airborne system defects / changes)

Remarks : Nil issues.

Laser SRL No: 2001 **Green RX SRL No:** 2002

Narrative: (Customer/Personnel related issues & general comments)

With low cloud / Fog forecast for Tralee, and Dirty water in Donegal Bay, a sortie was flown to finish off Galway Bay. Persistent Fog offshore spread into the bay despite the 25°C temperatures. Sortie Aborted after 1 hour on task.

24hr Forecast: Planned afternoon flight

DAILY SURVEY LOG

Survey Title : Ireland 2008 **Database :** 08_07NW Ireland

Date : Monday 02 June 2008 **Julian Day :** 154

Weather in Survey Area

Cloud :	1	octas	4000	feet	Sea State :	3
Wind :	SSW	degrees	10 - 30	speed	Visibility :	good
Swell :	nil	degrees	Nil	metres		

Weather Comments: Initially good conditions, storm front moved in from SW towards end of sortie, operations moved to Galway.

General Comments: Planned double sortie to Tralee Bay

Data Collection Cons flight: 8 Sortie Number : AS 1199 GS 13

Time On Task : 4:19 Downtime Env Nil Downtime Sys Nil

Flight Time: 5:42 Aircraft (Weather Holding / Aircraft AOG): nil

Crew: DH, TS, NAT, TPH, KJW Pilot Duty Hours: 7:18

Navcals : 1 Pilot Flying Hours: 5:42

Benchmarks :

Survey Lines : Galway - 2 x refly line (GPS)
Tralee - 30 x mainlines, 1 x refly line (cloud)

Remarks / Defects : Frontal system closed in from SW bring low cloud forcing operations in Tralee to be aborted, sortie finished at Galway

Progress: Galway Bay - 82% flown
Tralee Bay - 46% flown

**Environmental Relies Flown:**	**Cloud:**	Daily 0	Cumul. 0	**Swell:**	Daily 0	Cumul. 0	**Coverage:**	Daily 2	Cumul. 7
**System Reflies Flown:**	**KF - Tilt:**	Daily 0	Cumul. 0	**Sea Surface:**	Daily 0	Cumul. 0	**Other Cause:** GPS	Daily 2	Cumul. 4

Survey Issues

Data Processing:

Preval - sortie 12

Process Tides Data

**Survey Team Stats:**		**Flying Ops (hours):**	Daily 30	Cumul. 210	**Pre Val (Hours):**	Daily 20	Cumul. 260	**Validation (Hours):** Daily 0	Cumul. 0
**Survey Team**	**N° 7**	**GPS / Tides (Hours):**	Daily 10	Cumul. 412	**Planning (Hours):**	Daily 10	Cumul. 120	**Other (Hours):** Daily	Cumul. 160

Equipment / Logistics (to include aircraft and airborne system defects / changes)

Remarks : Nil issues

Laser SRL No: 2001 **Green RX SRL No:** 2002

Narrative: (Customer/Personnel related issues & general comments)

Good conditions in Tralee Bay, 4 hours on task achieved prior to frontal system bringing strong winds and low cloud. Sortie finished off in Galway Bay. Conditions in Tralee Bay expected to remain unsuitable for 2-3 days.

24hr Forecast: Day time flight to Donegal Bay.

DAILY SURVEY LOG

Survey Title : Ireland 2008 **Database :** 08_07NW Ireland

Date : Tuesday 03 June 2008 **Julian Day :** 155

Weather in Survey Area

Cloud :	3	octas	2500	feet	Sea State :	3
Wind :	WSW	degrees	30	speed	Visibility :	Moderate
Swell :	SW	degrees	1-2	metres		

Weather Comments: Strengthen wind from the SW, building swell

General Comments: Planned flight to Donegal Bay / Galway Bay

Data Collection Cons flight: 9 Sortie Number : AS 1200 GS 14

Time On Task : 1:04 Downtime WX 4:17 Downtime Sys Nil

Flight Time: 2:43 Aircraft (Weather Holding / Aircraft AOG): Nil

Crew: TS, DH, KJW, TPH, NAT Pilot Duty Hours: 4:00

Navcals : 1 Pilot Flying Hours: 2:43

Benchmarks : 2

Survey Lines : Donegal - 2 x mainline
Galway - 3 x Addnl Coverage lines

Remarks / Defects : Flight aborted due to water and weather conditions.

Progress: Galway Bay - 59% flown

Environmental Reflies Flown:	Cloud:	Daily	Cumul.	Swell:	Daily	Cumul.	Coverage:	Daily	Cumul.
		0	0		0	0		3	10
System Reflies Flown:	KF - Tilt:	Daily	Cumul.	Sea Surface:	Daily	Cumul.	Other Cause:	Daily	Cumul.
		0	0		0	0		0	4

Survey Issues

Data Processing:

Preval of Sortie 13 completed.

Survey Team Stats:		Flying Ops (hours):	Daily	Cumul.	Pre Val (Hours):	Daily	Cumul.	Validation (Hours):	Daily	Cumul.
			30	240		40	300		0	0
Survey Team	Nº	GPS / Tides (Hours):	Daily	Cumul.	Planning (Hours):	Daily	Cumul.	Other (Hours):	Daily	Cumul.
	7		0	412		0	120			160

Equipment / Logistics (to include aircraft and airborne system defects / changes)

Remarks : Extension of accommodation and office arranged

Laser SRL No: 2001 **Green RX SRL No:** 2002

Narrative: (Customer/Personnel related issues & general comments)

Flight attempted to Donegal, aborted due to poor water conditions, diverted to Galway, aborted due to high swell, wind and water conditions.

24hr Forecast: No planned flight, forecast 3.5m swell, strong winds

DAILY SURVEY LOG

Survey Title : Ireland 2008 **Database :** 08_07NW Ireland

Date : Wednesday 04 June 2008 **Julian Day :** 156

Weather in Survey Area

Cloud : 8 octas 2500 feet Sea State : 0
Wind : SW degrees 10 - 30 speed Visibility : Moderate
Swell : W degrees 3.0 metres rain

Weather Comments: Strong winds from the W, high swell building, rain

General Comments: No planned flight due to forecast bad weather

Data Collection Cons flight: Sortie Number : AS GS
Time On Task : Downtime WX Downtime Sys
Flight Time: Aircraft (Weather Holding / Aircraft AOG):
Crew: Pilot Duty Hours:
Navcals : Pilot Flying Hours:
Benchmarks :
Survey Lines :

No planned Flight due to Weather

Remarks / Defect
Progress:

Environmental Reflies Flown:	Cloud:	Daily	Cumul.	Swell:	Daily	Cumul.	Coverage:	Daily	Cumul.
		0	0		0	0		0	10
System Reflies Flown:	KF - Tilt:	Daily	Cumul.	Sea Surface:	Daily	Cumul.	Other Cause:	Daily	Cumul.
		0	0		0	0		0	4

Survey Issues
Data Processing:
Preval Sortie 14
Download Rossaveel and Tralee Tide Gauges

Survey Team Stats:		Flying Ops (hours):	Daily	Cumul.	Pre Val (Hours):	Daily	Cumul.	Validation (Hours):	Daily	Cumul.
			0	240		40	340		0	0
Survey Team	Nº	GPS / Tides (Hours):	Daily	Cumul.	Planning (Hours):	Daily	Cumul.	Other (Hours):	Daily	Cumul.
	7		25	437		5	125			160

Equipment / Logistics (to include aircraft and airborne system defects / changes)
Remarks : Office relocated to another meeting room within Hotel

Laser SRL No: 2001 **Green RX SRL No:** 2002

Narrative: (Customer/Personnel related issues & general comments)
Forecast bad weather arrived as predicted, no flight.
Field office moved due to hotel requirements.

24hr Forecast: Standby flying day, weather dependant.

DAILY SURVEY LOG

Survey Title : Ireland 2008 **Database :** 08_07NW Ireland

Date : Thursday 05 June 2008 **Julian Day :** 157

Weather in Survey Area

Cloud :	3	octas	2000	feet	Sea State :	2/3
Wind :	NW	degrees	10	speed	Visibility :	Good
Swell :	NW	degrees	0.5	metres	Rain:	

Weather Comments: Passing showers, with sunny patches

General Comments: Final sortie to complete Galway 200% and commence Oceanographic Buoy Area

Data Collection Cons flight: 10 Sortie Number : AS 1201 GS 15

Time On Task : 4:58 Downtime WX Nil Downtime Sys 0:27

Flight Time: 5:57 Aircraft (Weather Holding / Aircraft AOG): Nil

Crew: TS, DH, BCM, RJW Pilot Duty Hours: 7:30

Navcals : 1 Pilot Flying Hours: 5:57

Benchmarks : 2

Survey Lines : Galway - 19 x 200% Mainlines, 6 x Refly Lines (coverage)
3 x Refly (GPS)
Oceanographic Buoy - 5 x Mainlines

Remarks / Defects : WAGPS dropped out at times, laser failed to start, reboot required.

Progress: Galway Bay - 100% flown
Tralee - 48% flown

**Environmental Reflies Flown:**	**Cloud:**	Daily	Cumul.	**Swell:**	Daily	Cumul.	**Coverage:**	Daily	Cumul.
		0	0		0	0		6	16
**System Reflies Flown:**	**KF - Tilt:**	Daily	Cumul.	**Sea Surface:**	Daily	Cumul.	**Other Cause:**	Daily	Cumul.
		0	0		0	0	GPS	3	7

Survey Issues

Data Processing:

Process Tide Data

**Survey Team Stats:**		**Flying Ops (hours):**	Daily	Cumul.	**Pre Val (Hours):**	Daily	Cumul.	**Validation (Hours):**	Daily	Cumul.
			20	260			340			0
**Survey Team**	**N⁰**	**GPS / Tides (Hours):**	Daily	Cumul.	**Planning (Hours):**	Daily	Cumul.	**Other (Hours):**	Daily	Cumul.
	7		20	457		10	135	Day Off	30	190

Equipment / Logistics (to include aircraft and airborne system defects / changes)

Remarks : WAGOPS dropped out during sortie, Laser failed to fire up
Laser failed to start at start of sortie, reboot and second MBA required.

Laser SRL No: 2001 **Green RX SRL No:** 2002

Narrative: (Customer/Personnel related issues & general comments)

Sortie to Galway Bay to improve coverage with by extending 200% coverage, com plete refly lines and obtain coveage in the Oceanographic Buoy area.

24hr Forecast: Aftgernoon flight to Complete Tralee Bay

DAILY SURVEY LOG

Survey Title : Ireland 2008 **Database :** 08_07NW Ireland

Date : Friday 06 June 2008 **Julian Day :** 158

Weather in Survey Area

Cloud :	3	*octas*	2500	*feet*	Sea State :	1
Wind :	W	*degrees*	15	*speed*	Visibility :	Good
Swell :	NW	*degrees*	1-2	*metres*	Rain:	Nil

Weather Comments: Good conditions, swell slowly abating

General Comments: Planned double flight to Tralee Bay to finish off the contracted survey of Tralee Bay.

Data Collection Cons flight: 11 Sortie Number : AS 1202 GS 16,17

Time On Task : 7:13 Downtime Envir Downtime Sys

Flight Time: 8:58 Aircraft (Weather Holding / Aircraft AOG):

Crew: TS, DH, NAT, KJW, TPH Pilot Duty Hours: 11:00

Navcals : Nil Pilot Flying Hours: 8:58

Benchmarks : Nil

Survey Lines : Tralee - 36 x Mainlines, 6 x Addnl Coverage Lines
Tralee - 4 x Refly Lines (tilt), 2 x Refly (Coverage)

Remarks / Defects : Nil Issues

Progress: Galway Bay - 100% flown
Tralee Bay - 100% flown

**Environmental Reflies Flown:**	**Cloud:**	Daily	Cumul.	**Swell:**	Daily	Cumul.	**Coverage:**	Daily	Cumul.
		0	0		0	0		8	24
**System Reflies Flown:**	**KF - Tilt:**	Daily	Cumul.	**Sea Surface:**	Daily	Cumul.	**Other Cause:**	Daily	Cumul.
		4	4		0	0		0	7

Survey Issues

Data Processing:

Preval of Sortie 14, 15
Download Rosaveel Tide Gauge

**Survey Team Stats:**		**Flying Ops (hours):**	Daily	Cumul.	**Pre Val (Hours):**	Daily	Cumul.	**Validation (Hours):**	Daily	Cumul.
			30	290		30	370			0
**Survey Team**	**N°**	**GPS / Tides (Hours):**	Daily	Cumul.	**Planning (Hours):**	Daily	Cumul.	**Other (Hours):**	Daily	Cumul.
	7		10	467			135		0	190

Equipment / Logistics (to include aircraft and airborne system defects / changes)

Remarks : Nil issues

Laser SRL No: 2001 **Green RX SRL No:** 2002

Narrative: (Customer/Personnel related issues & general comments)

A successful double sortie to Tralee Bay to finish off this area.

24hr Forecast: No planned flight, flying in support of initial contract completed.

DAILY SURVEY LOG

Survey Title : Ireland 2008 **Database :** 08_07NW Ireland

Date : Saturday 07 June 2008 **Julian Day :** 159

Weather in Survey Area

Cloud :	3-4	*octas*	3000	*feet*	Sea State :	3
Wind :	SW	*degrees*	<10	*speed*	Visibility :	Varies
Swell :	W	*degrees*	1.5	*metres*	Rain:	

Weather Comments: overcast, passing showeres during afternoon

General Comments: Planned Non flying day, review survey data.

Data Collection Cons flight: Sortie Number : AS GS

Time On Task : Downtime WX Downtime Sys Nil

Flight Time: Aircraft (Weather Holding / Aircraft OG):

Crew: Pilot Duty Hours:

Navcals : Pilot Flying Hours:

Benchmarks :

Survey Lines :

Remarks / Defect

No Planned flight

Progress: Galway Bay - 100% flown
Tralee Bay - 100% flown

Environmental Reflies Flown:	Cloud:	Daily	Cumul.	Swell:	Daily	Cumul.	Coverage:	Daily	Cumul.
		0	0		0	0		0	24
System Reflies Flown:	KF - Tilt:	Daily	Cumul.	Sea Surface:	Daily	Cumul.	Other Cause:	Daily	Cumul.
		0	4		0	0		0	7

Survey Issues
Data Processing:
Preval Sortie 16

Survey Team Stats:		Flying Ops (hours):	Daily	Cumul.	Pre Val (Hours):	Daily	Cumul.	Validation (Hours):	Daily	Cumul.
			0	290		50	420			0
Survey Team	N°	GPS / Tides (Hours):	Daily	Cumul.	Planning (Hours):	Daily	Cumul.	Other (Hours):	Daily	Cumul.
	7		10	477		0	135	Day off	10	200

Equipment / Logistics (to include aircraft and airborne system defects / changes)
Remarks : Nil issues

Laser SRL No: 2001 **Green RX SRL No:** 2002

Narrative: (Customer/Personnel related issues & general comments)
Planned non flying day, flying operations in support of initial work contract work completed

24hr Forecast: Planned Non flying day, review survey coverage

DAILY SURVEY LOG

Survey Title : Ireland 2008 **Database :** 08_07NW Ireland

Date : Sunday 08 June 2008 **Julian Day :** 160

Weather in Survey Area

Cloud :	8	octas	3000	feet	Sea State :	2
Wind :	NE	degrees	11	speed	Visibility :	good
Swell :		degrees	0.5	meters	Rain:	

Weather Comments: Passing Showers

General Comments: Planned non flying day, reviewing coverage in completed contract areas.

Data Collection Cons flight: Sortie Number : AS GS

Time On Task : Downtime WX Downtime Sys

Flight Time: Aircraft (Weather Holding / Aircraft AOG):

Crew: Pilot Duty Hours:

Navcals : Pilot Flying Hours:

Benchmarks :

Survey Lines :

Remarks / Defect

No Planned flight

Progress: Galway Bay - 100% flown
Tralee Bay - 100% flown

**Environmental Reflies Flown:**	**Cloud:**	Daily	Cumul.	**Swell:**	Daily	Cumul.	**Coverage:**	Daily	Cumul.
		0	0		0	0		0	24
**System Reflies Flown:**	**KF - Tilt:**	Daily	Cumul.	**Sea Surface:**	Daily	Cumul.	**Other Cause:**	Daily	Cumul.
		0	4		0	0		0	7

Survey Issues

Data Processing:

Preval Sortie 17
Download Tralee Tide Gauge (8 hour round trip)

**Survey Team Stats:**		**Flying Ops (hours):**	Daily	Cumul.	**Pre Val (Hours):**	Daily	Cumul.	**Validation (Hours):**	Daily	Cumul.
			0	290		20	440			0
**Survey Team**	**N°**	**GPS / Tides (Hours):**	Daily	Cumul.	**Planning (Hours):**	Daily	Cumul.	**Other (Hours):**	Daily	Cumul.
	7		20	497		10	145		20	220

Equipment / Logistics (to include aircraft and airborne system defects / changes)

Remarks : Nil issues.

Laser SRL No: 2001 **Green RX SRL No:** 2002

Narrative: (Customer/Personnel related issues & general comments)

Quite day reviewing survey data.

24hr Forecast: Standby flying day,

DAILY SURVEY LOG

Survey Title : Ireland 2008 **Database :** 08_07NW Ireland

Date : Monday 09 June 2008 **Julian Day :** 161

Weather in Survey Area

Cloud :	8	octas	1500	feet	Sea State :	3
Wind :	SSW	degrees	10 -30	speed	Visibility :	good
Swell :	nil	degrees	Nil	metres		

Weather Comments: Strong winds from the SW, high swell building offshore

General Comments: No Planned flight, poor weather conditions in Lough Foyle, awaiting secchi results from boat recce in Lough Foyle

Data Collection Cons flight: Sortie Number : AS GS

Time On Task : Downtime Env Nil Downtime Sys Nil

Flight Time: Aircraft (Weather Holding / Aircraft AOG): nil

Crew: Pilot Duty Hours:

Navcals : Pilot Flying Hours:

Benchmarks :

Survey Lines :

Remarks / Defect

No Planned Flight due to Weather

Progress: Galway Bay - 100% flown
Tralee Bay - 100% flown

Environmental Relies Flown:	Cloud:	Daily	Cumul.	Swell:	Daily	Cumul.	Coverage:	Daily	Cumul.
		0	0		0	0			24
System Reflies Flown:	KF - Tilt:	Daily	Cumul.	Sea Surface:	Daily	Cumul.	Other Cause:	Daily	Cumul.
		0	4		0	0			7

Survey Issues

Data Processing:

Commenced Validation

Relocate processing office within hotel

Survey Team Stats:		Flying Ops (hours):	Daily	Cumul.	Pre Val (Hours):	Daily	Cumul.	Validation (Hours):	Daily	Cumul.
			30	320		20	460		0	0
Survey Team	Nº	GPS / Tides (Hours):	Daily	Cumul.	Planning (Hours):	Daily	Cumul.	Other (Hours):	Daily	Cumul.
	7		10	507		10	145			220

Equipment / Logistics (to include aircraft and airborne system defects / changes)

Remarks : Office relocated due to hotel requirements

Planning for move to Alaska

Laser SRL No: 2001 **Green RX SRL No:** 2002

Narrative: (Customer/Personnel related issues & general comments)

No flight to Lough Foyle due to strong SW winds and tides, waiting for secchi results from Loughs Agency boat. Processing office was relocated due to hotel requirements.

24hr Forecast: Survey Flight to Lough Foyle

DAILY SURVEY LOG

Survey Title : Ireland 2008 **Database :** 08_07NW Ireland

Date : Tuesday 10 June 2008 **Julian Day :** 162

Weather in Survey Area

Cloud :	3	octas	3000	feet	Sea State :	3
Wind :	WSW	degrees	20	speed	Visibility :	Moderate
Swell :	SW	degrees	1-2	metres		

Weather Comments: Strong wind from the W, building swell

General Comments: Planned flight to Lough Foyle cancelled due to weather and poor secchi readings.

Data Collection Cons flight: Sortie Number : AS GS

Time On Task : Downtime WX Downtime Sys

Flight Time: Aircraft (Weather Holding / Aircraft AOG):

Crew: Pilot Duty Hours: 2:00

Navcals : Pilot Flying Hours: 0:00

Benchmarks :

Survey Lines :

Remarks / Defects : Flight cancelled just prior to takeoff

Progress:

Environmental Reflies Flown:	Cloud:	Daily	Cumul.	Swell:	Daily	Cumul.	Coverage:	Daily	Cumul.
		0	0		0	0		0	24
System Reflies Flown:	KF - Tilt:	Daily	Cumul.	Sea Surface:	Daily	Cumul.	Other Cause:	Daily	Cumul.
		0	4		0	0		0	7

Survey Issues

Data Processing:

Validation, processing of tides.

Planning Blacksod Bay work

Survey Team Stats:		Flying Ops (hours):	Daily	Cumul.	Pre Val (Hours):	Daily	Cumul.	Validation (Hours):	Daily	Cumul.
			0	320		0	460		40	40
Survey Team	Nº	GPS / Tides (Hours):	Daily	Cumul.	Planning (Hours):	Daily	Cumul.	Other (Hours):	Daily	Cumul.
	7		10	517		20	165			220

Equipment / Logistics (to include aircraft and airborne system defects / changes)

Remarks : Extension of accommodation and office arranged

Laser SRL No: 2001 **Green RX SRL No:** 2002

Narrative: (Customer/Personnel related issues & general comments)

Flight cancelled at last minute when secchi readings for yesterday were received, Secchi readings for today showed only slight improvement, so tomorrows flight also cancelled.

24hr Forecast: No flight - Planned flight to Lough Foyle cancelled due to poor Secchi readings and strong winds.

DAILY SURVEY LOG

Survey Title : Ireland 2008 **Database :** 08_07NW Ireland

Date : Wednesday 11 June 2008 **Julian Day :** 163

Weather in Survey Area

Cloud : 8 *octas* 1000 *feet* Sea State : 0

Wind : NW *degrees* 10 *speed* Visibility : Moderate

Swell : W *degrees* 3.0 *metres* rain

Weather Comments: Low cloud and rain

General Comments: No planned flight due to forecast bad weather and poor water conditions in Lough Foyle

Data Collection Cons flight: Sortie Number : AS GS

Time On Task : Downtime WX Downtime Sys

Flight Time: Aircraft (Weather Holding / Aircraft AOG):

Crew: Pilot Duty Hours:

Navcals : Pilot Flying Hours:

Benchmarks :

Survey Lines :

No Planned Flight due to Weather

Remarks / Defect Progress:

Environmental Reflies Flown:	Cloud:	Daily	Cumul.	Swell:	Daily	Cumul.	Coverage:	Daily	Cumul.
		0	0		0	0		0	24
System Reflies Flown:	KF - Tilt:	Daily	Cumul.	Sea Surface:	Daily	Cumul.	Other Cause:	Daily	Cumul.
		0	4		0	0		0	7

Survey Issues

Data Processing:

Validation

Download and recover Rossaveel Tide Gauge

Survey Team Stats:		Flying Ops (hours):	Daily	Cumul.	Pre Val (Hours):	Daily	Cumul.	Validation (Hours):	Daily	Cumul.
			0	320		0	460		20	60
Survey Team	N°	GPS / Tides (Hours):	Daily	Cumul.	Planning (Hours):	Daily	Cumul.	Other (Hours):	Daily	Cumul.
	5		20	537		10	175			220

Equipment / Logistics (to include aircraft and airborne system defects / changes)

Remarks : Nil issues

2 surveyors departed for home (Tyson and Adam)

Laser SRL No: 2001 **Green RX SRL No:** 2002

Narrative: (Customer/Personnel related issues & general comments)

Reports from Lough Foyle indicate water conditions are improving. Weather also forecast to improve. Intend flying in Lough Foyle from tomorrow. Koen Verbruggen to visit site and fly tomorrow.

24hr Forecast: Daytime flight to Lough Foyle

DAILY SURVEY LOG

Survey Title : Ireland 2008 **Database :** 08_07NW Ireland

Date : Thursday 12 June 2008 **Julian Day :** 164

Weather in Survey Area

Cloud :	4	*octas*	2000	*feet*	Sea State :	1/2
Wind :	NW	*degrees*	15	*speed*	Visibility :	Good
Swell :	NW	*degrees*	0.5	*metres*	Rain:	

Weather Comments: Passing showers, with sunny patches

General Comments: First sortie to Lough Foyle, planned with a transit to Londonderry to refuel followed a full sortie.

Data Collection Cons flight: 12 Sortie Number : AS 1203 GS 18

Time On Task : 4:54 Downtime WX Nil Downtime Sys Nil

Flight Time: 6:58 Aircraft (Weather Holding / Aircraft AOG): Nil

Crew: TS, DH, BCM, RJW, NAT, Koen Verbruggen Pilot Duty Hours: 10:00

Navcals : Nil Pilot Flying Hours: 6:58

Benchmarks : Nil

Survey Lines : Lough Foyle - 29 x 100% Mainlines, 2 x Refly Lines (Roll Lock)

Remarks / Defects :

Progress: Galway Bay - 100% flown
Tralee - 100% flown
Lough Foyle - 20% flown

**Environmental Reflies Flown:**	**Cloud:**	Daily	Cumul.	**Swell:**	Daily	Cumul.	**Coverage:**	Daily	Cumul.
		0	0		0	0		0	24
**System Reflies Flown:**	**KF - Tilt:**	Daily	Cumul.	**Sea Surface:**	Daily	Cumul.	**Other Cause:**	Daily	Cumul.
		0	4		0	0	Roll Lock	2	9

Survey Issues

Data Processing:

Process Tide Data

Generate Interim Data, Validation

**Survey Team Stats:**		**Flying Ops (hours):**	Daily	Cumul.	**Pre Val (Hours):**	Daily	Cumul.	**Validation (Hours):**	Daily	Cumul.
			30	320		10	450			110
**Survey Team**	**N°**	**GPS / Tides (Hours):**	Daily	Cumul.	**Planning (Hours):**	Daily	Cumul.	**Other (Hours):**	Daily	Cumul.
	5		10	547			175			220

Equipment / Logistics (to include aircraft and airborne system defects / changes)

Remarks : Prepare for Transit to USA

Koen Verbruggen visited site and flew on aircraft.

Laser SRL No: 2001 **Green RX SRL No:** 2002

Narrative: (Customer/Personnel related issues & general comments)

A successful flight to Lough Foyle, with a refueling stop in Londonderry.

Londonderry ATC closed on the aircrafts arrival which delayed the flight by 1 hour, unexpected firing range activation restricted ops to 2000ft.

24hr Forecast: Afternoon flight to Lough Foyle

DAILY SURVEY LOG

Survey Title : Ireland 2008 **Database :** 08_07NW Ireland

Date : Friday 13 June 2008 **Julian Day :** 165

Weather in Survey Area

Cloud :	4	*octas*	3000	*feet*	Sea State :	1/2
Wind :	NW	*degrees*	15	*speed*	Visibility :	Good
Swell :	nil	*degrees*	nil	*metres*	Rain:	nil

Weather Comments: Good conditions, a little turbulence off high ground

General Comments: Planned second sortie to Lough Foyle, if possible refueling stop to Londonderry will be conducted.

Data Collection Cons flight: 13 Sortie Number : AS 1204 GS 20

Time On Task : 7:13 Downtime Envir Downtime Sys

Flight Time: 8:58 Aircraft (Weather Holding / Aircraft AOG):

Crew: TS, DH, BCM, KJW Pilot Duty Hours: 10:30

Navcals : Nil Pilot Flying Hours: 8:31

Benchmarks : Nil

Survey Lines : Lough Foyle - 37 x Mainlines, 1 x Refly Lines (off track)
2 x cross tie lines

Remarks / Defects : DIA failed to save on several lines

Progress: Galway Bay - 100% flown
Tralee Bay - 100% flown
Lough Foyle - 48% flown

**Environmental Reflies Flown:**	**Cloud:**	Daily	Cumul.	**Swell:**	Daily	Cumul.	**Coverage:**	Daily	Cumul.
		0	0		0	0		0	24
**System Reflies Flown:**	**KF - Tilt:**	Daily	Cumul.	**Sea Surface:**	Daily	Cumul.	**Other Cause:**	Daily	Cumul.
		0	4		0	0	off track	1	10

Survey Issues
Data Processing:
Preval of Sortie 18 completed
Recovered Tralee Tide Gauge

**Survey Team Stats:**		**Flying Ops (hours):**	Daily	Cumul.	**Pre Val (Hours):**	Daily	Cumul.	**Validation (Hours):**	Daily	Cumul.
			20	340		10	460			110
**Survey Team**	**Nº**	**GPS / Tides (Hours):**	Daily	Cumul.	**Planning (Hours):**	Daily	Cumul.	**Other (Hours):**	Daily	Cumul.
	5		20	567			175		0	220

Equipment / Logistics (to include aircraft and airborne system defects / changes)
Remarks : Freight for US dispatched, Survey office reduced to 1 terminal

Laser SRL No: 2001 **Green RX SRL No:** 2002

Narrative: (Customer/Personnel related issues & general comments)
A successful double flight to Lough Foyle in good conditions, refueling stop conducted in Londonderry. Tralee tide gauge recovered and will be placed at Mount Charles pier for 25 hour gauge / gauge connection.

24hr Forecast: Planned final flight to Lough Foyle or Blacksod

DAILY SURVEY LOG

Survey Title : Ireland 2008 **Database :** 08_07NW Ireland

Date : Saturday 14 June 2008 **Julian Day :** 166

Weather in Survey Area

Cloud :	3	octas	2000	feet	Sea State :	2
Wind :	N	degrees	<10	speed	Visibility :	Good
Swell :	W	degrees	0.5	metres	Rain:	

Weather Comments: Good Day

General Comments: Planned final flight of deployment to Blacksod Bay

Data Collection Cons flight: 14 Sortie Number : AS 1205 GS 19

Time On Task : 6:14 Downtime WX Nil Downtime Sys Nil

Flight Time: 7:11 Aircraft (Weather Holding / Aircraft OG): Nil

Crew: TS, DH, BCM, KJW Pilot Duty Hours: 9:00

Navcals : Nil Pilot Flying Hours: 7:11

Benchmarks : Nil

Survey Lines : Lough Foyle - 52 x Mainlines, 1 x Cross Lines

Remarks / Defect Nil

Progress: Galway Bay - 100% flown
Tralee Bay - 100% flown
Lough Foyle - 48% flown

**Environmental Reflies Flown:**	**Cloud:**	Daily 0	Cumul. 0	**Swell:**	Daily 0	Cumul. 0	**Coverage:**	Daily 0	Cumul. 24
**System Reflies Flown:**	**KF - Tilt:**	Daily 0	Cumul. 4	**Sea Surface:**	Daily 0	Cumul. 0	**Other Cause:**	Daily 0	Cumul. 10

Survey Issues

Data Processing:

Preval Sortie 20

Install Tide Gauge at Mount Charles for Gauge / Gauge comparison.

Recovered the GPS Base station

**Survey Team Stats:**	**Flying Ops (hours):**	Daily 20	Cumul. 360	**Pre Val (Hours):**	Daily 10	Cumul. 470	**Validation (Hours):**	Daily	Cumul. 110
**Survey Team** N° 5	**GPS / Tides (Hours):**	Daily 20	Cumul. 587	**Planning (Hours):**	Daily 0	Cumul. 175	**Other (Hours):**	Daily 10	Cumul. 230

Equipment / Logistics (to include aircraft and airborne system defects / changes)

Remarks : Processed aircraft freight

Commenced office shut down

Laser SRL No: 2001 **Green RX SRL No:** 2002

Narrative: (Customer/Personnel related issues & general comments)

flight not flown to Lough Foyle after discussion with GSI due to due to short

tidal window of only 3 hours, full flight flown to Blacksod Bay instead.

Excellent sortie in good water conditions, good progress made.

24hr Forecast: Load LCL for transit

DAILY SURVEY LOG

Survey Title : Ireland 2008 **Database :** 08_07NW Ireland

Date : Sunday 15 June 2008 **Julian Day :** 167

Weather in Survey Area

Cloud :	4	*octas*	3000	*feet*	Sea State :	2
Wind :	NE	*degrees*	11	*speed*	Visibility :	good
Swell :		*degrees*	0.5	*meters*	Rain:	

Weather Comments: Nice day, odd passing shower

General Comments: Load LCL, survey team depart, tides team continue to recover tidal equipment.

Data Collection Cons flight: Sortie Number : AS GS

Time On Task : Downtime WX Downtime Sys

Flight Time: Aircraft (Weather Holding / Aircraft AOG):

Crew: Pilot Duty Hours:

Navcals : Pilot Flying Hours:

Benchmarks :

Survey Lines :

Remarks / Defect

No Planned flight

Progress: Galway Bay - 100% flown, 1 full flight flown to Blacksod Bay
Tralee Bay - 100% flown
Lough Foyle - 48% flown

**Environmental Reflies Flown:**	**Cloud:**	Daily	Cumul.	**Swell:**	Daily	Cumul.	**Coverage:**	Daily	Cumul.
		0	0		0	0		0	24
**System Reflies Flown:**	**KF - Tilt:**	Daily	Cumul.	**Sea Surface:**	Daily	Cumul.	**Other Cause:**	Daily	Cumul.
		0	4		0	0		0	10

Survey Issues

Data Processing:

Conduct backups and shut down Processing system
Recover Lough Foyle and Mount Charles Tide Gauges

**Survey Team Stats:**		**Flying Ops (hours):**	Daily	Cumul.	**Pre Val (Hours):**	Daily	Cumul.	**Validation (Hours):**	Daily	Cumul.
			0	360			470			110
**Survey Team**	**N°**	**GPS / Tides (Hours):**	Daily	Cumul.	**Planning (Hours):**	Daily	Cumul.	**Other (Hours):**	Daily	Cumul.
	2		20	607			175			230

Equipment / Logistics (to include aircraft and airborne system defects / changes)

Remarks : Shut down office and Load LCL for Transit

Laser SRL No: 2001 **Green RX SRL No:** 2002

Narrative: (Customer/Personnel related issues & general comments)

Operations completed with the exception of the recovery of the BMTGs which will happen tomorrow. Office Shut down.
NAT, KJW departed field.

24hr Forecast: LCL depart for US, Logistics / Engineering team depart Galway, Tides party recover BMTGs from Donegal Bay.

Annex M Digital Aerial Mosaics

During flying operations digital photographs were taken at 1 Hz (1 second) using a downward looking geo-referenced 2 mega pixel digital camera. These images were then processed to provide 40cm per pixel resolution. Full coverage along the coastal areas was achieved.

Images captured along the coastline were then joined together using a semi-automated processing technique to provide a total series of forty seven 8 km tiled aerial mosaics covering the coastlines for Blacksod Bay, Donegal Bay, Galway Bay, Lough Foyle and Tralee Bay. These tiled mosaics have been provided digitally.

The aerial mosaics were then combined in ArcGIS to produce an overview mosaic for each survey area. These are provided digitally and presented in this annex.

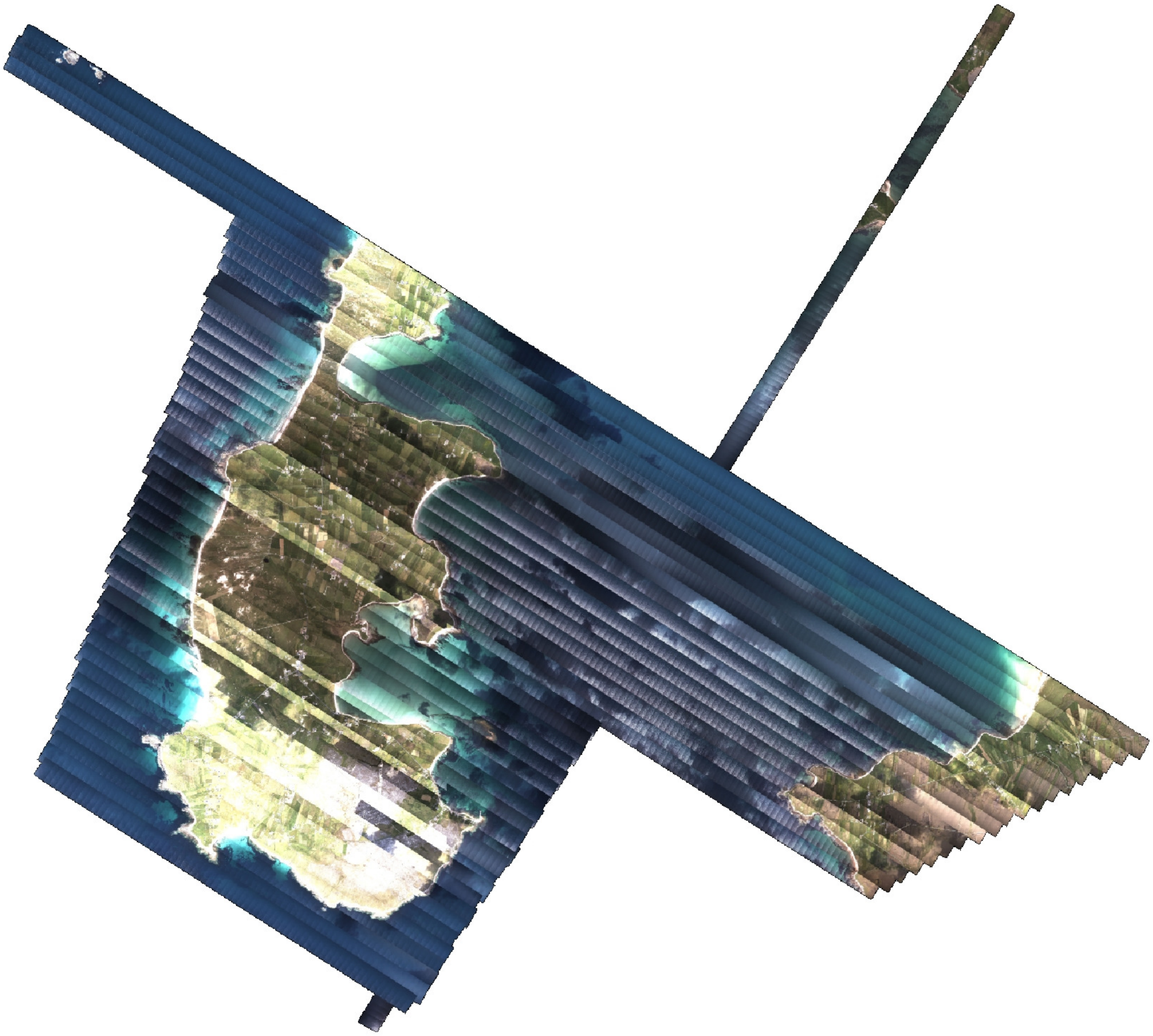

Blacksod Bay

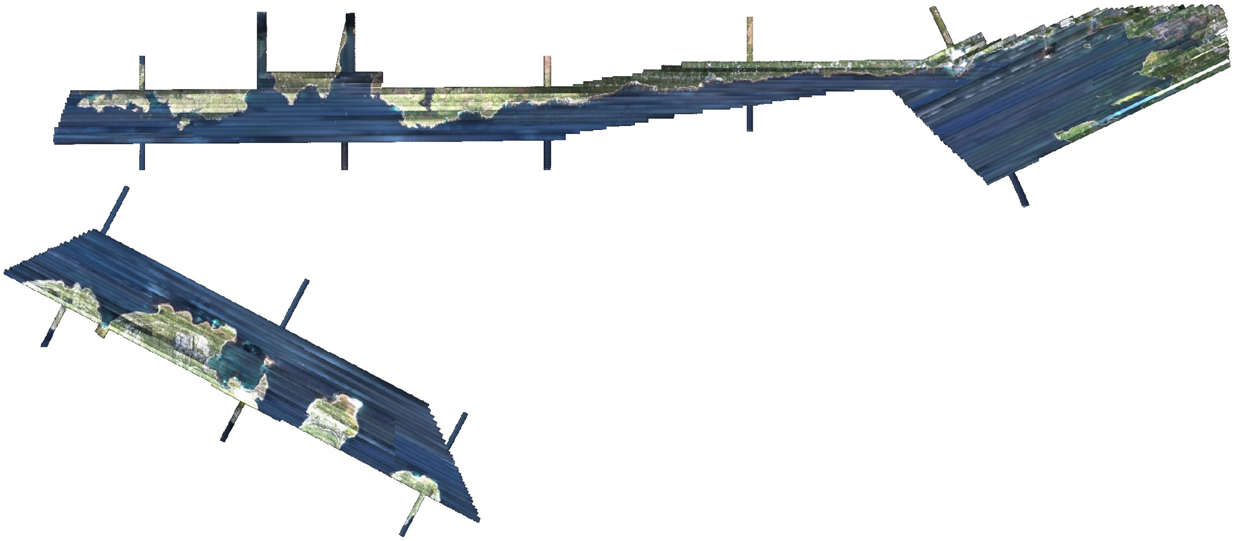

Galway Bay

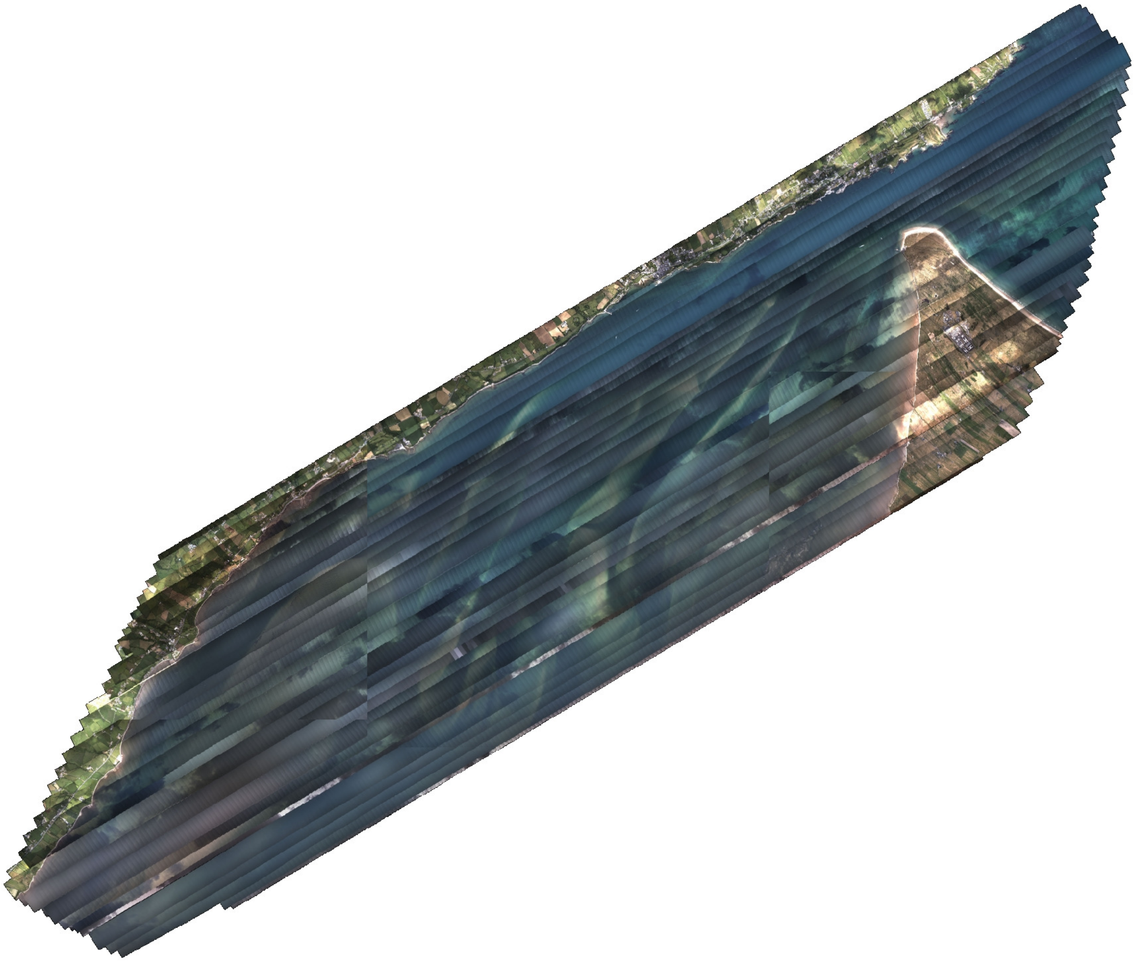

Lough Foyle

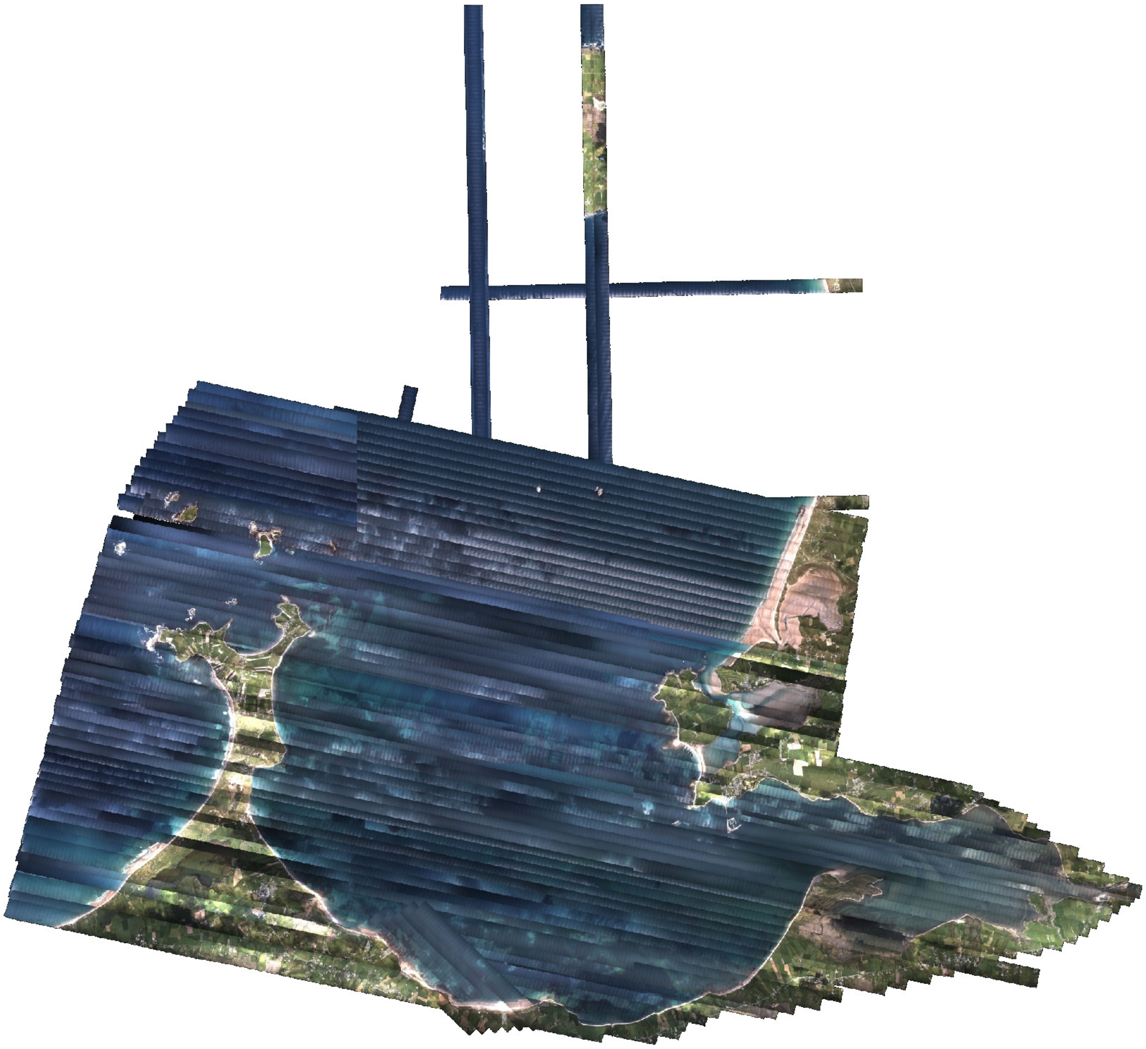

Tralee

Annex N. Hawkeye II Sensor and Aircraft

N.1 Hawkeye II Specification

The following description of the Hawkeye II system is taken from the BLOM Aerofilms tender documents.

Two lasers are used in the Hawk Eye II system. These are a topographic laser system (1064nm) running at 64kHz and a hydrographic laser system running between 1 - 4kHz with light of two different wavelengths being transmitted; green (532nm) and infrared (1064nm). In the hydrographic system the infrared laser light is reflected at the water surface whereas the green laser light proceeds into the water column. The green laser light is then reflected in the water column and at the seabed. A fraction of the reflected light reaches the Hawk Eye II receivers. The signal from the receiver is shown as a pulse response graph inside the "Pulse Response" boxes of the figure below.

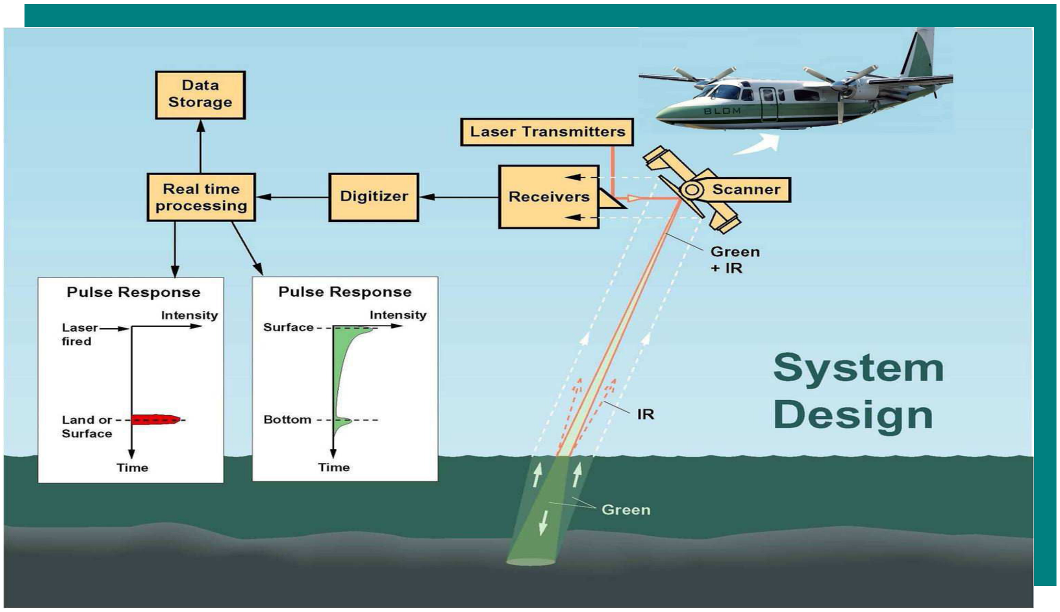

Figure 1: Dual Laser System

In the topographic system, the second infrared laser running at 64 kHz, scans the land, foreshore and any exposed land of the inter-tidal zone of the coastal margin. All land objects above the water reflect the laser beam including vegetation, structures and other features. A fraction of the reflected light also reaches the Hawk Eye II receivers. Each hit from the beam will be recorded as an object and the position will be stored together with information on its characteristics.

Key specification of the Hawkeye II system are listed in Table 1 below:

Parameter	Specification
Aircraft	Rockwell Commander 690A
Aircraft Transit Speed	170 Knots
Aircraft Range	Up to 1050 nautical miles
Aircraft Endurance	Typically 4 hours on task
Survey configuration	Altitude 200 – 400m dependent on swath, 400m for INFOMAR requirement<br>Speed 130-150kts dependent on spot spacing. 150kts for INFOMAR requirement.
Scan Pattern	Elliptical,<br>Constant angle set to 15-20° for normal operation dependant on swath. 20° for INFOMAR requirement.
Area coverage	10 km2 for INFOMER requirement
Operational capability	Full day and night operation
Airborne Survey Crew	1 Pilot, 1 operator
Airborne system	Single sensor unit, with separate cooling unit. Operator controls via laptop.
Depth Range	To 70m dependant on water clarity
Pulse rate, topographic laser	8000 pulse per second (8 pixels per return)
Pulse rate, hydrographic laser	1000 pulse per second (4 pixels per return)
Horizontal accuracy (refer note 1 and 2 appendix E)	5m (95% CEP) Bathymetry<br>0.5m (rmse) Topography
Vertical accuracy (refer note 1 and 2 appendix E)	0.5m Bathymetry<br>0.25m Topography
Object Detection	IHO Order 1 dependent on water clarity and sounding density.
Positioning and navigation	Applanix POS AV 410 GPS/IMU
Integrated digital camera system	uEye UCGA 2 MPixel. Final georeferenced digital mosaic with resolution <40cm per pixel
Reflectivity	0 - 255 value for per pulse reflectivity

Table 1: Hawkeye II ALB specification

N.2 Hawkeye II Flight operations

During the first survey sortie to each area, the cross tie lines were surveyed and main survey lines were sounded. During the subsequent sorties the cross ties were resurveyed and main survey lines sounded.

Automatic data processing to generate point cloud data occurs immediately following data collection. Data processing was initially conducted using no tidal model; observed tides were reapplied when received.

N.3 Hawkeye II Processing

The following description of the Hawkeye II Processing methodology is taken from the Hawkeye II tender documents.

Automatic data processing – generate point cloud

The first stage in the processing is the production of the aircraft trajectory from the airborne GPS data and the GPS data from the OSi Active Network base stations. The trajectory is processed using Applanix POSPac software. POSGPS is used for the production of the GPS positions and a second module POSProc is used to blend the inertial measurement using data and produce a final smoothed trajectory.

The next stage is the production of the laser point cloud using Coastal Survey Studio (CSS). This is a bespoke survey software package for the Hawk Eye II system.

In the post processing mode the trajectory data is combined with the scanning mirror data, timing information and laser ranges to generate a point cloud for each laser system. The result is a topographic laser point cloud and a bathymetric laser point cloud.

Ground survey data processing

The ground survey observations for the additional control points will be processed with the RTK GCA surveys. The data processing is carried out using Leica Geomatics Office (LGO) software. The observed static GPS data will be processed as a control framework to the OSi Active Network base stations. The RTK data will then be adjusted to the precise ETRF89 coordinates of the newly established base stations. The GCAs will be output in ASCII format suitable for input to the TerraSolid LiDAR processing software for QC of the land DTM data.

Vessel survey data processing

The SCA survey data from the single beam echosounder surveys is processed using Sonar XP software. The crossing lines are used to ensure data is well matched precise in the ETRF89 coordinate system. This results in soundings referenced in time and to the same precise ETRF89 surface of the bathymetric LIDAR point cloud. The soundings will also be output in ASCII format suitable for input to TerraSolid LiDAR processing software for QC of the seabed DTM data.

Laser data processing and checking

During the laser data processing the hydrographic and topographic laser data are kept separate. Once completed, the datasets can be combined together if required.

Having processed the data through CSS the next stage is to ensure that the laser point cloud is correctly aligned. This is achieved by identifying and then correcting for the heading, roll and pitch parameters present in the system. Using the TerraMatch software which runs within the MicroStation environment it is possible to identify the misalignments within a calibration area (using cross lines) and then apply these results to the entire data set. The final point cloud is then checked against the ground and sea control areas (GCA's and SCA's). After comparison it is then possible to apply a further shift to the data to ensure specification is met.

The hydrographic and topographic data are cleaned using CARIS Hips and Terrascan respectively. In both instances the laser data is systematically checked and any erroneous points and noise "spikes" are removed.

Tidal reduction of soundings and quality control.

The hydrographic point cloud data is output in the format of time, easting, northing and depth.

The tide gauge data will be used in the CARIS post processing as ambient sea surface heights together with the tidal model of each area provided by GSI. The water depths of the point cloud will then reduced by the tidal data and the resultant soundings will be to the Local Chart Datum in each area.

N.4 LADS QC and data approval

N.4.1 Quality Control

On receipt of the BLOM Hawkeye II data in the Adelaide office the LADS Survey team undertook a comprehensive quality review of the data to ensure it meet the required standards.

This process involves visualising data in other software packages to identify anomalies or inconsistencies. Quality Control (QC) is area based and will be conducted in manageable stages for each survey area. QC identifies anomalies or inconsistencies between lines.

The Easting, Northings and Depth were extracted from the BLOM raw data files for the Malin Head datum reference and used to build a LADS QCTools database, such that soundings could be identified by position and run number.

Contours, 3D tinned images and gridded difference plots (10m grid cells colour banded with difference between deepest and shoalest sounding) were generated to highlight anomalous soundings, datum differences between runs and excessive noise spread.

QC reports were generated and forwarded to BLOM highlighting data that it was felt required review. Resulting from this review new data sets were created and subject to renewed QC analysis. This was an iterative process with several rounds conducted for each data set before acceptable BLOM Hawkeye II data was received.

N.4.2 Datum Checks

Fledermaus and CARIS were used to check that the survey data matched the control area surfaces. The raw BLOM data was imported for both the Malin Head and LAT datums and spatial images of the differences between the two datums were created and interrogated to confirm the differences were correct. This analysis was performed on both the control areas and survey data. LAS files for both datums were also imported and checked for conformity. Again this was was an iterative process with several rounds conducted for each data set before acceptable BLOM Hawkeye II data was received.

Annex O Relative Reflectance Data

O.1 Relative Reflectivity

Relative reflectivity data is a measure of the reflectance of the seabed in a single wavelength (green/blue 532nm). The reflectance value for each pulse is calculated by determining the ratio between the transmitted laser pulse energy and the compensated returned laser energy. The numerical values for the relative reflectivity are scaled logarithmically to an 8-bit integer range 0 - 255.

Once a relative reflectivity value is calculated, further statistical cleaning to remove outliers is completed. Because the dataset is of relative reflectivity rather than an absolute value for each point, the entire dataset is scaled to ensure the full dynamic range is used over the dataset. This scaling is applied over an entire survey area to ensure consistency of the dataset.

The reflectivity data is treated as being relative. By treating the data as relative, the model is less sensitive to absolute values of water clarity, sea surface conditions and sensor operating parameters.

O.1.1 Reflectance Generation

Relative reflectivity data was generated using the RR model in the Ground System for the main survey areas to match the fairhsheet layout, namely Lough Folye (Sheet 1), Blacksod Bay (Sheet 2), Tralee Bay (Sheet 3), Galway Bay (Sheets 4 – 5), Aran Islands (Sheet 7) and a combined Galway Bay and Aran Islands sheet (Sheet 8). All the accepted lines except the cross lines were used in the calculations and then exported in ASCII files with the suffix RR1. The file format consists of the beam footprint position in easting and northing and then a reflectivity value (0 - 255).

The data was then imported into Fledermaus for visualisation. The images below show the relative reflectance for each area in sheets 1 to 8.

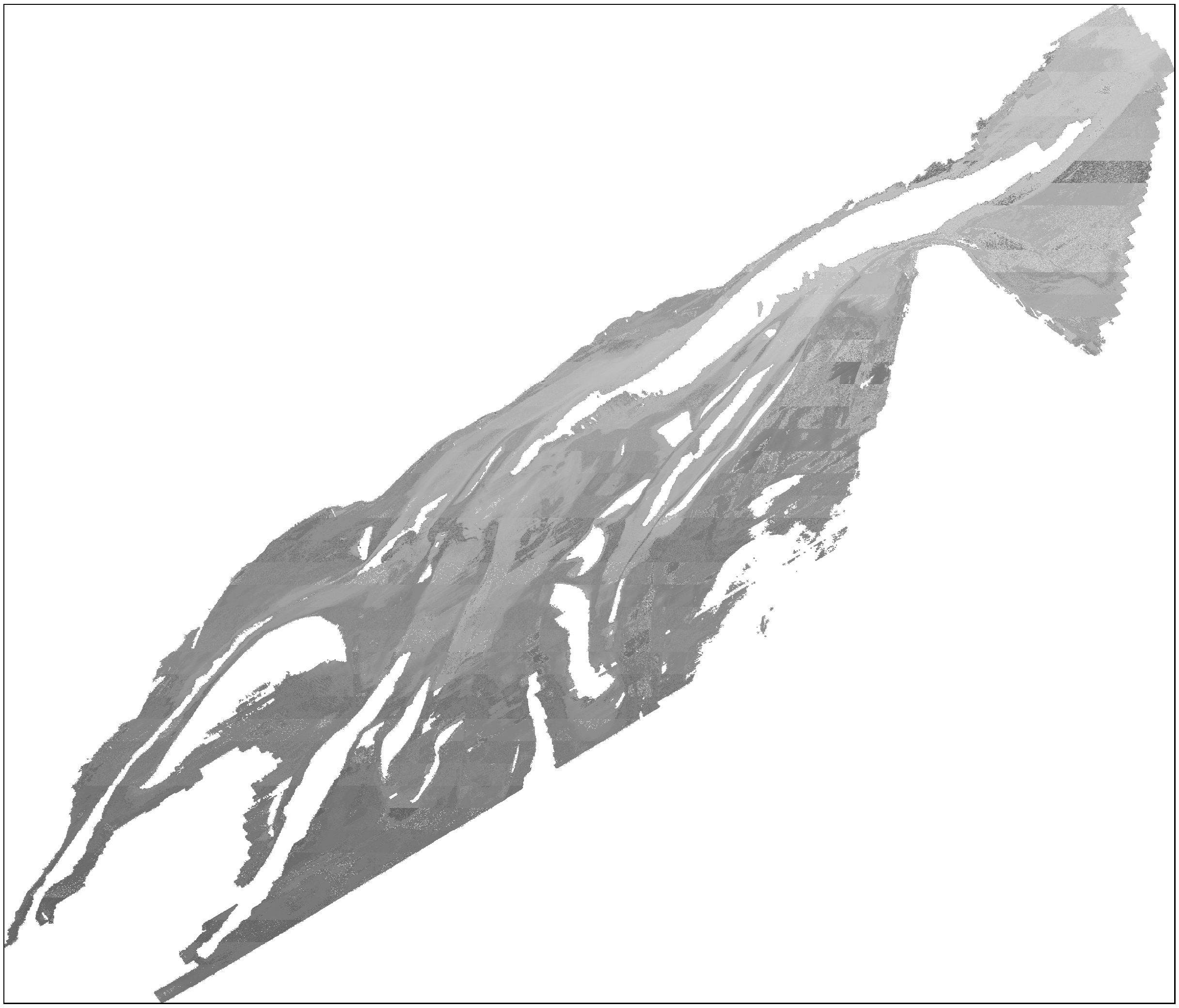

Fig 1 lough Foyle Sheet No LADS_01

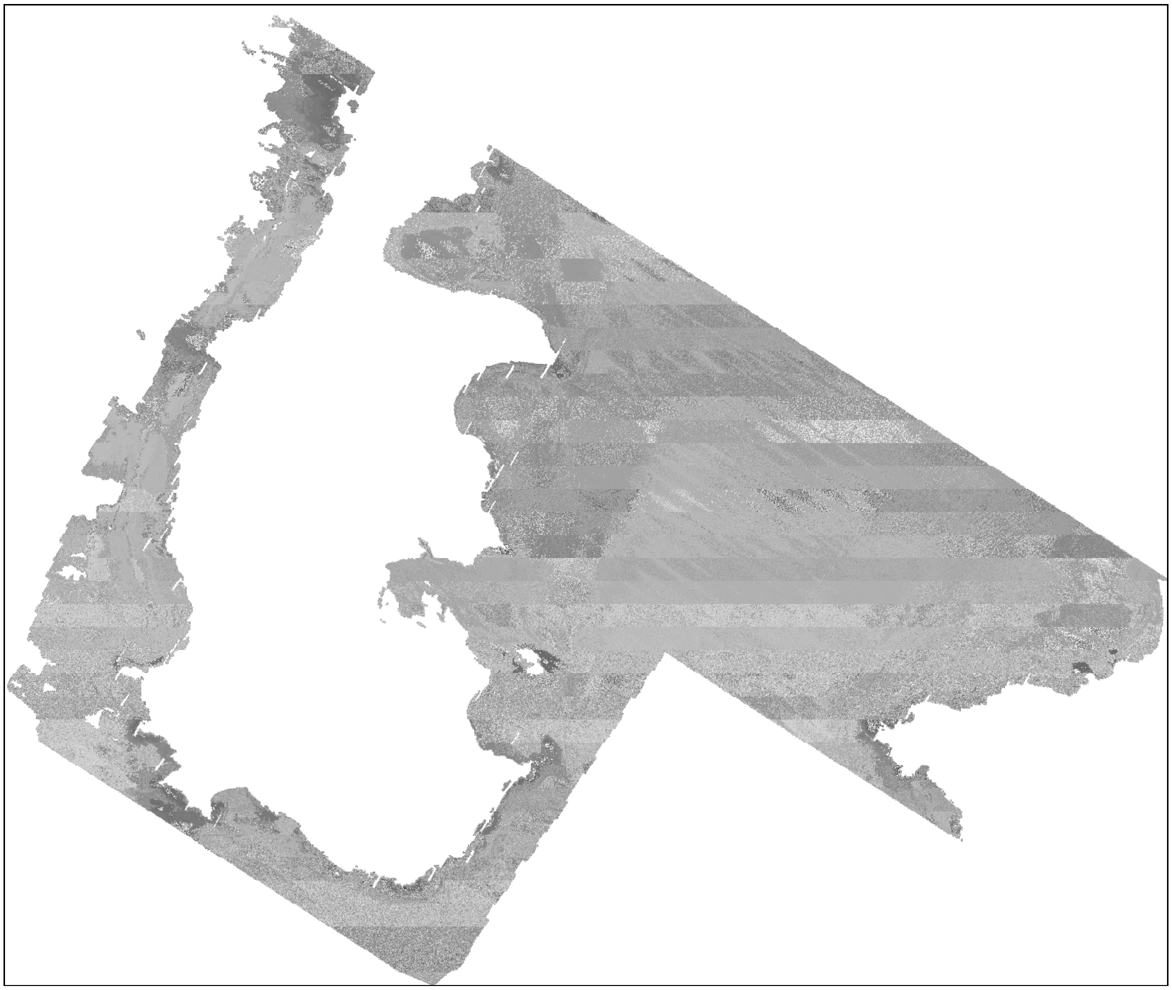

Fig 2 Blacksod Bay Sheet No LADS_02

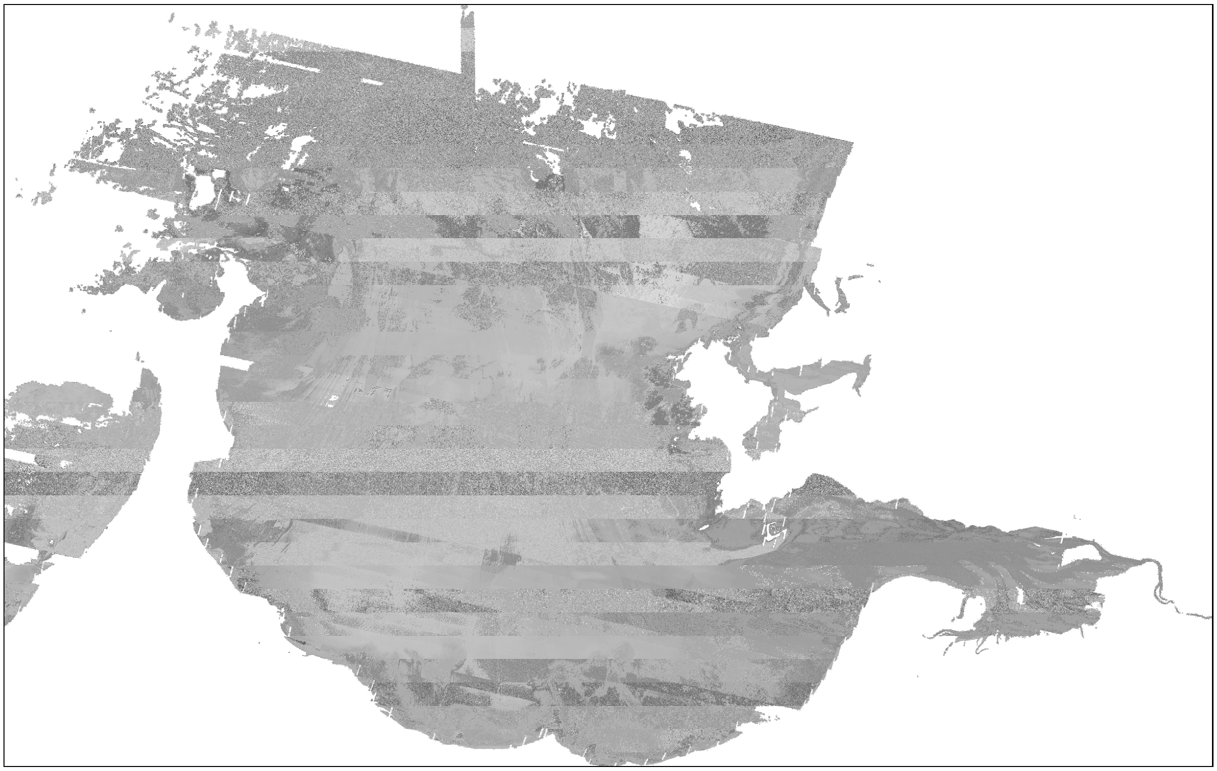

Fig 3 Tralee Bay Sheet No LADS_03

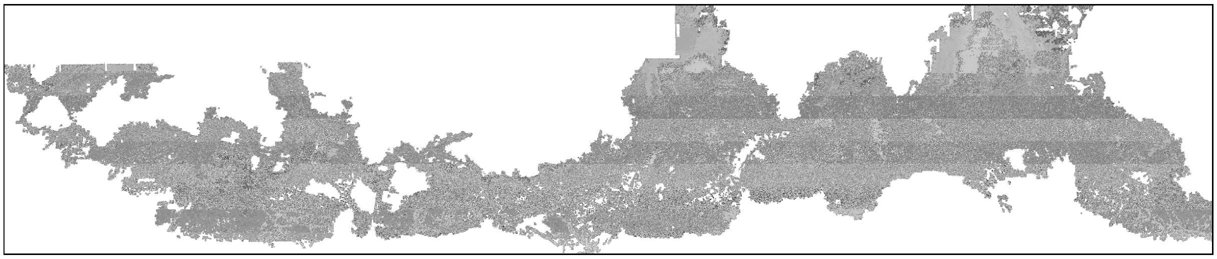

Fig 4 Galway Bay Sheet No LADS_04

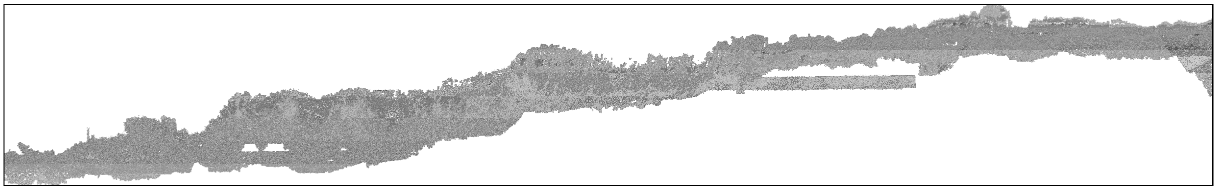

Fig 5 Galway Bay Sheet No LADS_05

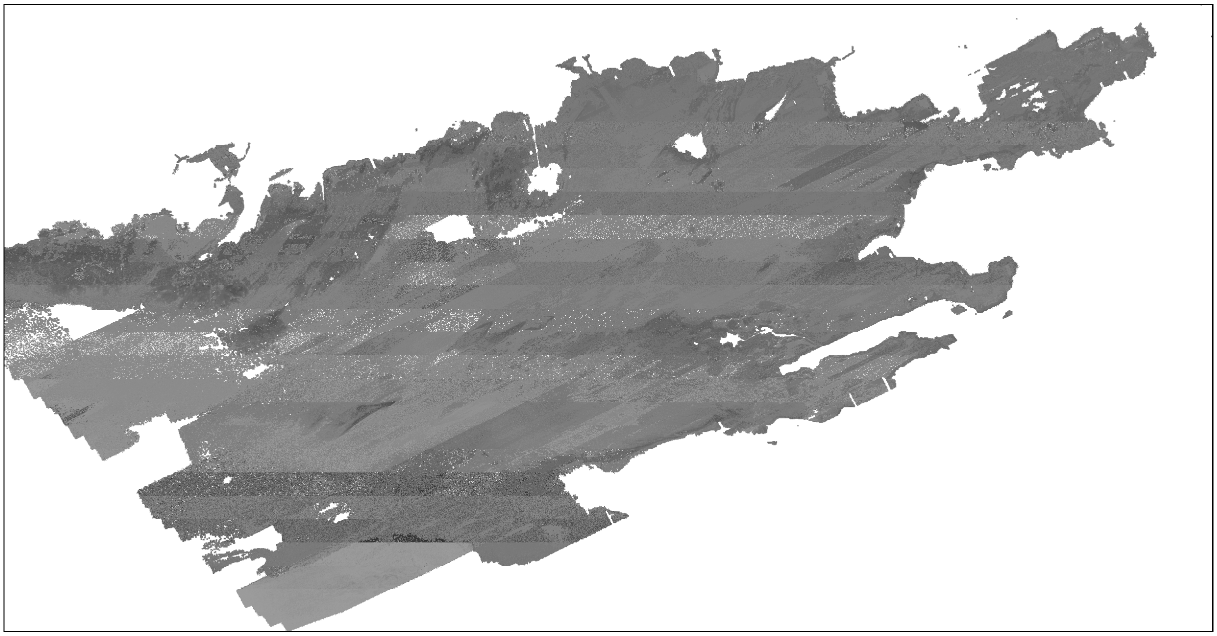

Fig 6 Galway Bay Sheet No LADS_06

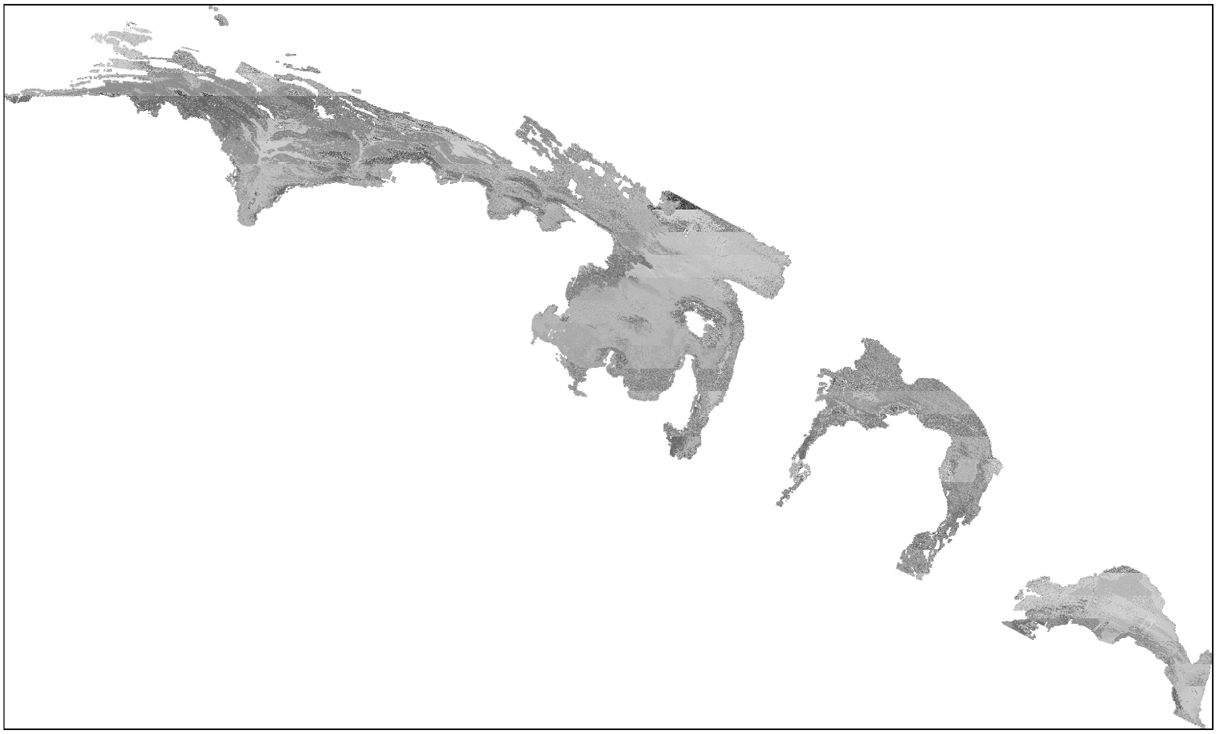

Fig 7 Aran Islands Sheet No LADS_07

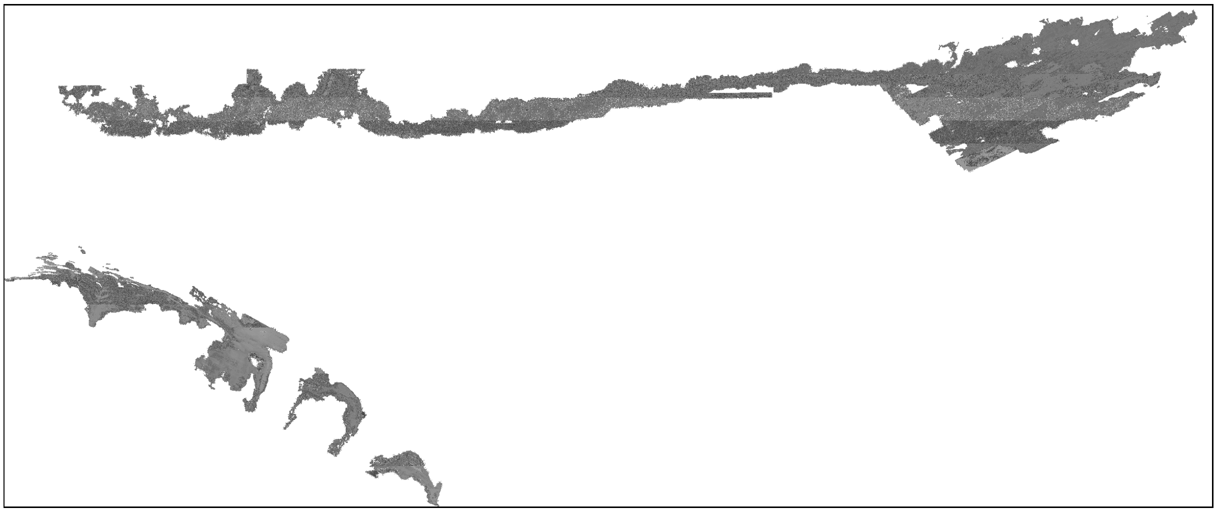

Fig 8 Galway Bay / Aran Islands Sheet No LADS_08
